# Characterizing Electrode Materials and Interfaces in Solid-State Batteries

**DOI:** 10.1021/acs.chemrev.4c00584

**Published:** 2025-02-04

**Authors:** Elif Pınar Alsaç, Douglas Lars Nelson, Sun Geun Yoon, Kelsey Anne Cavallaro, Congcheng Wang, Stephanie Elizabeth Sandoval, Udochukwu D. Eze, Won Joon Jeong, Matthew T. McDowell

**Affiliations:** † G. W. Woodruff School of Mechanical Engineering, Georgia Institute of Technology, Atlanta, Georgia 30332, United States; ‡ School of Materials Science and Engineering, Georgia Institute of Technology, Atlanta, Georgia 30332, United States

## Abstract

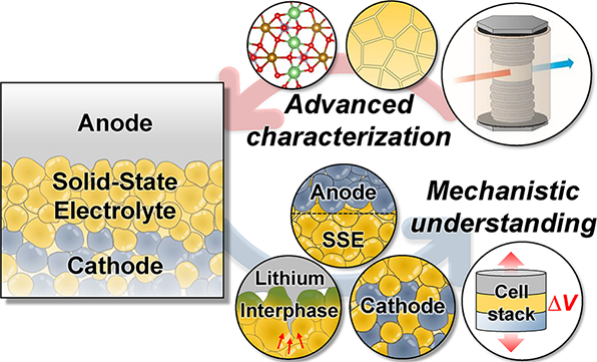

Solid-state batteries (SSBs) could offer improved energy density and safety, but the evolution and degradation of electrode materials and interfaces within SSBs are distinct from conventional batteries with liquid electrolytes and represent a barrier to performance improvement. Over the past decade, a variety of imaging, scattering, and spectroscopic characterization methods has been developed or used for characterizing the unique aspects of materials in SSBs. These characterization efforts have yielded new understanding of the behavior of lithium metal anodes, alloy anodes, composite cathodes, and the interfaces of these various electrode materials with solid-state electrolytes (SSEs). This review provides a comprehensive overview of the characterization methods and strategies applied to SSBs, and it presents the mechanistic understanding of SSB materials and interfaces that has been derived from these methods. This knowledge has been critical for advancing SSB technology and will continue to guide the engineering of materials and interfaces toward practical performance.

## 1 Introduction

Solid-state batteries (SSBs) are an emerging energy storage technology that may offer improved safety and energy density/specific energy compared to Li-ion batteries. SSBs do away with the flammable liquid electrolyte found in Li-ion batteries and replace it with a solid-state electrolyte (SSE) that conducts ions but not electrons. Interest in SSBs has increased in recent years due to multiple discoveries of new Li^+^-ion conducting materials. SSB architectures could enable the use of new, high-capacity electrode materials, since SSEs show different stability characteristics at interfaces. Inorganic SSEs are also single-ion conductors, which can promote high charge/discharge rates. SSBs can be more thermally stable than Li-ion batteries, and they can potentially enable new cell designs to further boost energy metrics. Although SSBs show great promise, challenges in their development have included accelerated cell degradation, uncontrolled interfacial evolution, and reliance on elevated temperatures or stack pressures to enable operation.

A key factor in the research and advancement of SSB materials and technologies over the past 10 years has been the characterization of materials and interfaces within SSB cells. Imaging, scattering, and spectroscopic techniques have been developed or used to gain substantial new understanding of how the materials and interfaces within SSBs evolve and degrade during cell cycling. Characterization of SSBs is different than liquid-electrolyte batteries–interfaces are buried within SSBs, and the lack of a liquid electrolyte alters some characterization strategies. Importantly, worldwide efforts in characterizing SSBs have revealed critical mechanistic differences when comparing SSBs to liquid-electrolyte batteries, and this knowledge has guided work toward engineering the electro-chemo-mechanical properties of electrode materials, SSEs, and interfaces toward improved performance and longevity.

The goal of this review paper is to present the *ex situ*, *in situ*, and *operando* characterization methods that have been developed and used to investigate SSBs, and to discuss our current understanding of the material and interfacial mechanisms governing SSB operation that has been gained by using these methods. Throughout, we compare SSB materials and interfaces to those in liquid-electrolyte batteries, both in terms of characterization strategies and materials mechanisms.

The paper is organized in the following manner. Section 2 presents an overview of the potential benefits of SSBs, based on the materials and cell designs used. Section 3 introduces various classes of SSEs, along with their electrochemical and interfacial behavior, and then compares SSEs to liquid electrolytes. Section 4 is a comprehensive discussion of characterization techniques that have been used for SSBs. Section 5 introduces the fundamental mechanisms that govern the behavior of lithium (Li) metal anodes in SSBs, as characterized using a wide variety of methods. Section 6 presents related mechanistic understanding for alloy anodes for SSBs, and Section 7 discusses characterization of composite cathodes in SSBs. Together, these sections comprise a comprehensive overview of characterization strategies and the fundamental understanding that has been gained on SSBs by using these techniques.

## 2 Potential Benefits of SSBs

SSBs offer potential advantages over current Li-ion battery technology, including improved energy density/specific energy, the capability for advanced cell/pack designs, and improved safety characteristics. The use of SSEs opens new possibilities for advancement of novel electrode materials and battery pack assembly, and it arguably mitigates some of the safety risks of conventional liquid electrolytes. While challenges still exist, much progress has been made in the development of SSBs with long-term cycling performance and superior capabilities compared to state-of-the-art Li-ion batteries.

### 2.1 Higher Energy Density Enabled by New Electrode Materials

The use of SSEs allows SSBs to potentially take advantage of electrode materials with higher ion storage capacity, which would result in higher energy density and/or specific energy than Li-ion batteries. Compared to liquid electrolytes, SSEs can feature more limited solid-electrolyte interphase (SEI) growth when used with reactive and large-volume-change materials such as Li metal. Incorporation of such electrode materials would result in more compact and lighter batteries, which is important for mobility applications.

Lithium metal has long been sought as an anode material for Li-ion batteries due to its high theoretical capacity (3860 mAh g^–1^) compared to that of graphite (372 mAh g^–1^).^1,2^ Lithium was used as the anode in early Li-ion batteries, but it was abandoned due to its tendency to form metal filaments and to react with the liquid electrolyte, causing short circuits and reducing Coulombic efficiency (CE).^3^ Because of the solid nature of SSEs, they are thought to enable improved control over the deposition and stripping of Li (although filamentary growth can still be an issue). If Li metal anodes could be incorporated into SSBs, the cells could exhibit up to 80% higher energy density compared to typical Li-ion batteries.^4^

Alloy anodes are another promising material for solid-state batteries.^5^ Alloy anodes form Li-rich compounds and avoid the issue of Li dendrite formation that plagues Li metal anodes.^5^ However, alloy anodes undergo significant volume changes during ion insertion/extraction. When used with liquid electrolytes, these volume changes can cause the SEI layer that forms on the material surface to break up each cycle, which leads to excessive SEI formation and rapid capacity decay. Recent work has shown that alloy anodes used in SSBs form more limited SEI since the SSE does not flow to contact new surfaces like liquids. High-capacity materials investigated as alloy anodes in solid-state batteries include Al (990 mAh g^–1^), Si (3579 mAh g^–1^), In (1012 mAh g^–1^), and Mg (3350 mAh g^–1^).^2,6^ It should be noted that the total energy of a cell is determined by its capacity multiplied by its voltage. Alloy anodes with higher potentials (or cathode materials with lower potentials) will slightly reduce the cell voltage and could reduce the overall cell energy density if the increase in specific capacity is not significant enough.

Other high-capacity materials also feature different reaction processes and potentially improved degradation behavior in SSBs. Sulfur (S) is an attractive cathode material due to its theoretical capacity of 1672 mAh g^–1^ and its high abundance.^7^ In liquid electrolytes, S cathodes suffer from the formation of soluble intermediate discharge products which results in the loss of active material from the cathode. In SSBs, S reacts with Li via a solid–solid reaction to directly form Li_2_S, which may extend cycle life. Moving to SSEs also provides the opportunity to design unique electrode microstructures with reduced tortuosity and enhanced rate capability, such as structured electrode composites and dense cathode structures.^8,9^

### 2.2 Pack-Level Advantages and Innovative Cell Designs

Beyond the cell level, the architecture of a battery pack is an important factor in determining the energy metrics of an energy storage system. A battery pack consists of multiple modules (each made up of multiple battery cells) and a protective enclosure protecting the battery cells. The battery pack delivers the required power for an application, and for electric vehicles the operation and thermal aspects of the pack are managed by a battery management system (BMS). While the BMS and associated thermal control systems (such as fluid lines) are critical for safe operation of Li-ion batteries, they add mass and volume to the energy storage system. Achieving high energy density at the pack level requires clever engineering to minimize these inactive components, such as the recent innovation in battery pack engineering by companies such as Our Next Energy and BYD.^10,11^

SSBs open new possibilities for battery cell and module designs that can improve energy density and specific energy at the pack level. SSBs can generally sustain higher operating temperatures than cells with liquid electrolytes,^12^ which lessens the need for thermal management systems within the pack, reducing pack weight and size. As a corollary, SSB cells may be able to effectively be used in larger form factors since heat buildup may not be as severe a safety issue as it is in Li-ion batteries. Larger individual cells further reduce inactive material use (like cell casings), again enhancing energy metrics. Note that a certain amount of stack pressure has been widely used to maintain interfacial contact in SSBs. Such stack pressures need to be approximately <1 MPa to avoid the use of bulky cell casings that would negate any energy density gains.^13^

**Figure 1:**
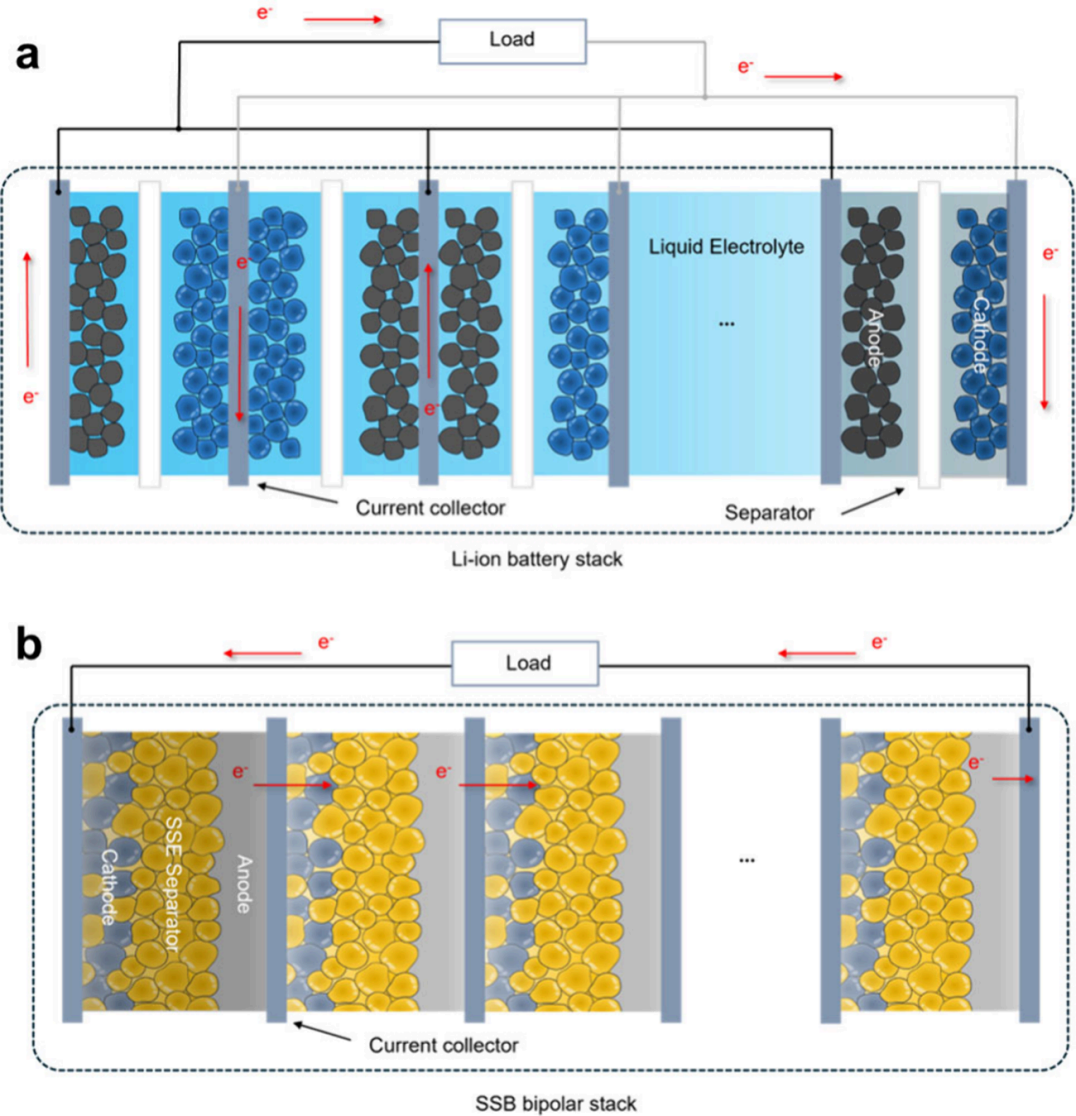
Electrode stacking in (a) a Li-ion cell and (b) a solid-state cell with bipolar stacking. In the SSB bipolar stack, the current collector (bipolar plate) is in contact with Li metal on one side and the cathode composite on the other.

Bipolar stacking is an alternative configuration of the electrodes within the cell that can further improve energy density/specific energy. Li-ion battery cells are generally manufactured in a stacked configuration with alternating layers of electrodes and separators (Figure 1a).^14^ Identical electrodes are coated on both sides of a current collector foil and wired via tabs at their ends. These electrodes are soaked in the liquid electrolyte that is shared by the whole cell. In a bipolar stacked cell, each negative electrode/SSE/positive electrode layer is connected to the next in series with a bipolar plate electrode (which also acts as the current collector) (Figure 1b), leading to high voltages and efficient use of materials.^15^

In a bipolar-stacked cell, the bipolar plate is coated with the negative electrode active material on one side and the positive electrode active material on the other. This means that the negative electrode of one layer stack has a direct electrical connection to the positive electrode of the next layer stack through the current collector. Bipolar stacking requires the prevention of ion flow between individual negative/positive electrode layers, which necessitates complex sealing for a battery using liquid electrolytes, adding to the cost and complexity of manufacturing.^15^ Since solid-state cells use SSEs, each layer stack can have its own isolated SSE separator, eliminating the risk of electrolyte leakage and simplifying cell construction. Bipolar stacking reduces the quantity of passive components including wiring and current collectors, allowing for higher overall specific energy and energy density at the cell and module levels.^16,17^ The shortened electrical connections in a bipolar stack cell also improve the power density and energy efficiency, while reducing thermal effects.^15,18^

While bipolar stacks operating from 6 to 12 V have been demonstrated in SSBs and quasi-SSBs,^19−21^ achieving high-performance bipolar stacking does have its challenges. For instance, ion flow between layers must be prevented, which makes preventing defects important. Since the anode and cathode are coated on either side of the same layer, the bipolar interlayer material must be compatible with both active materials and the electrochemical processes occurring within them. Additionally, since each layer is in series, the state of charge of each layer must be the same during cycling. Proper battery management systems will be important for monitoring individual cell voltages to increase longevity.

### 2.3 Safety

The flammable liquid electrolyte used in Li-ion batteries is a safety risk. Reports of fires and explosions involving Li-ion batteries have mounted in recent years as their use has expanded.^22^ Although the probability of thermal runaway and fire of Li-ion batteries is low if they are properly manufactured, handled, and managed, achieving maximal safety is important for electric mobility.

Under extreme conditions (overcharge, short circuit, and/or thermal abuse), a Li-ion battery can go into thermal runaway, which is a sequence of processes that results in the degradation of battery components, release of thermal energy, and buildup of gases within the battery casing/enclosure. At elevated temperatures, the constituents of the organic liquid electrolyte can react with released O_2_ (from breakdown of the SEI and decomposition of oxide-based cathode active materials such as LiCoO_2_ or LiNi_*x*_Co_*y*_Mn_*z*_O_2_).^23^ This generates even more heat that accelerates reactions and can generate toxic gases (e.g., CO) and cause swelling. This process can quickly lead to a fire or explosion, especially if the battery enclosure is damaged and atmospheric O_2_ makes its way into the cell.

SSEs can overcome some of these challenges because their solid nature makes them generally less flammable compared to the common solvents used in liquid electrolytes. The higher vapor pressure of liquids compared to solids makes liquids more volatile, and in the case of typical solvents used in liquid electrolytes, more susceptible to combustion if near an ignition source. The use of SSEs can also mitigate some of the previously mentioned triggers of thermal runaway in Li-ion batteries. An SSE separator may be a more robust barrier to dendrites and other causes of short circuiting (although this has not been proven). As previously mentioned, SSEs can also operate over wider temperature ranges, which means they are more likely to survive thermal abuse.

However, thermal runaway can still occur in SSBs. Rui et al. investigated the thermal stability of four sulfide SSEs (Li_3_PS_4_, Li_7_P_3_S_11_, Li_6_PS_5_Cl, and Li_10_GeP_2_S_12_) within cathode composites containing NMC811 and found that the total heat generation during the thermal stability tests was more than four times greater for the SSB electrodes compared to conventional electrodes with a liquid electrolyte.^24^ However, a higher onset temperature was observed for the first exothermic reactions in the SSB cathode composites compared to liquid electrolyte cathodes (>280 °C vs 220.8 °C). Accelerating rate calorimetry (ARC) has shown that various oxide SSEs release O_2_ during thermal decomposition, which can react exothermically with Li.^25^ It should be noted that these studies solely looked at thermal abuse as the trigger for thermal runaway within SSBs, and further work is needed to quantify how other triggers may influence safety characteristics.

## 3 Interfaces in Li-Ion vs Solid-State Batteries

### 3.1 Solid-State Electrolyte Materials and Interphase Formation

#### 3.1.1 Introduction to Li^+^-Conducting SSEs

**Figure 2:**
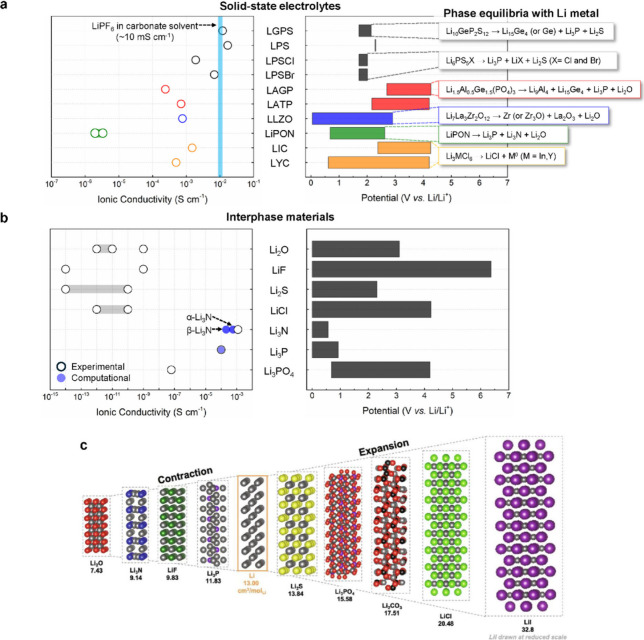
(a) Li^+^ ionic conductivities and electrochemical stability windows of various SSEs at room temperature: LGPS (Li_10_GeP_2_S_12_),^39,109^ LPS (Li_7_P_3_S_11_),^32,109^ LPSCl (Li_6_PS_5_Cl),^109,110^ LPSBr (Li_6_PS_5_Br),^110,111^ LAGP (Li_1+*x*_Al_*x*_Ge_2–*x*_(PO_4_)_3_),^59,109^ LATP (Li_1+*x*_Al_*x*_Ti_2–*x*_(PO_4_)_3_),^58,109^ LLZO (Li_7_La_3_Zr_2_O_12_),^50,109^ LiPON,^109,112^ LIC (Li_3_InCl_6_),^67,75,76^ and LYC (Li_3_YCl_6_).^66,75,76^ The ionic conductivity of a typical liquid electrolyte (LiPF_6_ in carbonate solvents) is denoted as the blue line for comparison. The chemical reactions to the right show the phase equilibria after reaction with Li metal; these phases may exist in the interphase. The phase equilibria shown in (a) only include the product species without considering the stoichiometry of reaction with Li metal. (b) Ionic conductivity and electrochemical stability window of typical interphase materials generated between Li metal and SSEs: Li_2_O,^113−115^ LiF,^114,116,117^ Li_2_S,^114,118^ LiCl,^114,119^ Li_3_N,^114,120,121^ Li_3_P,^114,122,123^ and Li_3_PO_4_.^124,125^ (c) Crystal structures and calculated molar volumes of Li metal and inorganic Li compounds. Reproduced with permission from ref (126). Copyright 2022 Elsevier.

SSEs with a variety of chemical compositions have been developed. Most efforts have been focused on developing SSEs for Li^+^ conduction, while SSEs for other ions (such as Na^+^) have seen less focus. SSEs function as both a separator and an electrolyte if used as a component within a composite electrode, and this inherently involves solid–solid contact at the SSE–electrode interface. Interfacial degradation mechanisms when using SSEs tend to be different than when using liquid electrolytes because of this solid–solid contact.^5,26,27^ While high ionic conductivity comparable to commercial liquid electrolytes (∼10 mS cm^–1^ at room temperature, Figure 2a) is a primary consideration for evaluating SSE materials,^28,29^ other properties, such as electrochemical/chemical stability, mechanical properties, structural and morphological effects, and processability, must also be taken into account when selecting an SSE.^26,30,31^ In this section, we will briefly introduce the well-known Li^+^-conducting SSEs, focusing on ionic conductivity and other notable features. The interfacial characteristics of SSEs, including interphase formation, are discussed in later sections.

##### 3.1.1.1 Sulfide SSEs

Sulfide SSEs contain S anions and can be categorized based on their structures, such as glassy and crystalline.

Most glassy sulfides consist of Li_2_S and P_2_S_5_ in the form of (100-*x*)Li_2_S-*x*P_2_S_5_ (abbreviated LPS), as well as their derivatives. These materials can include some partially crystallized structures, such as Li_3_PS_4_ and Li_7_P_3_S_11_.^32,33^ The ionic conductivity of the glassy sulfide SSEs covers a broad range (10^–7^–10 mS cm^–1^) with low activation energies (0.15–0.35 eV).^29,30^ The Li_2_S–P_2_S_5_ glass–ceramic conductor attained an ionic conductivity of 17 mS cm^–1^ by forming a partially crystalline structure, Li_7_P_3_S_11_, through densification with heat treatment up to 300 °C.^32^

The crystalline sulfides include Li argyrodites and thio-LISICON (Li Super-Ionic CONductor) materials. The argyrodite SSEs, with the general formula Li_6_PS_5_X (LPSX, where X = Cl, Br, or I), are of particular interest due to their relatively high ionic conductivities (>1 mS cm^–1^),^34−36^ good interfacial stability, and potential viability for commercial production.^37,38^ Another class, Li_10_MP_2_S_12_ (M = Si, Ge, Sn), is termed the thio-LISICONs and also exhibits high ionic conductivity.^28,39^ For instance, Li_10_GeP_2_S_12_ (LGPS) was reported to have an ionic conductivity of 12 mS cm^–1^ at room temperature (Figure 2a),^39^ and this family generally displays relatively high ionic conductivities ranging from 1 to 10 mS cm^–1^ with low activation energies of 0.2–0.3 eV.^28^ Numerous studies have investigated structure–property relationships in these crystalline sulfide SSEs, including aliovalent or halide substitutions to enhance the dimensionality of Li conduction pathways or to increase carrier density.^28^ These efforts have led to remarkable improvements in ionic conductivity, such as Li_9.54_Si_1.74_P_1.44_S_11.7_Cl_0.3_ (25 mS cm^–1^) and Li_5.5_PS_4.5_Cl_1.5_ (9.4 mS cm^–1^).^40−42^

Sulfide SSEs typically exhibit lower hardness and stiffness than oxide SSEs, with the elastic modulus of LPS and LGPS being less than 50 GPa.^30,43,44^ This feature allows densification along with the formation of intimate connections between SSE particles through cold pressing, enabling facile material processing.^30,32^ However, sulfide SSEs often show poor air stability arising from hydrolysis reactions when in contact with moisture, resulting in the creation of toxic H_2_S gas.^45,46^ As a result, sulfide SSE processing requires a dry atmosphere, which may be a bottleneck for commercial manufacturing. While a standard dry room operates at a dew point of −40 °C, this may not be sufficient to prevent sulfide electrolytes from decomposing. A recent study by Scharmann et al. concluded that a dew point temperature lower than −45 °C is useful for handling sulfide electrolytes.^47,48^ In addition, poor electrochemical/chemical stability of some sulfides in contact with electrode materials degrades their performance in SSBs, as discussed in later sections.

##### 3.1.1.2 Oxide SSEs

The family of oxide SSEs includes Li-garnet, Li -NASICON (Na Super-Ionic CONductor), perovskite, and LISICON materials, again defined by their crystal structures.^30,49^ Among these SSEs, only a few are known as fast Li^+^ ionic conductors with room-temperature ionic conductivity comparable to liquid electrolytes. Some notable oxide SSEs are introduced as representative examples.

Li-garnet SSEs have a chemical formula of Li_5_La_3_M_2_O_12_, where M is Nb or Ta, and they can feature ionic conductivity up to 10^–3^ mS cm^–1^.^50^ Later, cubic-Li_7_La_3_Zr_2_O_12_ (LLZO) was discovered to have a much higher ionic conductivity (0.3–0.5 mS cm^–1^ at room temperature, Figure 2a).^50^ However, its ionic conductivity can range from 10^–1^ to 10^–4^ mS cm^–1^ and strongly depends on synthesis conditions.^51^ The chemical composition of the garnet SSE was also modulated with aliovalent doping in order to improve ionic conductivity, particularly with Ta and Ga elements, reporting ∼0.9 mS cm^–1^ (Li_6.75_La_3_Zr_1.75_Ta_0.25_O_12_) and ∼2.1 mS cm^–1^ (Li_6.55_Ga_0.15_La_3_Zr_25_O_12_).^29,52−55^ The garnet SSEs are unstable in ambient air since moisture and CO_2_ can react with the SSE surface to form a contaminant layer (LiOH and Li_2_CO_3_), which increases interfacial resistance in contact with electrodes.^56,57^

Li-NASICON SSEs are a subcategory of Li^+^-conducting SSEs in the NASICON (Na Super-Ionic CONductor) family of materials, which is based on the crystal structure of NaZr_2_(PO_4_)_3_.^28^ The general formula of Li-NASICON SSEs is LiM_2_(PO_4_)_3_, where the two M sites can be composed of various tri- and tetravalent ions, such as Al^3+^, La^3+^, Ti^4+^, and Ge^4+^. Typical examples are Li_1+*x*_Al_*x*_Ti_2–*x*_(PO_4_)_3_ (LATP) and Li_1+*x*_Al_*x*_Ge_2–*x*_(PO_4_)_3_ (LAGP), having ionic conductivities of 0.7 mS cm^–1^ (*x* = 0.3) and 0.4 mS cm^–1^ (*x* = 0.5), respectively (Figure 2a).^58,59^ These values are an order of magnitude lower than the typical sulfide SSEs, but aliovalent doping of Cr^3+^ was found to increase ionic conductivity of LAGP to 6.7 mS cm^–1^ (Li_1.5_Al_0.4_Cr_0.1_Ge_1.5_(PO_4_)_3_).^60^ Li-NASICON SSEs exhibit better stability in water and air than LLZO-based materials.^61,62^

Oxide SSEs typically feature wider electrochemical stability windows (Figure 2a) and higher mechanical rigidity (>50 GPa elastic modulus) compared to sulfide SSEs,^27,30,43^ which could be beneficial for their chemical and mechanical compatibility with electrode materials. Interfacial stability of specific cases is discussed in later sections. The chemical tunability of oxide SSEs allows for diverse substitutions and a deep understanding of the structure–transport relationships in this class of materials. However, oxide SSEs typically require high synthesis temperatures during densification/sintering.^30,63^ For example, LLZO powder undergoes cold-pressing followed by sintering at over 1000 °C, which is a challenge for compatibility with commercial manufacturing.

##### 3.1.1.3 Halide SSEs

Halide SSEs are distinct from sulfide and oxide SSEs. Their general formula is Li*_a_*MX_*b*_, where M is a metal element and X is a halide element (X = F, Cl, Br, or I).^64^ The metal element M can include those from group 3 (Sc, Y, La), group 13 (Al, Ga, In), and various divalent metal elements (e.g., Ti, Mn, Fe, Cu, Zn, Mg).^64,65^ Halide SSEs historically received less attention due to their low ionic conductivities (<1 mS cm^–1^) at room temperature. However, in 2018, Asano et al. first reported Li_3_YBr_6_ and Li_3_YCl_6_ (LYB and LYC, Figure 2a) which have ionic conductivities of 1.7 and 0.5 mS cm^–1^.^66^ Following this, Li et al. reported Li_3_InCl_6_ (LIC, Figure 2a) with an ionic conductivity of 1.5 mS cm^–1^.^67^ Since then, rapid advancements have been made in discovering halide SSEs with various chemical compositions and high ionic conductivity.^68−72^ Additionally, superior electrochemical stability of halide SSEs at high potentials (>4.0 V vs Li/Li^+^) has been demonstrated, suggesting good compatibility with 4 V-class cathode materials.^65,66,72^ The halide SSEs also exhibit relatively low hardness and stiffness compared to oxide SSEs (e.g., LIC has an elastic modulus of 34.2 GPa and a hardness of 2.0 GPa).^73^ This can be beneficial when using halide SSEs within cathode composites, as the halides can more readily accommodate the volume changes of cathode materials during Li cycling.^73,74^ However, poor stability with Li metal and the necessity of costly metals (In and Y) remain as bottlenecks for their commercial application, necessitating further exploration of new halide SSE materials for better compatibility with commercial SSB manufacturing.^72,75−77^

##### 3.1.1.4 Solid-State Polymer Electrolytes (SSPEs)

SSPEs were developed as an alternative to liquid electrolytes, offering enhanced safety by eliminating flammable solvents while providing robust mechanical support as separators.^78−80^ SSPEs have several advantages compared to inorganic SSEs, such as low cost, low mass density, improved electrochemical stability, and better mechanical conformity at the electrode interface, which have led to extensive research efforts in this area.^49,81^ SSPEs can be considered as a variation of liquid electrolytes, where Li salt is dissolved within dry polar macromolecules.^82^ This distinguishes them from gel polymer electrolytes, which are composed of liquid electrolytes and partially gelated polymers that provide dimensional stability.^82^

Since the discovery of Li^+^-conducting polyether (-(CH_2_–CH_2_–O)_*n*_-) electrolytes, many variations of solid polymer electrolytes have been developed by altering their polymer structures.^83−85^ However, the ionic conductivities of poly(ethylene oxide) (PEO)-based electrolytes, which are widely studied, are typically less than 0.1 mS cm^–1^ at room temperature.^81,84,85^ PEO electrolytes have a semicrystalline structure, and only the amorphous regions are favorable for Li^+^ conduction.^79,81,85^ This necessitates operating PEO-based SSPEs at elevated temperature over ∼60 °C, which is close to the melting temperature of PEO, to disrupt its crystallinity and promote segmental chain motion, thereby increasing Li^+^ mobility within the polymeric structure.^81,85,86^ Efforts to increase the ionic conductivity of SSPEs include adding plasticizers or optimizing the amount of Li salt, which can increase the free volume for segmental chain motions. However, these methods compromise other properties, such as mechanical strength and electrochemical stability, reducing compatibility with the Li metal anode.^79,81,87^ Alternative strategies to improve the ionic conductivity of SSPEs involve chemically modifying the polymer structure or introducing inorganic filler materials.^78,79,81,88^ Incorporating inorganic filler materials, such as metal oxides and LLZO, has gained significant attention in the context of polymer–ceramic hybrid composites.^81,89−92^ This approach can significantly improve the transference number (>0.5), which is lower when using SPEs alone (<0.4).^81,86,93^

The flexible and soft characteristics of SSPEs, with an elastic modulus often less than 5 GPa, have been emphasized for their better mechanical compatibility at the electrode interface and higher suitability for large-scale manufacturing processes compared to oxide or sulfide-based SSEs.^49,81,85^ Although SSPEs have wider stability windows in comparison to oxide and sulfide SSEs, they can still form interphases at the Li metal anode interface (e.g., Li alkoxide and anion-derived species).^94^ Similar to oxide and sulfide SSEs, SSPEs also suffer from the morphological instability of Li metal.^95,96^ In the case of PEO-based SSEs, poor oxidation stability above 3.8 V has been identified as a major issue for compatibility with cathode materials.^81,97^ Furthermore, both PEO and Li salts are hygroscopic, necessitating a controlled atmosphere during processing to prevent degradation.^94^

##### 3.1.1.5 Other SSEs

Other types of SSEs, such as oxyhalides and borohydrides, have also emerged as promising candidate materials due to their relatively high ionic conductivities. Oxyhalide SSEs include a divalent oxygen anion (O^2–^) in a halide anion-dominant framework, and they have been reported in various chemical compositions like LiNbOCl_4_, LiTaOCl_4_, and Li_1.75_ZrCl_4.75_O_0.5_.^98,99^ These materials have ionic conductivities that can exceed 10 mS cm^–1^ and can exhibit electrochemical compatibility with high-voltage cathode active materials.^98−100^ Hydride-typed SSEs have shown relatively low Li^+^ conductivities compared to other inorganic SSEs.^101^ However, polyhedral borohydrides have demonstrated high ionic conductivities at room temperature, including around 30 mS cm^–1^ for Na^+^ and 6.7 mS cm^–1^ for Li^+^,^102−104^ enabling their use in SSBs.^101^ Borohydride SSEs can be compatible with typical alkali metal anodes,^101,105^ while they are reactive with high voltage cathode active materials, resulting in capacity degradation.^106^ Despite their high ionic conductivities, a systematic understanding of their electrochemical and mechanical stabilities in conjunction with various electrodes is essential to fully leverage their potential in SSB applications.

Redox-active SSEs have also garnered interest. Work by Cui et al. demonstrated a homogenization strategy using the mixed-ion conductor Li_1.75_Ti_2_(Ge_0.25_P_0.75_S_3.8_Se_0.2_)_3_ as the cathode active material and catholyte simultaneously. This approach mitigates side reactions and improves electrochemical stability.^107^ Other work by Song et al. demonstrated approximately 80 mAh g^–1^ extra reversible activity with a Li_3_VCl_6_ catholyte through V^2+/3+^ redox activity when combined with an LFP cathode.^108^

#### 3.1.2 Electrochemical Stability Windows

Despite their promising ionic conductivity, most SSEs suffer from instabilities at electrode interfaces, including chemical instabilities (e.g., the formation of interphase layers) or mechanical instabilities (e.g., loss of physical contact at the interface). The formation of interphase layers can impact electrochemical processes at the interface between SSEs and both positive and negative electrode materials. Several reports clearly demonstrate that the properties of the interphase can dictate cell performance depending on the ionic or electronic conductivities of the newly formed materials in the interphase, even though the parent SSE may have outstanding ionic conductivity.^126−129^ Hence, the formation and electro-chemo-mechanical properties of interphases at SSEs are attracting substantial attention in the battery research community. This section describes general electrochemical stabilities of SSEs and the formation of interphase regions in contact with Li metal electrodes. Section 3.2 contains comparative information for liquid electrolytes.

The electrochemical stability windows of various SSEs have been investigated using density functional theory (DFT) computations (Figure 2a).^75,109,114^ Most SSEs have narrow electrochemical stability windows regardless of their ionic conductivities. The stability windows expressed with reference to the Li electrode potential show that virtually all inorganic SSE materials are thermodynamically unstable in contact with the Li metal electrode. This generally causes interphase formation at the Li metal–SSE interface. However, previous work has shown that this thermodynamic instability is not always directly linked to inferior cell performance; instead, knowledge of the interphase formation kinetics is required.^130−132^ The kinetic stability of the Li–SSE interface can be determined by the nature of the reaction products between Li metal and the SSE.

The interphase may include metastable phases,^127^ nanoscopic particles with incorporated interfaces, or other complex phase distributions. Figure 2b shows the ionic conductivities and electrochemical stability windows of thermodynamically predicted interphase compounds that can form due to the reaction of Li with various SSEs. Three aspects should be considered for evaluating these interphase compounds.

(1) These phases are generally expected to have thermodynamic stability against Li metal.
(2) The ionic conductivities of these interphase materials are generally inferior to those of the parent SSE. With a few exceptions, such as Li_3_N and Li_3_P, which exhibit ionic conductivity >0.01 mS cm^–1^, ionic conductivities are typically less than 10^–3^ mS cm^–1^.
(3) The electronic conductivities of these interphase materials are typically poor,^115,118,121^ but other electronically conductive species, such as reduced metals or Li alloys, can also be produced during interphase formation depending on the chemical structure of the parent SSE (Figure 2a).

Overall, the distribution of phases and chemical characteristics of the interphase strongly influence the reversibility of electrochemical reactions and the impedance of the interface.

In addition to chemical effects, interphase growth also has mechanical consequences in SSBs. Interphase growth at an SSE in contact with a Li metal electrode could be terminated at the nanometer scale but may also extend to the micrometer scale and beyond, depending on the SSE. The formation of the interphase itself involves a change of the (partial) molar volume of Li, resulting in volume change at the interface during this process. Figure 2c shows estimated molar volumes of Li compounds typically found in the Li–SSE interphase. Due to these volume changes, thicker interphase growth could induce substantial mechanical stress.^126^ The mechanical stress may either be mitigated or intensified at the SSE interface depending on the molar volumes of interphase products. In some cases, this may cause nonuniform stress distribution throughout the cell and accelerate detrimental cell failure, such as mechanical damage to the SSE.^127,130,133^ Furthermore, these interphase volume changes may either promote or reduce contact loss depending on whether there is local contraction or expansion. This mechanical effect of interphase formation must be included with other related factors, such as the conductivities of the interphase materials and the kinetics of interphase growth, for a comprehensive understanding of contact evolution at electrode/SSE interfaces. More details of interphase formation and growth at the Li metal interface are discussed in Section 5.5.

### 3.2 Contact Evolution and Interfacial Characteristics

#### 3.2.1 Liquid vs Solid-State Electrolytes

**Figure 3:**
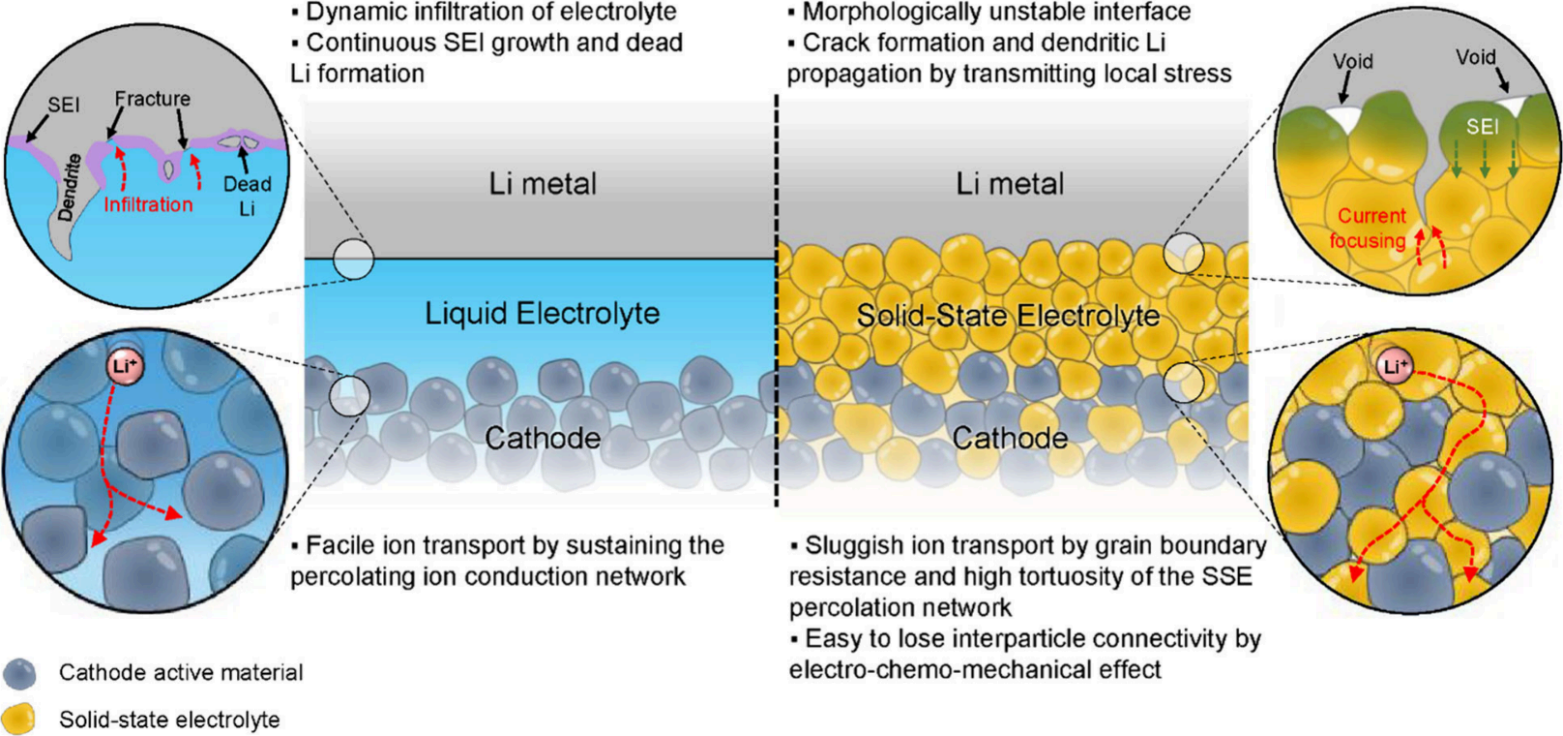
Schematic images of batteries with a liquid electrolyte (left) and a SSE (right), along with key differences at the anode and cathode interfaces shown in the magnified schematics and listed in the text.

Liquid electrolytes can flow readily and cannot sustain shear stress. They also typically exhibit dynamic wettability at the electrode/electrolyte interface since the high mobility of solvent molecules and dissolved salts allows for reorientation even within highly porous structures.^77,134^ This enables liquid electrolytes to infiltrate porous electrode microstructures and to accommodate structural and morphological changes of electrodes while maintaining contact at the interface (Figure 3). These characteristics are beneficial for maintaining percolating networks for ion conduction. However, as further discussed in Section 3.3, liquid electrolytes can be reduced or oxidized at electrode interfaces, with the reaction products, including inorganic Li compounds and organic species, precipitating at the electrode interface and forming a resistive solid-electrolyte interphase (SEI) layer. The flowability of liquid electrolytes allows them to dynamically contact internal porosity that may form within large-volume-change electrodes during cycling to form new SEI layers each cycle (Figure 3).^135−137^ This accelerates electrolyte consumption, causing continued growth of the resistive SEI and irreversible Li loss.^138^ For Li metal electrodes, this behavior also results in morphological instabilities, such as dendritic Li growth and Li isolation, which degrade cycle life (Figure 3).^135,138^ Thus, many high-capacity electrode materials (such as Li metal, alloy anodes, and conversion cathodes) have shown limited cyclability in liquid-electrolyte Li-ion batteries.

Unlike liquid electrolytes, SSEs have limited flowability due to constrained atomic motions within their crystalline or amorphous solid structures, preventing them from continuously wetting the electrode surface during electrode morphology changes. Voids and pores can be present or can form at the solid–solid electrochemical interface during cycling, which hinders transport and increases impedance due to current constriction effects (Figure 3).^26,27,77^ In general, sustaining intimate solid–solid contact during electrode morphological changes is difficult and is a key technical need for SSB development. Chemo-mechanics plays an important role via the transmission of stresses and strains across the interface, and both chemical and mechanical factors govern the formation of voids and contact evolution.^77,139,140^

Despite these contact limitations, the solid-state nature of SSEs has the benefit of not flowing into open porosity within an electrode to excessively form interphase layers, which may reduce the extent of interphase formation on large-volume-change electrodes and enhance cycling performance compared to liquid electrolytes. For instance, Li et al. compared cycling behavior of Li metal batteries with LiPON SSEs to those using a typical liquid electrolyte.^141^ The cycling performance of the liquid electrolyte system was shown to directly rely on the amount of electrolyte present, whereas the SSB showed outstanding cyclability over 10,000 cycles (90% retention with 99.98+% Coulombic efficiency). The cumulative capacity loss in the solid-state system was much lower than that in the liquid system, suggesting potential benefits of SSEs for battery longevity.

In addition to the aforementioned characteristics, electrochemical reactions in liquid electrolytes feature a strong dependence on interfacial charge transfer and bulk ion transport kinetics. These factors are generally influenced by the mobility of the molecules that make up the electrolyte. A typical example is the limitation of charge transfer and ion transport at low temperatures. Ionic motion can become sluggish at subzero temperatures due to solvent freezing, leading to large internal resistances and cell failure.^142−144^ Commercial electrolyte formulations, such as LiPF_6_ in carbonate solvents, typically exhibit rapid conductivity degradation at low temperatures.^39,145^ The sluggish ionic motion at low temperatures also limits interfacial ion transfer due to the high energy barrier for ion desolvation, creating a bottleneck in the overall ion delivery process.^142,144,146^ In contrast, Li^+^ ions in SSEs migrate within immobile crystal structures including anion frameworks, eliminating the solvation structure present in liquid electrolytes.^28,147^ This prevents the drastic drop of ionic conductivity caused by solvent freezing and avoids the energy consumption for ion desolvation during charge transfer at solid–liquid interface boundaries.^39,148−150^ However, since ionic conductivities of SSEs typically follow the Arrhenius equation, the activation energy still determines temperature-dependent conductivity.^28,147^ Additionally, Li^+^ ions in SSBs may cross multiple phase boundaries, including the anode and cathode interphases, and the impact of these boundaries on the charge transport process is still debated.^147^ In contrast to liquid electrolyte, the immobility of anions in SSEs allows for a transference number close to unity for inorganic SSEs (see Section 3.3). This can prevent undesired concentration polarization arising from Li^+^ depletion at the electrode interface, potentially enabling fast cycling rates of SSBs.

Interfacial stabilities of SSE materials with cathode active materials have also been explored (Figure 2a). The cathode–SSE interface can experience electro-chemo-mechanical degradation compared to the anode–SSE interface due to the higher interfacial area in cathode composites.^151,152^ Here, we provide a brief overview of the electrochemical stability of certain SSE materials, while comprehensive chemo-mechanical effects within composite cathodes are discussed in Section 7. Among the various SSEs, the sulfide SSEs have been extensively studied and have relatively narrow stability windows, leading to instabilities at high potentials (Figure 2a). Koerver et al. investigated the reaction of β-Li_3_PS_4_ with NMC811, revealing instabilities above 3.8 V vs Li/Li^+^, where oxidized sulfur and phosphorus species were formed.^153^ Sulfide SSEs can undergo oxidative decomposition at high potentials even in the absence of cathode active material, leading to increased interfacial resistance.^154^ These detrimental effects have spurred research into developing protective coating strategies, along with their methods and effectiveness, are discussed in other reviews (see Section 7.1.3).^134,155,156^

As discussed in Section 3.1.1, it is challenging for a single SSE material to meet all the requirements for stable SSB operation, such as cathode/anode interfacial stability and high ionic conductivity. Hence, bilayer SSE structures using halide SSEs at the positive electrode have been investigated to promote stability with cathode active materials. For example, LIC has often been used as a catholyte interfacing with cathode materials due to its relative stability in the high voltage range (Figure 2a) and high ionic conductivity (Section 3.1.1).^9,157−159^ Meanwhile, LPSCl has been deployed as an anolyte due to its kinetic stability in contact with many anode materials.^158,159^ However, the additional interface created by the bilayer separator structure introduces different chemo-mechanical characteristics compared to a single-layer SSE, requiring further investigation.^160−162^

#### 3.2.2 Factors Influencing Contact Evolution at Solid–Solid Interfaces

Volume changes of electrode materials result from the electrochemical insertion and removal of active ions and associated redox processes. Examples of anode reaction processes accompanied by volume changes include the Li^0^/Li^+^ redox reaction for Li metal, alloying of Li with materials such as Si, and intercalation of Li in graphite. On the cathode side, both conversion and intercalation reactions generally involve volumetric expansion and contraction. Additionally, interphase formation/growth may occur at both electrodes and involves morphological and volume changes.

The thickness of a Li metal negative electrode changes by 15 to 25 μm each cycle during deposition and stripping when using practical areal capacities. The deposition/stripping of this quantity of Li each cycle can be physically accommodated at the confined Li–SSE interface due to plastic deformation and creep of Li metal under relatively low applied mechanical stress (the Li yield strength in the bulk is ∼0.8 MPa).^163−166^ However, nonuniform deposition and stripping or other morphological instabilities at the interface can induce localized stresses, causing cracks or dendritic Li penetration into the SSE (Figure 3).^167−169^ Li metal has been reported to have higher mechanical strength at the nanoscale than in the bulk, which implies that Li may exhibit greater resistance to plastic deformation at length scales relevant to interfacial flaws.^164,170^ Finally, when Li metal is oxidized and ions are transferred into the SSE, vacancies are left behind. If these vacancies are not removed through self-diffusion or plastic deformation of Li, they can coalesce into voids, which causes contact loss at the interface. Further discussion of Li metal evolution in SSBs is included in Section 5.

**Figure 4:**
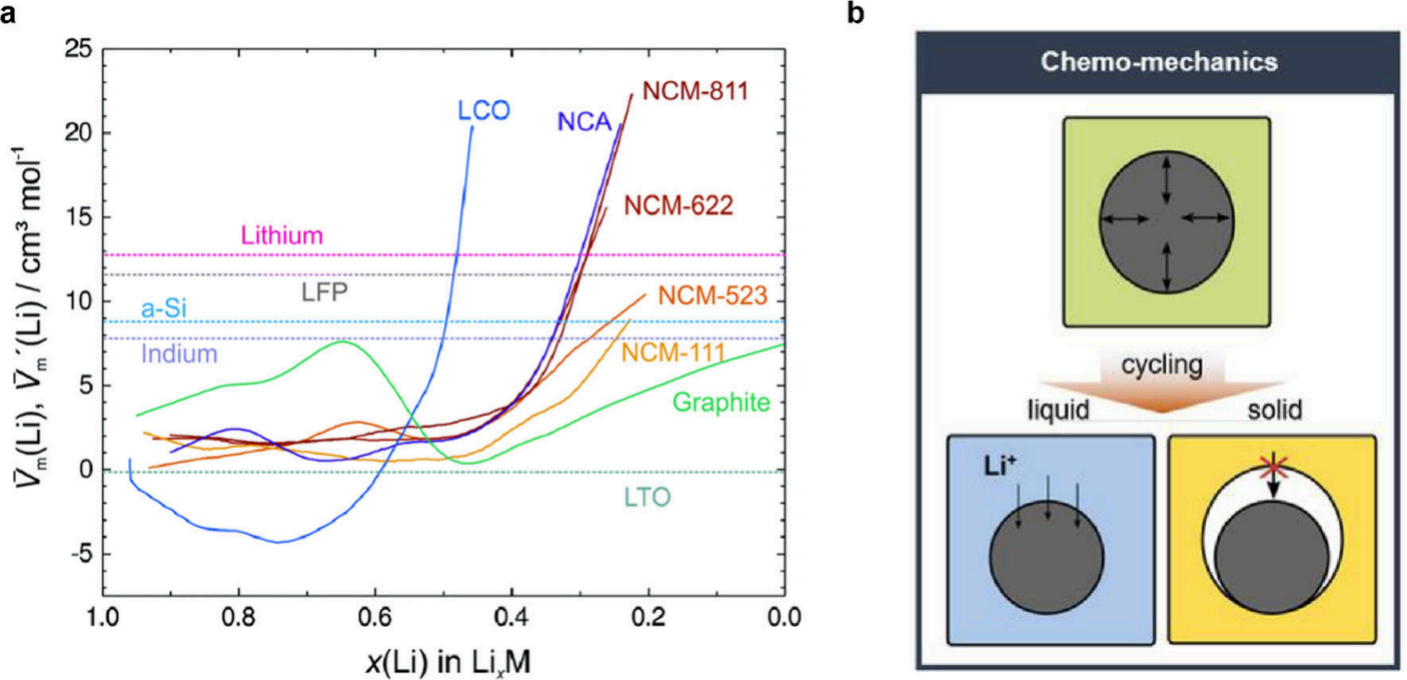
(a) Partial molar volumes of Li in homogeneous single-phase (*V̅*_*m*_(Li)) and two-phase (*V̅*_*m*_^′^(Li)) electrodes as a function of lithiation state. Reproduced with permission from ref (171). Copyright 2018 Royal Society of Chemistry. (b) Schematic image of interfacial contact between electrochemical active materials and liquid electrolyte (blue)/SSE (yellow). Unlike liquid electrolytes, which can flow to compensate for volume change of active materials, volume changes of active materials in composite particulate electrodes cause interfacial contact loss and reduced Li delivery pathways. Reproduced with permission from ref (134). Copyright 2022 Wiley-VCH GmbH.

Composite particulate electrodes in SSBs typically feature a mixture of active material particles and SSE, and they undergo volume expansion and contraction depending on their state of charge. Graphite experiences a volumetric strain of up to 13% at full lithiation, while alloy anodes can experience volume changes as high as 300%.^5,171^ The conversion reaction of S cathodes causes a volume change of up to 79%.^172^Figure 4a shows the partial molar volume of Li in various electrode materials (shown as *V̅*_*m*_(Li) and *V̅*_*m*_^′^(Li) for homogeneous single-phase materials and two-phase materials, respectively), which represents the differential volume of an electrode material as Li is added. The volume change of electrode materials upon lithiation can be calculated by integrating *V̅*_*m*_(Li) or *V̅*_*m*_^′^(Li).^171^ The partial molar volume is nonlinear as a function of state of charge in some intercalation or insertion cathodes, indicating a complex trend of volume changes during the Li insertion/removal process. The volume changes during cycling may cause morphological evolution of composite electrodes, which can result in the loss of ionically conductive pathways and a decrease in ionic conductivity (Figure 4b).^77,134,139,172,173^ Additionally, composite electrodes may experience electrochemical fatigue due to strain accumulation via repeated ion insertion and extraction, potentially leading to permanent capacity loss.^134,174^ Finally, chemo-mechanical balancing of electrode materials, where volume changes at both electrodes compensate for each other, may reduce the net stress in a cell and could be useful for managing global chemo-mechanical effects within SSBs.^171^

External stress, applied via uniaxial stack pressure, is widely used to control and alleviate stress-driven damage and contact loss at interfaces by inducing plastic deformation of the electrode materials and enhancing contact at interfaces. An example is the visco-plastic deformation of Li metal to regulate interfacial morphology. Undoubtedly, higher stack pressure is a direct route to activate deformation mechanisms and prevent interfacial disconnection at the electrode/SSE interface. However, recent reports indicate that even a moderate level of stack pressure can induce the propagation of Li filaments through the SSE to cause short circuiting.^167,175^ This indicates a conflicting effect of stack pressure in controlling the interfacial morphology during Li stripping and plating: Stack pressure helps maintain Li contact during stripping but can cause filament growth during plating. A recent study demonstrated that stack pressure application in cells with variable volume, such as when using springs on the cell housing, can reduce degradation in the cell.^176−178^ From a practical perspective, realizing stack pressures greater than a few megapascals for commercial SSBs is impractical, as it requires bulky cell housings.^13,179−181^ Hence, novel strategies to control chemo-mechanical effects and maintain solid–solid interfacial contact at relatively low stack pressures are necessary to realize SSB commercialization.

### 3.3 Liquid Electrolyte Properties and SEI Formation

In this section, we highlight the basics of liquid electrolytes and their interfaces within Li-ion batteries, with the purpose of comparing to SSEs in the previous section. The liquid electrolytes in Li-ion batteries are made up of various salts dissolved in organic solvents. Ideal properties for electrolytes include high ionic conductivity in the range of 1 to 10 mS cm^–1^,^182^ low cost, thermal and electrochemical stability, and low freezing points, although many of these properties tend to be in conflict. Because electrolytes often have salts, additives, and multiple solvents, the ion solvation structure, chemical properties, and decomposition mechanisms of electrolytes are complex. Understanding these factors is paramount to enhancing performance of future batteries.

#### 3.3.1 Electrolyte Constituents

##### 3.3.1.1 Solvents

Various types of solvents are used in Li-based batteries, with different stability windows, viscosities, and thermal and environmental stability. A selection of these solvents is described here.

##### 3.3.1.1.1 Ethers

Ether solvents contain an O atom bonded to two organyl groups, with the general form ROR’. Many ethers, like dipropyl ether, dimethyl ether, and diethyl ether, are used as cosolvents in electrolyte research. These solvents tend to show good stability against Li metal anodes, resulting in denser Li morphology during Li deposition compared to other solvent classes.^183,184^ However, ethers show poor oxidative stability and decompose around 3.5 V vs Li/Li^+^.^185^ They tend to be more volatile^82^ and can cointercalate along with Li^+^ ions in graphite,^186^ resulting in destruction of the graphite structure. These issues have hindered the widespread adoption of ethers in battery electrolytes, although some challenges can be overcome by using high concentration electrolytes.^187,188^

##### 3.3.1.1.2 Esters

Esters such as ethyl, propyl, and butyl acetates are of the general form RCOOR′, where R and R′ are organyl groups. They are commonly used in electrolyte formulations to promote fast charging of Li-based batteries.^189,190^ They have good oxidative stability (to around 4.0 V vs Li/Li^+^),^191,192^ high polarity, and low viscosity,^189^ resulting in common use as cosolvents. Their low melting points result in their use in low-temperature electrolytes.^186,193^ However, esters exhibit low flash points,^189^ which increases safety concerns and manufacturing difficulty, and many salts have low solubility in these solvents. Additionally, they have worse cathodic stability compared to ethers, leading to poor stability with Li metal.^194,195^

##### 3.3.1.1.3 Carbonates

Carbonates are the most common solvent in Li-ion battery electrolytes. This is due in large part to the success of ethylene carbonate in forming a stable SEI on graphite anodes,^196,197^ which was a key factor in the commercialization of rechargeable Li-based batteries. Carbonates are a subclass of esters with the general form ROCOOR′, where R and R′ are organyl groups. They include cyclic molecules like ethylene carbonate, propylene carbonate, and fluoroethylene carbonate, as well as acyclic molecules like dimethyl carbonate and diethyl carbonate. These solvents have improved anodic stability compared to ethers and other esters, enabling cathodes with higher electrode potential.^198,199^ They also tend to exhibit high ionic conductivity. The downsides of these solvents are that they are highly flammable^200^ and viscous.^201^ Ethylene carbonate, which is used in most commercial electrolytes, is electrochemically stable from around 1.0 to 4.5 V vs Li/Li^+^,^202,203^ but it has a melting point of 36.4 °C,^204^ hindering low temperature cycling. Bulk and interfacial properties of carbonate electrolytes are highly dependent on whether the solvent is cyclic or acyclic,^205^ and prior work has shown that cyclic carbonates are more likely to be present in the inner solvation sheath of Li^+^ ions,^201,206^ resulting in domination of the decomposition products in the SEI.^207^

##### 3.3.1.1.4 Other Solvents

As research on anode materials other than graphite expands, new classes of solvents are being included in liquid electrolyte formulations to reduce costs, expand operating temperature windows, and reduce safety and environmental concerns. Sulfones have high oxidative stability and low flammability but cannot form a passivating SEI on graphite and are highly viscous.^208,209^ Amides, like dimethylformamide, are also inexpensive and widely available,^189^ and the low lowest unoccupied molecular orbital of amides resulting in preferential decomposition over other solvents and improved transport properties within the SEI. Nitriles, like propionitrile, have improved safety, low viscosity, and low melting point, making them ideal for low temperature applications.^189,210^

##### 3.3.1.2 Salts

Li salts are necessary in liquid electrolyte formulations to enable field-driven transport of Li ions;^82^ otherwise, only Fickian diffusion would occur between electrodes and ionic conductivity would be negligible. While there are many organic solvents that are appropriate for Li battery electrolytes, there are far fewer salts that meet the requirements to form an electrolyte.^211^ Salts must be easily dissolved in organic solvents, preventing the use of simple salts^82,212^ like LiF or LiCl, which will not dissociate. The anion must also be relatively stable toward the solvent, the inert cell components, and to extraneous side reactions.

##### 3.3.1.2.1 Lithium Hexafluorophosphate (LiPF_6_)

LiPF_6_ is the most commonly used salt in commercial electrolyte formulations for Li-ion batteries^213^ due to its relatively high ionic conductivity^214^ and ability to resist oxidation up to 5.1 V vs Li/Li^+^,^196^ enabling use of high voltage cathodes. Although LiPF_6_ has lower conductivity, dissociation constant, and ionic mobility than various other salts,^215^ its combination of properties make it optimal for meeting the multifaceted requirements of Li-ion batteries. LiPF_6_ does exhibit low thermal stability^216^ and can generate toxic gases even at moderate temperatures, however.

##### 3.3.1.2.2 Lithium Tetrafluoroborate (LiBF_4_)

LIBF_4_ has garnered interest because of its improved safety compared to other salts.^217^ While it has one of the highest mobilities of known Li salts,^215^ its dissociation is limited,^218^ resulting in a low ionic conductivity,^218^ and it forms a less stable SEI than LiPF_6_,^219,220^ both of which have prevented large scale commercial adoption.

##### 3.3.1.2.3 Lithium Bis(oxalate) Borate (LiBOB)

LiBOB is of interest because it demonstrates high thermal stability,^221^ potentially enabling high temperature Li-ion batteries and can form sufficient passivation layers on graphite.^222,223^ However, the salt exhibits low solubility in carbonates,^221,224^ limiting its use in many electrolyte formulations.

##### 3.3.1.2.4 Lithium Bis(trifluoromethanesulfonyl)imide (LiTFSI)

LiTFSI has been studied in recent years due to it high donor number,^225^ good conductivity,^212^ high thermal stability,^226^ and its ability to form stable passivation layers on Li metal. Corrosion of the Al current collector can occur above 4.0 V vs Li/Li^+^, although this can be minimized with other additives.^227^

##### 3.3.1.2.5 Lithium Nitrate (LiNO_3_)

LiNO_3_ is used extensively in next-generation battery chemistries, like Li–S^228^ and Li -air batteries,^229^ because of its ability to stabilize Li deposition and minimize dendritic growth. It generally exhibits poor solubility in organic solvents but can improve battery performance with low concentrations,^228^ and dissociation can be promoted through additives to enable high voltage operation.^230^

#### 3.3.2 SEI Formation at Negative Electrodes

The electrode potential of most negative electrodes exists outside of the stability window of most organic solvents used in Li-ion battery electrolytes, resulting in the reductive decomposition of electrolyte constituents. Li metal, in particular, will reduce all known organic solvents.^231^ If the decomposition products are solid, they form a layer on the surface of the anode. When this layer is electronically insulating and ionically conducting,^197,232^ decomposition of the electrolyte ceases after the layer has grown to a certain thickness, and a passivating interphase is produced. Desired properties for the SEI include minimal thickness, good mechanical strength and flexibility,^197,232^ and low solubility in the electrolyte.^233^ SEI layers in liquid electrolytes have been shown to have a bilayer structure,^234−236^ with an inner layer made of mostly inorganic decomposition products (e.g., Li_2_O, LiF, and Li_2_CO_3_) that provide good ionic conductivity, and an outer layer made of mostly organic decomposition products (e.g., ROCO_2_Li and ROLi) which improve flexibility. Mechanical and chemical stability of the SEI is vital to the function of Li-ion batteries and the SEI has thus been widely studied. This section contains information on SEI formation on various anode materials in Li-ion batteries, for comparison to the field’s current understanding of interphase formation in SSBs in the previous section.

##### 3.3.2.1 Li Metal

Because Li metal has an extremely low electrode potential, decomposition of electrolyte constituents is rapid, and all electrolyte components are likely to contribute to the decomposition products making up the SEI.^231,237^ The high reactivity of Li makes the electrochemical stability and longevity of the SEI of utmost importance, and much work on Li metal batteries has focused on engineering the SEI with desirable properties. Early work attributed the difference of cycling stability of cells with ethylene carbonate and propylene carbonate to changes in the SEI.^238,239^ Although the two solvents are quite similar in structure and decompose to form semicarbonates,^240^ ethylene carbonate is more reactive with Li metal,^238,239^ potentially due to the electron donating properties of the methyl group in propylene carbonate.^240^ Li deposition in ethylene carbonate-based electrolytes tends to be dendritic.^238,240^ However, Li metal was abandoned early in Li-ion battery development because of short lifetimes and safety concerns.^240−242^ The recent renewed interest in Li metal electrodes has led to the expansion of electrolyte compositions and advances in characterization techniques allowing for better understanding of the SEI structure and composition.^243−246^

##### 3.3.2.2 Graphite

Due to its ability to stabilize the Li metal interface, propylene carbonate-based electrolytes were extensively investigated with graphite anodes in the early days of Li-ion batteries.^247,248^ However, poor electrochemical performance was observed.^249,250^ It was found that propylene carbonate intercalates into the graphite, leading to delamination of the graphene layers.^249,251^ Ethylene carbonate, despite showing poor stability in Li metal batteries, does not induce the delamination of graphite electrodes and indeed leads to reversible lithiation/delithiation behavior in graphite.^252^ The decomposition of this solvent creates a stable SEI layer,^253,254^ which forms fully over the first few cycles and may suppress solvent intercalation.^197,255,256^ Depending on solvent and salt combinations, the SEI formed on graphite can be 5–200 nm thick.^257,258^ Most commercial electrolyte formulations include ethylene carbonate for these reasons.

The electrode potential of lithiated graphite is only slightly above that of Li metal (0.1 V vs Li/Li^+^),^231,251^ and stepwise decomposition of the electrolyte constituents occurs during initial charge to form the SEI.^231^ For example, when ethylene carbonate is included with propylene carbonate, a SEI arising from the ethylene carbonate decomposition products can form to prevent intercalation of the propylene carbonate, improving performance.^197,259^ Additionally, preferential solvation can result in favored decomposition of a particular electrolyte constituent, providing a mechanism to control the chemistry of the SEI.^206^

##### 3.3.2.3 Alloy Anodes

SEI formation on alloy anodes (such as Si, Sn, and Al) is of particular importance because of the large volume changes that occur during lithiation and delithiation. Capacity loss and battery failure commonly occur because of severe solid electrolyte interphase formation,^260,261^ either entirely consuming the active Li inventory and electrolyte or electronically isolating the active particles and preventing reaction.^262,263^ While intercalation electrode materials expand and contract 5–10% during reaction, alloy anodes expand and contract up to 300%. With large volume changes, the SEI can fracture and the liquid electrolyte will react with the newly exposed active surface area, creating more SEI.^264−266^ It is thus important to use electrolyte compositions whose decomposition products create an SEI with stable chemistry and mechanical properties that avoid fracture with alloy anodes.^262,265,267,268^

Prior work has shown that SEI formation on alloys can differ greatly from graphite,^269^ despite the fact that many alloys react at similar potentials to graphite and both are less reactive than Li metal. Stability of the SEI can vary based on the alloy used, as some alloys can catalyze the decomposition of initial SEI constituents, while others do not, and active material can be incorporated into the SEI.^270,271^ Like graphite, alloys experience preferential decomposition of particular electrolyte solvents,^262,268,272^ resulting in an SEI made mostly of those decomposition products, even if the species is only an additive making up a small portion of the solvent mixture.^268,272^ A smaller body of work focuses on understanding SEI formation on alloys compared to graphite and Li metal, and more research is necessary to fully understand SEI formation on various alloy anodes.

#### 3.3.3 Liquid Electrolyte Properties and Relationship to SEI

##### 3.3.3.1 Rate Constant of Reduction, *k*_e_

Rapid formation of the SEI has been shown to be important for creating an effective protective layer. The rate constant of reduction, *k*_e_, is a good measure of the reactivity of various electrolyte constituents and is a well-documented value for many molecules. High *k*_e_ values of salts and solvents correlate to the inclusion of decomposition products in the SEI, providing a mechanism to control the chemistry of the SEI.^233^ High reactivity also corresponds to high decomposition potentials, as lower overpotentials are required for reactions to occur.^186,233,273^

##### 3.3.3.2 Solvation

The molecular solvation structure around Li ions in the electrolyte solution plays a large part in determining the electrolyte conductivity and desolvation energy, and it can dictate the final chemical composition of the solid electrolyte interphase.^143,144^ Solvation occurs because of electrostatic interactions between the electrolyte components and the dissociated Li ion. While the bulk electrolyte is in contact with the interface, solvents that exist in the inner solvation sheath are preferentially reduced to form the SEI.^205,274^ Therefore, understanding how solvent and salt molecules interact with Li^+^ ions can allow for control of the SEI properties, although solvation structure can be difficult to predict due to the complex nature of electrolyte mixtures. In general, solvents are more likely than salts to have strong interactions with Li ions, resulting in more organic constituents in the SEI; however, high-concentration electrolytes can alter the solvation structure to force interaction between Li^+^ ions and salts to generate an anion-derived SEI.^275−278^ The chemical structure of solvent molecules has also been shown to dictate solvation, with a strong preference for solvation of cyclic solvents over linear.^206,279^ Steric hindrance of molecules prevents solvation, which in turn alters the chemistry of the SEI.^207,278^

##### 3.3.3.3 Transference Number

The transference number of Li^+^ ions is the fraction of ionic conduction in the electrolyte that is carried by Li ions. Increased Li^+^-ion transference numbers provide a mechanism to increase the power density of Li-ion batteries.^280^ Most liquid electrolytes have a transference number of Li ions less than 0.5, indicating that the majority of the ionic conductivity is a result of the motion of the anion instead of Li^+^ ions.^182,281,282^ Reduced transport of the Li^+^ ions occurs because the bulky solvation structure hinders mobility, while anions are relatively free to move about.^280,283^ High transference of the anion leads to concentration gradients, increased overpotential, and reduced current.^280^ We note that this is a key difference between liquid- and solid-state electrolytes, where inorganic SSEs typically exhibit Li^+^ transference numbers of 1.0 since there are no mobile anions. Multiple works have found correlations between increasing the transference number and the stability of Li metal deposits and the SEI.^284,285^ However, as the transference number is strongly tied to solvation,^280^ changes in the stability of the SEI could be due to alterations of the solvation structure instead. For example, work has demonstrated the inverse relationship between transference number and stability in potassium systems.^286^

## 4 Characterization of Solid-State Batteries

**Figure 5:**
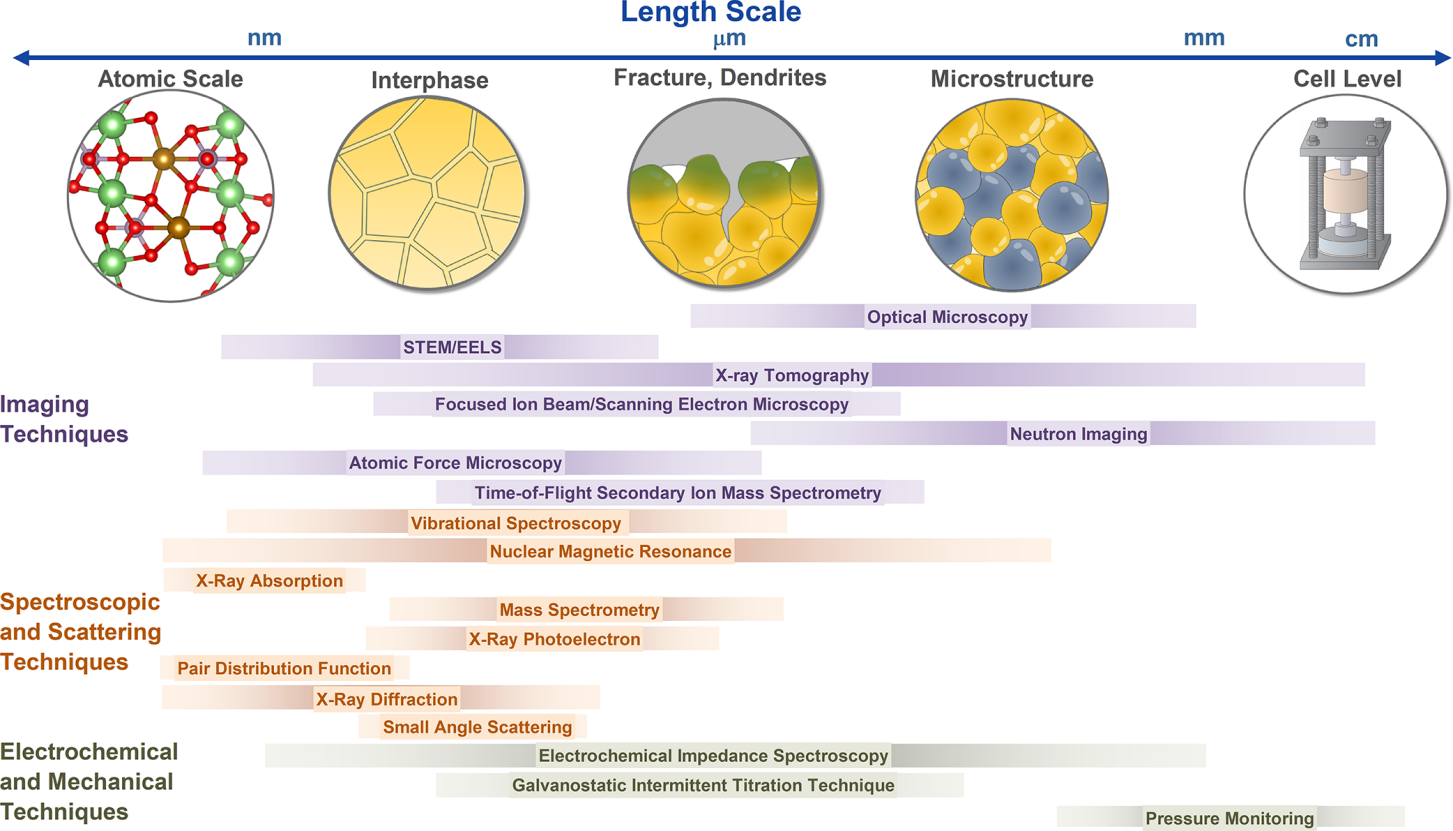
Summary of various imaging, spectroscopic, electrochemical, and mechanical characterization techniques and their length scales used to characterize SSB electrodes and electrode–electrolyte interfaces at the atomic, microscale, and cell levels.

To realize commercially viable SSBs, understanding how the materials and interfaces within SSB cells evolve and degrade during electrochemical cycling is paramount. Electrochemical characterization itself is foundational for understanding SSB materials. Beyond electrochemical characterization, a variety of other *ex situ*, *in situ*, and *operando* characterization techniques has been developed to investigate the structure, chemistry, morphology, and other properties of SSEs and electrode materials in SSBs. Various governing phenomena in SSBs have been experimentally investigated, including Li deposition and stripping behavior,^133,287−290^ SSE and electrode degradation mechanisms,^291−294^ and mechanical stress evolution during cycling.^175,295,296^ Still, there remains much to understand about the operational mechanisms of materials within SSBs, particularly regarding the evolution of electrodes other than Li metal during cycling, as well as the behavior of SSB systems with commercially relevant SSE thicknesses (<50 μm) and stack pressures (<1 MPa). In this section, we provide a comprehensive overview of the characterization methods that have been used for SSB research (Figure 5), including their characteristics, distinctive needs for SSB research, and important findings enabled by these various techniques.

### 4.1 Electrochemical Characterization

#### 4.1.1 Design of Electrochemical Cells

The design of electrochemical cells for solid-state electrochemical measurements is important for obtaining accurate and reliable understanding of SSB electrochemistry. Designs that can accommodate ceramic pellets or other SSE form factors are needed, and the control of stack pressure is often necessary since stack pressure can affect behavior and contact at interfaces. Various factors need to be considered for these cell designs, including housing material, stack pressure application procedure, alignment of cell components, and reproducibility. Several cell formats are commonly employed for electrochemical characterization of SSBs, each offering advantages and disadvantages.^293,297−299^

Cell formats and designs that allow for controlled application of pressure are important in SSB research. Typical liquid-electrolyte batteries are generally assembled after calendering electrodes but without applying an excess “formation pressure” to establish contact between the electrodes and separator. These batteries are then cycled with minimal to no external applied pressures. SSBs, on the other hand, often require the use of controlled formation pressures throughout cell assembly to ensure interfacial contact.^1,300,301^ SSBs are also often cycled under an externally applied stack pressure.^27,302−304^

**Figure 6:**
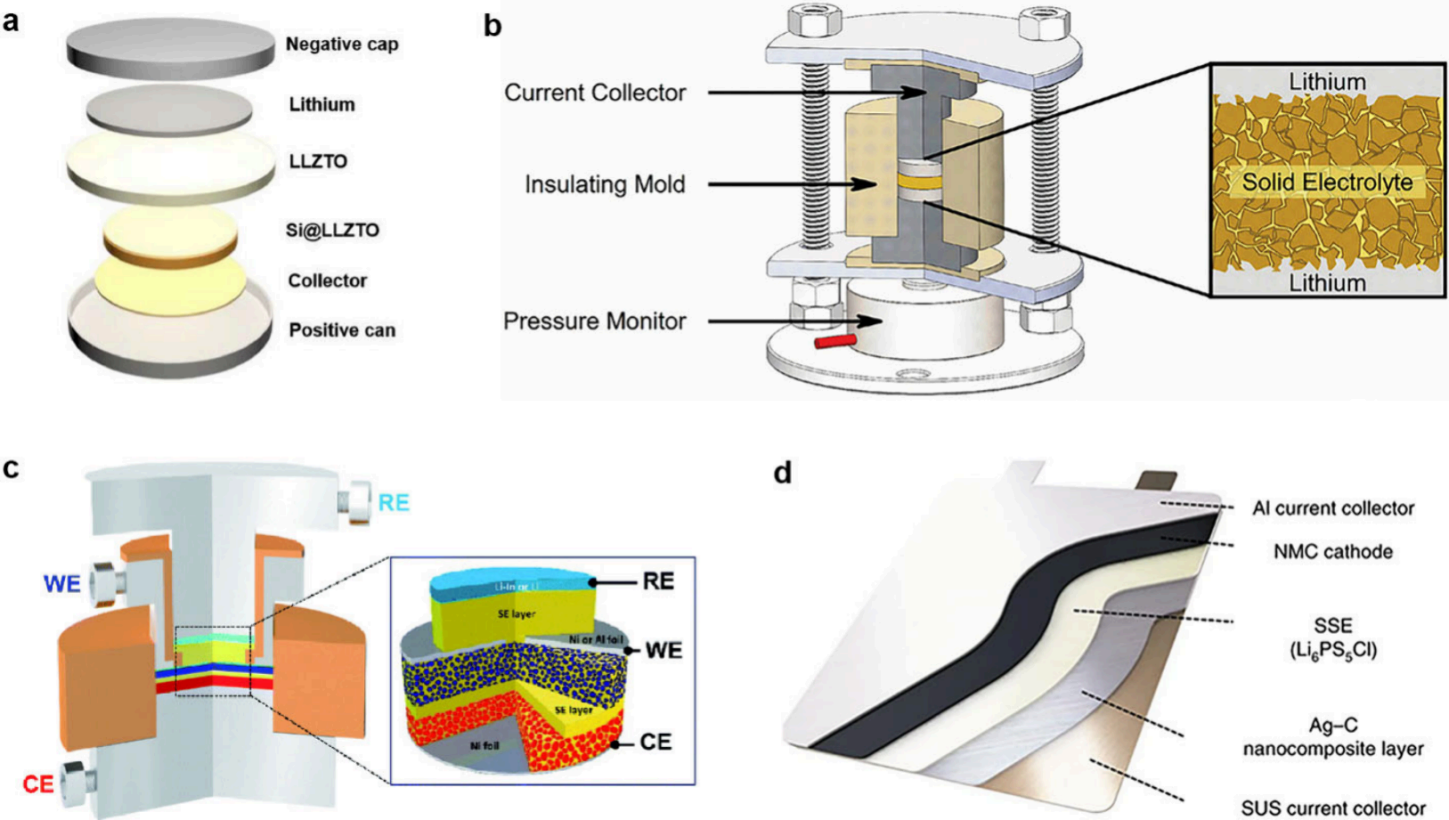
Schematics of various electrochemical cell designs for SSBs. (a) Schematic of a coin cell used with an LLZO-based battery. Reproduced with permission from ref (307). Copyright 2024 Elsevier. (b) Anvil cell schematic with pressure sensor. Reproduced with permission from ref (311). Copyright 2020 WILEY-VCH Verlag GmbH & Co. Kga. (c) Three-electrode anvil cell; WE = working electrode, RE = reference electrode, CE = counter electrode. Reproduced with permission from ref (312). Copyright 2018 Royal Society of Chemistry. (d) Schematic of a pouch cell. Reproduced with permission from ref (313). Copyright 2020 Springer Nature.

Coin cells are commonly used for electrochemical characterization of liquid-electrolyte batteries due to their simplicity and compatibility with standard testing equipment. Coin cells are easy to fabricate and provide an airtight seal, allowing for testing in atmosphere. Coin cells have been used for some SSB testing. In Figure 6a, the SSE is a sintered ceramic pellet with electrodes (such as Li metal) pressed on either side.^293,305−307^ However, the limitations of coin cells have precluded widespread use for SSBs. In particular, they do not allow for controlled or uniform stack pressure application, since the pressure within a coin cell is governed by the compression of the internal spring. Cell stack thickness and spring size will therefore affect the internal stack pressure.^308,309^ The internal pressure can change when the stack thickness is varied, with pressures in one study having been found to vary between 40 and 103 kPA.^310^

“Anvil cells” are a common cell format used for SSB testing. These cells are similar to Swagelok-type cell designs used in liquid-electrolyte battery research, but they are customized for SSB investigations. Anvil cells consist of an electrically insulating die (such as polyether ether ketone (PEEK)) with two inert metal rods (such as steel or Ti) inserted into the die (Figure 6b). The cell stack is inserted into the die, and formation pressures can be applied with the rods to densify material as needed. The rods are then tightened between plates to apply a stack pressure while simultaneously electrically contacting the electrodes.

Anvil cells offer more precise control over formation and stack pressures than coin cells.^9,314−316^ Stack pressures in the low MPa range are often necessary in SSBs to reduce void formation at Li anodes and to maintain contact at solid–solid interfaces.^27,317^ Springs can be used between the stack plates and nuts to minimize stack pressure variation due to electrode volume changes during cycling, and force sensors can be directly integrated into the cell to measure stack pressure.^176,296,311^ Anvil cells are often custom-built by SSB researchers and thus vary from study to study. Many designs do not provide an airtight seal, requiring that battery testing occur in an inert environment such as a glovebox.

Three-electrode anvil cell designs have been implemented in some SSB studies to enable measurement of potentials with respect to a reference electrode.^169,301,312,318−320^Figure 6c shows a schematic of a three-electrode anvil cell. In this configuration, a standard SSB is assembled within the anvil cell with a cell stack consisting of the working electrode/SSE/counter electrode. The third electrode is then introduced by adding a reference electrode/SSE bilayer in contact with the working electrode. This cell design is different than three-electrode liquid cells, where the reference is usually found between the working and counter electrode. Quemin et al. used a three-electrode cell to track the cathode composite electronic conductivity by integrating an Al mesh between the SSE and the cathode composite.^319^ Other work has used airtight three electrode cells to track the anode impedance during cycling.^320^

The pouch cell format has also emerged as an important cell type for SSB research. The electrode area of pouch cells is more flexible and can be larger than in anvil cells.^321^ However, pouch cells often necessitate the use of a thin SSE membrane upon which electrodes can be laminated (Figure 6d).^51,159,322,323^ Free-standing sulfide SSE membranes and electrode films with high ionic conductivities that can be used for pouch cells have been fabricated with dry and wet processing.^324,325^ Traditional slurry casting processes have limitations such as solvent toxicity and reactivity between the SSE and solvents, sometimes leading to inferior ionic conductivity.^325,326^ Solvent-free dry processing using fibrous polytetrafluoroethylene (PTFE) binders has been used to make thin-film SSE membranes and electrodes,^326−328^ providing benefits of low cost and high conductivity. However, the PTFE binder can be converted to carbon due to reductive defluorination, which can result in interfacial instability during cycling.^297,329−331^ Using binder-free active materials at the anode like thin sputtered film electrodes, foil-type alloy anodes, or “anode-free” Li cells can simplify the manufacturing process for pouch cell formats.^51,313,332^ Pouch cells are often tested with stack plates that can apply controlled stack pressures, similarly to anvil cells.

#### 4.1.2 Electrochemical Characterization

Common electrochemical testing methods such as galvanostatic cycling, cyclic voltammetry, and constant-current constant-voltage charging are used for SSB testing. Additionally, other more complex electrochemical methods are used and have distinctive characteristics when applied to SSBs. Some are described here.

##### 4.1.2.1 Electrochemical Impedance Spectroscopy

Electrochemical impedance spectroscopy (EIS) is a powerful, nondestructive, highly effective electrochemical technique used to analyze transport properties and the complex impedance of different components of a battery cell. It is particularly well suited to investigate changes of local impedance at interfaces and other regions during cell operation, assuming the data can be fit to an accurate physical model of the cell. EIS measurements consist of an applied sinusoidal input voltage or current signal that is swept across a wide range of frequencies. The resulting output current or voltage signal is measured. The impedance, *Z*(*t*), is determined by the ratio of these two signals. Experiments in which the input signal is a voltage, as described here, are referred to as potentiostatic EIS. When the input signal is an alternating current, the experiment is referred to as galvanostatic EIS.

**Figure 7:**
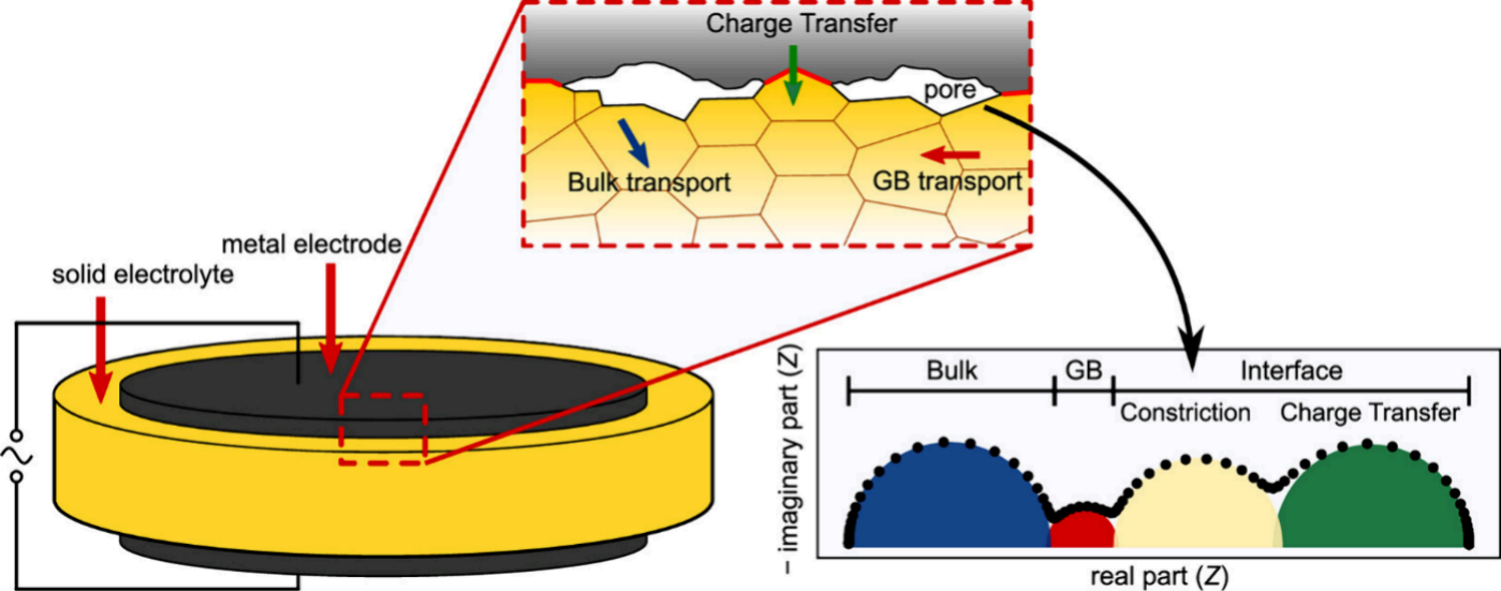
Schematic highlighting the different types of transport processes that give rise to EIS features in SSBs. Voids at the solid–solid interface (“constriction”) can lead to separate or overlapping contributions in the spectra. Reproduced with permission from ref (333). Copyright 2022 American Chemical Society.

Nyquist plots are created by plotting the real and imaginary impedance at each frequency. In liquid electrolyte systems, 1D equivalent circuit models are often fitted to EIS data to model the cell internals as electrical components in a circuit that represents the overall electrochemical behavior of the battery. In SSB characterization, different transport processes contributing to the overall electrical response of the cell can be isolated if their response frequencies differ (e.g., bulk, grain boundary, and interface transport; Figure 7).^333^ However, recent studies have shown that contact loss at interfaces and the resulting current constriction in SSBs cannot be effectively represented with typical 1D equivalent circuit models, since current constriction is inherently a 3D effect near the electrode/SSE interface. As a result, analyzing and interpreting EIS of SSBs can be challenging and requires more complex approaches.^334^

Numerous studies have used EIS to monitor subtle changes in the electrochemical response of cells during cycling and have related this to physical mechanisms.^154,320,335^ Krauskopf et al. explored the electro-chemo-mechanical behavior of Li metal at a variety of pressures and temperatures in an LLZO symmetric cell with GEIS. Constriction effects were evident at low pressures, thus resulting in high interfacial resistances.^303^ Koerver et al. used EIS to reveal a resistance increase in the first cycle due to the formation of a passivating cathode–SSE interphase layer, which was ultimately responsible for the low-capacity retention in the first cycle.^153^ Other work has used PEIS to investigate the effects of alloy interlayers on the nucleation, growth, and stripping of Li in anode-free SSBs. Clear signatures of interfacial contact loss were observed in the EIS spectra.^315^

##### 4.1.2.2 Galvanostatic Intermittent Titration Technique (GITT)

GITT is an electrochemical technique that can be used to characterize diffusion kinetics and transport properties of battery electrode materials. In GITT experiments, a small constant current is applied to the electrode for a short time interval, followed by removal of the current and an open-circuit hold, during which the voltage (or potential if a three-electrode cell is used) of the system relaxes toward equilibrium. This process is repeated throughout charge/discharge cycling. The data reveals the overpotential as a function of state of charge, which provides qualitative information about reaction/diffusion kinetics. If performed under controlled conditions in which a variety of assumptions are met, GITT experiments can be used to extract chemical diffusion coefficients in single-phase materials.^336,337^ However, GITT experiments are often not carried out with all assumptions satisfied, resulting in inaccurate diffusion coefficient extraction. Regardless, GITT measurements can provide valuable information about the kinetics and thermodynamics of electrodes and cells.

### 4.2 Battery Characterization Strategies

#### 4.2.1 *Ex Situ, In Situ*, and *Operando* Characterization

The operation of batteries is largely governed by dynamic processes within materials and at interfaces. When designing experiments to observe and understand the behavior of battery materials with methods beyond electrochemical techniques, one must decide whether a phenomenon of interest can be observed *ex situ*, *in situ*, or *operando*. While these terms are frequently used to describe different types of characterization experiments in battery research, there is no universally accepted definition for each. We attempt to provide general definitions and descriptions of these characterization strategies here as applied to battery research.

The terminology *ex situ* refers to characterization that is performed separately from any reaction processes (e.g., imaging of an electrode before and after electrochemical cycling by removing the electrode from the cell). The terminology *in situ* denotes experiments that are performed on battery materials in a native cell environment, with or without dynamic reaction processes occurring. The terminology *operando* describes experiments on materials in a cell environment where the characterization and reaction process are performed simultaneously, and in which the experimental electrochemical conditions closely match realistic battery conditions (for instance, if electrochemical cycling is performed using large-area electrodes at practically relevant current densities). In this section, we will delve into the differences among *ex situ*, *in situ*, and *operando* characterization in the context of SSB research.

**Figure 8:**
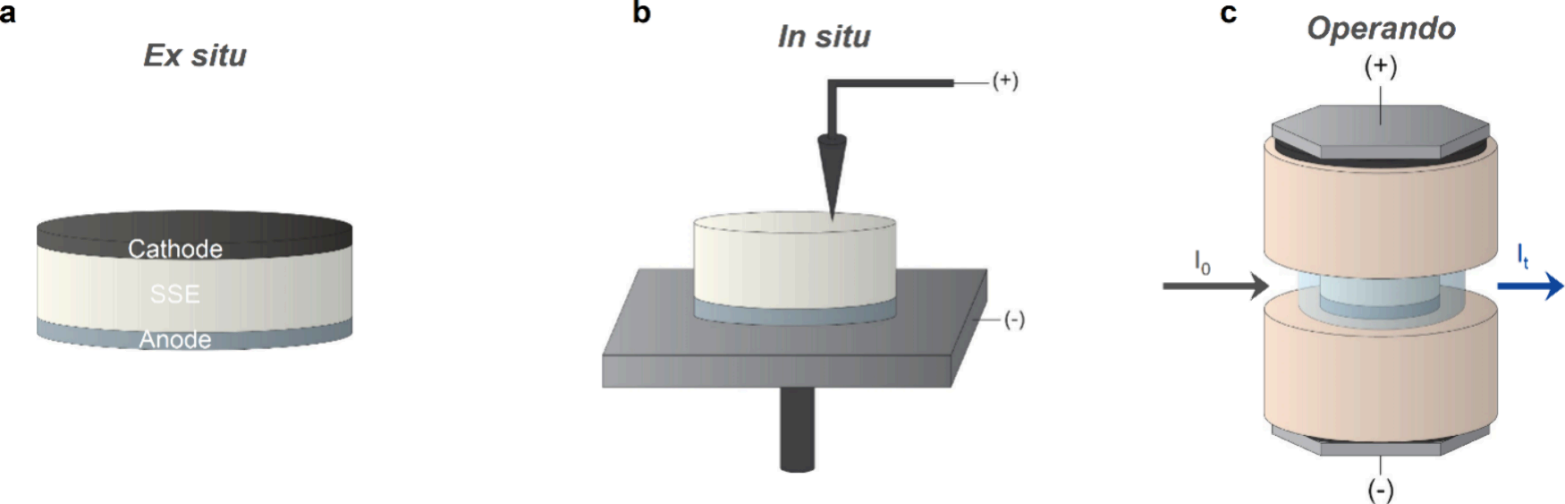
*Ex situ*, *in situ*, and *operando* SSB experimental design examples. (a) General schematic for *ex situ* SSB experiments showing a cell stack consisting of a cathode, SSE, and anode; the material is removed from the cell for characterization. (b) General schematic for *in situ* SSB experiments showing possible potential control within a modified cell environment, and the materials are characterized in place. (c) General schematic for *operando* SSB experiments showing characterization of materials within an operating SSB cell.

*Ex situ*: In SSB research, *ex situ* characterization is performed by removing battery components from the electrochemical cell or from the electrochemical conditions in which reaction processes were carried out. The reaction process usually takes place in a normal battery cell environment, and experimental designs for characterization are generally simple. Owing to these attributes, *ex situ* techniques are the most common characterization strategy in SSB research (and in battery research broadly). A typical example would be performing imaging or spectroscopy on an electrode before inserting into a cell, then assembling a battery cell and electrochemically testing, and finally removing the electrode from the cell and characterizing the electrode again (Figure 8a).

There are several issues that should be taken into consideration when characterizing battery materials with *ex situ* methods. First, *ex situ* techniques do not provide real-time information regarding phenomena of interest, leaving out the dynamics of reaction processes. This leaves room for misinterpretation of how material evolution in the SSB takes place. Next, multiple samples need to be fabricated when testing different variables, such as potentials, current densities, or stack pressures; there may be unknown deviations from cell to cell that cause difficulties in analyzing the data. Finally, extraction of the SSB materials from the housing that it was electrochemically cycled within may cause damage, contamination, extraneous side reactions, or microstructural changes before accurate characterization can take place. When using *ex situ* characterization, a challenge is not only that these changes can occur, but that they are also difficult to identify or track.

*In situ* and *operando* techniques allow for observation of materials within the environment of a battery cell (*in situ*) or during reaction processes caused by charge/discharge of a battery (*operando*). Such experiments can provide rich information about the behavior of materials in batteries, but they are generally more challenging than *ex situ* experiments and have some limitations. While there is some overlap in the use of these terms, the general differentiation between the terms within battery research is described below.

*In situ*: The Latin term *in situ* means “in place.” *In situ* measurements are carried out on materials within electrochemical cells in a static state or under dynamic electrochemical conditions.^338^ Generally, however, the experimental conditions, cell design, or other parameters are different than conventional electrochemical testing to simplify experimental procedures or to enable the characterization technique to be carried out. For instance, testing may occur in small-scale probe-type cells or under the influence of high/uncontrolled voltages (Figure 8b).

*Operando*: *Operando* characterization is carried out on batteries undergoing charge and/or discharge under realistic cell conditions. *Operando* experiments are thus performed *in situ*, but *in situ* experiments are not performed *operando*. The general distinction between the two terms is that *operando* characterization is done under conditions identical to or closely mirroring those used in real batteries (Figure 8c). As such, the electrochemical cell design, current density, areal loading, applied stack pressure, and other operational parameters relevant to the system being studied are realistic. Furthermore, a key aspect of most *operando* measurements is that reliable electrochemical signatures can be measured and directly linked to the observed phenomena of interest.

Real-time experiments for characterizing SSB materials, whether *in situ* or *operando*, provide important insight into the evolution of battery materials under application-relevant conditions. Because the performance of batteries is often determined by how materials behave under dynamic conditions, these experimental strategies have become critical for understanding and advancing battery systems. For SSBs, a variety of *in situ* and *operando* techniques are used to visualize reaction dynamics, such as *in situ* transmission electron microscopy (TEM)^127,132,339^ and X-ray imaging,^133,315^ or probe chemical properties, such as X-ray absorption spectroscopy,^340^ Raman spectroscopy,^341^ and solid-state nuclear magnetic resonance (NMR).^342^

#### 4.2.2 *In Situ* and *Operando* Experimental Design Considerations for SSBs

A critical aspect of *in situ* and *operando* characterization is the design of the characterization cell and/or the control of experimental conditions to be representative of the electrochemical scenario being investigated. For SSBs, the phenomenon being observed is typically the evolution of the structure, chemistry, or morphology of electrode or SSE materials.^26,343^ The features that distinguish *operando* from *in situ* experiments are either the cells themselves (for instance, some *in situ* experiments are performed in characterization cells that are much different than conventional electrochemical cells) or the testing parameters used (such as current density, stack pressure, or electrode/SSE thickness). Because of these requirements, some characterization techniques do not readily lend themselves to *operando* or *in situ* experiments. Here, we briefly discuss the opportunities and limitations of *in situ* and *operando* characterization of SSBs, as well as the overarching factors that must be considered for successful application of these types of experiments. The specifics of characterization cell design required for each technique will be discussed in their respective subsections later in this section.

SSBs have a distinct advantage over traditional liquid-electrolyte batteries for certain types of *in situ*/*operando* characterization: there is no liquid in the system. Liquid electrolytes are largely incompatible with the vacuum conditions required by many characterization techniques, such as electron microscopy and X-ray photoelectron microscopy (XPS).^344^ The solid nature of SSEs largely mitigates this concern. While creative experimental design can enable liquid electrolytes to be contained and used in vacuum environments (such as liquid cell TEM holders),^344−347^ it is usually more straightforward to investigate SSBs with such techniques.

A challenge for *in situ* and *operando* characterization of SSBs is the buried nature of the materials or interfaces to be investigated.^343,348^ In general, SSEs or current collectors cover the electrode surface, and techniques which require surface interrogation (such as scanning electron microscopy (SEM), time-of-flight secondary ion mass spectrometry (ToF-SIMS), or atomic force microscopy (AFM) can have limited utility. For optical imaging experiments, the opaque nature of most SSEs limits viewing,^349^ while optically transparent liquid electrolytes allow for *in situ* visualization of important electrochemical phenomena (such as Li growth and stripping behavior) with optical microscopy.^350^ Additionally, many laboratory SSBs require an externally applied stack pressure to operate, and bulky pressure jigs can take up space that could otherwise be used to enable characterization within a cell.^13,77^ However, *in situ* characterization of SSBs without applied pressure can still provide useful information, as discussed in the TEM imaging subsection.^351^

*In situ* cell designs should be able to enable the observation of phenomena of interest while also protecting the SSB materials from environmental degradation. Traditional electrochemical cell housings are not necessarily required, and many *in situ* experiments on SSBs use cells that feature exposed SSE surfaces with a conductive probe to produce the desired electrochemical reaction only at a defined point for imaging or spectroscopic analysis (Figure 8b). Provided that materials are stable in air, or if the experiment is performed under vacuum or in inert atmosphere, such a method can produce useful *in situ* results without the complicated engineering of an *operando*-capable cell housing. With inventive experimental design, most characterization techniques can be performed *in situ* unless they require destruction of the sample (like FIB-SEM).

For *operando* experiments, custom cell housings are required that differ depending on the technique used. For transmission- and scattering-based techniques, the housing walls may need to be thin enough to allow for signal penetration and escape (as with X-ray absorption or optical microscopy) (Figure 8c), but they also need to support any applied stack pressure and protect air-sensitive materials from the external atmosphere. For bulk mechanical measurements, the SSB cell can typically be assembled with a built-in detector that does not require alterations to SSB cell housing dimensions. In all cases, care must be taken in designing cells for *operando* experiments, as the modified housing can affect the electrochemical performance of the SSB.

### 4.3 Imaging Techniques

This section provides an overview of imaging techniques that have been used for SSB research. The basics of the methods are provided, as well as key results that have been obtained with these methods.

#### 4.3.1 Optical Microscopy

Optical microscopy is perhaps the most long-standing, accessible, and widely used imaging characterization technique. It has seen extensive use in electrochemical research,^352^ and it is particularly valuable for investigating conventional Li-ion battery systems due to the optical transparency of the liquid electrolyte.^350^ The imaging resolution of optical microscopy (∼250 nm at best) is worse than techniques like X-ray or electron microscopy but greater than others such as magnetic resonance and neutron imaging (Figure 5). Owing to its availability and ease of use, optical microscopy has seen extensive utilization in SSB research and has unique applications because of the mechanisms by which contrast is generated.^353^ Optical color contrast is useful for discerning different parts of cells and can also be used to identify different phases during battery transformations.^354^ Because *ex situ* optical imaging is rather widespread, here we focus on *in situ* and *operando* optical characterization focused on SSBs.

An important consideration for the design of *in situ* and *operando* optical microscopy strategies for SSB cells is that light cannot penetrate optically opaque materials unlike X-rays, neutrons, and magnetic fields. As such, the SSB cell housing used for these experiments must typically have an optically transparent window. This could be a transparent current collector for top-down imaging or a transparent cell wall for cross-sectional imaging. Alternatively, a cell could contain an exposed surface if the materials are chemically stable in the testing atmosphere. Transparent windows can be avoided if material morphology is imprinted through covering materials such as a current collector.^355^ Care should be taken so that implementation of a transparent window or other cell modification does not alter the electro-chemo-mechanical behavior of the SSB materials during operation.

**Figure 9:**
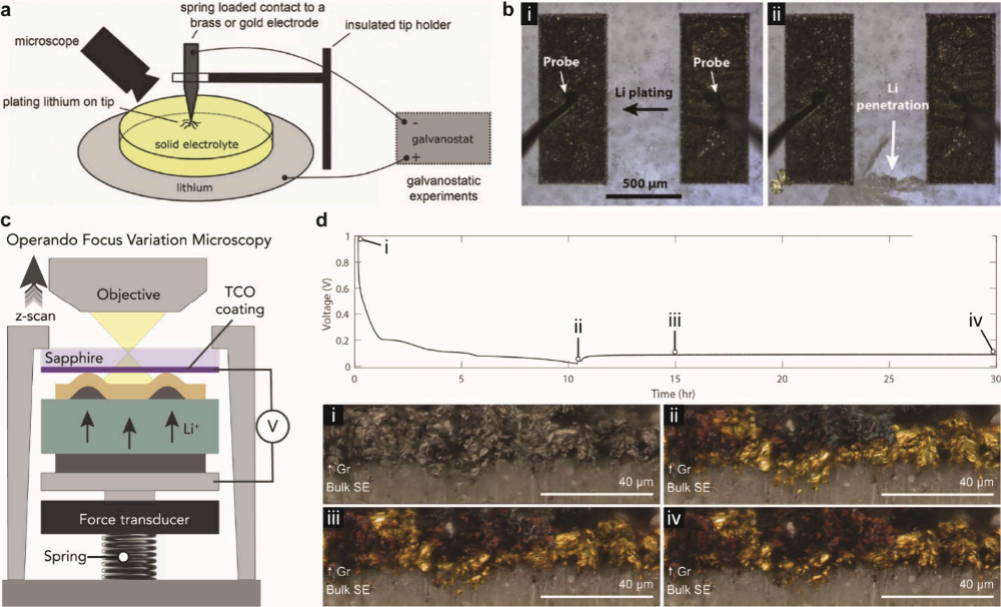
Optical microscopy of SSBs. (a) Diagram of a common method for *in situ* and optical imaging of SSBs where the surface of the SSE is exposed and current is passed through an electrical probe to allow for imaging of Li deposition at the probe tip. Reproduced with permission from ref (358). Copyright 2017 WILEY-VCH Verlag GmbH & Co. KGaA. (b) LLZO SSE with two Li electrodes before (i) and after (ii) Li penetration due to biasing by external probes using *in situ* optical microscopy. Adapted with permission from ref (363). Copyright 2020 Elsevier. (c) Diagram of an *operando* optical microscopy SSB housing used for imaging through a transparent conducting oxide window. Reproduced with permission from ref (355). Copyright 2022 Elsevier. (d) Voltage profile (top) and *operando* cross-sectional optical imaging (bottom) of a graphite/LPSC composite electrode in the following states: pristine (i), lithiated (ii), 5 h rest (iii), and 20 h rest (iv). Reproduced with permission from ref (367). Copyright 2024 Royal Society of Chemistry.

Significant effort has gone into characterizing the growth of Li metal using *in situ* and *operando* optical imaging at both the anode/SSE interface^355−358^ and within SSEs as filaments or dendrites.^175,359−363^ Such experiments require unique cell designs to enable electrical control and simultaneous optical imaging of Li growth. A diagram showing a typical setup for such an experiment is shown in Figure 9a, where a spring-loaded electrical probe is contacted to the SSE to apply a bias and allow for the *in situ* visualization of Li growth at this point.^357,358^ Other work has visualized SSB cross-sections to observe the behavior of Li metal anodes.^356,359,361,362,364^ Alternatively, Li electrodes can be placed on the top surface of a SSE with a lateral displacement between them, and applying a bias can cause Li to grow.^175,360,363,365,366^ Such a method was used by Kazyak et al. for dynamic optical imaging of Li dendrite growth through LLZO and LPS SSEs (Figure 9b).^363^ In other work, the deposition of Li metal in an anode-free cell configuration was carried out by imaging the back of a current collector to observe the evolving shape of Li domains as they formed and grew while applying stack pressure with a transparent conducting oxide window (Figure 9c).^355^

As in conventional Li-ion batteries, phase transformations in graphite have been studied in SSBs using optical microscopy, taking advantage of the drastic color shift as graphite is lithiated.^354^ Graphite composites containing LPS^368,369^ and LPSC^349,367,370^ SSEs have been studied using *operando* optical imaging. Liao et al. revealed the state-of-charge relaxation of a lithiated graphite/LPSC composite electrode when left at open circuit due to the diffusion of Li among graphite particles (Figure 9d).^367^

*Operando* optical microscopy has also been used to observe the effects of applied external stress on Li growth in the SSE,^175^ as well as to measure the magnitude of internal stresses due to filament growth.^365^ One such *operando* experiment demonstrated that applied stress controls the filament propagation direction; furthermore, this study showed that an applied transverse load can entirely deflect the growth of Li filaments through LLZTO SSE.^175^ Other work has utilized the dependence of the refractive index of a material on mechanical strain (the photoelastic effect) to directly measure the evolving 2D stress state in the vicinity of Li filament-driven cracks within LLZTO SSEs.^365^ Information on the internal stress and strain within SSB materials can also be obtained using digital image correlation (DIC), where subsequent images in an *operando* or *in situ* experiment are compared to the initial image to measure the displacement of each pixel from its original state (thus measuring strain and calculating stress). This technique has been employed in multiple *in situ* optical microscopy experiments to observe strain evolution in LLZTO,^371^ LAGP,^372,373^ and Li halide^374^ SSEs.

*In situ* optical imaging has also been used to investigate Li–S SSBs.^375−377^ Using a custom SSB cell with a window for cross-sectional optical viewing, a variety of phenomena have been investigated, including the deleterious effects of polysulfide shuttling,^375^ the temperature dependence of this degradation mechanism,^377^ and the effects of Li salt additives^376^ in quasi-SSEs composed of LLZTO and ionic liquid components. These studies highlight the versatility of *in situ* optical imaging for investigating multiple degradation mechanisms in the same electrochemical system.

#### 4.3.2 X-ray Computed Tomography

X-ray computed tomography (XCT) is a common technique in medical imaging, and it has also seen extensive use in other fields due to its nondestructive nature and ability to access and image buried features within materials or objects.^378−381^ XCT operates by rotating a sample under X-ray illumination (either monochromatic or polychromatic) and capturing 2D projection images at intervals during this rotation. After the X-rays are transmitted through or fluoresce from the sample (depending on the collection mode being used), they pass through a scintillator to be converted to visible light, which is then focused through a lens and detected at a camera. Contrast is generated either through attenuation or phase contrast. Attenuation (or absorption) contrast tomography records differences in the resulting amplitude of the X-rays due to attenuation from the imaginary portion of the refractive index of the sample, while phase contrast tomography records the phase-shift of X-rays passing through the sample resulting from the real portion of the material refractive index.^380^ A key difference in these contrast generation modes is their dependence on atomic number, where attenuation contrast is related to Z^3^ and phase contrast is independent of atomic number (though it still depends on electron density, which is affected by the atomic number).^381^ Because of this difference, phase contrast imaging can generally improve contrast of low atomic number elements (like Li) as compared to attenuation contrast imaging. Once the set of 2D projection images with their contrast information is collected, an algorithm is employed to reconstruct the projections into a 3D tomograph, creating a 3D volumetric data set of the sample.

The capability to nondestructively generate high-resolution tomographs sets XCT apart from other 3D imaging techniques like FIB and ToF-SIMS, which require the ablation of material from the sample to generate tomographs. The resolution/voxel size of XCT imaging is highly versatile, and it depends on the nature of the X-ray source, optics/detector, and experimental setup, generally ranging from voxel sizes of tens of nanometers for nano-CT to tens of microns for micro-CT (Figure 5). Sample size and volume also influence voxel size; obtaining high spatial resolution generally reduces the field of view available for imaging, which must be balanced with sample size. Finally, the requirement to collect multiple projection images limits the temporal resolution of XCT compared to continuous imaging techniques like optical and electron microscopy, as the sample rotation and projection image collection process can require several minutes of time even with high-flux synchrotron X-ray sources.

**Figure 10:**
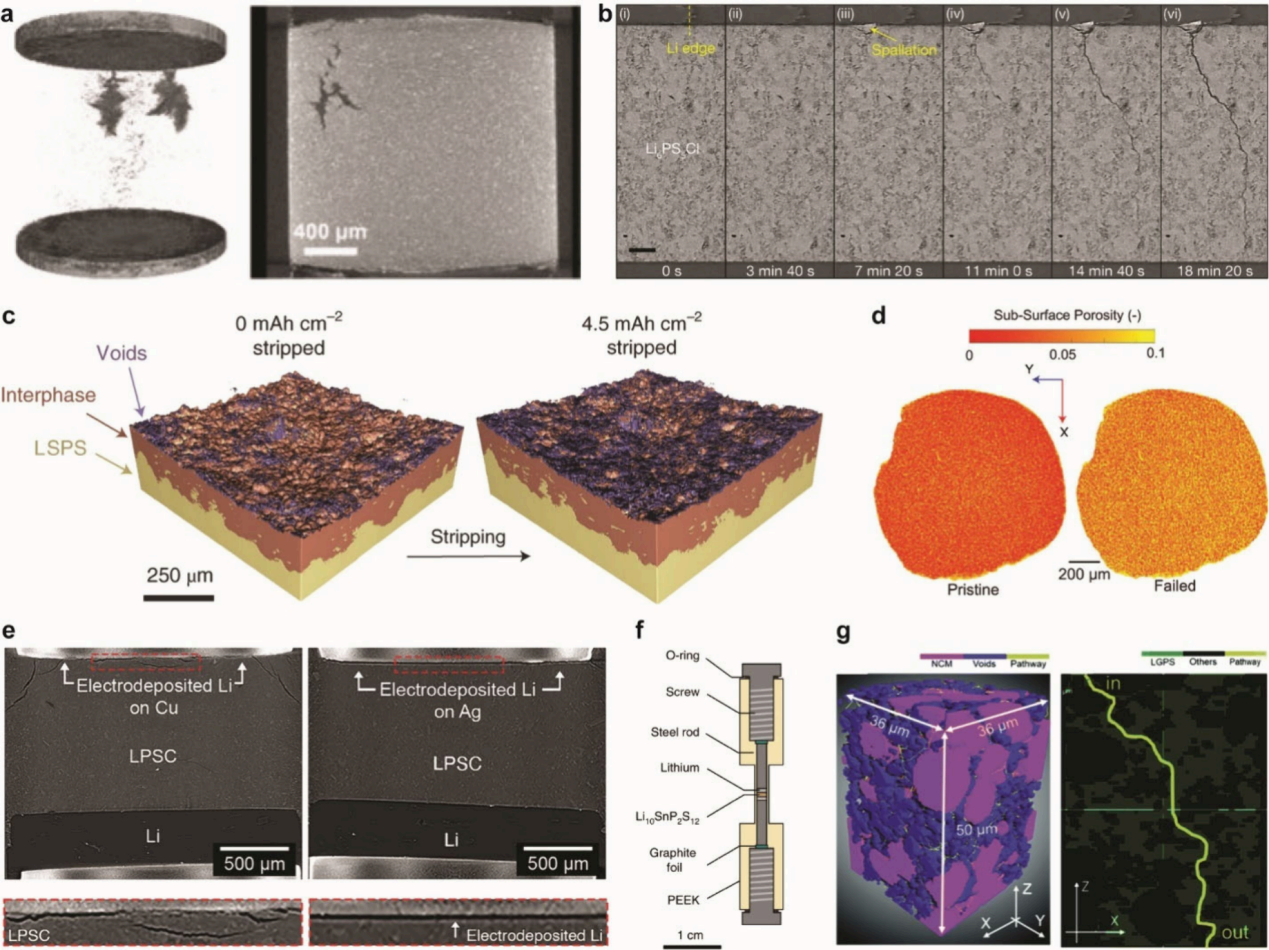
Examples of XCT investigation of SSB materials. (a) *Ex situ* benchtop XCT of a Li dendrite grown through an SSE with the electrolyte hidden (left) and shown (right). Reproduced with permission from ref (311). Copyright 2019. WILEY-VCH Verlag GmbH & Co. KGaA. (b) *Operando* synchrotron XCT cross-sectional images showing a Li dendrite growing from one side of a symmetric cell to the other through LPSC SSE. Scale bar is 100 μm. Reproduced with permission from ref (167). Copyright 2023 Springer Nature. (c) Reconstructed 3D volumes of a Li–LSPS interface in a SSB in the pristine (left) and stripped (right) states, showing voids as purple. Adapted with permission from ref (133). Copyright 2021 Springer Nature. (d) Projections of subsurface porosity on the Li electrode side of LLZO SSE before (left) and after (right) cell failure generated from *in situ* synchrotron XCT data. Reproduced with permission from ref (389). Copyright 2020 American Chemical Society. (e) Synchrotron XCT cross-sections of plated Li layers in anode-free SSBs on bare Cu (left) and 100 nm evaporated Ag interlayer on Cu (right) current collectors. Reproduced with permission from ref (315). Copyright 2023 Elsevier. (f) Schematic of an *operando* XCT SSB cell housing design. Reproduced with permission from ref (133). Copyright 2021 Springer Nature. (g) Segmented 3D volume (left) and simulated ion conduction pathway (right) of an NMC/LGPS composited cathode. Reproduced with permission from ref (390). Copyright 2022 Royal Society of Chemistry.

The ability of X-rays to penetrate electrochemical cells and generate 3D tomographs with relatively high spatial resolution has made XCT a valuable tool for the *in situ* and *operando* characterization of SSB cells (Figure 5). XCT has proven to be useful for observing buried interfaces between electrodes and SSEs during charge and discharge of battery cells. The contrast between different materials obtained with XCT also allows for quantification of the 3D distribution of different materials and defects in a system, as well as the evolution of these materials when experiments are performed *in situ* or *operando*. Whereas *ex situ* XCT with micron-scale resolution can be performed on extracted portions of materials from a cell, *in situ* and *operando* experiments require a battery housing with a very thin diameter to enable sufficient X-ray transmission (Figure 10f). Such cells commonly have external diameters of several millimeters at most (including the SSB materials and the housing walls on either side) in the region where X-rays will pass through the sample, which can enable the entire cell stack to be in the field of view of the imaging.^133,168^ The remainder of the battery housing need not be as thin, and wider diameters outside of the X-ray window are commonly used to allow for the inclusion of pressure-application rods or screws and sealing gaskets to protect the battery materials from the atmosphere during X-ray imaging (Figure 10f).^133,382^

There are two varieties of XCT that differ based on the nature of the X-ray source. “Benchtop” XCT is conducted using a laboratory instrument in which X-rays are generated in an X-ray tube by accelerating electrons in a vacuum between a cathode and an anode under high voltages (many tens or hundreds of kV). Benchtop XCT can achieve nanoscale spatial resolution, and commercial benchtop XCT systems feature integrated user interfaces. Despite high spatial resolution and ease of access, the temporal resolution of benchtop XCT is a limiting factor. Collecting sufficient projection images for high quality tomographic reconstruction can require many hours on a benchtop XCT, severely limiting *operando* experimental capabilities. This limitation arises because X-ray tube sources generate a much lower X-ray flux than other sources such as synchrotrons, requiring more time per scan to collect enough X-rays to generate the desired contrast for each 2D projection. *In situ* experiments can still be performed with benchtop XCT, but electrochemical cycling must generally be paused during imaging.^293^

Synchrotron XCT offers some advantages compared to benchtop XCT. X-rays generated from a synchrotron source are generated with much greater flux than an X-ray tube, allowing for faster projection imaging (down to several minutes). This capability to generate 3D images every few minutes has established synchrotron XCT as a powerful *operando* technique for SSB research. The drawback of synchrotron XCT is that its use is limited by the scarcity of synchrotrons and the limited access time available. Both methods thus have their own benefits and drawbacks, and both synchrotron and benchtop XCT have provided important new understanding of the mechanisms that govern SSB operation. However, synchrotron XCT has been used more commonly in SSB research, especially for *operando* studies.

To visualize materials and interfaces within SSBs using *in situ* or *operando* XCT, there are two important considerations for the design of the cell housing. First, the battery stack itself as well as the housing surrounding it should fit within the field-of-view of the X-ray beam so that the entire active region can be visualized.^379^ For high-resolution micro-CT, this can require a total cell width of several millimeters at most, while nano-CT requires cell sizes on the order of hundreds of microns. Second, judicious choice of cell walls and other materials is needed to ensure minimal interaction with the X-ray beam. Selecting a material with high X-ray transmission at the beam energy of interest and using cells with thin walls can ensure that significant X-ray attenuation is not caused by the cell housing itself as compared to the battery materials of interest.

The growth of Li dendrites through SSEs has been investigated with synchrotron XCT. The relatively low grayscale intensity of Li compared to other heavier elements when imaged with X-rays provides strong contrast for identifying Li. Benchtop XCT has been used to visualize Li growth within SSEs *in situ*.^311,383^ A study by Doux et al. highlights the ease with which a Li dendrite and the Li electrodes can be distinguished from the SSE (Figure 10a).^311^ Other researchers have conducted *in situ* and *operando* XCT experiments on Li dendrite growth through SSEs using synchrotron XCT, with various studies on LLZO,^384,385^ LPS,^386−388^ and LPSC.^167,168^ A recent *operando* synchrotron study captured the initiation of a dendrite caused by spallation cracking at the Li–SSE interface and subsequent propagation of a crack through the LPSC SSE by wedge opening (Figure 10b).^167^

In addition to the growth of dendrites, the chemo-mechanics of Li plating and stripping at its interface with the SSE has also been investigated with XCT. Using *operando* synchrotron XCT, Lewis et al. observed the dynamic formation of micron-scale voids at the Li–LSPS interface during stripping of the Li electrode (Figure 10c).^133^ This work demonstrated the ability of XCT to isolate voids from bulk Li and the SSE and was the first to quantify the dynamic evolution of voids at the Li–SSE interface. Synchrotron XCT has also been used *in situ* to observe the effects of pores in the SSE on the behavior of Li metal plating and stripping. Studying the LLZO SSE, Dixit et al. used *in situ* synchrotron XCT combined with image segmentation to visualize and quantify the distribution of subsurface micron-scale pores in the SSE and correlate this to degradation of the Li–LLZO interface (Figure 10d).^389^ Sandoval et al. used synchrotron XCT to observe the morphology of plated Li electrodes in anode-free SSBs (Figure 10e). This work showed that a Ag or Au interlayer at the current collector allowed for uniform plated Li thickness.^315^ Other benchtop XCT studies have also explored the chemo-mechanical behavior of the Li–SSE^169^ and Na–SSE^391^ interfaces. Such experiments have demonstrated the imaging of micron-scale voids at the Li–LPSC interface in cells that were cycled at varying temperature and pressures.^392^ While the dynamic formation of interfacial voids is not easily explorable with benchtop XCT due to the expensive time requirements for high-resolution scans, this study highlights the efficacy of benchtop sources for *ex situ* investigations.

Beyond the observation of physical or morphological changes, XCT can be used to identify the formation of new phases or microstructural evolution if the density of a material changes during a transformation process. When combined with image segmentation methods, identifying different phases based on their texture and grayscale intensity is possible.^393^ As an example, the formation of an interphase layer at the anode–SSE interface is a common phenomenon due to the electrochemical instability of many SSEs. This has rarely been observed with benchtop XCT, though one such *in situ* study was conducted by Tippens et al. on the very thick interphase formation that occurs in LAGP.^293^ O*perando* synchrotron XCT has been used to investigate interphase formation in greater detail.^133,394^ Lewis et al. used *operando* synchrotron XCT to observe the dynamic reaction of LSPS with Li metal to form an interphase layer during electrochemical cycling using a custom-made *operando* XCT cell (Figure 10c and 10f).^133^ The distribution of Li within alloy anode materials such as tin has also been observed, with contrast generated due to the lower grayscale intensity of the lithiated phase compared to the pure material.^382^ Even without the formation of new phases, the evolution of microstructure,^300,395^ such as the closing of voids in a SSE under compression due to electrode volume changes, has been observed.^300,396^

XCT has also proven useful for quantifying pore space in solid-state composite cathodes to support electrochemical and mechanical modeling of such systems. When performed *ex situ* with benchtop XCT,^8,397^ this allows for direct measurement of the distribution of components within a composite electrode. Using *ex situ* benchtop XCT and image segmentation, Huang et al. additionally used the distribution of the components in an NMC-811/polymer composite cathode to create a Li-ion flux model for the electrode.^8^ The effectiveness of such models can be improved when created from *in situ* and *operando* synchrotron XCT experiments due to the additional information gathered at multiple states of charge for the same region of the composite electrode.^390,398^ For example, Sakka et al. demonstrated the *operando* control of both electrochemistry and stack pressure of an NMC-111-LGPS composite cathode SSB in a synchrotron XCT experiment, allowing for ion tortuosity simulations to be performed for different stack pressures (Figure 10g).^390^

Finally, synchrotron XCT is well suited for use in tandem with spectroscopic techniques due to relatively fast scan times. Such multimodal characterization is useful for complementing XCT data with chemical and/or structural information. XRD^394,399−401^ and XANES^402−406^ have both been used simultaneously with synchrotron XCT to characterize SSB materials during *in situ* and *operando* experiments, such as using X-ray diffraction microscopy with *in situ* synchrotron XCT for studying LLZO.^401^ This study revealed the stochastic nature of failure in LLZO grains, potentially resulting from local concentration inhomogeneity, and provides a useful basis for performing such tandem experiments on SSBs. Alternatively, synchrotron CT-XANES is well suited for studying the 3D chemical composition of SSB electrodes during electrochemical cycling.^404−406^ Future research on other SSB materials with these techniques would be useful, as well as the use of alternative spectroscopic techniques to combine with CT imaging, such as total scattering computed tomography.^407^ Such multimodal analysis may help understand the differences in bulk-scale stress and reaction homogeneity between liquid and solid-state systems and between different SSB chemistries.

As demonstrated in this section, benchtop and synchrotron XCT techniques are quite valuable for SSB research, and they have been used for observing the chemo-mechanical behavior of Li metal and quantifying the dynamic formation of interphase and voids in SSBs. While a variety of XCT studies have been carried out on Li metal anode systems and composite oxide cathodes, relatively little work has focused on other high-capacity electrodes (such as alloy anodes or S). Performing *in situ* and *operando* characterization of these systems would further improve our understanding of next-generation SSB materials by exploring chemo-mechanical degradation mechanisms, mechanical failure modes, and electrochemical interphase reactions.

#### 4.3.3 Neutron Imaging

Neutron imaging is similar in principle to X-ray imaging, though far less common for SSB research. In this technique, neutrons are generated from a source and then transmitted through a sample, and they are collected to create an image. Neutron imaging differs from X-ray imaging because X-rays interact with the electron cloud of atoms, while neutrons interact with the nucleus.^408^ As such, neutrons attenuate with different trends across the periodic table compared to X-rays. For instance, certain light elements (such as H and Li) are more sensitive to neutrons than various heavier elements, whereas the reverse trend is true for X-rays. Neutrons are also sensitive to atomic isotopes whereas X-rays are not, providing opportunities for in-depth studies of systems in which different elemental isotopes are intentionally added to generate contrast. Contrast in neutron imaging is generated similarly to XCT, where neutrons attenuate and/or scatter within the sample to create contrast in the image. Neutron images are typically generated by converting the transmitted neutron beam into a different signal; this is accomplished by capturing the neutrons with a neutron-absorbing material (like Li) and recording the emission of X-rays that are produced.^408^ In this manner, 2D projection images of a sample are recorded. By rotating the sample and collecting these 2D projections at uniform intervals along the rotation, a 3D neutron tomograph can be reconstructed from the 2D projections to generate a volumetric image data set.

Neutron imaging has infrequently been used in SSB research due to its relatively poor spatial resolution (typically tens of microns at best) (Figure 5). Additionally, small neutron-generating devices are uncommon and are limited greatly in neutron flux compared to large-scale reactor- or accelerator-based neutron sources, limiting the availability of neutron imaging for many researchers. Despite this, neutron imaging has a key advantage for battery systems over X-ray imaging in that neutrons strongly interact with Li atoms in SSBs, whereas X-rays do not.^409^ Neutron imaging, then, has potential to be an important characterization technique for observing the distribution of Li within buried SSB materials *in situ* and *operando*. However, its limited resolution can require abnormally thick battery electrodes, as discussed below.

Neutron imaging, like XCT, is a nondestructive imaging technique where the signal can penetrate cells to provide information about buried materials and interfaces. Therefore, *in situ* and *operando* SSB cell design follows similar principles to those typically used for XCT experiments. For instance, the cell housing must be small enough to allow for the entire SSB to fit within the field of view of the neutron beam. Where these techniques differ in cell design is the optimal choice of material for the cell housing; because neutrons can interact more strongly with various lighter elements but can travel almost unimpeded through certain metals, selecting an appropriate material for the housing can prevent excessive neutron attenuation by the housing walls that lead to decreased signal from the SSB itself. While a nonconductive, chemically stable material must still be used for the cell housing walls to contact the SSB materials, this can be a thin sheathe within a larger, sturdier metal housing.

**Figure 11:**
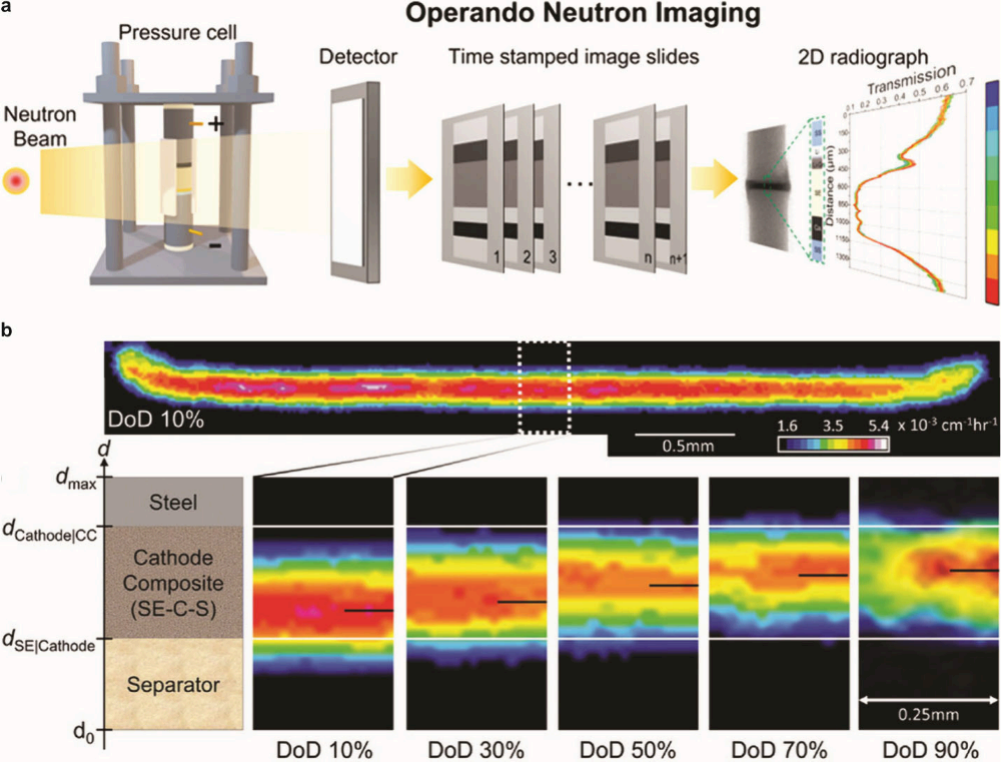
*Operando* neutron imaging of SSBs. (a) Diagram of an *operando* SSB cell design for neutron imaging. Reproduced with permission from ref (410). Copyright 2024 American Chemical Society. (b) *Operando* neutron imaging of a S/LPSC/C solid-state composite cathode at different states of charge with the concentration of Li overlaid. Reproduced with permission from ref (412). Copyright 2023 Wiley-VCH GmbH.

Cao et al. used *operando* neutron imaging to observe the distribution of Li in a graphite interlayer at the Li anode/sulfide electrolyte interface (Figure 11a).^410^ Their diagram highlights the limited resolution capabilities of neutron imaging, but also demonstrates the sensitivity of neutrons to the presence of Li. Bradbury et al. used *operando* neutron imaging to investigate the distribution of Li in a composite S cathode (Figure 11b).^411,412^ While they observed the uneven distribution of Li within the cathode during lithiation, they were forced to use a very thick (several hundreds of microns) cathode layer to achieve substantial differentiation with neutron imaging, which may not be representative of ideal SSB systems. Still, a thicker cathode layer is not unreasonable in some systems, and this work highlights the importance of selecting a system that is appropriate for the resolution capabilities of the imaging technique.

#### 4.3.4 Magnetic Resonance Imaging (MRI)

Magnetic resonance imaging (MRI) is a spatially resolved application of nuclear magnetic resonance (NMR) spectroscopy that allows for 3D reconstructions to be generated nondestructively. As detailed further in the NMR spectroscopy section, during NMR the sample is subjected to a strong magnetic field and a weak oscillating magnetic field using superconducting electromagnetic coils. Different elements have different resonant frequencies at which the nucleus vibrates depending on their atomic number and the atoms to which they are bonded; when the oscillating field reaches a resonant frequency, it will cause atoms of that element in the sample to absorb and release large amounts of energy as electromagnetic waves that can be recorded.^413^ In NMR spectroscopy, the relative magnitude of the response is plotted against the “chemical shift,” a value that is inversely dependent on the applied magnetic frequency. In MRI, however, a gradient magnetic field is applied across the sample to alter the resonant frequency of different locations of the sample; this allows for spatial mapping of the sample’s response to a given element’s resonant frequency by changing of the gradient field.^414^ This allows for visualization of the 2D distribution of the magnetic response in different regions of the sample. If a third axis is added to the movement of the superconducting coils, then 3D data sets can be generated showing the magnetic response throughout the entire volume of a sample.

The nondestructive nature and wide field-of-view of MRI have made it an indispensable tool in the medical field. In addition to being nondestructive, NMR (and MRI) have the added advantage of being able to identify specific elements in a sample by tuning the magnetic frequency to the characteristic resonance of the desired element (if it contains an isotope with a nonzero nuclear spin). As such, MRI can easily discern Li atoms from other elements in a sample because both ^6^Li and ^7^Li have nonzero spin, whereas X-ray-based techniques have much more limited capabilities in this regard. This is particularly useful in Li-ion battery research, where MRI has been used to some advantage.^415−417^ The main limitation of MRI is its spatial resolution (>100 μm at best), which is not sufficient for detailed observation of features within typical Li-ion battery electrodes. In addition to this, an MRI scan can take tens of minutes depending on the field of view used, making *operando* investigations difficult. Owing to these drawbacks, MRI has seen limited use in SSB research, much like neutron imaging. Its usefulness, however, is still highlighted in the examples provided below.

Owing to the nondestructive and penetrating imaging capabilities of MRI, effective cell designs for experiments using *in situ* and *operando* MRI of SSBs can be modeled after XCT cell designs. This is accomplished by creating a housing out of a material that will not strongly interact with the applied signal; in this case the housing material must not contain atoms of the element being imaged (such as Li). The material selection is the most important consideration to avoid interference with the applied oscillating magnetic field; beyond this requirement, the housing need only be small enough to allow for the sample to fit entirely within the MRI instrument and operate electrochemically during imaging.

**Figure 12:**
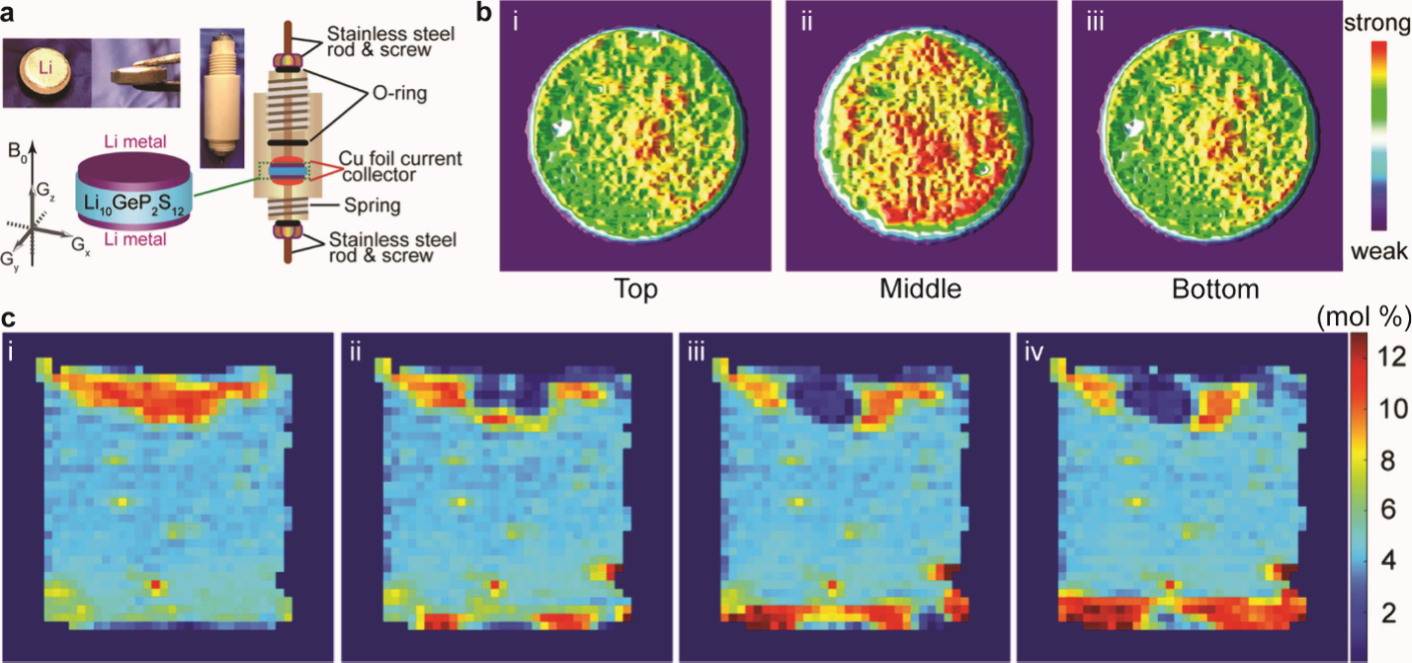
*In situ* MRI characterization of SSBs. (a) Schematic of an *in situ* MRI SSB cell housing. Reproduced with permission from ref (420). Copyright 2018 American Chemical Society. (b) MRI of a polymer-coated LGPS SSE showing the top, middle, and bottom of the cell and displaying the concentration of Li in the electrolyte. Adapted with permission from ref (420). Copyright 2018 American Chemical Society. (c) 2D MRI cross-sections of an OIPC Li-ion SSE after (i) 0 h, (ii) 17 h, (iii) 34 h, and (iv) 51 h of operation at −10 μA displaying the concentration of Li in the electrolyte. Adapted with permission from ref (421). Copyright 2016 American Chemical Society.

As mentioned, the most useful aspect of MRI is its ability to discern Li from other elements within SSBs with various materials and chemistries. MRI has been performed *ex situ* on several SSEs, namely solid polymer electrolytes^418^ and LLZTO.^419^ While the resolution of MRI is limited, its wide field of view allows for straightforward disassembly and *ex situ* imaging of a SSB to observe the distribution of the desired element. With a carefully designed cell, like the one shown in Figure 12a, such experiments can also be performed *in situ*. By constructing a symmetric Li-LGPS-Li SSB in a custom-made *in situ* MRI cell, Chien et al. observed the 3D distribution of Li within LGPS and PEO-coated LGPS SSEs (Figure 12a,b).^420^ An example of 2D cross-sections from a similar analysis are provided by Romanenko et al. in Figure 12c, where they performed *in situ* MRI on an organic ionic plastic crystal (OIPC) SSE to observe the concentration of Li within it during electrochemical cycling.^421^ The unique imaging capabilities of MRI offer promise as a diagnostic tool for research- and commercial-scale SSBs to observe regions of nonuniformity or dendritic growth in the SSE.

#### 4.3.5 Scanning Electron Microscopy (SEM)

Scanning electron microscopy (SEM) is a foundational imaging technique in many research fields, and it is widely used in battery research.^422^ SEM operates by generating electrons from a thermionic or field-emission source and accelerating them using a voltage difference (typically kilovolts) toward a sample. The electron beam is focused to be several nanometers wide with condenser lenses and is then rastered across the surface of the sample; images are created by collecting either the generated secondary electrons emitted from the surface or the elastically scattered primary electrons. SEM can be readily performed on most materials with greater spatial resolution than optical microscopy (down to several nanometers) (Figure 5). In addition, SEM can provide elemental information and crystallographic details through energy-dispersive X-ray spectroscopy (EDS or EDX) and electron backscatter diffraction (EBSD) analysis, respectively. As such, it has seen extensive use in SSB research.^313,423,424^

SEM imaging requires the sample to be inside a vacuum chamber, and there must be an exposed surface over which the electron beam can raster. SEM image contrast is affected by the atomic number, the surface morphology, and the electrical conductivity of the materials being imaged. Some materials can be damaged by the high-energy electron beam (for instance, some polymeric materials, Li metal, and sulfide materials), and care must be taken to understand and control beam damage.

We focus here on *in situ* SEM imaging methods that have been developed and used to observe SSB behavior during cycling. Multiple studies have used *in situ* SEM imaging to investigate the deposition and stripping of Li metal, with different cell architectures possible. For instance, a battery can be assembled with a current collector contacting the SSE, and SEM can be used to image changes of the shape of the current collector as Li plates beneath it^425,426^ or to observe the cross-section of the cell.^427,428^ A unique method of imaging a SSB cell with current supplied in this manner was demonstrated by Li et al., who employed a rotating cell that allowed for *in situ* imaging of the same region from multiple angles during Li plating.^287^

**Figure 13:**
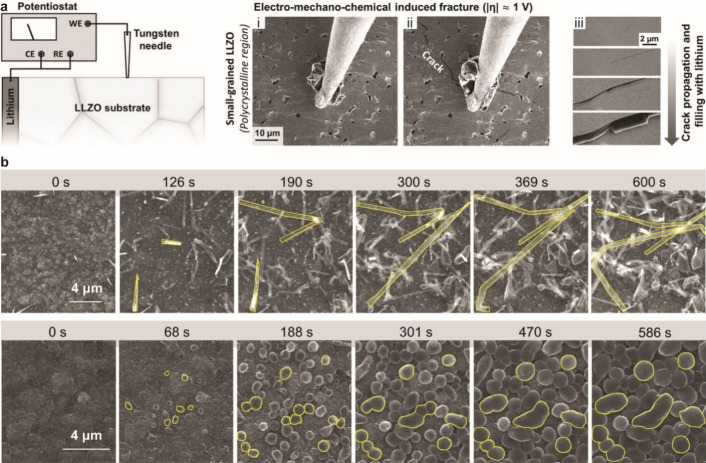
*In situ* SEM imaging of SSB materials. (a) Diagram (left) and images (right) of *in situ* plating of Li metal on an LLZO SSE, where the images show (i) the onset of Li plating, (ii) cracking of the LLZO driven by a Li dendrite, and (iii) time-series frames of the crack initiation and propagation driven by the Li dendrite. Adapted with permission from ref (429). Copyright 2020 WILEY-VCH Verlag GmbH & Co. KGaA. (b) *In situ* SEM time-series images of Li plating on Cu (top) and In (bottom) with yellow highlights showing the unique microstructures formed on the different substrates. Reproduced with permission from ref (430). Copyright 2022 American Association for the Advancement of Science.

A second cell design involves bringing a fine probe (usually tungsten) into contact with an SSE/counter electrode stack, and then applying current or voltage.^429,430^ This action causes electrochemical reaction(s) at the probe tip, and SEM imaging of this specific area allows for the experimenter to capture the material dynamics at this location. Krauskopf et al. observed the initiation and propagation of a crack in LLZO SSE during Li growth, finding that Li growth through the SSE was dependent upon the SSE microstructure rather than local current density (Figure 13a).^429^ Cui et al. utilized this technique to observe the nucleation and growth of Li on different metallic substrates *in situ* by supplying a current to the conductive substrates with the tungsten tip, demonstrating the importance of the Li-side current collector material for stabilizing Li growth to prevent dendrites (Figure 13b).^430^ While applying current with a probe does allow for isolation of features of interest, current constriction from the isolated current field and the lack of stack pressure on these cells may not fully replicate true cycling conditions.

*In situ* SEM has also been used to study failure modes in various SSEs, including solid polymer electrolytes^431^ and sulfide electrolytes.^432^ Rather than using a tungsten tip to apply current to a local area, cells of this type are typically studied with an exposed cross-section for *in situ* SEM imaging of a wide region of the SSE to increase the likelihood of observing failure mechanisms. Such a setup also allows for more reversible cycling of the cell as it is operating under typical stack pressure and current density conditions. Multiple failure modes in SSBs have been observed with *in situ* SEM imaging, including interphase formation,^431^ polysulfide shuttling,^433^ and mechanical degradation.^432^ Similar imaging could be performed on high-capacity electrodes, like alloy anodes and conversion cathodes, to improve the community’s understanding of degradation mechanisms of these systems.

#### 4.3.6 Focused Ion Beam (FIB)

While SEM is a useful tool on its own, pairing SEM with focused ion beam (FIB) milling is particularly advantageous for SSB research. FIB is a powerful technique used to precisely mill away portions of the sample to create thin specimens for electron microscopy or to reveal the internal structure of materials. FIB milling uses an ion beam (typically Ga, but also Xe or other elemental plasmas) to ablate away portions of a sample within a vacuum chamber. FIB milling is combined with SEM to enable high-resolution imaging of the milled specimens. FIB can also be coupled with other techniques such as EDS to determine elemental composition of specific regions within a sample.

FIB-SEM methods have played a major role in SSB research to probe buried electrode materials, SSEs, and interfaces *ex situ*. SSB cells can be electrochemically cycled followed by extraction and transfer of the cell stack to the FIB for characterization without the need for specialized cell housings. The cell stack remains intact during this process, preserving interface and material integrity. Typical milled regions using Ga FIB are tens of microns in width, which is usually sufficient to reveal electrode and SSE layers in SSBs (depending on their thickness). However, these relatively small milled regions can be unrepresentative of global behavior. Plasma FIB (PFIB) can enable significantly enhanced milling rates (up to 40 times faster).^434^ Due to its destructive nature, FIB characterization is primarily limited to *ex situ* evaluation of samples.

**Figure 14:**
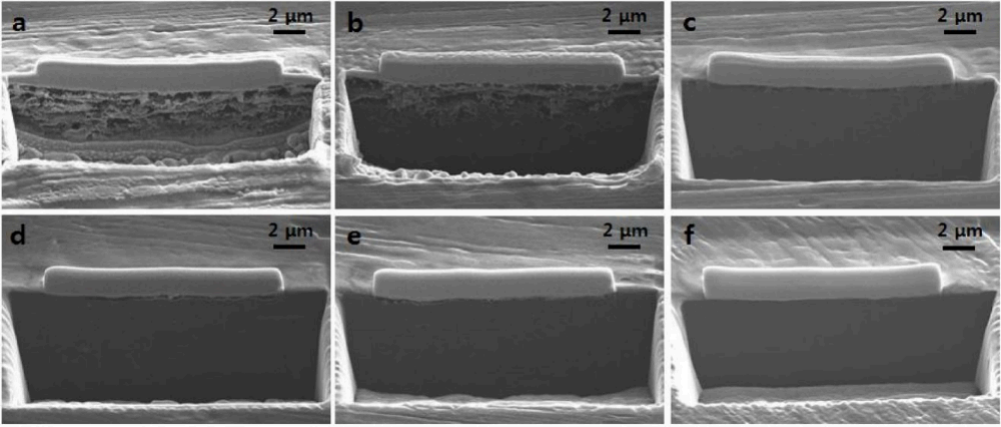
SEM images of Li metal foil after Ga-FIB milling at (a) room temperature, (b) 0 °C, (c) −50 °C, (d) −100 °C, (e) −150 °C, and (f) −170 °C. Adapted with permission from ref (435). Copyright 2019 American Chemical Society.

A challenge of FIB-SEM characterization of battery materials is the possibility of detrimental interactions between the ion beam and various materials of interest.^435^ For example, Li metal readily forms alloys with Ga at room temperature, which causes morphological/compositional changes and damage during milling instead of forming the desired milled structures.^436^ Borrowing from biological studies, cryogenic FIB (cryo-FIB) characterization has been widely used to minimize beam damage.^434,435^Figure 14 shows that Li metal is highly porous after milling at room temperature, whereas milling at lower temperatures resulted in more precise, cleaner cuts. Sulfide SSEs such as argyrodite LPSC can also be damaged and show redeposition under Ga milling at room temperature, with significantly improved milling behavior at cryogenic temperatures.^298^ PFIBs use a less reactive source than Ga FIBs, which allows for stable milling without necessitating cryogenic temperatures. These studies highlight the need to understand potential artifacts that can arise from the milling process to ensure that they do not affect data analysis.

**Figure 15:**
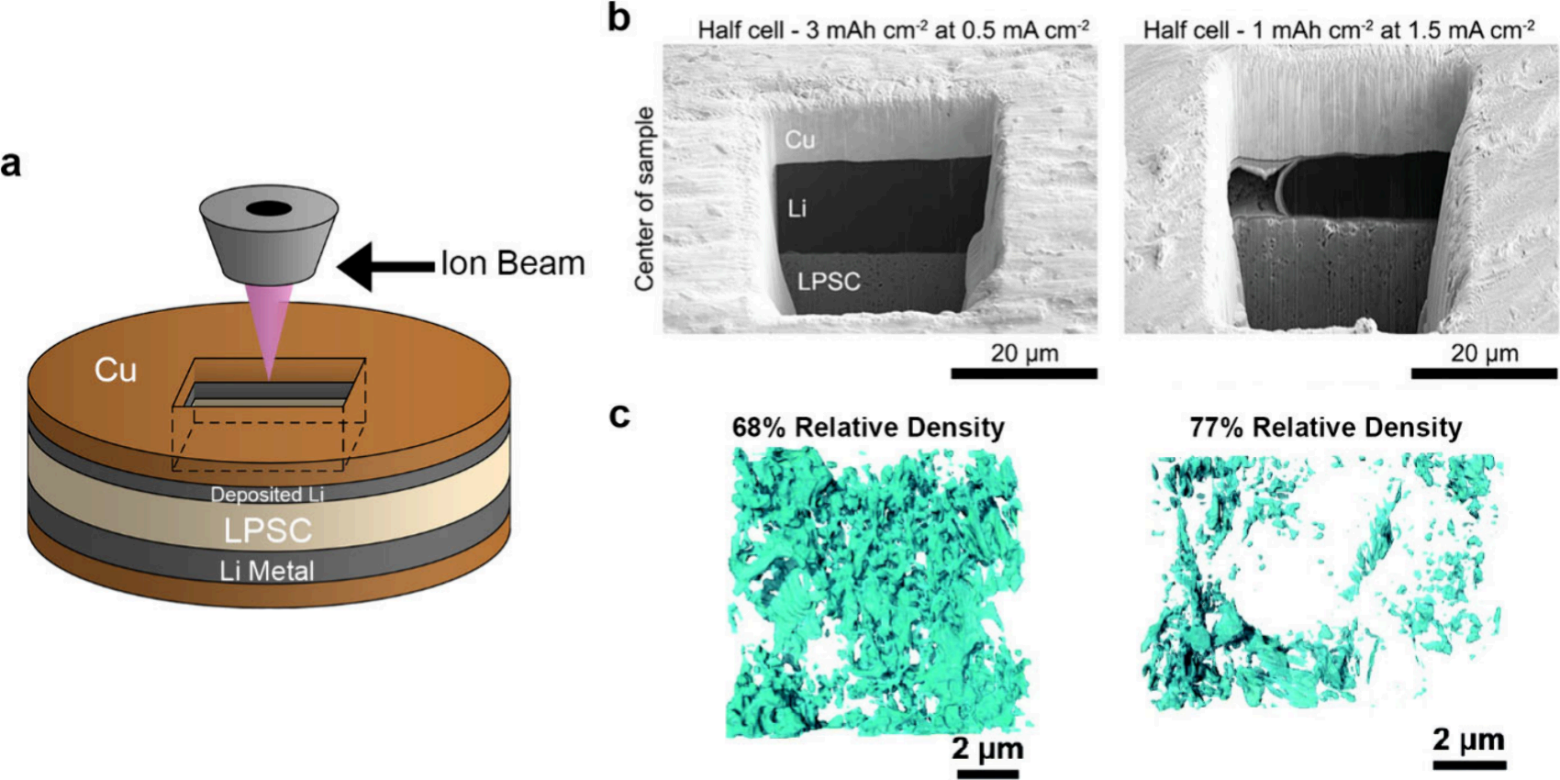
(a) Schematic of FIB milling of an anode-free SSB cell after Li deposition. Adapted with permission from ref (315). Copyright 2023 Elsevier. (b) Cryo-FIB reveals variation in Li thickness across the interface at high current densities, while greater thickness uniformities are achieved at low current density. Adapted with permission from ref (298). Copyright 2023 Wiley-VCH GmbH. (c) 3D volume of the SSE porosity from tomographic FIB-SEM, for two different samples pelletized at 50 MPa (left) and 370 MPa (right). Reproduced with permission from ref (314). Copyright 2020 Royal Society of Chemistry.

Cryo-FIB-SEM has recently been used to image Li grown in anode-free SSBs^289,298,315,355,437^ (Figure 15a). FIB milling was carried out from the back of a Cu current collector to reveal the Li morphology between the SSE and the current collector. These experiments showed that Li deposition was nonuniform at high current density, with increased thickness uniformity at lower current density (Figure 15b).^298^ Cryo-FIB imaging has also been used to visualize the evolution of metallic interlayers in anode-free SSBs, revealing that Ag and Au interlayers exhibit evolving morphology during Li growth.^315^

FIB-SEM characterization can be further used for 3D tomographic visualization. Sequential milling away of thin slices and interspersed SEM imaging allows for a library of 2D images to be assembled, which can be combined into a 3D data set. This characterization allows for quantitative analysis of parameters such as porosity and surface area, such as the investigation in Figure 15c showing the effect of pressure on relative density of sulfide electrolytes.^314^ Additionally, FIB tomography has been used to study the influence of microstructure on ionic transport in composite cathodes.^438,439^ This technique enables the morphology/microstructure of composite cathodes to be tracked over time, allowing for visualization of the active material, pore formation, and/or void space. Image segmentation is often done to quantify the evolution of different microstructural features. Pairing the 3D physical microstructure of the composite from FIB tomography with other electrochemical techniques and modeling has helped further our understanding of transport characteristics within composite cathodes.

#### 4.3.7 Time-of-Flight Secondary-Ion Mass Spectrometry (ToF-SIMS)

ToF-SIMS operates by exposing a sample surface to an ion beam to eject material from the sample as ions. The time between the ion pulse and detection of the ejected material by a mass spectrometer, as well as the ion mass, are recorded to create compositional maps of the sample with resolution down to tens of nanometers (Figure 5). ToF-SIMS poses a unique advantage over other imaging techniques as it can readily identify the distribution of Li in a sample, whereas many X-ray-based techniques cannot since the Li–K characteristic radiation produced in such experiments is often weak and/or absorbed by detector windows. While 2D images are typically formed with ToF-SIMS, creating 3D reconstructions of sample volume is also possible at small scales via depth profiling. The identification of elemental composition at high resolution makes ToF-SIMS a powerful tool for characterizing the elemental composition of materials and interfaces in SSBs.

ToF-SIMS does have several limitations for SSB investigations. ToF-SIMS requires an exposed sample for analysis; thus, it is difficult to carry out *in situ* ToF-SIMS analysis of SSBs. Additionally, ToF-SIMS is a destructive technique due to the ablation required for mass spectrometry. Thus, *ex situ* ToF-SIMS is the most common mode for SSB research, but several *in situ* ToF-SIMS studies have been conducted.

**Figure 16:**
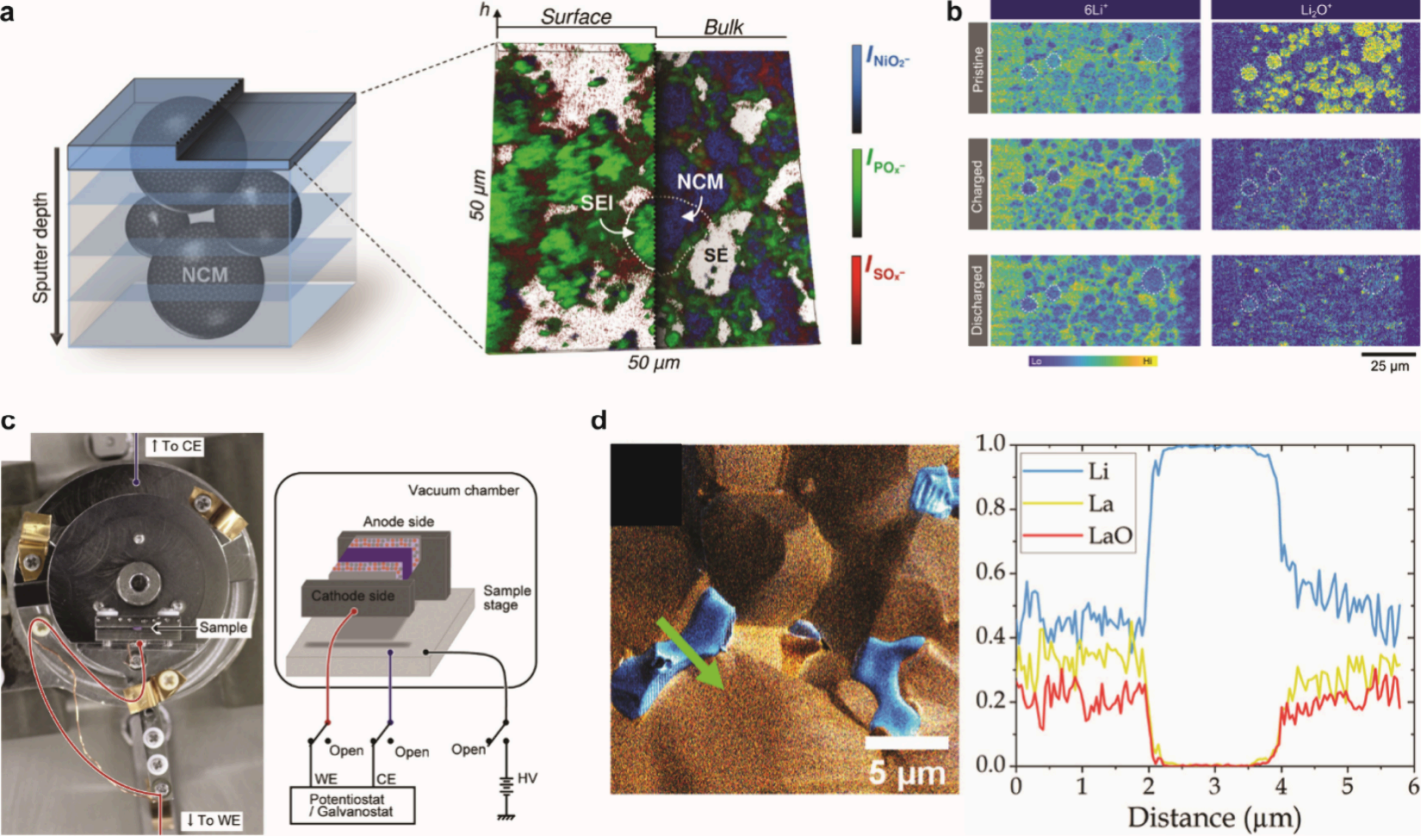
Examples of ToF-SIMS characterization of SSBs. (a) Diagram (left) of *ex situ* ToF-SIMS depth profiling of an NMC-622/LPSC composite solid-state cathode showing (right) the surface CEI layer and the bulk unreacted phases. Reproduced with permission from ref (442). Copyright 2019 American Chemical Society. (b) *In situ* ToF-SIMS of an NCA/LPS composite solid-state cathode in its pristine, charged, and discharged states, showing the concentration of Li (left) and oxidized Li (right) in the cathode. Reproduced with permission from ref (446). Copyright 2021 American Chemical Society. (c) Image (left) and diagram (right) of an *in situ* ToF-SIMS SSB cell design. Reproduced with permission from ref (447). Copyright 2018 Elsevier. (d) Merged elemental maps (left) of LLZO SSE after electrochemical cycling. The green arrow points to the horizontal location where a vertical line scan (right) of each element was performed. Reproduced with permission from ref (448). Copyright 2023 American Chemical Society.

ToF-SIMS has been used for analyzing the interphase formed between electrodes and the SSE. *Ex situ* investigations of interphases at both positive and negative electrodes^440−445^ have been conducted on a variety of electrode/SSE combinations. A representative example was performed by Walther et al. on a composite cathode consisting of a LiNi_0.6_Mn_0.2_Co_0.2_O_2_ (NMC-622) cathode active material and a Li_6_PS_5_Cl (LPSC) SSE (Figure 16a).^442^ Using ToF-SIMS depth profiling, they conducted 3D elemental mapping on the cathode composite in the pristine state and after 100 electrochemical cycles to identify the composition of the CEI layer between the NMC and LPSC. CEI layer formation was also observed with *in situ* ToF-SIMS elemental mapping for a LiNi_0.8_Co_0.15_Al_0.05_O_2_ (NCA) composite cathode with a 75Li_2_S·25P_2_S_5_ (LPS) SSE by Yamagishi et al. (Figure 16b).^446^

ToF-SIMS is also useful for analyzing the effects of environmental exposure on air- and moisture-sensitive SSB materials.^449,450^ The 3D depth profiling capability of ToF-SIMS allows for enhanced environmental stability analysis because it can reveal the depth at which degradation due to O or moisture in the atmosphere penetrates into the sample. *Ex situ* ToF-SIMS has been used to reveal the degradation of LPSC SSE in humid air^449^ and has provided insight into the air stability of the LPS SSE and Li metal interface when treated with a Li_*x*_SiS_*y*_ coating.^450^

*In situ* ToF-SIMS analysis of SSBs has been performed by clamping a presintered SSB composed of Li_1+*x*_Al_*x*_Ge_2–*x*_(PO_4_)_3_ (LAGP) SSE and symmetric composite electrodes consisting of LiCoPO_4_ (LCP) active material composites (Figure 16c).^447^ The clamp allowed for the application of stack pressure and electrical contacts, while the exposed edge provided access for *in situ* ToF-SIMS elemental mapping of the Li distribution in the SSB. Other researchers have similarly utilized *in situ* ToF-SIMS to observe the distribution of Li ions in different SSB materials, such as composite cathodes,^446^ composite anodes,^451^ and electrolytes (Figure 16d).^448^ There are opportunities to utilize ToF-SIMS for high-capacity SSB material systems such as anode-free configurations, Si anodes, and conversion cathodes; such investigations, whether *ex situ* or *in situ*, could provide key information on interphase composition and thickness in these systems.

#### 4.3.8 Atomic Force Microscopy (AFM)

Many scanning probe microscopy (SPM) techniques, including conductive AFM, Kelvin probe force microscopy (KPFM), and electrochemical strain microscopy, are variations of atomic force microscopy (AFM).^452^ While there are other forms of SPM that diverge from AFM, such as scanning tunneling microscopy, variations of AFM are the most common scanning probe techniques in SSB research.

AFM involves translating a sample underneath a cantilever with a fine tip that deflects as it interacts with the sample topography beneath it. The nanometer-level deflections of the tip can be detected with various methods, such as with a piezoelectric sensor or a laser deflected from the cantilever. By recording the deflection of the cantilever under different applied conditions (i.e., an electric bias, varied cantilever height, etc.), a nanometer-scale rendering of the surface topography and/or material property distribution (such as conductivity or Young’s modulus) can be generated. The ability of AFM and other SPM techniques to measure and map topographic, electrical, and mechanical properties rather than structural or chemical properties distinguishes it from many other characterization techniques. For battery materials, visualizing surface topography or electrochemical/mechanical properties makes AFM and SPM useful techniques.^453^

AFM has similar limitations to ToF-SIMS in that it requires an exposed sample surface. For *in situ* experiments, a custom cell design with an exposed surface must be created that may not be representative of a normal SSB system. As with most high-resolution imaging techniques, AFM also has a limited field of view (typically a few microns in any direction) (Figure 5). AFM is solely a surface technique; it can probe surface topography, but it cannot access material buried beneath the surface of the sample. Despite these limitations, AFM and its variations have been used in SSB research for both *ex situ* investigations of pristine and cycled materials as well as *in situ* investigations of evolving materials during electrochemical cycling.^454^

**Figure 17:**
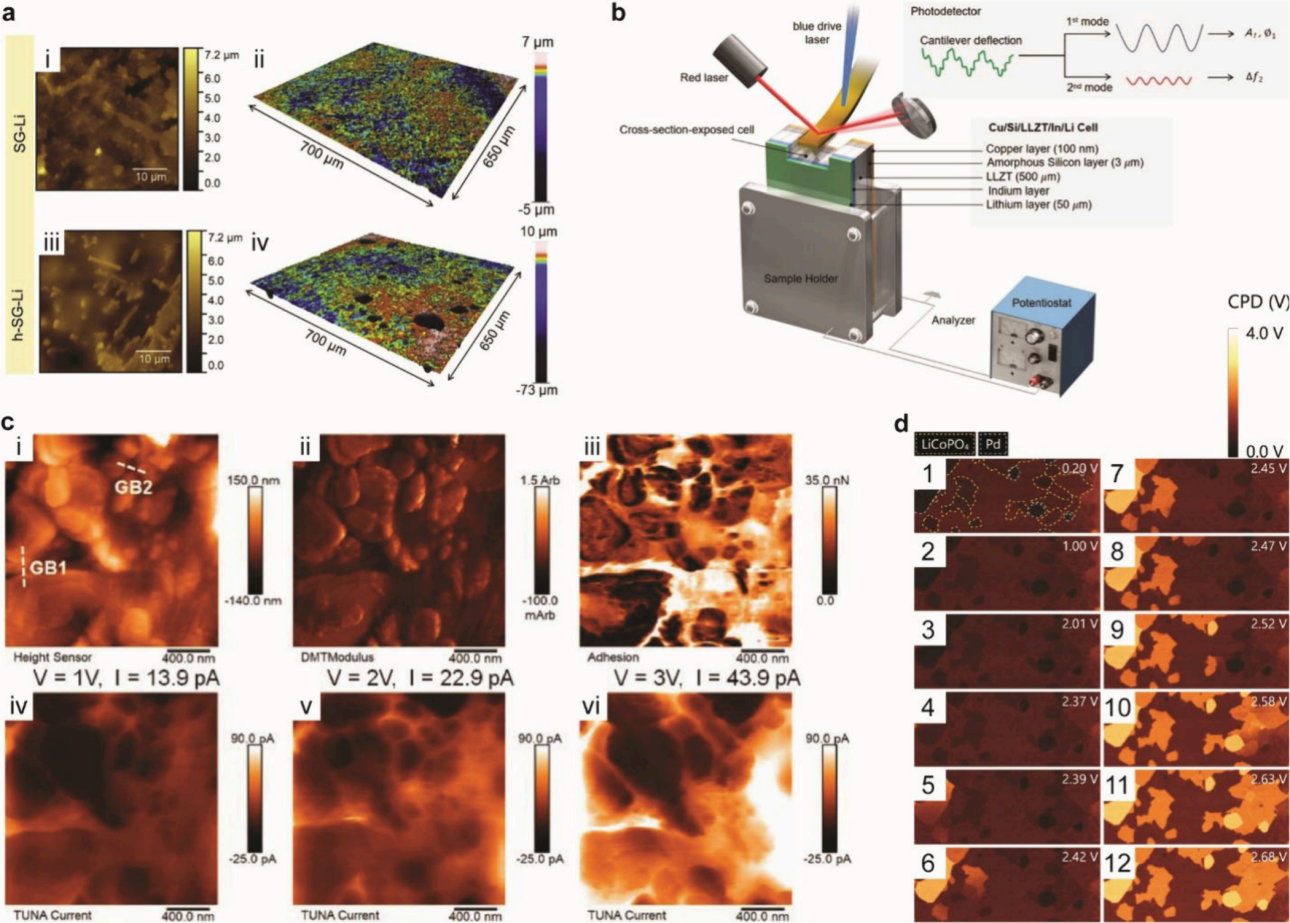
Examples of AFM characterization of SSB materials. (a) 2D AFM topographic maps (i and iii) and confocal microscopy images (ii and iv) for low (top) and high (bottom) dislocation density Li metal anodes after stripping against an LLZO SSE. Reproduced with permission from ref (456). Copyright 2023 Elsevier. (b) Diagram of an *in situ* AFM SSB housing allowing for the application of stack pressure with an exposed cross-section for AFM imaging. Reproduced with permission from ref (458). Copyright 2024 American Chemical Society. (c) Topography (i), Young’s modulus (ii), adhesion (iii), and conductive AFM current at 1 V (iv), 2 V (v), and 3 V (vi) for a 75% LLZO-PEO(LiClO_4_) SSE at 55 °C obtained through *in situ* AFM. Adapted with permission from ref (459). Copyright 2021 Elsevier. (d) *In situ* KPFM of an LCP/LATP/Pd solid-state composite cathode during charging (images 1–12). Adapted with permission from ref (460). Copyright 2019 Springer Nature.

The ability of AFM to observe the surface microstructure of a sample is an attractive feature for studying the deposition and stripping behavior of Li metal at the anode/SSE interface.^356,455−457^ A recent *ex situ* investigation used AFM to observe pores that formed at the Li–LLZO interface after stripping of a Li metal electrode (Figure 17a).^456^ Other *ex situ* studies have explored the effect of interlayers on Li deposition at Li–LLZO interfaces.^457^ While *in situ* AFM experiments in SSBs are largely limited to observing the cross-section of a cell as shown in Figure 17b (rather than a planar electrochemical interface), such experiments have been performed to observe the effect of Li growth in SSEs such as LLZO^455^ and gel polymer electrolytes.^356^

Because of its nanoscale spatial resolution, AFM has also been used to observe the evolution of the surface morphology of electrodes and SSE materials in addition to their electrochemical and mechanical properties (Figure 5). Several investigations of the surface structure of SSEs have been performed *ex situ* for polymeric electrolytes,^461,462^ oxides,^358,463,464^ and sulfides. One such study by Dixit et al. mapped out the adhesion of a PEO polymeric electrolyte surface in addition to its topography.^462^*In situ* AFM studies on the structure and properties of these materials typically utilize two different biasing modes. The first involves applying a bias with the cantilever tip to observe local changes in electrochemical properties.^465^ A more common method is to assemble a custom SSB cell housing that allows for complete electrochemical cycling of the cell and *in situ* observation.^376,377,458,459^ In addition to observing the effect of electrical biasing, other relevant environmental conditions have been explored, such as the effect of increasing temperature on an LLZO-PEO composite SSE. (Figure 17c).^459^ The resulting *in situ* AFM maps in Figure 17c material are representative of typical results.

Characterizing the surface topography and the relevant electrochemical and mechanical properties of interphases formed in SSB systems is also possible with AFM. The interphases of several SSEs have been investigated *ex situ*, including gel and polymeric electrolyte SEI,^361,466^ LAGP SSE SEI,^467^ and NMC-811/LLZTO CEI.^468^*Ex situ* AFM is particularly useful for comparing engineered interphases to the interphases naturally formed between SSEs and Li metal. *In situ* investigations of interphase formation with AFM have primarily focused on cathode-side CEI formation but have also been used to investigate SEI formation of a Li–In electrode in contact with LGPS electrolyte.^469^ A representative example was performed by Guo et al. on a coated NMC-523 cathode in contact with an interpenetrating network poly(ether-acrylate) SSE, wherein the formation of the CEI film was observed *in situ* with AFM during cyclic voltammetry.^470^

A variant of AFM used in SSB research is Kelvin probe force microscopy (KPFM),^460,471,472^ which operates without contacting the sample by creating a contact potential difference (CPD) between the cantilever and the sample. KPFM offers analytical capabilities for electronic properties of surfaces, which is useful for SSBs. Figure 17d highlights the work of Masuda et al., who used *in situ* KPFM to collect evolving CPD maps of a composite cathode consisting of LiCoPO_4_ (LCP) cathode material and LATP SSE.^460^ Using KPFM instead of traditional AFM allowed for analysis of the local electric potential in individual particles of the cathode composite during cycling.

Finally, other unconventional AFM and SPM techniques have been used in SSB research. Electrochemical strain microscopy (ESM) evaluates the local lattice strain induced by the electric field at the tip–sample junction; it has seen *ex situ*(473) and *in situ*(474) usage for both composite and thin-film solid-state electrodes to evaluate the ionic and electronic conduction behavior. Catarelli et al. used intermittent contact alternating current scanning electrochemical microscopy (ic-ac-SECM) to observe the AC response of LLZO SSE in contrast to the DC conditions imposed in most other AFM experiments in SSB research.^475^ Finally, time-resolved electrostatic force microscopy (trEFM) was utilized by Harrison et al. to observe the dynamics of Li^+^ ions in a polymeric SSE, further highlighting the unique and varied possibilities for AFM research in SSBs.^476^ A wide breadth of work has already been carried out with AFM, but there is more work to be done in designing *in situ* experiments that are representative of true electrochemical systems.

#### 4.3.9 Transmission Electron Microscopy (TEM)

Transmission electron microscopy (TEM) and its scanning form (STEM) are powerful imaging techniques used to understand the atomic-to-nanoscale structure and chemistry of materials. (S)TEM has the highest spatial resolution of any imaging technique discussed so far (down to hundreds of picometers), with the ability to resolve atomic lattice structures with high-resolution TEM (HR-TEM)^477^ or individual atomic columns with STEM (Figure 5). In general, (S)TEM involves transmission of a high-energy electron beam through a sample, followed by the detection of scattered or transmitted electrons. Although (S)TEM and SEM both employ an electron beam from a thermionic or field emission source, (S)TEM uses much higher accelerating voltages (hundreds of kV for (S)TEM compared to ∼10 kV for SEM). A higher accelerating voltage creates an electron beam with a shorter wavelength to resolve smaller features. Because of the transmission of electrons through the sample, both real-space and reciprocal-space (i.e., diffraction) images can be generated with (S)TEM to reveal information about a sample’s morphology, crystal structure, and crystallography.

The power and versatility of (S)TEM has made it one of the most used imaging techniques for characterizing battery materials.^478^*Ex situ* (S)TEM has been employed for many decades to characterize the crystal structure and structural changes with cycling of battery materials.^479^ In recent decades, a great deal of effort has gone into developing *in situ* (S)TEM experiments for characterizing the dynamic evolution of battery materials,^478^ such as intercalation electrodes,^480^ Li alloy anodes,^481^ conversion electrodes,^482^ Li metal,^483^ and Na-ion electrodes.^484^ Using *in situ* (S)TEM, the nano- and atomic-scale lithiation/sodiation reaction processes of these various electrode materials have been visualized. In recent years, related experiments have also provided important information about the fundamental electrochemical reaction mechanisms of SSB materials.^485^

Despite its utility, (S)TEM does feature some major limitations compared to other techniques. Samples must be extremely thin (ideally 100 nm or less) to allow for adequate transmission of the beam to the detector, which tends to limit how representative *in situ* experiments are of full-scale SSBs. Additionally, the high voltage beam (typically 80–300 kV) can cause damage to samples (especially battery materials) through either heating or knock-on effects.^486^ Transmission imaging is sometimes a source of difficulty as well, as it produces projection views of the 3D sample. While commercial (S)TEM instruments are widely available, the learning curve to become an expert in this technique is somewhat steeper than many other characterization methods. Despite these drawbacks, (S)TEM is a critically important imaging technique for SSB research.

There are a variety of operating modes and capabilities of (S)TEM,^486^ and we briefly describe and contrast these modes and note appropriate uses here. Conventional TEM is performed by transmitting a parallel beam of electrons through a sample. STEM focuses the beam of electrons onto a nanometric spot on the sample and rasters the beam, while collecting scattered electrons to create the image. Conventional TEM has three main imaging modes: amplitude contrast (bright field or BF), diffraction contrast (dark field or DF), and phase contrast (high-resolution TEM or HRTEM). Because of the convergent beam used in STEM, the transmitted beam requires ring-shaped detectors; thus, the term “annular” is used in its three imaging modes, annular bright field (ABF), annular dark field (ADF), and high-angle annular dark field (HAADF). Bright field imaging (BF and ABF) detects transmitted electrons and displays mass–thickness contrast in the resulting image. Dark field imaging (DF, ADF, and HAADF) detects electrons that have been scattered and is thus very sensitive to changes in a crystal lattice’s orientation due to amplitude variations from diffraction. HAADF imaging in STEM creates contrast by detecting electrons scattered at very high angles; because the intensity is related to the square of the atomic number of elements, HAADF is well-suited for providing contrast between elements with different atomic numbers. Phase-contrast imaging in TEM utilizes interference between the direct and scattered beams to create high-resolution images of crystal lattices.

In addition to imaging, the electron diffraction patterns arising from diffraction from a sample’s crystal lattice can be observed with selected-area electron diffraction (SAED) in TEM and convergent-beam electron diffraction (CBED) in STEM. These diffraction patterns are obtained by imaging the back focal plane of the objective lens; SAED creates an array of spots while CBED creates discs in the pattern owing to the different shapes of the electron beam in each technique. TEM and STEM can both be used to obtain chemical information about samples through EDS or electron energy loss spectroscopy (EELS) elemental analysis. However, only STEM can create spatially resolved elemental maps with these techniques due to its rastering operation. Conventional TEM is limited to obtaining spectra from the entire illuminated area, but this information can still provide important details about the elemental makeup of the region being imaged.

##### 4.3.9.1 *Ex Situ* (S)TEM Studies

**Figure 18:**
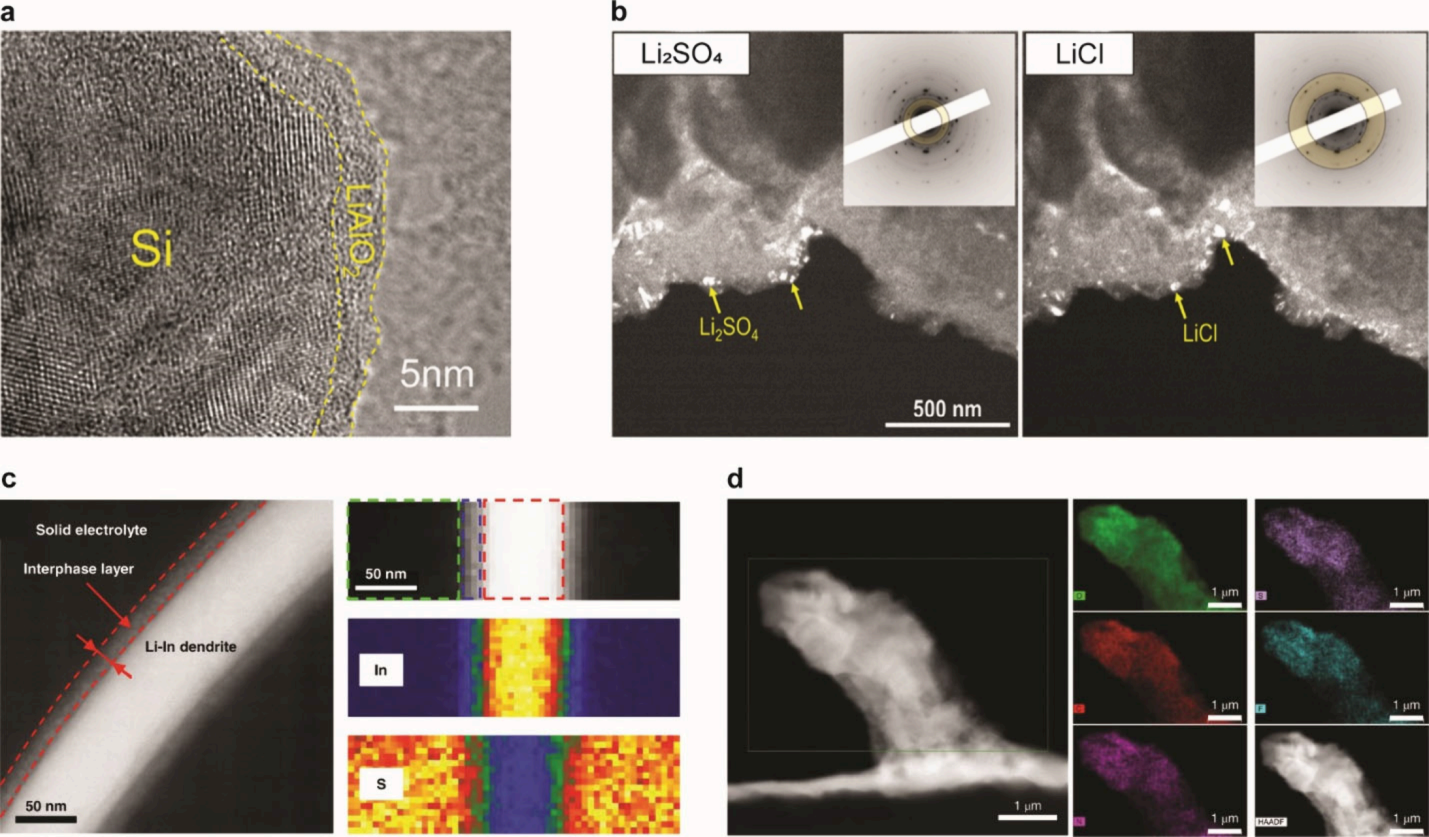
*Ex situ* (S)TEM imaging and analysis of SSB materials. (a) Bright field HR-TEM image of a Si nanoparticle coated with a protective layer of LiAlO_2_ for use in a SSB anode. Reproduced with permission from ref (491). Copyright 2023 Wiley-VCH GmbH. (b) Dark field TEM images and SAED patterns (insets) of (i) Li_2_SO_4_ and (ii) LiCl degradation components of LPSC SSE after air exposure. Reproduced with permission from ref (449). Copyright 2022 Elsevier. (c) HAADF-STEM images (left and top right) and EELS maps (center and bottom right) of a Li–In dendrite grown in LPSC SSE. Reproduced with permission from ref (494). Copyright 2021 Springer Nature. (d) Cryogenic STEM-HAADF image (left) and EDS elemental maps (right) of a Li dendrite grown in a solid polymer electrolyte. Reproduced with permission from ref (364). Copyright 2022 Springer Nature.

Here, we outline prior work using *ex situ* TEM to study SSBs, followed by discussing *in situ* investigations in the next section. The most common application of conventional TEM is to use bright field imaging to characterize SSB materials, typically in combination with HRTEM imaging and/or SAED. Examples of this are investigations of the crystal structure of SSEs,^487,488^ nanoscale protective coatings on active materials,^489−491^ and interphases formed between electrodes and the SSE.^492,493^ A recent example is Xu et al., who used HRTEM to observe the atomic-scale structure of a LiAlO_2_ coating on Si nanoparticles for use as an anode material in SSBs (Figure 18a).^491^ An example of dark-field TEM and SAED to enhance contrast between the degradation products of LPSC SSE when exposed to air is shown in Figure 18b.^449^

STEM can utilize the enhanced contrast between atoms of different atomic number using a HAADF detector; this has been particularly useful for SSB research in which multielement compounds are used as SSEs^495,496^ and cathode active materials.^497^ It is additionally useful for studying the makeup of the interphase at the cathode^498,499^ and the anode.^364,494,500^ The mapping capabilities of EDS and EELS in STEM can provide spatially resolved information at the nanoscale, such as in work that characterized the interphase layer formed between Li–In and LPSC SSE with STEM-HAADF imaging and EELS (Figure 18c).^494^ Holding the sample under cryogenic temperatures is useful for minimizing beam damage to sensitive materials like Li metal (Figure 18d).^364^

##### 4.3.9.2 *In Situ* (S)TEM Studies

*In situ* (S)TEM studies have been conducted on SSB materials to reveal dynamic transformation mechanisms. This section focuses on aspects to consider when carrying out *in situ* (S)TEM experiments on SSB materials.

**Figure 19:**
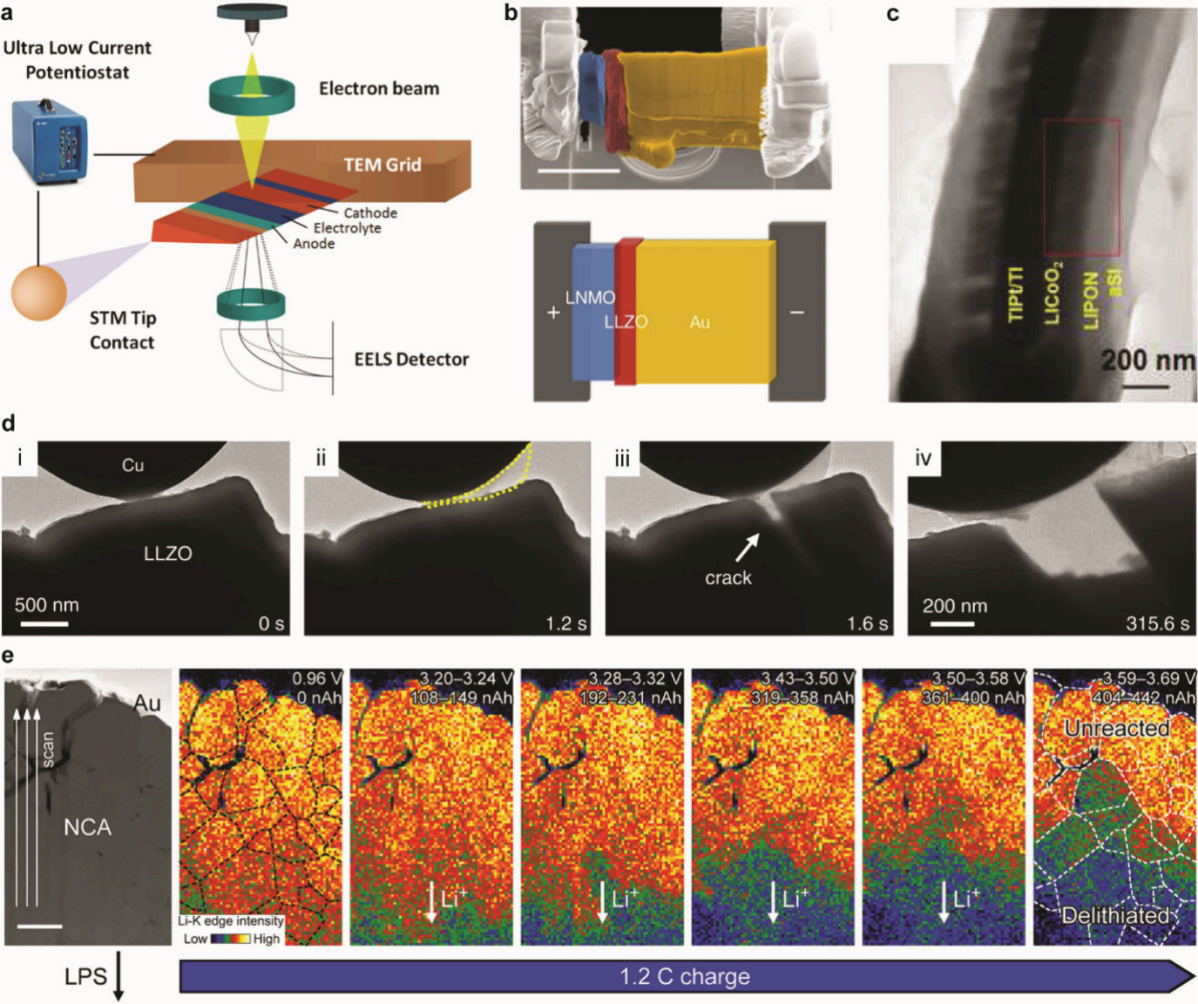
*In situ* (S)TEM imaging and analysis of SSBs. (a) Schematic of a FIB cross-sectioned SSB for *in situ* STEM imaging and analysis. Reproduced with permission from ref (504). Copyright 2016 American Chemical Society. (b) FIB image (top) and labeled schematic (bottom) of a MEMS chip with an FIB-cross-sectioned SSB for *in situ* STEM imaging and analysis. Scale bar is 5 μm. Adapted with permission from ref (505). Copyright 2018 Springer Nature. (c) *In situ* bright-field TEM image of a nanowire-based solid-state nanobattery (the red box indicates the LCO/LiPON interface). Reproduced with permission from ref (506). Copyright 2012 American Chemical Society. (d) *In situ* visualization of a Li dendrite growing between Cu foil and a single crystal of LLZO from the pristine state (i) to full dendritic growth (iv). Reproduced with permission from ref (507). Copyright 2022 Springer Nature. (e) ADF-STEM image (left) and EELS Li concentration maps (right) of an NCA solid-state cathode during delithiation. Scale bar is 500 nm. Reproduced with permission from ref (501). Copyright 2020 American Chemical Society.

One of the key challenges that *in situ* (S)TEM experiments face is fabricating cells that are thin enough to allow for electron beam transmission while allowing for (electro)chemical reactions to take place and remaining representative of larger-scale cells. Significant effort has gone into the design and operation of solid-state “nanobatteries” for *in situ* (S)TEM investigations. Many are fabricated by first creating a SSB through sequential thin-film deposition techniques (sputtering, pulsed laser deposition, thermal evaporation, etc.) and then using a FIB to create and mount a thin lamella of the battery stack, which can be biased within a TEM while imaging.^501−504^ A schematic example of such an experiment by Wang et al. is shown in Figure 19a for *in situ* STEM-EELS analysis on an LCO/LiPON/Si nanobattery.^504^ Other work has FIB-mounted solid-state nanobatteries onto micro-electro-mechanical systems (MEMS) chips for *in situ* testing (Figure 19b).^505^ An early method of solid-state nanobattery fabrication was reported by Ruzmetov et al., who created nanowire batteries by conformally depositing SSB materials onto metallized Si nanowires to create LCO/LiPON/Si SSBs with submicron diameters (Figure 19c).^506^ While none of these nanobatteries were operated with applied stack pressure, many were operated under appropriate electrochemical cycling conditions and displayed electrochemical activity. Other *in situ* investigations discussed below have been conducted not with full nanobatteries but instead by designing experiments to focus on a single phenomenon, such as interphase formation between the anode and electrolyte.^132^ These experiments avoid the lengthy and complicated assembly of nanobatteries and can still provide important information about nanoscale phenomena in SSBs.

A useful attribute of many (S)TEM instruments for *in situ* experiments is found in environmental capabilities that can heat specimens or introduce different gaseous environments near the specimen within the TEM column. Such experiments have been used to observe the structural evolution of various SSEs during heating^182,508^ and under exposure to air.^449,509^ Further *in situ* experiments of this kind could shed light on the manufacturing requirements of SSB material candidates in terms of moisture and heat exposure.

A key area of interest for *in situ* SSB (S)TEM research is the nanoscale behavior of Li metal at the anode/SSE interface.^507,510−512^ One experiment by Gao et al. captured the growth of a Li dendrite into a single crystal of LLZO SSE by embedding the LLZO crystal into Li foil and then contacting the crystal with a Cu probe to apply a bias (Figure 19d);^507^ this allowed for modeling of the GPa-levels of interfacial pressure that would be required for such an effect to occur. In addition to the growth of Li metal at this interface, its electrochemical stability and interphase formation has also been investigated by *in situ* (S)TEM. These types of experiments often utilize a specialized “nanoprobe” TEM stage with the capability to position a sample with nanoscale accuracy via piezo control while simultaneously applying a bias. Without the need for fabrication of a full nanobattery, SSEs can be brought into contact with Li metal under an applied bias to observe the formation of the interphase at the interface.^127,132,386^ Likewise, bringing electrode materials like Si or tin oxide into contact with Li or a SSE with Li behind it under the application of a bias can cause lithiation of the electrode materials, allowing for *in situ* observation of the lithiation pathways.^481,513,514^ These experimental techniques for SSB materials are based on earlier *in situ* TEM experiments to observe lithiation reactions of alloy anodes.^481,514−516^ As an example, LLZO was brought into contact with Li metal under bias to observe the formation of a nanoscopic interphase with STEM imaging.^132,517^

Using other analytical techniques in conjunction with *in situ* (S)TEM investigation can also be fruitful. The spatial mapping abilities of STEM-EELS and STEM-EDS analysis can create time-series maps of elemental distributions in SSB electrodes. Such an experiment was performed *in situ* by Nomura et al. on a fabricated nanobattery consisting of LPS SSE, NCA/LPS composite cathode, and an In counter electrode (Figure 19e).^501^ Electron holography (EH) has also been used for SSB research; this technique involves measuring the distribution of electromagnetic fields within a sample. *In situ* (S)TEM experiments have quantified the electric potential distribution within LCO cathodes in solid-state nanobatteries.^493^ These results have provided important information about the nonuniformity of Li reactions and the resulting electric potential distribution within LCO. Expanding the techniques discussed thus to materials including Si anodes, S and other conversion cathode materials, and alternative SSEs would be beneficial to the research community.

##### 4.3.9.3 Cryogenic (S)TEM Studies

Many battery materials are sensitive to electron beam damage in TEM due to their relatively high ionic conductivities and/or low melting points. Cryogenic sample stages can be used to cool samples to enhance the stability of these materials under electron beam illumination.^518,519^ Furthermore, cryogenic sample preparation can be used to preserve structures after removal from a cell housing, which can prevent extraneous side reactions with atmosphere and preserve their structure/chemistry. While cryogenic conditions are likely not suitable for *in situ* investigations due to the limited electrochemical reaction and diffusion kinetics under these low temperatures, these techniques are very useful for *ex situ* investigation. Cryo-(S)TEM has been employed in liquid electrolyte systems to observe the crystal structure and SEI makeup of Li dendrites^520−522^ as well as interphase on other electrode materials.^523−525^

In SSBs, cryo-(S)TEM has been used to observe interphase formation and dendrite crystallinity, to maintain the morphology of Li electrodes and dendrites during imaging,^362,364,494^ and to investigate interphase formation on other electrodes.^131,500^ A representative example using HAADF-STEM imaging is shown in Figure 18d, where the morphology and elemental composition of Li dendrites grown in a solid polymer electrolyte were investigated.^364^ There remains an opportunity for researchers to investigate cryogenically preserved interphases of various electrode/SSE combinations.

### 4.4 Spectroscopic Analysis

#### 4.4.1 X-ray Techniques

##### 4.4.1.1 X-ray Absorption Spectroscopy (XAS)

X-ray absorption spectroscopy (XAS) is used for investigating the local chemistry, coordination, and electronic structure of materials. It is an inner shell spectroscopy method in which incoming X-ray photons are absorbed to excite core electrons to higher energy levels above the Fermi level. The X-ray absorption coefficient in an element-specific energy range is of interest.^526^

When the energy of a core electron within an atom is equal to the incident photon energy, a sharp rise in the absorption (denoted as an “absorption edge”) is observed. This absorption increase occurs when the incident beam excites the core electrons of the absorbing atom to one of the unoccupied states in the valence shell. The location of the absorption edge is element-specific and scales approximately as the square of the atomic number (*Z*^2^).^526,527^ Since atomic numbers vary across the periodic table, the energy range of absorption for various elements is broad. X-rays are typically classified based on their energy range: hard X-rays (>6 keV) have a high penetration ability, tender X-rays (from 2 to 6 keV) are suitable for materials composed of low-Z elements, and soft X-rays (<2 keV) have a low penetrating ability.^528^

XAS is a measurement of the absorption coefficient of an element of interest in a sample, with three typical measurement modes. Transmission mode is similar to UV-absorption spectroscopy, and it measures the intensity of transmitted X-rays. The absorption length is a key factor that influences the measurement. Fluorescence mode measures the intensity of emitted X-rays due to X-ray fluorescence. Fluorescence mode has no dependence on the absorption length and therefore it can be more suitable for samples that do not feature appropriate thicknesses for transmission mode, as well as *in situ* and *operando* measurements. The total electron yield mode measures the flux of Auger electrons that escape from the material; this technique is highly surface sensitive. While most XAS is performed using synchrotron radiation because of the ability to control the monochromatic X-ray energy value, there has been recent improvement in lab-scale X-ray absorption spectrometers and they have been used for battery studies.^529,530^ However, these instruments typically employ X-ray tubes as a source which generate lower-flux X-ray emission over narrower energy ranges compared to synchrotron facilities.^531^ Therefore, the high flux and wider energy ranges of synchrotron-based XAS facilities are particularly useful for *in situ* and *operando* measurements.

**Figure 20:**
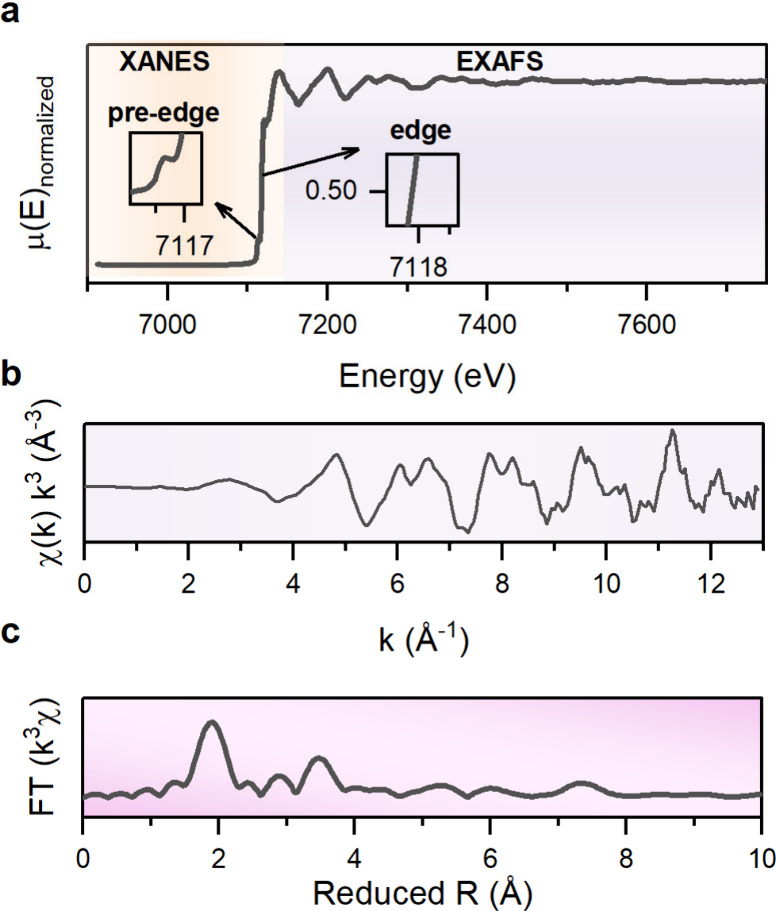
(a) Baseline subtracted and normalized Fe K-edge X-ray absorption spectrum of FeS_2_, as a typical example of XAS data. The spectrum is divided into two main regions: XANES and EXAFS. The insets highlight the pre-edge and edge regions. The location of the edge is determined by the half-height of the normalized spectrum. (b) Reduction of the Fe K-edge XAS spectrum in panel (a). The *x*-axis represents the wavevector (k) and the *y*-axis is the k^3^-weighted probability of absorption as a function of the photoelectron wavevector. (c) Fourier-transformed XAS spectrum in real space. The spectrum represents real-space distances and can be used for modeling coordination shells and extracting local coordination information such as bond distance and coordination number.

A typical XAS spectrum can be divided into two regions. These two regions reveal different details of the absorber atom, and they are complementary to each other. The first region, called the X-ray absorption near-edge structure or near-edge X-ray absorption fine structure (XANES or NEXAFS) region, comprises shoulders and peaks near the absorption edge (Figure 20a). XANES analysis gives information on the molecular structure, electronic structure, symmetry of coordination environments, and the oxidation state of the element. XANES includes electronic transitions from the ground state to localized states, and dipole-allowed transitions to localized states yield a prominent peak called the white line. Pre-edge features occur before the white line and are often useful in distinguishing symmetry and structural distortions (Figure 20a, inset). The second region is called the extended X-ray absorption fine structure (EXAFS) region that typically extends approximately 1 keV above the absorption edge (Figure 20a).^526^ EXAFS oscillations in this region result from the interaction between photoelectrons from the core level of the absorbing atom and the electrostatic potentials of the atoms near the absorbing atom. EXAFS gives information on the distance between absorbing atoms and their neighbors, coordination numbers, and disorder (Figure 20b,c).

For SSB research, XAS is a powerful bulk technique for the investigation of electronic structure and composition of electrodes, electrolytes, interfaces.^532−537^ For instance, transition metal oxides can undergo phase transformations during cycling, and XAS can be used to investigate atomic-level structural changes.^538^ Additionally, XAS provides detailed information about the electrode material chemistry, including the oxidation state of ions in transition metal oxides such as LCO and LFP, as well as the chemical properties of anode materials such as alloys.^539−542^

**Figure 21:**
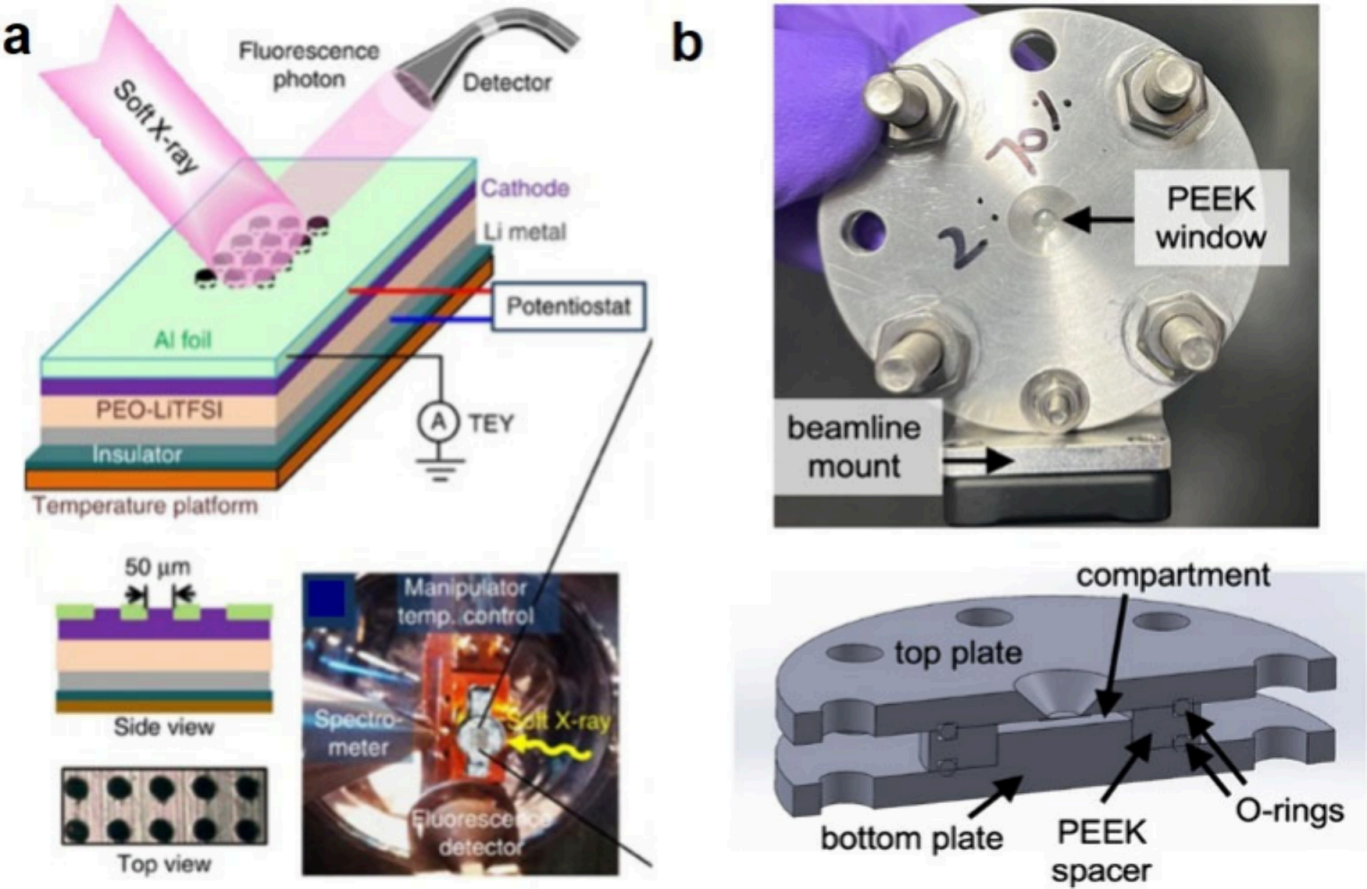
Considerations for *in situ/operando* XAS measurement cell design for SSB cells. (a) *In situ* cell design of cell with a Li anode, a polymer electrolyte, and an NMC or LFP composite cathode with an Al current collector containing holes. Adapted with permission from ref (538). Copyright 2013 Springer Nature. (b) *In situ* micro-XANES cell design with an X-ray transparent polyether ether ketone (PEEK) window. Reproduced with permission from ref (546). Copyright 2024 IOP Publishing Limited.

*In situ* and *operando* XAS techniques have been developed and used in SSBs to track chemistry, oxidation state, and local structure over time.^543^*In situ* and *operando* XAS experiments require the design of a suitable spectro-electrochemical cell. The cell requires a window with minimal X-ray absorption, appropriate sample thickness, and the ability to apply currents and stack pressures. Multiple cell designs have been used for *in situ* and *operando* XAS experiments (Figure 21). Liu et al. used Al current collectors with 50 μm diameter holes for transmission of X-rays to measure the Ni L-edge of NMC cathode material, as well as the Fe L-edge of LFP cathode material with polymer electrolyte (Figure 21a).^538^ Yamanaka et al. used a homemade cell for investigated a LiMn_2_O_4_ cathode using fluorescence mode.^544^ A few studies have used coin cells with a window covered by Mylar or Kapton,^536,545^ although these cells can be difficult to uniformly control stack pressure. Stavola et al. reported a cell with an X-ray transparent polyether ether ketone (PEEK) window that was able to accommodate a 50 MPa stack pressure during testing (Figure 21b).^546^ In another design, Cao et al. used an *operando* cell to track reactions in Si composite electrodes under 150 MPa applied pressure.^547^

**Figure 22:**
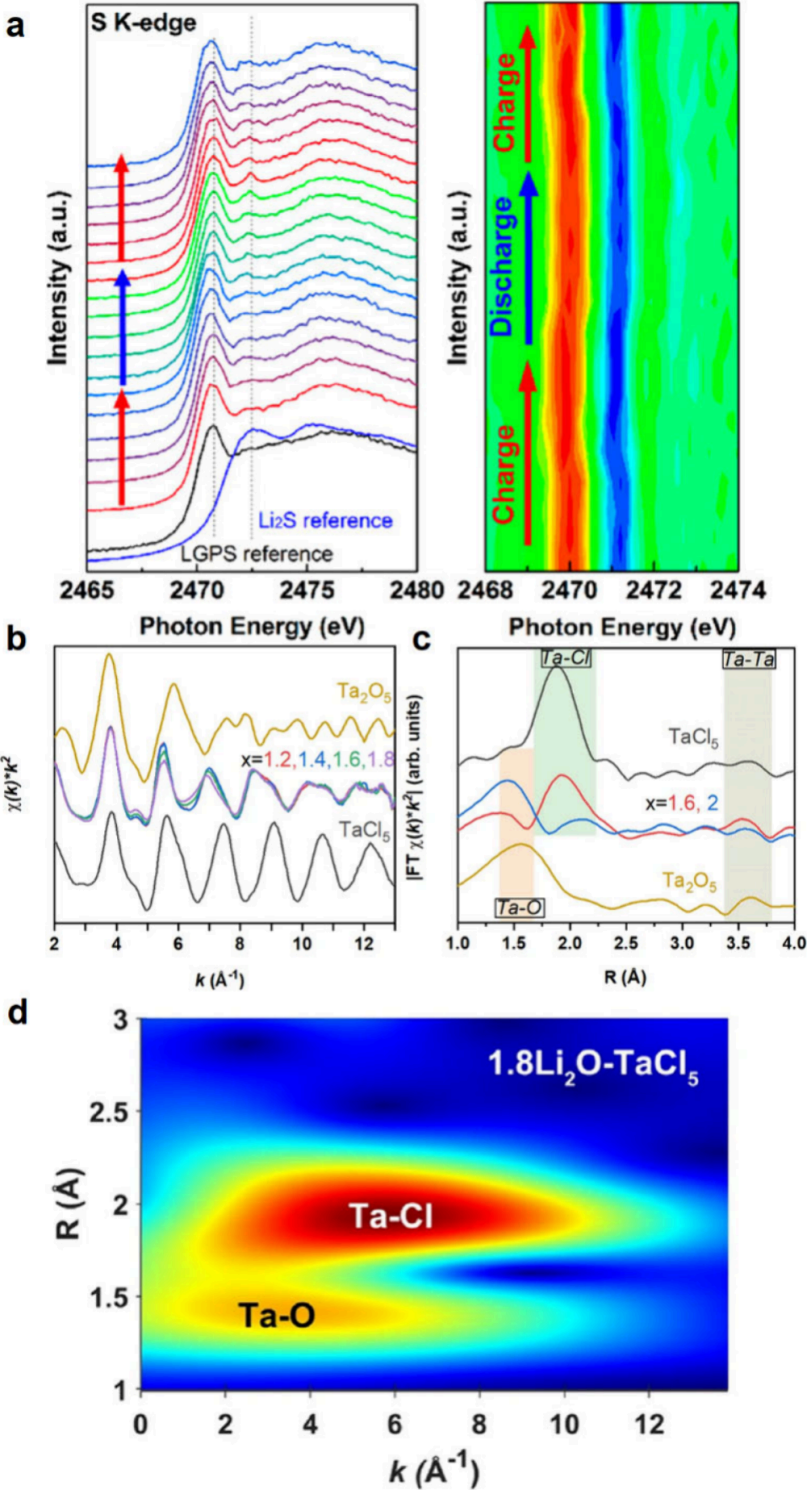
X-ray absorption spectroscopy studies. (a) *Operando* S K-edge and corresponding first derivative mapping. Reproduced with permission from ref (536). Copyright 2019 American Chemical Society. (b) k^2^-weighted Ta-L_3_ edge spectra. (c) Fourier-transformed Ta-L_3_ edge spectra of amorphous *x*Li_2_O-TaCl_5_ SSE, as well as Ta_2_O_5_ and TaCl_5_ precursors. (d) Wavelet spectra of 1.8Li_2_O-TaCl_5_. (b–d) Adapted with permission from ref (554). Copyright 2023 Springer Nature.

The XANES spectral region contains information about electronic and molecular properties such as oxidation state, symmetry, and structural distortions.^548,549^ Since transition metals or other ions in cathode materials undergo valence changes during Li insertion/extraction, XANES analysis is useful for investigating reaction mechanisms in cathode materials. Li et al. used *operando* XANES on a LiNi_0.8_Mn_0.1_Co_0.1_O_2_ (NMC811) cathode material to investigate chemical characteristics at the interface with LGPS (Figure 22a). The XANES spectrum of the Ni K-edge confirmed the electrochemical reaction at Ni sites, with the oxidation state increasing from Ni^2+^ to Ni^4+^. The LGPS stability was examined by measuring the S K-edge, with the results demonstrating decomposition of the SSE to form Li_2_S.^536^ To promote chemical stability of interfaces in cathode composites, cathode active materials can be coated with materials such as Li_4_Ti_5_O_12_ or LiNbO_3_.^550^ Morino et al. investigated the decomposition mechanism of a LiNbO_3_ coating on NMC532/LPSC with XAS. Based on the Nb K-edge spectra, the Nb species were preserved in the Nb^5+^ state. The Nb L_3_-edge revealed the release of O and Li in the charged state.^551^

The EXAFS region of an XAS spectrum provides information about the local coordination structure, such as the bond distance, the number of neighboring atoms, and local disorder. The crystallography of SSEs plays an important role in ion transport. Kwak et al. demonstrated that an SSE consisting of a nanocomposite ZrO_2_–2Li_2_ZrCl_5_F mixture exhibits improved compatibility within a LiIn-NMC cell. Zr K-edge spectra were collected to confirm the formation of the Zr–O bond due to the mechanochemical reaction between ZrCl_4_ and Li_2_O. EXAFS analysis showed that expansion of Cl-containing polyhedra due to the formation of smaller ZrO_2_ particles and longer Zr–Cl bond lengths could improve ion transport.^552^

Amorphous SSEs are uniform without grain boundaries, but in general they have somewhat lower ionic conductivity than various crystalline SSEs. Analysis of amorphous structures is challenging with traditional XRD, but XAS is a useful technique for understanding the local bonding environment and thus ion transport dynamics. Furthermore, the wavelet transform (WT) is used to differentiate neighbor-specific interactions in k and R space. In WT analysis, the wave is weighted in a particular part of k-space after the multiplication of the wave function by a Gaussian function. Therefore, the WT provides a description of the nature of each shell.^553^ Zhang et al. used EXAFS and wavelet transform (WT) analysis with a series of *x*Li_2_O-TaCl_5_ (*x* = 1.2, 1.4, 1.6, 1.8) SSEs. Based on Ta L_3_-edge EXAFS fitting, the coordination number was estimated to be five, which indicates the presence of Ta-centered trigonal bipyramidal structures. Furthermore, the formation of Ta–O bonds upon Li_2_O incorporation was found to be responsible for the amorphization of the SSE by bridging TaCl_5_ networks. Higher Li_2_O content leads to mixed short-range ordered structures of [TaCl_4_O]^−^ and [TaCl_5–1_O_*a*_]^*a*−^ and increased ionic conductivity (Figure 22b–d).^554^ EXAFS investigation also showed that introduction of fluoride in the structure induced bonding with Ta and Li and increased overall stability.^555^ The influence of carbon conductive additives on the valence of Ni in cathode composites after high-temperature processing has also been investigated.^556^

Although XAS is a bulk technique, spatially resolved information can be obtained through depth-resolved XAS. In this technique, the fluorescence signal is analyzed as a function of escape angle. Lower angles contain signals from species closer to the surface, while higher angles include signals from both the surface and bulk.^534^ Okumura et al. used this technique to investigate a thin film LiCoO_2_/Ni oxide/LATP stack with 7 nm thickness resolution.^534^ Chen et al. studied the effect of a Li_3_PO_4_ interlayer at a LiCoO_2_/80Li_2_S·20P_2_S_5_ interface before and after charging with depth-resolved XAS, observing that the interlayer suppressed the reduction of Co species in LCO.^533^ Tsai et al. investigated the interfacial reaction between LFP and LPSC SSE using nano-X-ray fluorescence and nano-XAS mapping. The mixed Fe(II) and Fe(III) valence of LFP indicated a decomposition reaction between these materials, with different Fe byproducts forming (including FeS and Li_2_FeP_2_O_7_).^541^

**Figure 23:**
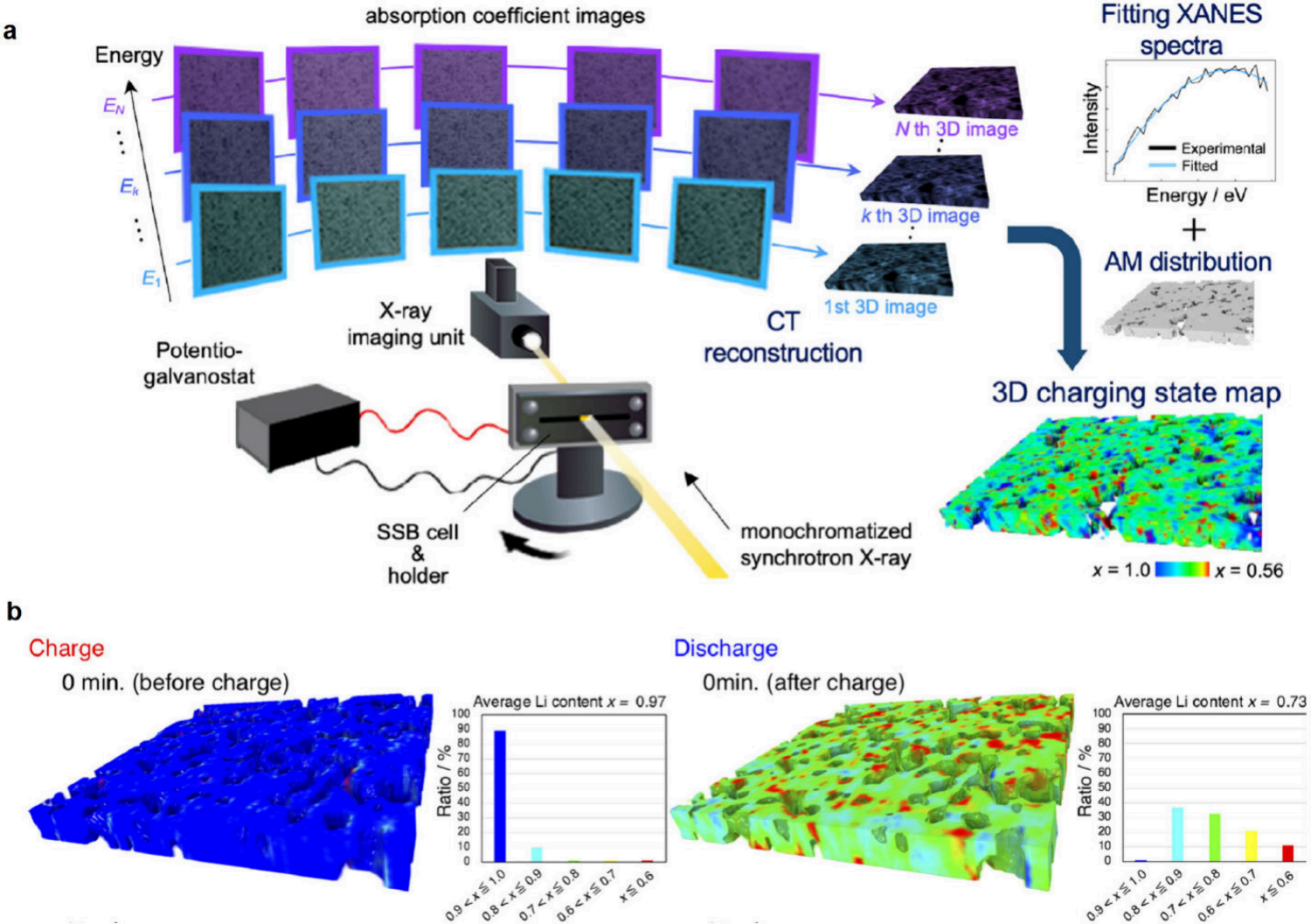
XANES mapping to elucidate oxidation state of the active material with respect to the state of charge. (a) *Operando* Co K-edge CT-XANES measurement and data analysis procedure. Reproduced with permission from ref (406). Copyright 2020 American Chemical Society. (b) CT-XANES measurement of a Li_*x*_CoO_2_ cathode to evaluate reaction homogeneity. Adapted with permission from ref (404). Copyright 2020 American Chemical Society.

XAS can be complementary to other techniques to enhance understanding of mesoscale reaction dynamics. *Operando* synchrotron imaging can be used with multidimensional 2D or 3D XANES techniques for micro- or nanoscale mapping of chemical features. Computed tomography CT-XANES has been used to investigate the local diversity of structural evolution within electrode architectures (Figure 23). In this technique, monochromatic X-rays irradiate the sample and transmission images are collected, which are then algorithmically combined into a 3D reconstruction.^405,406^ The working principle of *operando* CT-XANES is shown in Figure 23a.^406^ Kimura et al. used this technique with a polymer-based SSB system and observed inhomogeneous reaction processes and variations of Li charge depending on distance of the cathode active material from the SSE (Figure 23b).^404^ Wang and co-workers used 2D-transmission X-ray microscopy (TXM) and XANES to investigate Li-rich NMC, showing Mn ion activation and structural distortion when charged to potentials higher than 4.4 V in liquid electrolyte systems.^557^ Other work has used TXM-XAFS to evaluate the chemical state and degradation behavior of Fe_2_(MoO_4_)_3_ anode and LCO cathode pairs in thin film SSBs, showing nonuniform Li ion insertion and removal at the anode layer causing cracks within the electrode.^558^

In conclusion, XAS is a useful technique for SSB research for providing detailed electronic structure, oxidation state, and local atomic coordination information on electrodes and SSEs. The applicability of *in situ* and *operando* techniques and its compatibility with other techniques such as XCT allow for detailed analysis of bulk and interfacial properties.

##### 4.4.1.2 X-ray Photoelectron Spectroscopy (XPS)

X-ray photoelectron spectroscopy (XPS) is used to determine the chemical and electronic characteristics of elements within a sample, such as the oxidation state, ionization energies, surface composition, and valence band structures. In XPS, an X-ray beam is directed at a sample in a vacuum chamber, ejecting photoelectrons from the inner shells of atoms near the surface of the sample. The kinetic energies of the photoelectrons are measured to determine their characteristic binding energies, providing information about chemical composition and electronic structure. While the kinetic energy is independent of X-ray energy, the scale of binding energy and the peak positions of Auger electrons depend on the X-ray source. XPS is highly surface-sensitive, with a photoelectron depth sensitivity of around 10 nm. It can also be used in depth-profiling mode, in which a sample is sequentially sputtered in conjunction with gathering XPS spectra, which allows for investigation of properties within the depth of a material.

XPS has been used to investigate the chemical/electronic properties of surfaces and interfaces of SSBs. *Ex situ* XPS has been widely used to characterize the chemical properties and stability of anode, cathode, and SSE materials.^67,559^*Ex situ* experiments can be complicated by side reactions or other material evolution during sample preparation and transfer. To overcome these challenges, a variety of interesting approaches has been developed for *in situ* SSB investigations.

**Figure 24:**
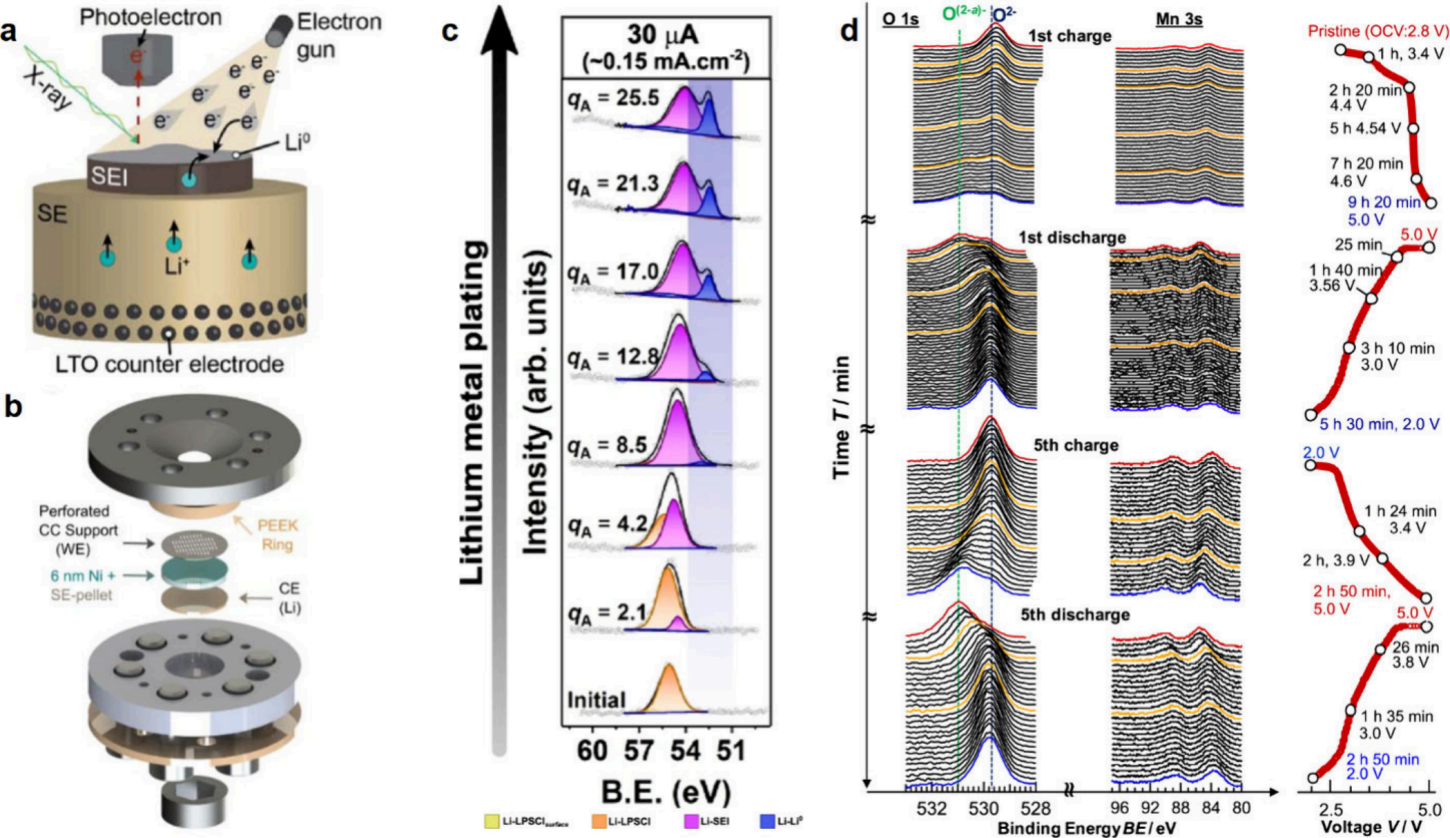
(a) *In situ* XPS cell where an electron gun is used as a virtual electrode. Reproduced with permission from ref (299). Copyright 2021 IOP Publishing Limited. (b) *In situ* cell design for a HAXPES experiment. Reproduced with permission from ref (566). Copyright 2024 American Chemical Society. (c) XPS measurement of Li 1s spectra to track interphase formation. Adapted with permission from ref (567). Copyright 2022 Springer Nature. (d) O 1s and Mn 3p HAXPES spectra of a Li_2_MnO_3_ cathode film. Reproduced with permission from ref (568). Copyright 2022 American Chemical Society.

In one *in situ* approach, an argon ion gun was used to sputter Li from a source onto the sample of interest.^560,561^ This allowed for the investigation of interphase formation behavior at the Li–LLTO interface by *in situ* lithiation, and the authors uncovered the formation of multivalent Ti species.^561^ Koerver et al. employed *in situ* XPS of the Li_3_PS_4_ SSE by probing a composite electrode containing the SSE and carbon from the backside in an XPS chamber, finding that the SSE decomposed to form redox-active species.^562^ Wood et al. developed a methodology to investigate chemical evolution at the Li–LPS interface through the use a “virtual electrode.” An electron gun was used to direct an electron flux to the SSE surface, which effectively caused a negative bias at the interface to cause thin Li to grow and form an interphase (Figure 24a). This study shed light on the formation of interphase components that affect the transport of Li ions.^563^ Davis et al. applied a similar methodology to demonstrate the effect of ALD-deposited Al_2_O_3_ interlayers on the formation of SEI at the Li|LGPS interface. Such interlayers suppressed SSE degradation, but the interlayer was lithiated during the initial charge.^564^ Other similar work has investigated interphase formation on LPSC and LGPS electrolytes in anode-free architectures.^299^

Although *in situ* XPS techniques provide detailed insights on interphase formation and the chemical composition of interfaces, the use of these technique often requires cell designs that are quite far removed from conventional cell housings. Effort has gone into bridging this gap: Wu et al. demonstrated an *in situ* XPS cell design that can accommodate stack pressure for a LCO|LPS|LiIn cell stack.^565^ The authors observed the formation of SSE decomposition byproducts at a potential above 2.1 V vs LiIn, which forms a passivation layer.

Synchrotron-based hard X-ray photoelectron spectroscopy (HAXPES) with higher photon energies provides penetration depths beyond 10 nm and can thus be used to investigate bulk properties and buried interfaces. Aktekin et al. employed HAXPES to reveal the decomposition of the LPSC SSE in an anode-free SSB cell (Figure 24b). The reduction of LPSC was observed to begin at a potential of 1.75 V vs Li/Li^+^.^566^ Narayanan et al. investigated interphase formation between Li and LPSC during plating using *in situ* HAXPES. Figure 24c shows the Li 1s core-level XPS spectra, where the broadening of the peaks was attributed to the formation of interfacial products confirmed to be a metallic Li layer.^567^

*In situ* XPS can also reveal useful information about cathode materials. Hikima et al. used *in situ* HAXPES to investigate reaction mechanisms in Li_2_MnO_3_ cathodes, revealing that the oxygen species take part in charge transfer during cell cycling (Figure 24d).^568^ The band structure of a thin film of Li_2_MnO_3_ has also been investigated using *in situ* HAXPES.^569^ Ultraviolet photoelectron spectroscopy (UPS) can be used to evaluate band structures and stability limits of SSEs. Kochetkov et al. used UPS to evaluate the oxidative stability of Li_2.5_Y_0.5_Zr_0.5_Cl_6_ and Li_2_Sc_1/3_In_1/3_Cl_4_ SSEs. Li_2_Sc_1/3_In_1/3_Cl_4_ was observed to have a higher valence band energy and is less susceptible to oxidation, while In^3+^ in Li_3_InCl_6_ SSE possesses higher electronegativity and a lower valence band energy.^570^ Overall, XPS is a useful technique to obtain information on the electronic and chemical structure of SSB materials, with opportunities for *in situ* and *ex situ* investigation.

##### 4.4.1.3 X-ray Diffraction (XRD)

X-ray diffraction (XRD) is a widespread technique in materials science used for analyzing crystal structures. XRD can provide information on crystallography, atomic spacing, grain orientation, strain, and defect character, among other features. Using XRD, the crystal structure of a given material can be determined by the diffraction peak locations and intensity through Rietveld refinement.

The operational mechanism of XRD usually involves directing an incident beam of monochromatic X-rays at a sample. X-rays that are scattered from the atoms within the sample experience constructive and destructive interference, with constructive interference resulting in outgoing diffracted X-ray radiation at particular angles. Through Bragg’s law, the angles of diffraction can be related to interplanar spacings within the crystal lattice of the sample. Multiple experimental geometries are used for diffraction pattern measurements. A common mode is the Bragg–Brentano geometry, which is a reflection geometry in which either the X-ray source is fixed and the sample and detector move by θ degrees and 2θ degrees, respectively, or the sample is fixed and the X-ray source and detector move at the same rate of θ degrees min^–1^. Another mode is the Debye–Scherrer geometry, in which samples are prepared in a capillary or thin film geometry for X-ray transmission. While sample preparation is more straightforward when using the Bragg–Brentano geometry, the use of a capillary in the Debye-Scherer geometry can be advantageous for air-sensitive materials. The peak intensity with the Debye–Scherrer geometry is weaker and more time is therefore required to collect XRD patterns.^571^

*Ex situ* XRD has frequently been used to investigate the crystal structure of a wide variety of battery materials. However, *ex situ* analysis of SSBs can be complicated by material instabilities or reactions with atmosphere.^572−574^ Therefore, *in situ* and *operando* XRD have also been widely used to track phase transformations in materials for Li-ion batteries and SSBs.^575−577^*Operando* XRD of SSBs requires an airtight cell assembly and an X-ray transmitting window material such as low-Z Be to ensure sufficient signal. Choudhary et al. introduced a cell design that is compatible with laboratory X-ray instruments, where the problem of bent Be or glassy carbon windows was overcome by using a smaller window with a 250 μm thickness.^578^ Other work has used Swagelok-type cell designs in SSBs to investigate crystal structure changes in electrode materials such as TiS_2_.^579^*In situ* and *operando* XRD is most readily performed using synchrotron radiation because of its advantageous capabilities of high flux to penetrate cells and to provide for fast scan times, as well as its tunable X-ray energy. However, laboratory X-ray instruments are also widely used for *in situ* XRD on battery materials.

An important use of XRD is extracting the crystallographic strain or volume changes through the measurement of lattice parameters.^580−582^ Koerver et al. demonstrated such measurements of various anode and cathode materials upon lithiation and delithiation.^171^ Dixit et al. used XRD to investigate the effect of phase heterogeneity in LLZO-type SSEs on chemo-mechanical behavior. High-energy XRD and far field high-energy diffraction microscopy revealed the formation of multiple cubic polymorphic phases.^401^

**Figure 25:**
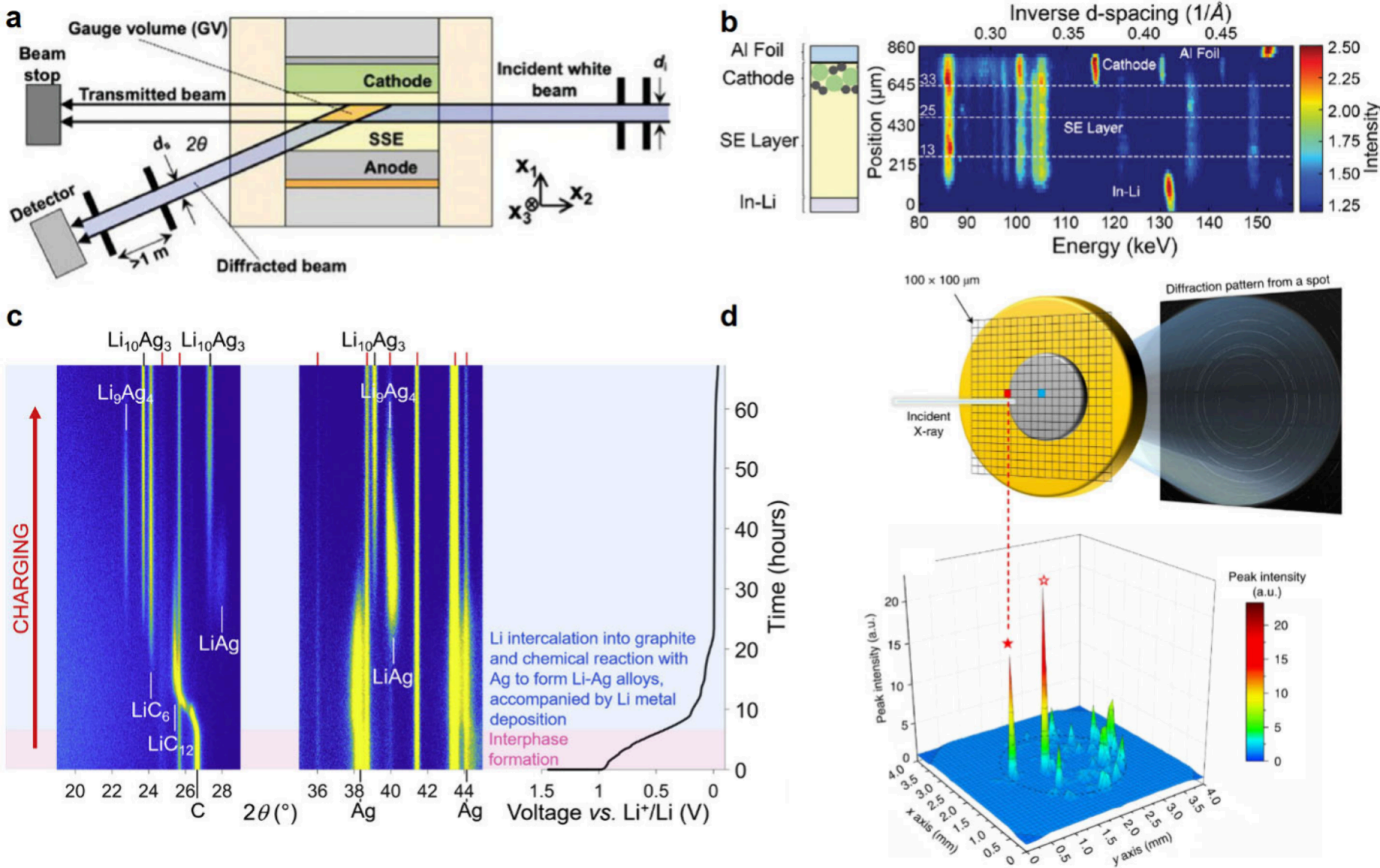
*Operando* XRD and ED-XRD studies on SSBs. (a) Schematic of the working principle of ED-XRD. (b) ED-XRD data from a SSB with a Li–In anode, FeS_2_ cathode, and Li_6.6_Ge_0.6_Sb_0.4_S_5_I SSE. (a, b) Adapted with permission from ref (583). Copyright 2021 Wiley-VCH GmbH. (c) *Operando* powder XRD pattern collected during lithiation of a Ag-graphite anode with LPSC electrolyte. Adapted with permission from ref (584). Copyright 2023 Elsevier. (d) XRD mapping of a symmetric cell with LPSC SSE. (upper panel). Diffraction intensity of the Li(110) plane that shows the spatial distribution of Li dendrites within the cell (lower panel). Adapted with permission from ref (168). Copyright 2021 Springer Nature.

Energy dispersive X-ray diffraction (ED-XRD) is a synchrotron-based technique that utilizes a high-flux, high-energy polychromatic X-ray beam that can penetrate battery cells (Figure 25a). Due to the polychromatic beam, diffraction patterns can be rapidly collected without changing the scattering angle, with spatial resolution of several tens of microns. The data can be collected continuously, which is useful for *operando* measurements.^583,585,586^ Jeong et al. used *operando* ED-XRD to investigate the reaction characteristics of NMC811 in SSBs by measuring the evolution of the (003), (101), and (104) planes. Lithiation was observed to begin at the SSE-cathode interface and spread toward the current collector.^587^ Another ED-XRD study examined the electrochemical compatibility of the Li_6.6_Ge_0.6_Sb_0.4_S_5_I SSE within FeS_2_ composite cathodes (Figure 25b).^583^ The results indicated that the SSE undergoes structural breakdown at potentials below 0.7 V vs LiIn, with increased *d*-spacing and a decrease in crystallinity. O*perando* ED-XRD has also been used to investigate anode evolution in SSBs. A Ag–C composite interlayer was investigated with *operando* ED-XRD, showing phase transitions from the LiAg to Li_9_Ag_4_ and Li_10_Ag_3_ phases as Li was deposited (Figure 25c).^584^ In another study, XRD mapping was conducted along with X-ray tomography imaging on a Li|LPSC|Li symmetric cell to gain insight into the spatial distribution of Li dendrites (Figure 25d).^168^ The diffraction map revealed that dendrites were more concentrated at the edges of the electrode rather than at the center.

##### 4.4.1.4 X-ray Pair Distribution Function (PDF)

**Figure 26:**
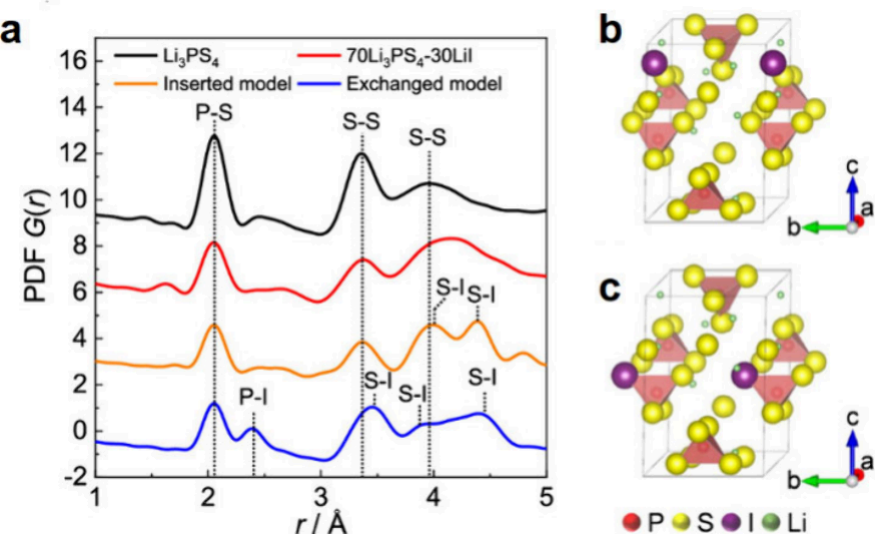
Pair distribution function analysis of the local coordination of LiI-doped Li_3_PS_4_. (a) Reduced PDF spectra of Li_3_PS_4_ (black) and 70Li_3_PS_4_–30LiI (red), along with simulated PDF spectra. (b) Inserted model (corresponding to the orange line in panel a). (c) Exchanged model (corresponding to the blue line in panel a indicates I^–^ is inserted between PS_4_^3–^ sites. Reproduced with permission from ref (593). Copyright 2021 American Chemical Society.

Pair distribution function (PDF) analysis is an X-ray (or neutron) scattering technique that can be used to determine both short- and long-range ordering in crystalline and amorphous materials. The PDF is the Fourier transform of the total scattering diffraction pattern (both Bragg scattering and diffuse scattering). Therefore, PDF analysis can provide local structural information on disordered, noncrystalline, or nanoscale materials, as well as information about long-range order.^588^ A typical PDF diffractogram (*G*(*r*) vs *r*) can provide deep structural insight (Figure 26a). *G*(*r*) is proportional to the number of atomic pairs weighted by the product of the scattering powers of the atoms, and *r* is the distance between pairs of atoms. The width of the peaks relates to static disorder and thermal motion. Quantitative analysis can be performed by fitting models to the data (e.g., small-box or large-box modeling).

A high-quality PDF measurement should be recorded to a relatively high *Q* range, where *Q* is the magnitude of the scattering vector, for optimal real space resolution. Therefore, short X-ray wavelengths and relatively large diffraction angles are required.^588^ Synchrotron facilities with high-flux X-ray sources (or centralized neutron facilities) can provide high *Q* measurements for X-ray PDF or neutron PDF analysis. Samples for *ex situ* analysis are generally loaded into borosilicate glass tubes or Kapton capillaries and sealed in a glovebox.

Although PDF can be applied to wide variety of materials,^589,590^ it is particularly useful in SSB research for determining the local atomic structure of SSEs.^34,591,592^ Takahashi et al. reported improved ionic conductivity of Li_3_PS_4_ with LiI doping, and PDF analysis showed that I^–^ was inserted between PS_4_^3–^ polyhedral (Figure 26).^593^ Schlem et al. investigated the effect of site disorder of rare-earth elements (e.g., Y and Er) in mechanochemically synthesized Li_3_MCl_6_ electrolytes on ionic conductivity.^594^ Dong et al. investigated the local structure of Ce-doped Li_5_La_3_Nb_2_O_12_ and found that there was a longer Ce–O bond distance compared to Nb–O at the octahedral sites.^595^

Although PDF analysis provides detailed insights into the local structure of SSB materials, this technique has largely been used for *ex situ* measurements. *In situ* and *operando* PDF measurements of SSBs with PDF are likely limited due to low resolution since high purity and thick samples are preferred, and other battery components can interfere with the quality of the results.^596,597^

#### 4.4.2 Neutron Techniques

##### 4.4.2.1 Neutron Diffraction

Neutron diffraction provides crystallographic information and is suitable for both single crystals and polycrystalline or powder samples. It can also provide magnetic structural details and superior contrast between some elements. Neutrons scatter from atomic nuclei and interact strongly with some elements that do not strongly scatter X-rays, such as Li. The scattering properties of an atom depend on the interaction between the nucleus (with its spin state) and neutron spin, which can lead to coherent or incoherent scattering. Coherent scattering is used in neutron diffraction, and Rietveld refinement can be used for structural analysis. Incoherent scattering occurs when the wavelength of the neutron changes. It does not contain structural information but adds background signal for analysis of the diffraction pattern.^597−600^ While neutron diffraction is a powerful technique, it is rarely used as the first choice to investigate the structure of materials, since a neutron source (either a nuclear reactor or a pulsed spallation source) is required.

Neutron diffraction is useful for studying complex structures and certain elements with low atomic numbers. The neutron scattering length does not have a consistent trend across the periodic table. Of relevance to SSB research, Li scatters neutrons relatively strongly and can show significant contrast in neutron powder diffraction, and the position of Li atoms in a crystal structure can be determined with neutron powder diffraction. This contrasts with the weak X-ray scattering of Li.

**Figure 27:**
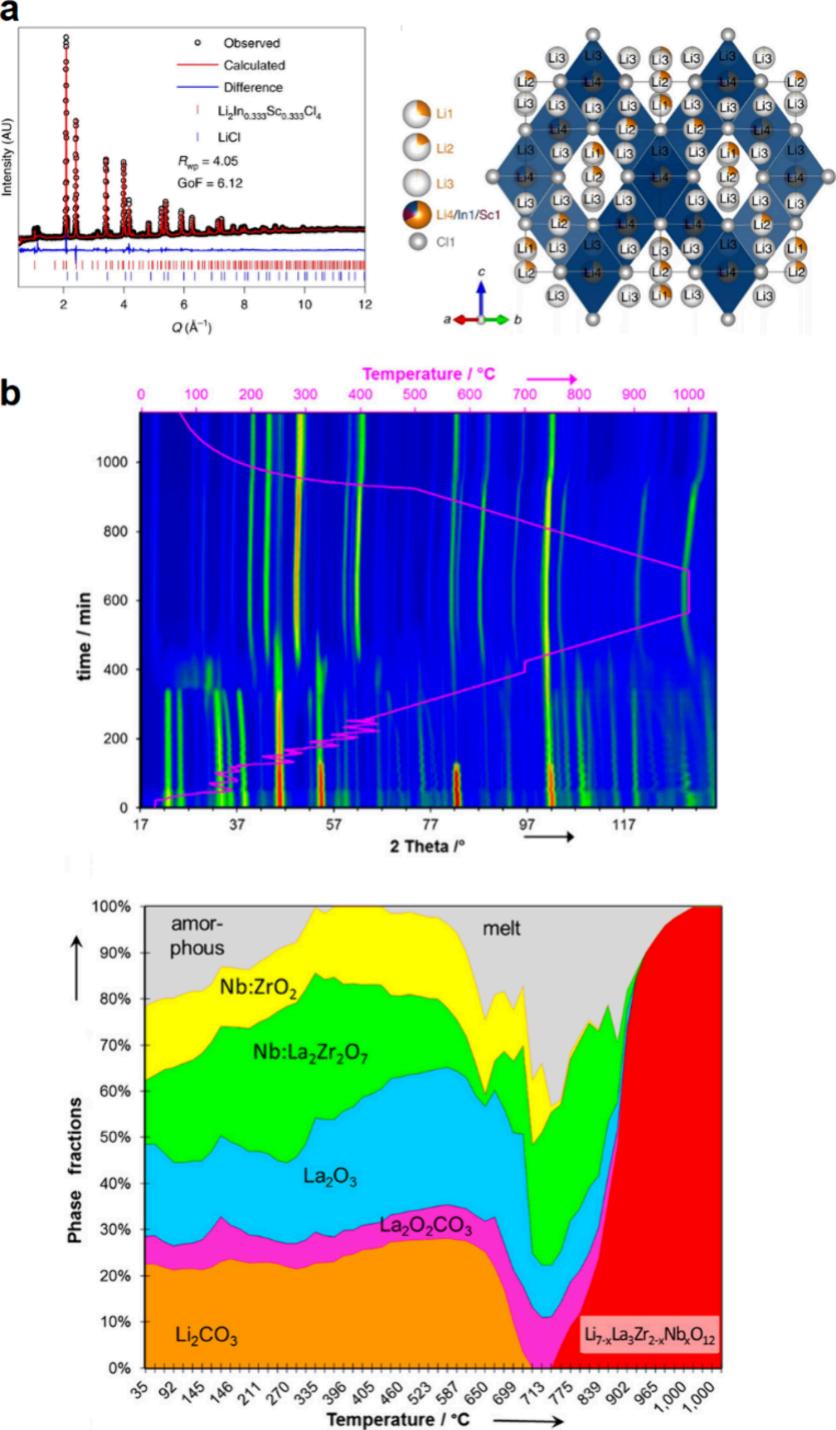
Neutron diffraction studies on various SSEs. (a) Time-of-flight neutron diffraction pattern (black) and Rietveld refinement (red) of Li_2_In_1/3_Sc_1/3_Cl_4_ SSE (left panel) and the obtained structure of the material from Rietveld refinement. Adapted with permission from ref (69). Copyright 2022 Springer Nature. (b) Temperature-dependent intensity map (upper panel) and mass fraction of the phases at different temperatures determined by Rietveld refinement (bottom panel) of the Li_6.75_La_3_Zr_1.75_Nb_0.25_O_12_ SSE. Reproduced with permission from ref (609). Copyright 2015 American Chemical Society.

For SSBs, neutron diffraction has been used to investigate the site occupancy and distribution of Li sites in SSEs, which is important because Li occupancy and distribution affects the ionic conductivity of SSEs. Zhou et al. reported a fast ionic conductor Li_2_In_1/3_Sc_1/3_Cl_4_ with an ionic conductivity of up to 2.0 mS cm^–1^.^69^ Neutron powder diffraction analysis revealed that although the SSE exhibited a similar structure as the halospinel Li_2_Sc_2/3_Cl_4_, the Li has a different site occupancy in the two materials, resulting in improved ionic conductivity in Li_2_In_1/3_Sc_1/3_Cl_4_ (Figure 27a). Neutron diffraction has also been used to investigate other SSEs, including argyrodites,^601−603^ garnets,^604,605^ and halides.^606−608^

*In situ* neutron diffraction has been used to investigate phase transitions of SSEs during synthesis, such as the crystallinity of LPSC during heat treatment.^603^ Similar experiments have been performed during heat treatment of Li_7_La_3_Zr_2_O_12_ and Li_6.75_La_3_Zr_1.75_Nb_0.25_O_12_ SSEs to determine phase fractions via Rietveld refinement (Figure 27b).^609^*In situ* or *operando* experiments have also been used to examine transformation behavior in cells (primarily for conventional Li-ion batteries). For instance, Taminato et al. used *operando* neutron diffraction to probe commercial liquid-electrolyte 18650 battery cells.^610^ The results provide valuable structural and kinetics information about the reactions that take place during cell operation. Use of similar techniques for SSBs, especially in conjunction with other diffraction or imaging techniques, could provide comprehensive information about electrode and SSE evolution.

##### 4.4.2.2 Neutron Scattering

Neutron scattering is a powerful technique for obtaining information about material structure. Elastic or inelastic scattering takes place when neutrons interact with elemental nuclei. During elastic scattering there is no energy exchange, whereas there is energy transfer in inelastic scattering. This technique offers several advantages for battery research. It is nondestructive and is therefore convenient for *in situ* and *operando* investigation. As in neutron diffraction, the interaction of elements with neutrons depends on atomic number (and isotope) with a different trend than X-rays, and neutrons are especially sensitive to some light elements.^600^ Because of the different neutron scatting lengths of different materials, it is possible to reveal the effect of incorporation of transition metals in different materials. There are various types of neutron scattering, as discussed below.

Small-angle neutron scattering (SANS), with a scattering length scale ranging between 1 and 1000 nm,^611^ is an elastic scattering technique that can be used to extract nanostructural features such as particle morphology, size distribution, and surface area of materials.^612,613^ Isotopic differences in scattering cross-sections (such as between H and deuterium) can provide contrast. Typical SANS spectra show the scattering intensity as a function of differences in scattered and incident wavevectors (i.e., the scattering vector Q in Å^–1^). The resolution of a SANS measurement is dependent on the neutron wavelength range.^597^

SANS can be used to understand phase transformation behavior of SSEs.^614^ For instance, SANS spectra of Li_1+*x*_Al_*x*_Ge_2–*x*_(PO_4_)_3_ glass–ceramics with Y_2_O_3_ added showed faster crystallization and improved ionic conductivity compared to those without Y_2_O_3_.^615^*Operando* SANS was applied to a SSB pouch cell with a diblock copolymer electrolyte to investigate the stability of the electrolyte during cycling.^616^ Yang et al. investigated Li filament growth in a Li|Li_6.5_La_3_Zr_1.5_Nb_0.5_O_12_|Cu cell using *operando* SANS. A Cu ring electrode was used to ensure neutron beam flux and to diminish the interfering Cu signals. Li had almost zero SANS signal since SANS is sensitive to inhomogeneities in the sample, but the LLZNO SSE had a strong SANS signal.^617^

Quasi-elastic neutron scattering (QENS) is a sensitive technique used to investigate atomic-scale ion dynamics.^618,619^ QENS measures the intensity of scattered neutrons as a function of momentum (*Q*) and energy transfer (ω). Spectra are plotted as energy transfer (μeV) versus scattering function S(*Q*,ω). The broadening of the elastic line indicates deviation from average atomic positions.

QENS is a suitable technique for materials with high ionic mobility, as it detects ion motion occurring on picosecond to nanosecond time scales.^600,619^ Understanding diffusion within SSEs is important for designing SSBs with fast charge/discharge capabilities.^620,621^ Altorfer et al. employed QENS to obtain mean residence time and diffusion pathways of Li between octahedral and tetrahedral sites in Li_2_S.^621^ QENS has also been used to measure self-diffusion coefficients, atomic hop distances, and residence time of Li ions within Li_7_P_3_S_11_.^620^ Other work on ion dynamics has focused on SSEs including Li_10_GeP_2_S_12_,^622^ garnets,^623,624^ lithium phosphate glasses,^625^ polymer SSEs,^626^ and Li_15_Si_4_.^627^*In situ/operando* experiments using QENS for SSBs is uncommon.

**Figure 28:**
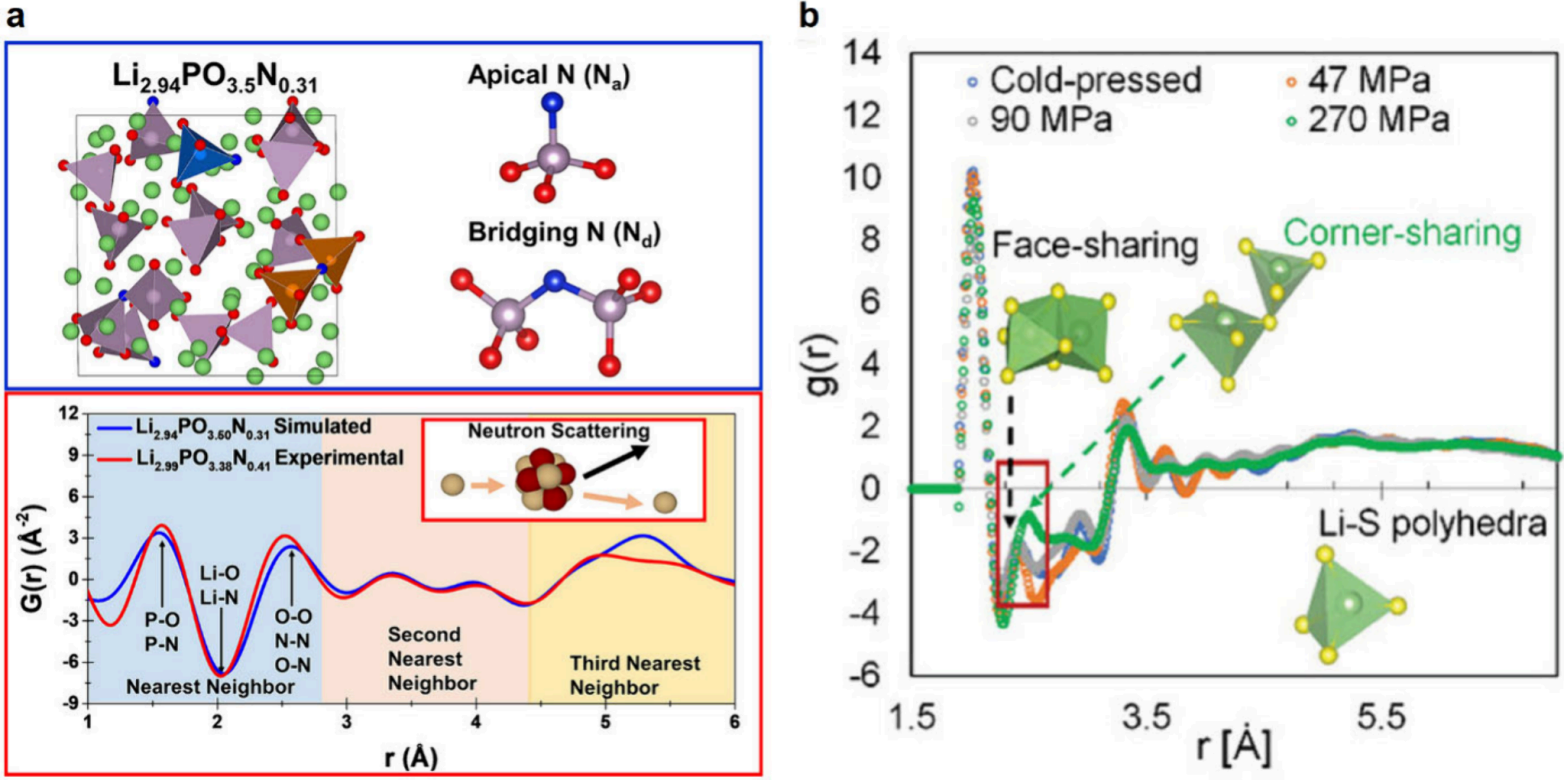
Neutron pair distribution function analysis of SSEs. (a) Schematic of simulated Li_2.94_PO_3.50_N_0.31_ structure (upper panel) and comparison of simulated and experimental neutron PDF data. Reproduced with permission from ref (628). Copyright 2018 American Chemical Society. (b) N-PDF analysis of Li_2_S–P_2_S_5_ samples processed at different molding pressures showing differences in local ordering. Reproduced with permission from ref (629). Copyright 2020 WILEY-VCH Verlag GmbH & Co. KGaA.

Neutron pair distribution function (N-PDF) analysis is similar to X-ray PDF analysis and is often used for studying the local atomic structure of materials. Although some lab-scale X-ray instruments have X-ray PDF capabilities, N-PDF is mostly accessible at synchrotron facilities. Figure 28a shows an N-PDF spectrum (bottom) of the LiPON SSE along with its structural model (top). The findings show a direct correlation of N sites to ionic conductivity.^628^ Garcia-Mendez et al. investigated the local structure of Li_2_S–P_2_S_5_ SSEs prepared with varying hot-pressing procedures (Figure 28b). A correlation between the local coordination of Li–S polyhedra and elastic constants was found.^629^

##### 4.4.2.3 Neutron Depth Profiling (NDP)

Neutron depth profiling (NDP) is a nondestructive technique that is sensitive to light elements that have isotopes, such as Li, Be, and H. The isotope undergoes a nuclear exoergic reaction with thermal neutrons and, depending on the isotope species, produces an α particle or proton and recoiling nucleus (e.g., ^1^n + ^6^Li → ^3^H + ^4^He).^630^ The charged particles have distinct kinetic energy, and some of the energy is emitted as they travel in the sample. The energy loss is then correlated to the spatial location and distribution of the elements as a function of depth within a sample. In solid-state batteries, NDP has been used to explore electrode–electrolyte interfaces and Li plating/stripping behavior.^631−634^

**Figure 29:**
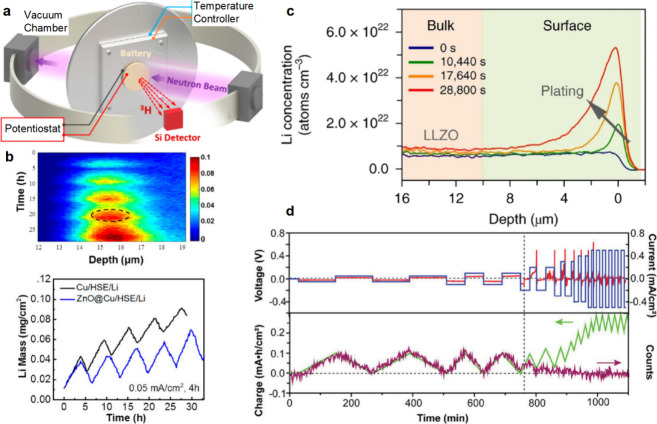
Neutron depth profiling studies. (a) Scheme of an NDP experiment. Adapted with permission from ref (634). Copyright 2017 American Chemical Society. (b) The effect of an interlayer on a Cu current collector in an anode-free cell. Li deposition without ZnO interlayer (upper panel). The effect of ZnO interlayer on Li density evolution (bottom panel). Adapted with permission from ref (635). Copyright 2020 American Chemical Society. (c) Time-resolved Li concentration plot for a Li–LLZO–Cu cell. Reproduced with permission from ref (636). Copyright 2019 Springer Nature. (d) Voltage profile and NPD curve to investigate short-circuiting behavior in garnet-based SSBs. Reproduced with a permission from ref (637). Copyright 2020 WILEY-VCH Verlag GmbH & Co. KGaA.

A schematic of an *in situ* NDP experiment on a SSB is shown in Figure 29a. The cell is attached to a temperature controller in a vacuum chamber. The incoming neutron beam interacts with the sample during Li plating/stripping, and a Si detector is used to detect ^3^H particles. In this work, the effects of carbon nanotubes as a Li metal host were investigated with a garnet SSE, and NDP identified dead Li sites.^634^ Other work has investigated Li plating in a Li|LLZTO|Ti cell with *in situ* NDP, showing that Li deposited into the void spaces of a 3D Ti electrode.^287^ Liu et al. explored the effect of a ZnO interlayer on a Cu current collector in an anode-free cell with an inorganic/organic hybrid SSE. The density of Li was reported to increase with the use of the interlayer (Figure 29b).^635^*In situ* observation of Li dendrite formation has been carried out using LiPON, LLZO, and Li_3_PS_4_ SSEs.^636^ Li tends to deposit into LLZO and Li_3_PS_4_ SSEs, and the authors suggested that electronic conductivity could cause internal Li growth within the SSE (Figure 29c).^636^ Other work has investigated the Li–garnet interface, providing information on Li transport and reversible short-circuiting behavior, which is caused by a conductive Li metal network that forms (Figure 29d).^637^ Chen and co-workers examined the degradation mechanisms of a microbattery based on an Li_3_PO_4_ SSE. The authors revealed the formation of an immobilized Li layer at the anode interface during initial charging.^633^ Shimizu et al. observed increased Li concentration at the LNMO|LiPON interface, which was attributed to overlithiation of LNMO.^638^ Overall, *in situ* and *operando* NPD can provide useful insight on various dynamic processes for a wide range of SSB materials, although the design of *operando* cells is challenging.^639^

#### 4.4.3 Vibrational Spectroscopy

##### 4.4.3.1 Raman Spectroscopy

Raman spectroscopy probes the vibrational modes of a material through phonon-induced excitation, with the resulting data giving information on the vibrational spectra of the material. Raman spectroscopy involves irradiating a material with a laser, with some photons undergoing elastic scattering (Rayleigh scattering) and others being scattered inelastically. These inelastically scattered photons induce a frequency shift that is equivalent to the vibrational frequency of molecular interactions. A Raman spectrum is the plot of Raman shift (the difference in Raman frequency from incident photons) vs intensity. Raman spectroscopy provides qualitative information on the structure of molecules or crystals based on the characteristic vibrational energy levels of interatomic bonds. Crystallinity, structural distortions, and the symmetry of a lattice can be investigated using Raman spectroscopy.

Raman spectroscopy is a convenient and highly sensitive technique for the investigation of the structure of materials. It is accessible in many laboratories, and a vacuum environment is not required. Spatial mapping can be used by scanning the laser with a microscale spot size to provide locally resolved information. Raman spectroscopy has been used for SSBs to probe the structure of electrodes and SSEs, as well as to investigate degradation mechanisms at interfaces.^640^ It can be used *ex situ* or *in situ/operando* for battery investigations. Limitations of Raman spectroscopy include its near-surface sensitivity due to the visible light probe, the relatively low intensity of Raman scattering, and the fact that not all materials are Raman active. Raman selection rules are determined by lattice vibrations, atomic structures, and phonon interactions. Raman active or inactive modes in crystal lattices are predicted by group theory, in which the lattice vibrations are classified according to the irreducible representation of point groups of the crystal lattices.^641^

**Figure 30:**
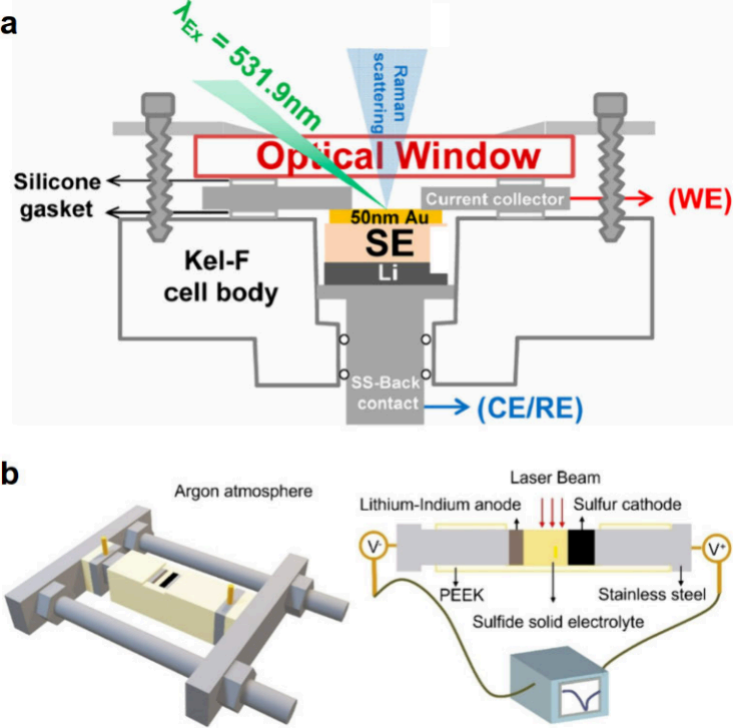
*In situ/operando* Raman spectroscopy experimental cell design. (a) Spectroelectrochemistry cell designed to investigate the interface between a Au film and an SSE. Reproduced with permission from ref (341). Copyright 2017 American Chemical Society. (b) Schematics of the SSB cell used to analyze a S–LPSC interface. Reproduced with permission from ref (644). Copyright 2023 Wiley-VCH GmbH.

*In situ* and *operando* cells for Raman spectroscopy of SSBs are similar to those for optical imaging. An example is shown in Figure 30a, where the optical window is made from quartz and silicone gaskets are used to maintain a sealed environment.^341^ Kuwata et al. prepared a similar cell for testing a thin film battery in which a Li anode, a Li_3_PO_4_ SSE, and a LiMn_2_O_4_ cathode were used.^642^ Other designs allow for investigation of SSB cross-sections,^643^ and designs have also been developed that allow for application of stack pressure (Figure 30b).^644^

**Figure 31:**
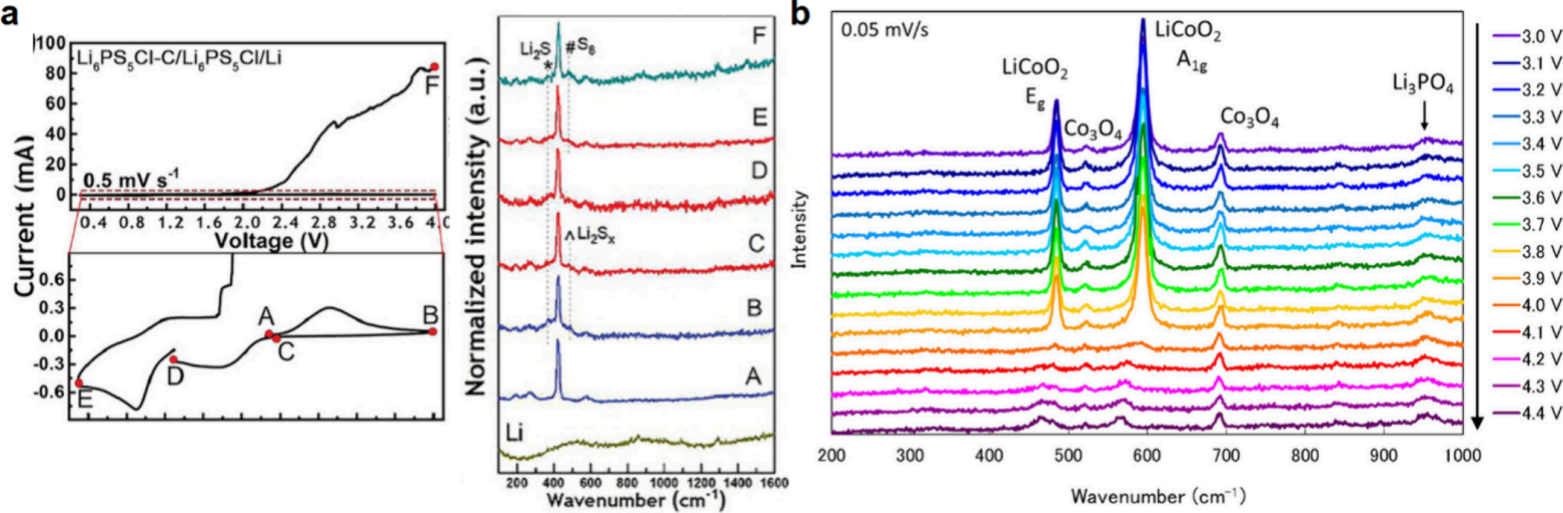
*In situ* Raman spectroscopy studies on anodes and cathodes in SSBs. (a) CV curves from a Li_6_PS_5_Cl-C|Li_6_PS_5_Cl|Li cell and corresponding *in situ* Raman spectra from the anode–SSE interface that show interfacial reactions between the anode and SSE. Adapted with permission from ref (648). Copyright 2021 Wiley-VCH GmbH. (b) *In situ* Raman spectra of LCO cathode during charging. Reproduced with permission from ref (650). Copyright 2019 Elsevier.

*In situ* or *operando* Raman spectroscopy can be used to evaluate the electrochemical stability of SSEs. Cao et al. investigated LPSC degradation and observed transformation into polysulfide and S species above 3.0 V vs Li/Li^+^.^490^ Mezzomo et al. used *in situ* Raman spectroscopy to demonstrate that a PEO-TiO_2_-LiTFSI electrolyte maintained its Raman peaks after cycling in a symmetric Li/Li cell, suggesting structural stability.^645^ Raman spectroscopy is not suitable for directly detecting Li metal signals due to its lattice symmetry.^640,646^ Therefore, Raman studies on SSB anodes studies have primarily investigated the Li–SSE interface or other types of anode materials such as graphite.^647^ For instance, Zhang et al. performed o*perando* Raman spectroscopy on graphite in a graphite|LPSC|LiCoO_2_ cell to investigate the microstructural evolution of the graphite by tracking the G-band.^647^ Zheng et al. performed *in situ* Raman spectroscopy with cyclic voltammetry on a Li|LPSC|LPSC-C cell to investigate side reactions between the Li anode and LPSC. The Raman spectrum showed the formation of Li_2_S, Li_2_S_*x*_ polysulfides, and sulfur species during potential sweeping (Figure 31a).^648^ Zeng et al. explored the effect of Cl^–^ distribution in LPSC on side reactions at the interface, observing the formation of P_2_S_5_ and Li_2_S_*x*_ species at potentials above 3.6 V vs Li/Li^+^ for Li_6.4_PS_5.4_Cl_0.6._ In contrast, no decomposition products were observed with Li_5.7_PS_4.7_Cl_1.3_.^649^

Raman spectroscopy is also useful for observing structural evolution of cathode active materials during cell cycling. Matsuda et al. used *in situ* Raman spectroscopy on a LiCoO_2_|Li_3_PO_4_|Li cell to investigate the structural transformation of LCO upon Li insertion and extraction, tracking changes in the lattice parameter. The authors reported the formation of inactive Co_3_O_4_ spinel during charging (Figure 31b).^650^ Zhou et al. investigated the evolution of the LCO–LPSC interface and observed the formation of S and P_2_S_*x*_ species during charging and the formation of Li_2_S and CuS after deposition of Li on a Cu current collector surface.^651^ Cao et al. studied the conversion reaction mechanism of a sulfur cathode with *operando* Raman spectroscopy.^644^ S offers high capacity, and the SSB environment can prevent the redox shuttling that occurs in liquid cells.^652^ The *operando* Raman spectroscopy results revealed that the main Raman peaks of sulfur do not completely disappear after the initial discharge, suggesting that S was not thoroughly lithiated. A transient Li_2_S_2_ species was detected during partial charge, converting to sulfur after complete charge.^644^*In situ* Raman spectroscopy has also been used to understand the conversion reaction mechanism of FeS_2_. The results showed that the material has a complex reaction mechanism, with the formation of Fe_2_S_3_ and FeS species observed as byproducts. Inactive Li_2_S accumulated over 50 cycles, causing capacity fade.^653^

**Figure 32:**
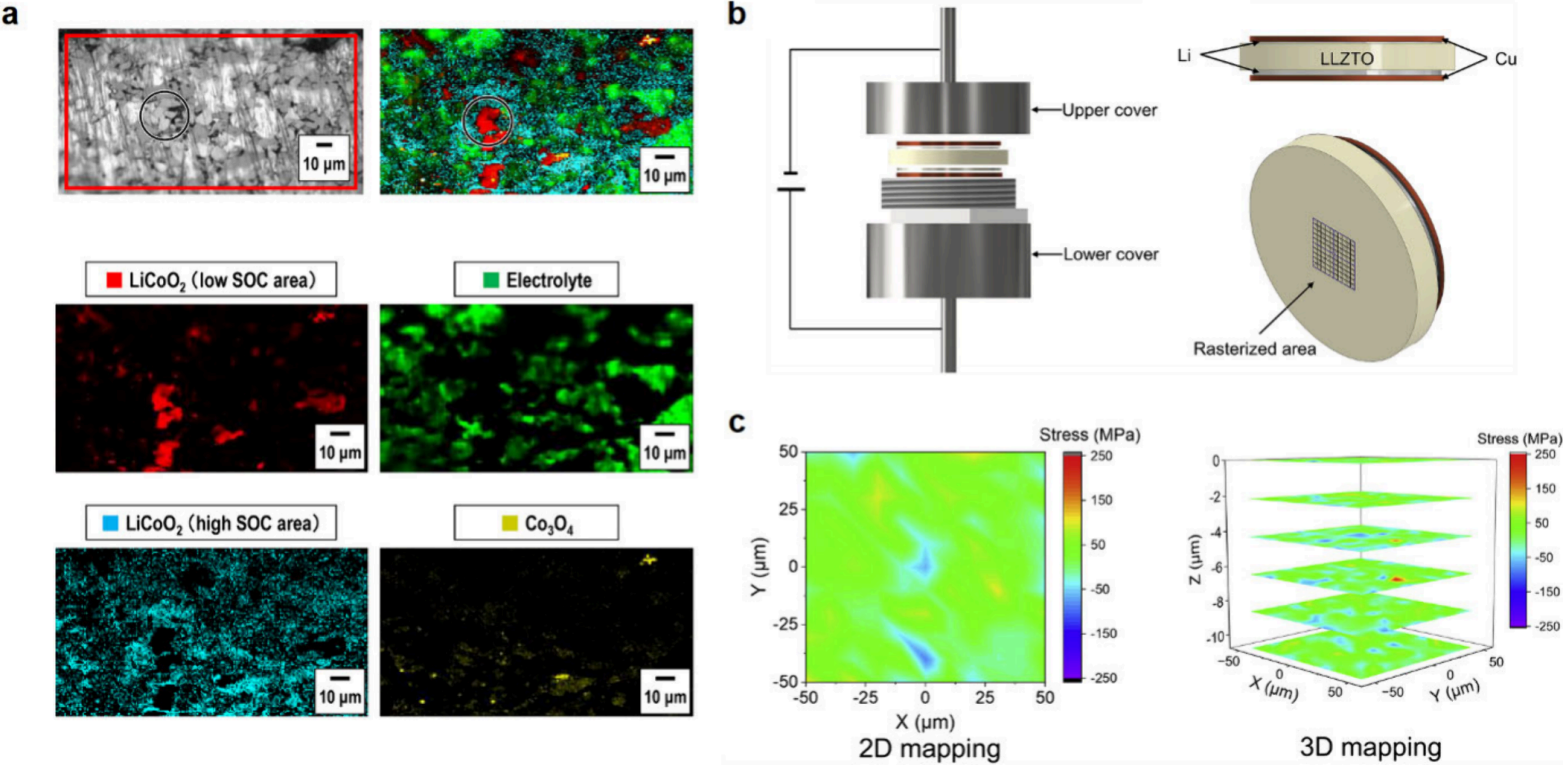
Applications of Raman mapping. (a) Optical and Raman imaging of a LiCoO_2_ composite and Raman mapping of active material, SSE, and Co_3_O_4_. Reproduced with permission from ref (643). Copyright 2016 Elsevier. (b) A Swagelok cell used for Raman mapping of a Li symmetric cell with LLZTO SSE. (c) 2D (left) and 3D (right) stress mapping analysis from Raman data. (b, c) Reproduced with permission from ref (654). Copyright 2022 Elsevier.

Raman mapping involves gathering Raman spectra using a rastered laser beam with micron-scale spot size, enabling locally resolved information to be obtained. It is particularly useful to discern different components of a SSB composite electrode or SSE. Otayama et al. used Raman mapping along with optical microscopy to investigate a LiCoO_2_ cathode composite before and after charging, observing the distribution of active material, SSE, and the formation of Co_3_O_4_ as a byproduct (Figure 32a).^643^ Confocal Raman mapping has been used to investigate microscopic strain evolution in a Li_6.4_La_3_Zr_1.4_Ta_0.6_O_12_ SSE by mapping lattice parameter changes through Raman spectroscopy (Figure 32b,c).^654^ Although generally not as sensitive to lattice parameter changes as XRD, the impact of stresses and strains on vibrational modes in a material makes Raman spectroscopy suitable for investigating chemo-mechanical aspects of SSBs.

In summary, Raman spectroscopy offers a versatile and rapid technique for investigating the structure, composition, phase distribution, and strain of SSB materials, without the need for large-scale user facilities such as synchrotrons or neutron sources. Furthermore, o*perando* or *in situ* cell design is straightforward, and Raman mapping can be performed in conjunction with optical microscopy. There is thus good future potential for the use of Raman spectroscopy in SSB research.

##### 4.4.3.2 Fourier Transform Infrared Spectroscopy (FTIR)

Fourier transform infrared (FTIR) spectroscopy is a vibrational technique that is used to investigate chemical and structural characteristics of materials and molecules. It is a nondestructive technique that is commonly employed for organic and polymeric materials to determine the nature of functional groups, for fingerprinting of an unknown substance, or for stereochemistry investigation. The infrared region in the electromagnetic spectrum is divided into three domains: high-energy near-infrared (14,000–4000 cm^–1^) for overtone and harmonic oscillations, mid-infrared (4000–400 cm^–1^) for vibrations, and low energy far-infrared (400–50 cm^–1^) for rotational spectroscopy. IR-active vibrations cause changes in dipole moments.

In this technique, infrared radiation is directed at a sample, and some radiation is absorbed and transmitted by the sample. The FTIR spectrum represents absorbance or percent transmittance of the vibrational modes related to molecular bonding environments. Therefore, it can be used to identify unknown molecules, changes in molecular structures, or composition.^655^ Considering speed, accuracy, cost, sensitivity, and resolution, FTIR is an efficient and precise technique for characterizing samples.

In the battery field, FTIR spectroscopy has mainly been used to investigate electrode and electrolyte decomposition^656^ and SEI formation.^657^ For SSBs, it has been used *ex situ* for quantitatively analyzing surface carbonaceous species on NMC cathodes,^658^ probing interactions of polymer SSEs with a LLZO SSE,^659,660^ investigating SSE moisture sensitivity,^661^ probing the stability of SSEs,^662^ and analyzing properties of cathode coating layers.^663^*In situ* and *operando* FTIR spectroscopy techniques have been developed for a variety of battery applications, including Li-ion,^657^ Li–S,^664^ Li–air,^665^ and others. However, the use of *in situ* FTIR spectroscopy for SSBs is quite limited compared to other battery architectures. The better suitability of FTIR spectroscopy for organic or polymeric materials limits usage of the technique for inorganic materials, such as sulfide and oxide-based SSEs.

#### 4.4.4 Magnetism-Based Techniques

##### 4.4.4.1 Nuclear Magnetic Resonance (NMR)

A nuclear magnetic resonance (NMR) signal arises from the interaction of nuclei with an external magnetic field in the radio frequency range. This interaction reveals atomic-scale structural and electronic properties in the sample. NMR spectroscopy is a powerful technique, especially for investigating the local chemical structure of materials. This section will discuss the basics of NMR spectroscopy, various approaches for using NMR spectroscopy for SSBs, *in situ/operando* measurements on SSBs, and their outcomes with examples from previous studies. For further information, readers are directed to other excellent reviews on NMR spectroscopy for various battery applications.^666−669^

NMR spectroscopy gives insight into chemical structure from chemical shifts, couplings, and the relative intensity of the resonance information in an NMR spectrum. Structural information is obtained from manipulation of spin systems and averaging or removing some of the solid-state interactions. Depending on the orientation of a nuclear spin vector with respect to the magnetic field, there are distinct interactions that contribute to spectral broadening. The most important interactions are as follows. i) The *chemical shift interaction* results from the modification of the applied magnetic field on the nucleus by the electrons surrounding the nucleus. It provides information regarding the local structure that surrounds the nucleus. ii) The *Zeeman interaction is* caused by the interaction between an applied static magnetic field (H_0_) with the magnetic moment of a nucleus (μ_n_). This interaction leads to separation of energy levels of the nucleus. iii) The *dipolar interaction* is observed due to the interaction between two like or unlike spins which cause spectral broadening. It provides insights into interatomic distances and crystal structures. iv) The *quadrupolar interactions* are caused by the interaction between nuclear spin with I > 1/2 (i.e., quadrupolar) nuclei and the electric field gradient at the nucleus. This type of interaction occurs for atoms or ions that do not exhibit cubic symmetry. Therefore, local structural distortion information can be obtained.^669−672^

##### 4.4.4.1.1 Solid-State NMR (ss-NMR)

Solid-state NMR is a useful technique particularly for materials that lack long-range order, which reduces the utility of traditional XRD techniques. Ionic or atomic diffusion properties and local structure (such as site occupancy) can be investigated with ss-NMR.^669,673^ Several methods have been developed for ss-NMR spectroscopy, such as pulsed field gradient (PFG) NMR, magic angle spinning (MAS) NMR, two-dimensional exchange spectroscopy (2D EXSY) NMR, NMR relaxation time measurements, and magnetic resonance imaging (MRI) techniques.^619,673−675^ Li diffusion characteristics can be directly measured with NMR techniques, and this capability can therefore provide valuable information that is complementary to electrochemical measurements.

As an example of ss-NMR usage, Zheng et al. reported the effect of mechanical energy on the phase stability of Li_10_SnP_2_S_12_ electrolytes treated with hand-grinding and different ball-milling rates.^676^ Sulfide SSEs offer high ionic conductivity, but their stability window is narrow compared to oxide and halide-based SSEs. According to ^31^P NMR, Li_3_PS_4_ species were observed at 84 ppm, and ^119^Sn NMR confirmed the formation of Li_4_SnS_4_ species at ∼50 ppm. The chemical decomposition led to a decrease in ionic conductivity.^676^ Li metal exhibits paramagnetic behavior since it has an unpaired electron, while Li ions at the interface or in an SSE exhibit diamagnetic behavior; therefore, the resonance frequencies of these Li species differ from each other. This difference enables differentiation via NMR to monitor structural changes.^677^

##### 4.4.4.1.2 Magic Angle Spinning (MAS)

**Figure 33:**
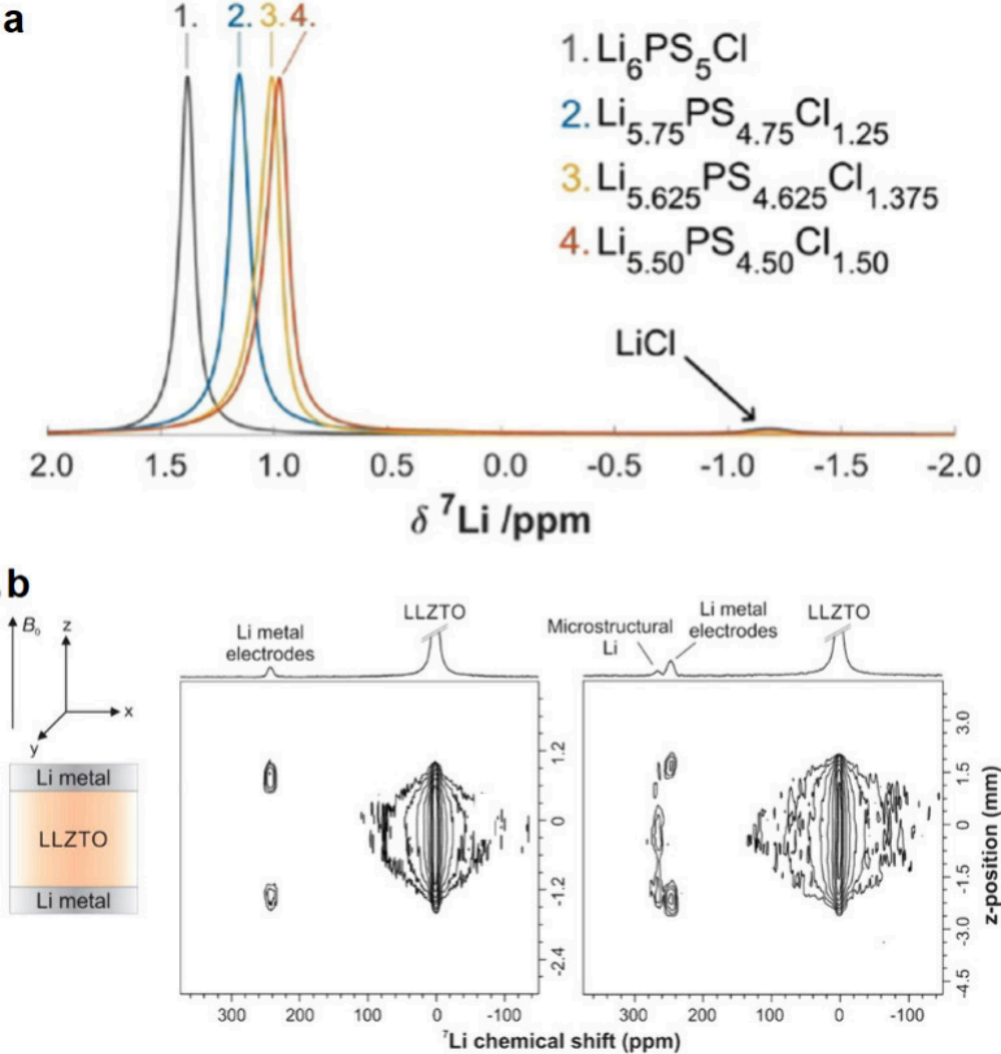
Solid-state NMR approaches. (a) ^7^Li MAS NMR spectra for Li_6–*x*_PS_5–*x*_Cl_1+*x*_. Reproduced with permission from ref (40). Copyright 2019 Wiley-VCH Verlag GmbH & Co. KGaA. (b) Schematic of the cell oriented to the external magnetic field. (left). ^7^Li CSI images of pristine (middle) and cycled (right) cell. Reproduced with permission from ref (419). Copyright 2019 American Chemical Society.

MAS NMR is an indispensable technique used for SSBs to determine local structure and ion dynamics in materials.^678,679^ The technique provides enhanced NMR resolution by spinning the sample at a certain angle (the “magic angle”) with respect to the magnetic field direction and averaging the anisotropic interactions between nuclear spins.^669^ The main purpose of using MAS NMR for SSBs is to investigate local coordination environments of Li in SSEs and electrodes. ^27^Al and ^71^Ga MAS NMR has been used for Al- and Ga-doped LLZO SSE to investigate species formed at the interface and to understand the precise site occupancy in this material.^680,681^Figure 33a shows ^7^Li MAS NMR spectra of LPSC argyrodite with varying levels of halogen substitution. The authors attributed the shifting isotropic resonance to lower frequency with increasing Cl^–^ substitution to a withdrawing of electron density from Li.^40^ Karasulu et al. used ^27^Al, ^71^Ga, and ^17^O NMR on Al- and Ga-doped LLZO to determine defect sites near dopants in the SSE.^681^^31^P MAS NMR was used to investigate interfacial stability between carbon-coated LiFePO_4_ and a Li_3+*x*_P_1–*x*_Si_*x*_O_4_ SSE, and structural changes were observed upon Fe migration into silicate environments.^682^ MAS NMR studies of various cathode materials has been discussed elsewhere.^683^

Another application of ss-NMR is to investigate the microstructural behavior of Li growth by measuring NMR signals and ^7^Li chemical shift as a function of position in a SSB cell (Figure 33b).^419^ In these experiments, the comparison of the spectra between the pristine and cycled states of the symmetric cell revealed the formation of dendritic Li. The chemical shifts of Li in the SSE and Li metal are located at different regions in the spectra. Two-dimensional chemical shift imaging (CSI) reveals the formation of dendritic microstructural Li species throughout the cell.^419^

##### 4.4.4.1.3 Pulsed-Field Gradient (PFG) NMR

PFG NMR is used to measure diffusion coefficients and to study ion dynamics of SSEs at 10^–2^ to 10^–1^ s time scales and ∼1 μm length scales. In PFG-NMR, a series of magnetic field gradient pulse pairs are applied with varied diffusion times (the interval between two gradient pulses) and magnetic field gradient strength. Diffusion coefficients can be extracted from the Stejskal and Tanner equation, which is the relationship between NMR signal intensity with applied gradient pulse and the diffusion coefficient of the molecules in the sample.^618,619,684^ PFG NMR has been used to measure long-range Li diffusion in various ionic conductors such as LGPS,^685^ LSPS,^686^ argyrodites,^40,687^ (Li_2_S)_7_(P_2_S_5_)_3_,^688^ garnets,^689^ and Li_7_SiPS_8_.^690^

##### 4.4.4.1.4 Two-Dimensional Exchange Spectroscopy (2D EXSY) NMR

2D EXSY NMR is another related technique that is useful for investigating dynamic processes such as transport rates and molecular paths between sites, and it can be sensitive to exchange between isotopes such as ^7^Li and ^6^Li.^674,691^ It has been used to investigate charge transfer between Li_6_PS_5_Br and Li_2_S,^692^ LiI coatings on Li_2_S electrodes,^691^ and a variety of other SSE systems.^693,694^

**Figure 34:**
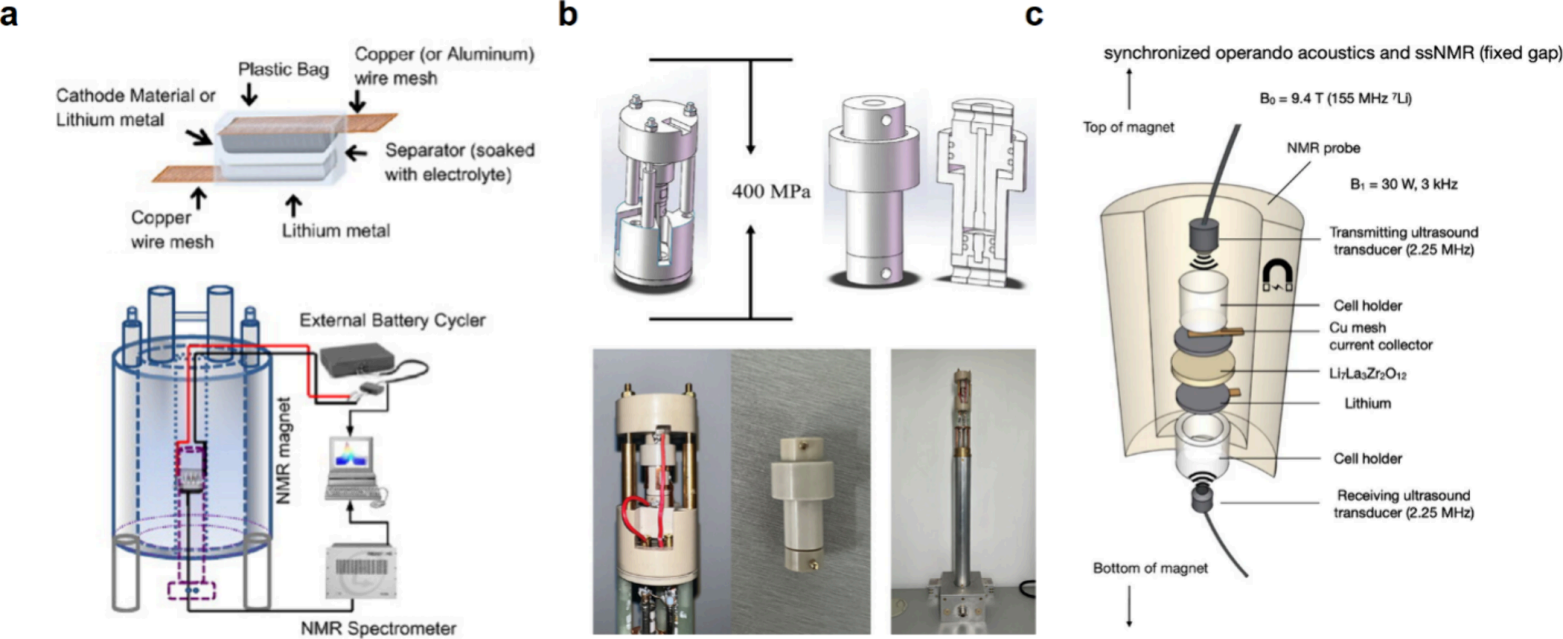
Schematics of *in situ* NMR spectroscopy cell designs. (a) Design of a bag-cell battery. Reproduced with permission from ref (695). Copyright 2012 Elsevier. (b) Schematics and photos of an *operando* NMR cell that enables stack pressure application and the cell connected to the probe. Reproduced with permission from ref (696). Copyright 2023 Wiley-VCH GmbH. (c) Schematic of the cell used for synchronized *operando* acoustic transmission and ss-NMR spectroscopy. Reproduced with permission from ref (697). Copyright 2021 Springer Nature.

Most NMR analysis on SSBs has been performed *ex situ*. *In situ* NMR can provide rich structural and dynamic information related to electrodes, electrolytes, and interfaces; however, several challenges associated with NMR, such as poor temporal resolution and difficulties in cell design, make such experiments relatively rare. The design of an *in situ* NMR cell plays an important role in obtaining high-quality data. Figure 34a demonstrates a cell design for Li-ion batteries. The use of this design for ss-NMR is straightforward because of the flexible case, but this design lacks external pressure application during cycling.^695^ Chen et al. presented an *operando* cell design for a ^6^Li–In|Li_10_GeP_2_S_12_|In SSB cell to investigate Li transport in the SSE during electrochemical operation. The cell was composed of a PEEK casing and could support up to 400 MPa external pressure (Figure 34b).^696^ The authors investigated the ionic conductivity and phase transition properties of LGPS materials. The results indicated that a phase transition occurs from the beta to gamma phase of the Li_3_PS_4_ matrix at current densities above 0.5 mA cm^–2^. The authors attributed this to stress evolution due to anode alloying and discrepancies between local ionic transport coefficients. Chang et al. employed *operando* ss-NMR and acoustic transmission techniques to investigate interfacial contact and microstructural evolution at the Li–LLZO interface. The authors demonstrated a fixed gap cell design in which two wide-band ultrasonic transducers were attached to a PTFE cell holder with epoxy. To improve the signal-to-noise ratio, the same cell was used for ss-NMR for investigating the rate of microstructure formation and for acoustic transmission to probe interfacial contact mechanics (Figure 34c).^697^ The results demonstrated stack pressure-dependent interfacial dynamics; including void formation due to insufficient contact at the interface at low stack pressures, creep-type interfacial dynamics at intermediate stack pressures, and cell shorting and fracture due to local Li yielding at high stack pressures. A final example of *operando* NMR spectroscopy demonstrated failure mechanisms and quantified (in)active Li in anode-free SSBs with LPSC, LGPS, LPS, and Li_9.54_Si_1.74_P_1.44_S_11.7_Cl_0.3_ SSEs along with LCO cathode composites. The plated Li metal was found to react with LGPS to form inactive Li at the interface.^342^

In conclusion, NMR spectroscopy provides detailed information about local structure, electronic properties, and ion diffusion characteristics. Implementation of *in situ* NMR can significantly enhance our understanding of ion dynamics in SSB materials, and it has been used in multiple studies. We note that magnetic resonance imaging (MRI) is based on NMR and provides imaging capabilities. The details and applications of MRI are discussed in Section 4.3.4.

##### 4.4.4.2 Electron Paramagnetic Resonance (EPR)

Electron paramagnetic resonance (EPR), or electron spin resonance, has a similar working principle as NMR, but EPR detects unpaired electrons. Like protons, electrons have spin and this creates a magnetic moment. When an external magnetic field is applied to a sample, the magnetic moments of unpaired electrons (i.e., paramagnetic electrons) are aligned parallel or antiparallel to the applied magnetic field direction. This alignment creates two energy levels for the unpaired electrons, and EPR measurements are made between these energy levels. Thus, EPR spectroscopy measures and interprets the energy differences between atomic/molecular states. As many materials have unpaired electrons, this technique can be applied to a wide range of materials, such as those containing transition metals ions and free radicals.

**Figure 35:**
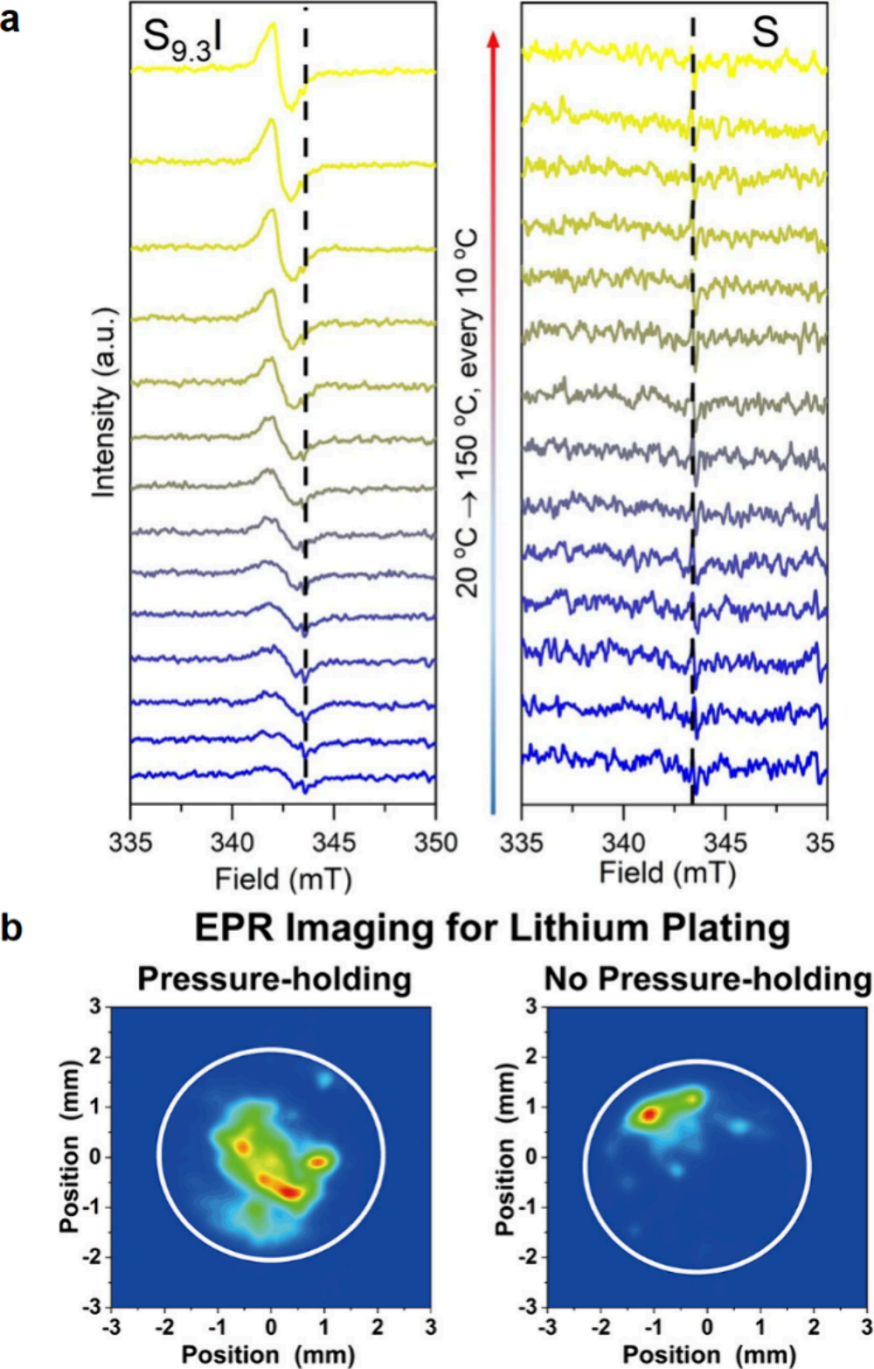
EPR studies. (a) Variable temperature EPR spectra of S_9.3_I and elemental S as a cathode for a solid-state Li–S battery. Reproduced with permission from ref (698). Copyright 2024 Springer Nature. (b) *In situ* EPR imaging for the first and second cycles of an anode-free SSB with Li–In|LPSC|Cu configuration. Reproduced with permission from ref (699). Copyright 2023 American Chemical Society.

*In situ* and *operando* EPR has been used for Li-ion batteries to characterize the coordination environment of electrodes that have paramagnetic electrons, Li interactions and degradation mechanism, and electrodes that contain transition metals.^700−704^ The use of EPR for SSBs has been limited to *ex situ* structural analysis of SSEs and electrodes, as well as *in situ* EPR imaging to study Li deposition.^698,699,705^ Wolfenstine et al. investigated the stability of an LLZO SSE before and after reaction with molten Li and observed the emergence of a magnetic field signal at ∼3500 G, indicating the formation of unpaired electrons that are trapped at O vacancies.^705^ Electronic structure analysis can be performed on anode or cathode materials that have unpaired electrons. Zhou et al. performed an *in situ* EPR study on S cathodes for SSBs at varied temperatures (Figure 35a).^698^ Comparison of spectra between S and I-incorporated S showed that increased radical formation with increasing temperature improves the electronic conductivity of the cathode. Jiang and co-workers used EPR-based imaging to observe the effect of a 360 MPa holding pressure on Li metal plating within a cell.^699^ The authors found that pressure promotes deposition of Li over a larger area on a Cu current collector (Figure 35b). These studies show that EPR has the capability for providing rich information related to electrode materials and is compatible with *in situ* or *operando* testing.

#### 4.4.5 Other Techniques

##### 4.4.5.1 Mass Spectrometry

Mass spectrometry is an analytical technique for identifying unknown substances, quantifying known samples, and elucidating the chemical properties of materials. In mass spectrometry, ions from a sample are generated by a high-energy electron beam. These ions are separated in an analyzer with a magnetic or electric field since ions with distinct mass-to-charge ratio (*m*/*z*) will follow different trajectories. Since the charge of the ion is known, the ion mass is revealed with the measurement. The main advantage of this technique is that it helps to identify materials in solid, liquid, or in gaseous phases.^706^

**Figure 36:**
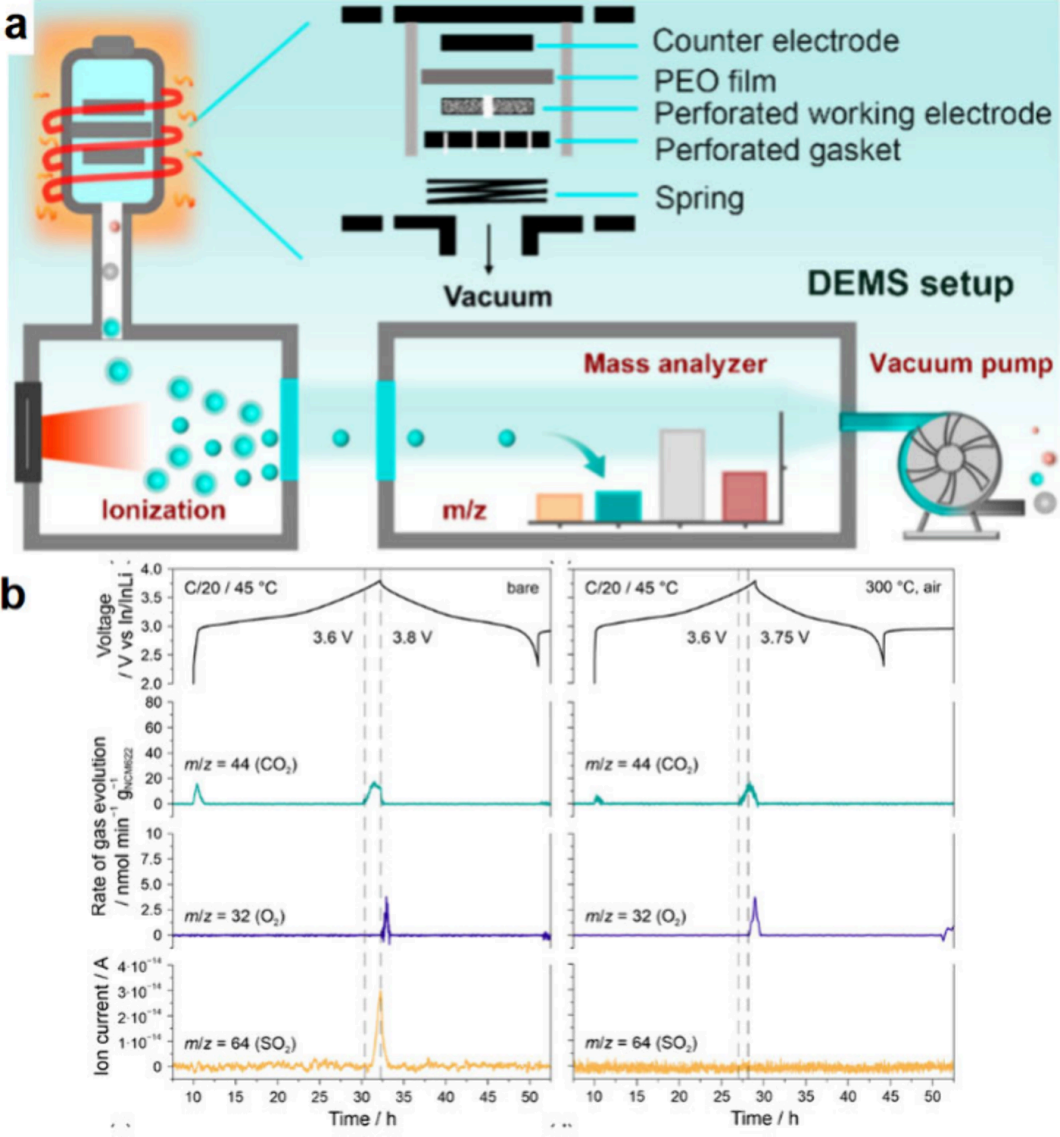
DEMS studies on SSBs. (a) Schematic of a DEMS setup and an *in situ* DEMS cell. Reproduced with permission from ref (709). Copyright 2020 American Chemical Society. (b) Voltage profile of the first cycle and corresponding DEMS measurements of NMC cathodes prepared under different conditions (uncoated vs. coated and annealed at 800 °C in air). Adapted with permission from ref (710). Copyright 2020 American Chemical Society.

Mass spectrometry can be combined with other techniques to investigate various aspects of materials. For example, differential electrochemical mass spectrometry (DEMS) integrates electrochemical analysis with mass spectrometry and allows for *in situ* tracking of gas evolution from battery materials.^707,708^ The schematic in Figure 36a shows a DEMS setup and the design of a LiCoO_2_|PEO-LiTFSI|Li cell.^709^ Bartsch et al. reported the first DEMS study on a SSB, which included an In anode, a Li_3_PS_4_ SSE, and a NMC622 cathode. The results indicate that H_2_ was produced via the water reduction reaction, and a trace amount of SO_2_ was detected due to β-LPS oxidation. By using the ^13^C-isotope labeling method, the authors confirmed the formation of CO_2_ gas due to residual carbonate species.^658^ Another study showed the effect of synthesis conditions of the NMC622 cathode on the evolution of carbonate species and gaseous products due to interactions between the SSE and cathode.^710^ DEMS measurements demonstrated that the cathode heated at 300 °C in O_2_ possessed fewer carbonate species and provided better interfacial stability in contact with the LPSC SSE (Figure 36b).^710^ The effect of a coating layer on NMC622 has been studied by Kim et al. by utilizing pure Li_2_CO_3_ and Li_2_CO_3_-LiNbO_3_ coatings. The results reveal that the decomposition of Li_2_CO_3_ at potentials above 4.2 V vs Li/Li^+^ was diminished with LiNbO_3_ coatings.^711^ Yang et al. introduced a methodology for improving the interphase of Li_6.4_La_3_Zr_1.4_Ta_0.6_O_12_ (LLZTO) by coating LCO with the reaction products of Li_2_CO_3_ and Co_3_O_4_.^712^ DEMS analysis showed that the evolution of CO_2_ at high potentials was suppressed. Identification of decomposition products in SSBs with DEMS has been extended to a wide variety of SSEs^709,713,714^ and SSE–electrode interfaces.^715,716^

Finally, another application of mass spectrometry is time-of-flight secondary ion mass spectrometry (ToF-SIMS), which is an imaging technique used to elucidate elemental and molecular information from solid surfaces. The technique and its application to SSBs are discussed in Section 4.3.7.

### 4.5 Mechanical and Thermal Characterization Techniques

#### 4.5.1 Mechanical Characterization Techniques

##### 4.5.1.1 Force and Displacement Measurements of SSBs

The stack pressure applied to a SSB is a critical, and often underemphasized, parameter that influences electrochemical behavior.^717^ Without a liquid electrolyte that wets the surface of each electrode, the application of pressure ensures that physical contact is maintained at interfaces to allow for ionic and electronic transport. Though the influence of stack pressure is usually more pronounced when using inorganic SSEs compared to polymeric SSEs, the vast majority of studies on SSBs utilize some level of stack pressure for cell testing. For commercial applications, stack pressure needs to be minimized (<1 MPa) since bulky housings for stack pressure application negate energy density advantages.^13^ Thus, understanding the influence of stack pressure and reducing its magnitude are urgent areas of investigation for SSBs.

Although external pressure is usually applied to SSBs, the resulting internal pressure is dependent on the nature of confinement within the cell housing, as well as the local environment around electrode materials that undergo volume changes during cycling. The partial molar volume of Li is different in different electrode materials, leading to different magnitudes of volume change (ranging from zero to 300%) during insertion/extraction. SSBs are a complex electro-chemo-mechanical system, with the mechanical aspect being necessary to understand in more detail. To this end, force sensors can be incorporated into a SSB stack to monitor stress changes without interrupting the operation of the cell, and this has become a popular characterization method.

**Figure 37:**
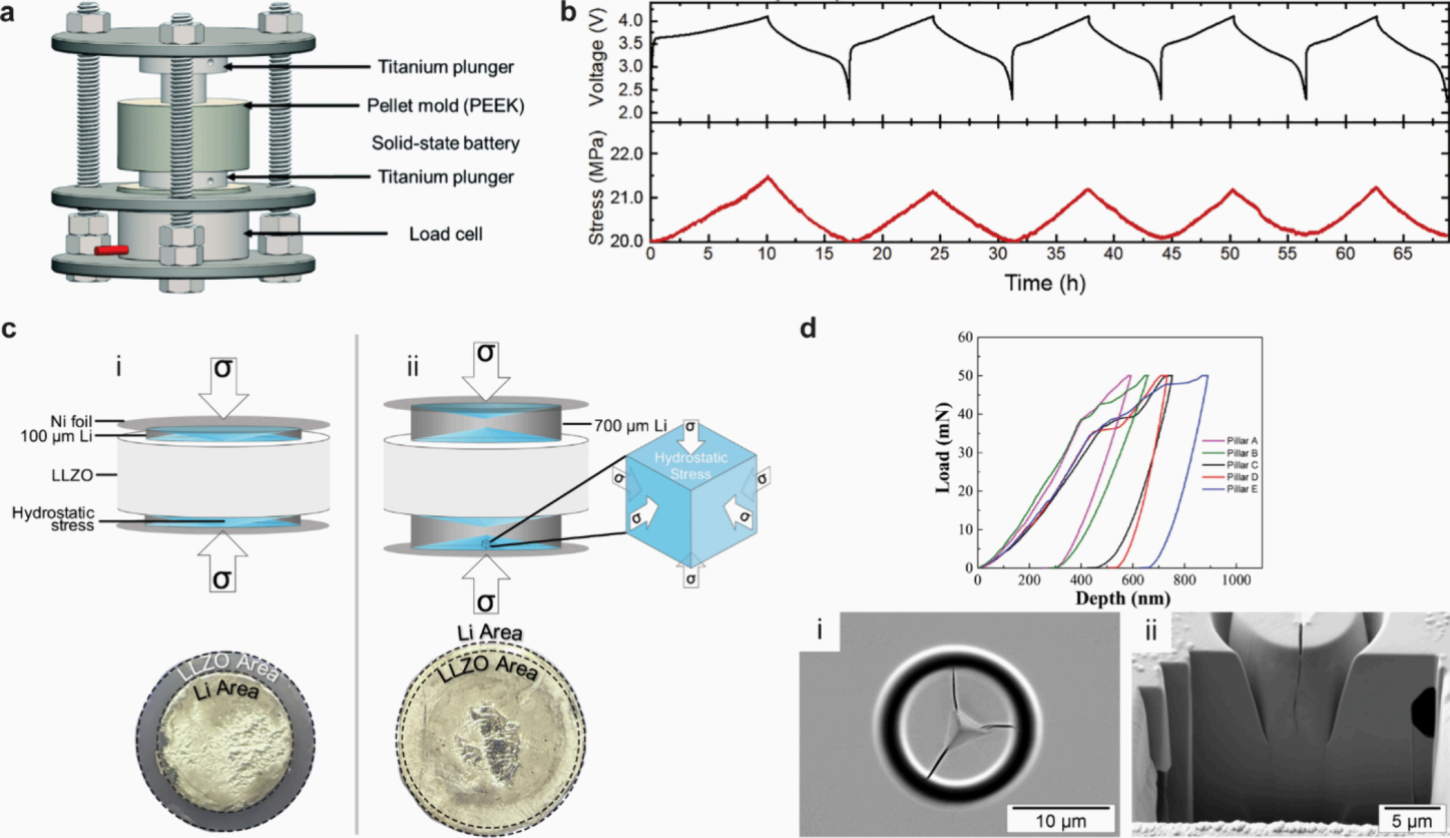
Applying and measuring forces within SSBs *ex situ*, *in situ*, and *operando*. (a) Schematic of a SSB housing with an integrated force sensor for *in situ* and *operando* force and pressure monitoring. Adapted with permission from ref (314). Copyright 2020 Royal Society of Chemistry. (b) *Operando* stack pressure measurement showing the voltage profile (top) and measured stack pressure (bottom) for a Si/LPSC composite anode cycling with an NMC-111 composite cathode and LPSC SSE. Reproduced with permission from ref (296). Copyright 2021 Elsevier. (c) Schematics (top) and resulting Li coverage (bottom) of Li on a LLZO SSE with a hydrostatic pressure applied to Li of different thicknesses ((i) 100 μm and (ii) 700 μm). Adapted with permission from ref (721). Copyright 2022 Elsevier. (d) Load vs depth curves (top) from nanoindentation measurements performed on micron-scale pillars of LLZO SSE; panels i and ii show deformation of pillar B of the LLZO. Adapted with permission from ref (726). Copyright 2018 Elsevier.

A typical setup for measuring the force (and therefore stress) within a SSB stack is shown in Figure 37a, where a force sensor is incorporated between the SSB and the steel plates used to apply pressure to the battery stack.^314^ In this study, the effects of different densification and stack pressures on the cycling stability of NCA/LPSC/LiIn cells were investigated. Such force sensor measurements can also be used for *operando* measurement of the evolving stack pressure, as shown in Figure 37b, where subtly different stress evolution was found when using different alloy anode composites in SSBs.^296^ Other researchers have used similar experimental setups to characterize the mechanical evolution of SSB systems using a variety of SSE and electrode combinations (refs (130, 171, 300, 301, 718−720)). This has provided insight into important chemo-mechanical concepts, including “mechanical matching” of opposing electrodes to compensate volume changes and reduce total stress buildup in the cell,^171^ the generation of stress due to interphase reactions,^130^ and enhanced stress dissipation when using smaller active material particle sizes.^296^ Beyond these experiments, the influence of applied pressure on the deformation of electrode materials such as Li has been investigated.^721^ For instance, the thickness of a Li metal electrode in contact with a rigid electrolyte has been found to strongly influence its creep behavior (Figure 37c), with implications for contact evolution at SSB interfaces.^721^ Other researchers have used pressure application experiments to measure the altered electro-chemo-mechanical properties of SSB systems under varying mechanical^722−724^ and electrochemical^725^ conditions.

While a variety of SSB chemistries have been characterized with different *in situ* and *operando* stress measurements, most of these investigations have involved measuring stress evolution of SSBs with relatively large externally applied stack pressure (i.e., >10 MPa). There is still a lack of understanding of stack pressure effects in systems designed and able to operate at lower stack pressures, as well as how stress evolves in systems engineered for stress dissipation.

##### 4.5.1.2 Measuring Mechanical Properties

Load frames (such as tensile and compression testers) and micro-to-nanoscale indention testing have been widely used to characterize the mechanical properties of electrode materials used in SSBs. These systems typically operate by applying a load or displacement to a sample and measuring the resulting displacement or load. Through such experiments, mechanical properties like hardness (through indentation), tensile strength, fracture toughness, time-dependent creep, and fatigue (through cyclic loading) can be quantified, allowing for informed decisions on the processing and operational parameters that are best suited for different SSB materials. Experiments have been performed on electrodes like Li metal,^163,165,727−729^ Na metal,^730−732^ and Si^733−739^ at the macro-, micro-, and nanoscales. While many of these experiments were not explicitly performed for SSB systems, the results are highly relevant to the chemo-mechanical evolution of these materials in SSBs.

The mechanical properties of SSE materials are also of high interest. Researchers have used mechanical measurements and nanoindentation to investigate the mechanical properties of LiPON,^740−743^ LLZO,^43,726,744−747^ LATP,^748−750^ LLTO,^751,752^ LAGP,^43^ LSPS,^43,753−755^ and LPSC,^43^ among others. Fracture properties are of particular interest for SSBs, since fracture of the SSE can result in cell failure. Nanoscale fracture characteristics of SSEs obtained through nanoindentation are important for alkali metal SSBs since alkali metals exhibit strong size dependence of yield strength at small scales. Wang et al. performed nanoindentation on LLZO micropillars with varying microscale dimensions, obtaining different loading patterns for the different pillars and providing insight into interfacial design of SSEs for improved mechanical properties (Figure 37d).^726^

##### 4.5.1.3 Optical Techniques for Stress and Strain Measurement

**Figure 38:**
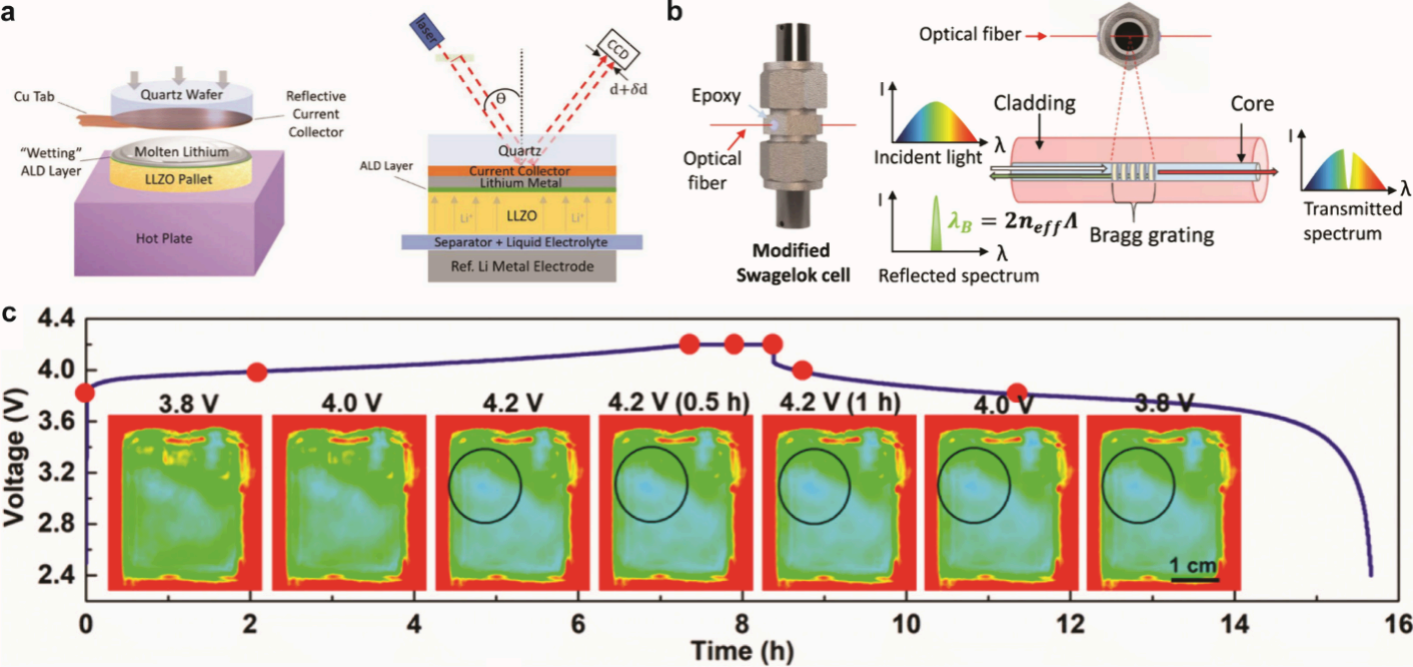
*In situ* and *operando* mechanical characterization of SSBs using optical sensors, image analysis, and acoustic methods. (a) Schematics of the battery configuration (left) and sensing experiment (right) of an *in situ* multibeam optical stress sensor (MOSS) experiment on an LLZO SSE symmetric cell. Reproduced with permission from ref (759). Copyright 2022 Wiley-VCH GmbH. (b) Schematic of an *operando* fiber Bragg grating (FBG) experiment to measure strain evolution within LPS SSE-based cells. Reproduced with permission from ref (761). Copyright 2022 Springer Nature. (c) Voltage profile with inset ultrasonic images of an LCO/LLZTO/Li SSB pouch cell. An evolving gaseous region represented by a blue color is circled in black. Reproduced with permission from ref (763). Copyright 2022 American Chemical Society.

Optical sensor methods have been used for characterizing the internal stress/strain and thermal evolution of Li-ion batteries.^756^ One such technique used for measuring stress in thin films is the multibeam optical stress sensor (MOSS) method. MOSS is conducted by measuring the deflection of an array of parallel laser beams from the back of a wafer on which a material of interest is bonded. If a battery material is on the wafer, the stress and strain within this material during reaction processes can be measured. MOSS is useful as it allows for direct internal stress measurements of battery electrode materials without requiring intrusive internal components. A number of investigations have used MOSS to observe stress and strain evolution in alloy thin-film batteries.^739,757,758^ A schematic for performing MOSS on SSBs is shown in Figure 38a; here, Cho et al. used a quartz wafer to support an anode current collector to observe the strain during deposition and stripping of Li metal.^759^ Other groups have used similar *operando* experiments on other SSB systems.^760^

Fiber Bragg grating (FBG) is another optical characterization method. FBG operates by placing an optical fiber with a periodically varying refractive index inside a battery cell. As the fiber is strained due to deformation from neighboring electrode materials undergoing electrochemical reaction or temperature variations, the Bragg wavelength is shifted within the fiber, allowing for precise measurements of internal stress and strain evolution. The drawback of FBG is that it requires the internal structure of the battery to be modified through insertion of the fiber, potentially altering electrochemical behavior. While FBG techniques have primarily seen use in conventional Li-ion battery systems, they have also been used in SSBs to investigate stress and strain evolution both *in situ* and *operando*. Blanquer et al. carried out *in situ* FBG experiments on an SSB (Figure 38b). Measurements taken with the embedded cable were shown to differ from those measured externally, highlighting the importance of exploring buried interfaces in SSBs *in situ* and *operando*.^761^ Other groups have similarly embedded optical fibers into SSBs to explore evolving strain, investigating cells with polymeric SSEs that could promote stress relaxation.^295,762^ In general, optical sensing techniques offer a direct measure of strain within SSBs, warranting further investigation of high-capacity electrode materials.

##### 4.5.1.4 Acoustic Techniques

Acoustic techniques involve transmitting acoustic vibrational patterns through a sample and recording how they are modified upon their reception at a sensor. Different materials transmit acoustic waves differently depending on their mechanical properties, allowing for correlation of acoustic signals to the internal structure of batteries; these methods are advantageous as they can be performed on completely sealed batteries using an ultrasonic transmitter. This allows for nondestructive and noninterfering spectroscopic and imaging characterization of the mechanical state of batteries and is valuable for analyzing the state-of-health of commercial Li-ion batteries.^764−766^

An application to SSBs is shown in in Figure 38c, where acoustic transmission was used to identify gas production in an SSB cell.^763^ The different acoustic transmittance of the gas pocket compared to the rest of the battery allowed for identification of the problematic region. Other experiments have been conducted on LLZO SSE systems, where it was shown that the evolving Li interface^697^ and dendrite growth^767^ can be observed with ultrasonic imaging. Researchers have also studied the acoustic behavior of LiPON^768^ and polymeric SSEs.^762^ These experiments highlight the multiple modes of acoustic characterization, which can be used to generate ultrasonic images *in situ* and *operando*(762,763) or acoustic spectra^697,767,768^ of the sample. These techniques could provide opportunities for characterization of the degradation of commercial-scale batteries as SSBs are scaled up due to the capability to observe degradation mechanisms nondestructively.

#### 4.5.2 Thermal Analysis

Thermal analysis is used to understand the chemical and physical properties of materials as a function of temperature. Various techniques are widely applied to explore phase transitions, thermal decomposition of materials, and thermal conductivity,^769−771^ which are of high relevance for battery research.^772^ Two popular characterization methods are thermogravimetric analysis (TGA) and differential scanning calorimetry (DSC), which are rapid and can conveniently be used with relatively small sample quantities.

##### 4.5.2.1 Thermogravimetric Analysis (TGA)

**Figure 39:**
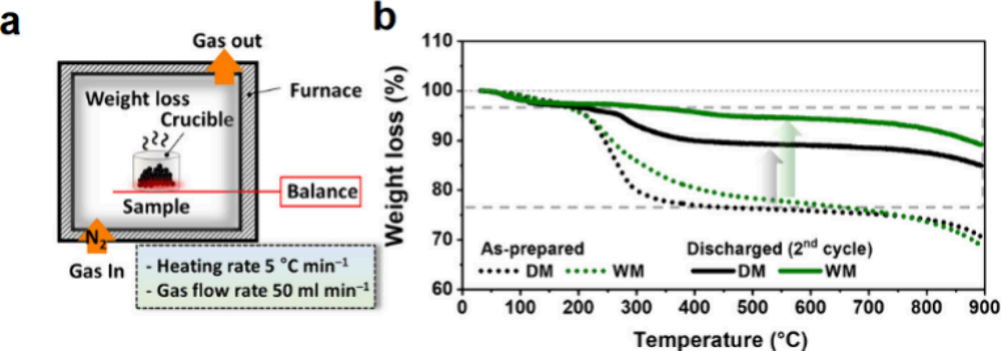
TGA studies of SSB materials. (a) Schematic of the sample TGA experiment. (b) TGA curves of pristine and after two galvanostatic cycles of a sulfur cathode. Reproduced with permission from ref (773). Copyright 2023 American Chemical Society.

Thermogravimetric analysis (TGA) records the mass loss or gain of a sample as the temperature is swept at a given rate (Figure 39a).^773^ This technique can probe thermal decomposition of materials or other reaction processes that cause mass changes (Figure 39b).^773^ TGA data can enable quantitative analysis of phenomena such as compositional changes, thermal stability limits, melting points, glass transition temperatures, and the formation of gaseous byproducts. TGA has been broadly used to explore the thermal decomposition behavior of SSB materials.^772,774^ Zhang et al. investigated the thermal stability limits of Li_6.75_La_3_Zr_1.75_Ta_0.25_O_12_ (LLZTO) with poly(vinylidene fluoride) PVDF as a composite electrolyte membrane. While the decomposition temperature of pristine PVDF is ∼500 °C, the decomposition temperature decreased to 310 °C with the addition of 10% LLZTO.^775^ TGA can also be used to identify the mass loading of composite components in SSBs.^773,776−778^ Kim et al. fabricated a S-LPSC cathode composite with both wet mixing and dry mixing. TGA indicated that S begins to vaporize between 200 and 300 °C, but that the composite prepared by wet mixing exhibits an extended vaporization curve indicating better binding between the SSE and the S cathode.^773^ Another application of TGA in SSBs is to identify phase transitions, such as the transition between the cubic and tetragonal phases of LLZO.^779^

##### 4.5.2.2 Differential Scanning Calorimetry (DSC)

Differential scanning calorimetry (DSC) records heat flow as the temperature of a sample is varied. It can be used to identify first- and second-order phase transitions because of the distinct enthalpy changes that occur during these transitions. Two methods can be used to measure heat flow: i) a furnace is heated at a defined heating rate, and the heat is transferred to the sample and reference pan. The temperature difference between the sample and reference pan is measured, with any differences being due to the heat capacity of the sample being tested. ii) The sample and the reference pan are kept at the same temperature during heating in separate furnaces, and the difference of the thermal power required to maintain consistent temperature is recorded.

**Figure 40:**
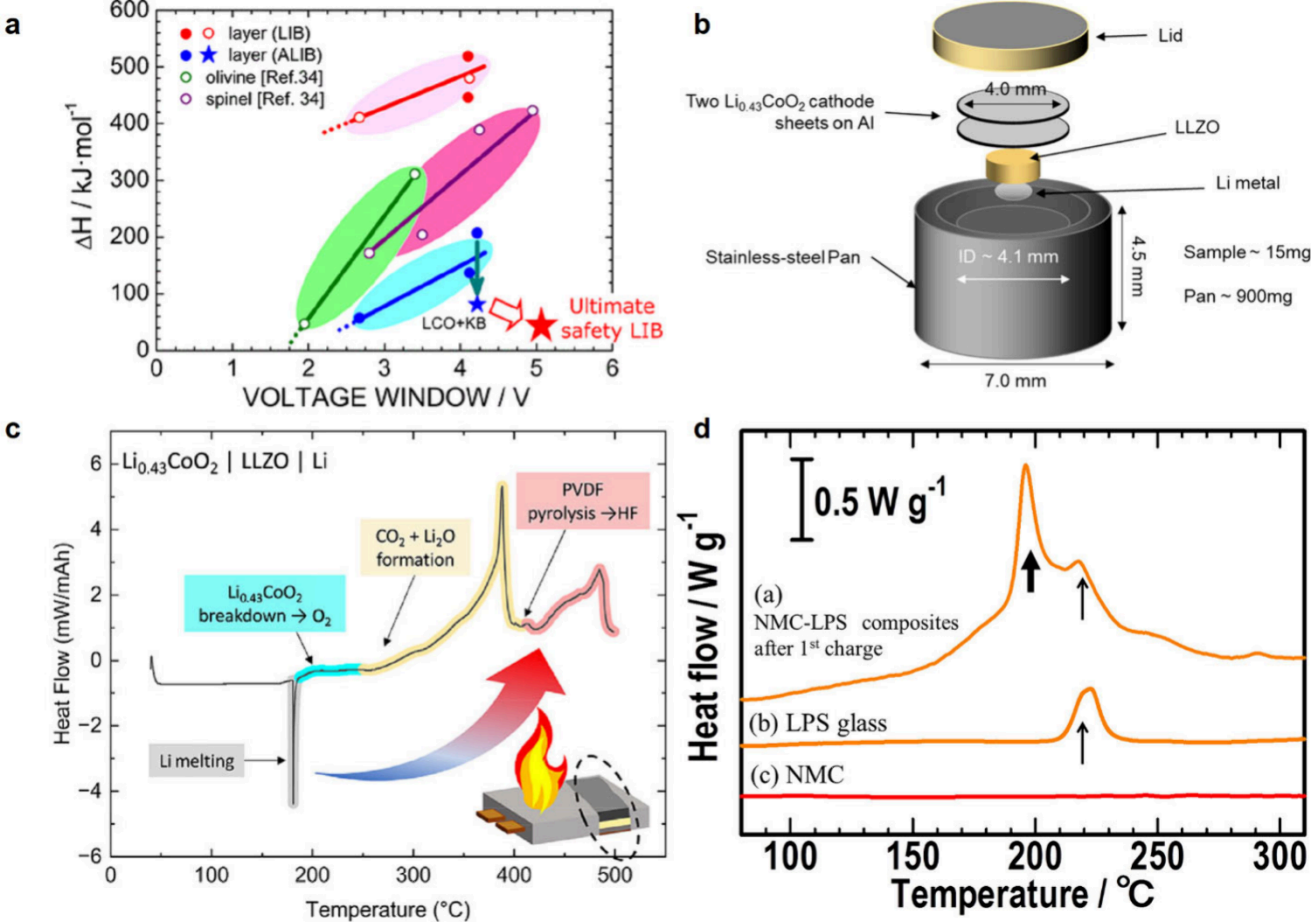
DSC studies on SSBs. (a) Safety map of Li-ion and SSBs generated from DSC analysis. Reproduced with permission from ref (787). Copyright 2017 American Chemical Society. (b) Schematic of a DSC pan for Li–LLZO–LCO SSB heat flow analysis. (c) DSC curve of a Li–LLZO–LCO SSB. (b,c) Reproduced with permission from ref (788). Copyright 2023 American Chemical Society. (d) Comparison of DSC curves of LiNi_1/3_Mn_1/3_Co_1/3_O_2,_ 75Li_2_S·25P_2_S_5_ SSE, and cathode–SSE composite. Reproduced with permission from ref (789). Copyright 2017 Elsevier.

DSC has been used for measuring the thermal behavior and stability limits of Li-ion battery materials,^780,781^ SSEs,^782−784^ cathodes,^774^ electrode–electrolyte interphases,^785,786^ and other battery components. Inoue et al. used DSC to compare the thermal runaway behavior of Li-ion batteries to that of SSBs containing various anodes. The SSBs (which contained garnet SSE and LCO cathodes) exhibited exothermic reactions at elevated temperatures, but the total heat generation from the SSBs was approximately 30% lower than that in traditional Li-ion batteries (Figure 40a).^787^ Various cell components of SSBs can contribute to exothermic reactions, such as Li or carbon as reductants and oxide cathodes as an oxidant. Therefore, measuring the stability limits of SSB component stacks could provide useful insights for safer SSBs.

A sealed cell design for DSC testing is important for preventing gas leaks during the heating process. Johnson et al. built a microcell to test the thermal safety of a LCO|LLZO|Li stack in a hermetically sealed stainless-steel pan assembly (Figure 40b).^788^ The anode and cathode had matching capacity and the LLZO was thick enough to prevent short circuits. DSC showed that O_2_ was released due to a reaction between carbon additives and LCO at 230 °C, while the PVDF binder pyrolyzed to release HF at 400 °C (Figure 40c).^788^ These findings imply that the interaction between components in a composite electrode could lead to exothermic reactions. Tsukasaki et al. investigated the thermal stability and crystallization behavior of a LiNi_1/3_Mn_1/3_Co_1/3_O_2_ cathode, a Li_2_S-P_2_S_5_ SSE, and a composite mixture of these materials (Figure 40d).^789^ The DSC curve of the SSE showed a single exothermic reaction at 220 °C due to the crystallization of LPS, while crystallization in the composite occurred at a lower temperature. This indicates that interfacial interactions in the composite influenced crystallization behavior.^789^ DSC in conjunction with mass spectrometry has been used to explore thermal runaway mechanisms of sulfide-based glass–ceramic (Li_3_PS_4_ and Li_7_P_3_S_11_) and crystalline (Li_6_PS_5_Cl and Li_10_GP_2_S_12_) SSEs with LiNi_0.8_Co_0.1_Mn_0.1_O_2_ cathode materials.^24^ Crystalline SSEs remained stable against O_2_ until 200 °C. In contrast, glass–ceramic SSEs generated SO_2_ gas at approximately 200 °C because of the reaction with O_2_ released from the cathode. Furthermore, heat generation from both types of SSEs with NMC cathodes was much higher than NMC cathodes in liquid electrolytes.

## 5 Li Metal Electrodes

### 5.1 Challenges of Li Metal Anodes in SSBs

While Section 4 provided detailed information on an array of characterization methods used for SSBs, this section presents information on the characterization of Li metal anodes in SSBs and our current understanding of the fundamental behavior of Li metal anodes derived from these characterization efforts. Li metal anodes are lightweight and have a low electrode potential, leading to high voltage batteries. However, as discussed in Section 3, moving from a liquid electrolyte to a SSE gives rise to novel electro-chemo-mechanical phenomena and issues at the Li–SSE interface. Challenges include the loss of contact (void formation) during stripping, Li filament growth during plating, and interphase formation. These factors have hindered the implementation of Li metal anodes in SSBs. Electro-chemo-mechanical aspects of Li at the SSE interface are critical to understand for controlling the interfacial evolution of Li in SSBs.

### 5.2 General Chemo-Mechanical Considerations

The yield strength of bulk Li has been measured to be ∼0.8 MPa, and if the external stress exceeds this value, slip from dislocation glide leads to plastic deformation.^163,165,728,729^ Li metal exists at a relatively high homologous temperature at room temperature (0.65 at 25 °C), leading to creep behavior at stresses lower than the yield strength.^165,790^ The mechanical deformation of Li can help to retain contact at SSE interfaces to some extent, but applied stack pressure cannot entirely overcome all morphological instabilities. This is due to the complex nature of Li metal deformation, which varies with strain rate and length scale.^728^ Prior work has shown that the Li yield strength ranges from 0.2 to 1 MPa at strain rates from 10^–6^ to 1 s^–1^ and is also dependent on the Li domain size. These trends suggest that stress evolution within Li metal varies depending on battery cycling rates and the micro-to-nanoscale morphology at the SSE interface.^170,728,729^ Furthermore, as discussed in the previous section, the achievable stack pressures in full-scale practical cells are typically no more than ∼1 MPa, which is substantially lower than the pressure levels routinely used in laboratory experiments on SSBs.^179,180,790^ These points demand a comprehensive understanding of underlying mechanisms relevant to pressure effects to overcome the morphological issues at the Li metal–SSE interface, as detailed in this section.

### 5.3 Void Formation during Li Stripping

#### 5.3.1 General Mechanism

**Figure 41:**
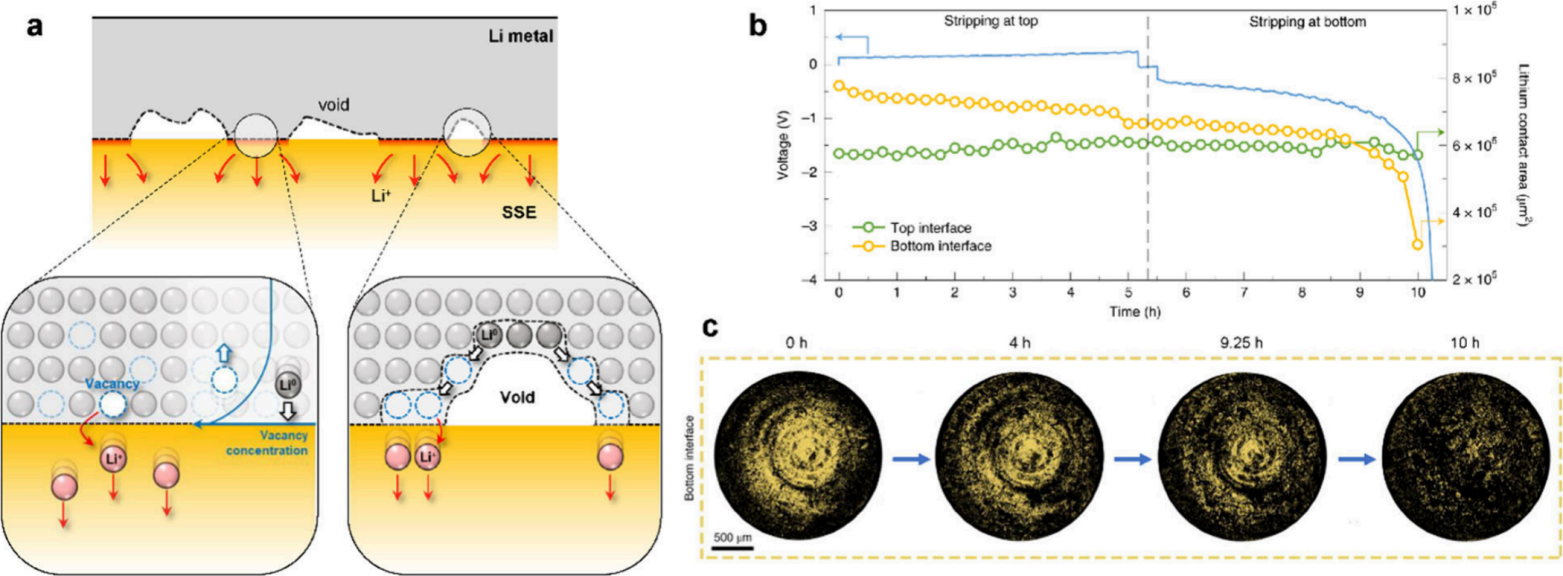
(a) Schematics of the Li metal/SSE interface during anodic dissolution. The bottom left schematic shows the case when interfacial contact is sustained when vacancies produced by Li stripping can diffuse at a faster rate than Li^+^ migration due to the anodic current. The vacancy concentration profile is depicted based on a vacancy relaxation model from literature.^27,303^ The bottom right schematic displays the case of void growth. This occurs when the anodic current load exceeds the rate of vacancy replenishment either through diffusion or mechanical deformation, causing voids to form and grow inward into the Li bulk. (b,c) Galvanostatic voltage profiles measured during an *operando* X-ray tomographic imaging experiment and corresponding contact area variation analysis for a Li–SSE interface. (b) Voltage profile of a Li symmetric cell cycling at 1 mA cm^–2^ (blue line), along with measured Li contact areas at the top (green) and bottom (yellow) SSE interfaces. (c) Li–SSE contact area maps at one interface (the yellow profile shown in panel b). The colored pixels correspond to a pixel of interfacial contact, and the black pixels denote a pixel without contact. (b,c) Reproduced with permission from ref (133). Copyright 2021 Springer Nature.

The morphological changes of Li metal during stripping at the SSE interface have been challenging to characterize, understand, and control. The rigid nature of most SSEs combined with the plasticity of Li metal make it difficult to retain conformal contact with the SSE during electrochemical stripping, resulting in contact loss at the interface. As previously mentioned, contact loss causes increased impedance and detrimental current concentrations. Generally, this behavior is caused by coalescence of Li vacancies at the SSE interface to form voids.^27,169,791^ In the absence of pressure, void formation is governed by the balance between vacancy diffusion and Li anodic dissolution (Figure 41a). Anodic dissolution results in Li^+^ ion migration away from the Li metal–SSE interface, leaving electrons and vacancies on the Li metal side of the interface. These vacancies can diffuse away from the Li metal–SSE interface, but since Li^+^ ion mobility in SSEs is typically high, the vacancy diffusion rate in the Li metal can be a bottleneck that causes vacancy accumulation and eventual void formation.^303^ If the anodic dissolution rate (i.e., vacancy formation rate) is less than the vacancy diffusion rate (Figure 41a, bottom left), interfacial contact can be maintained. If the anodic dissolution rate exceeds the vacancy diffusion rate, vacancies can accumulate at the Li–SSE interface to form voids (Figure 41a, bottom right). Li atom self-diffusivities in the bulk have been measured to be ∼10^–10^ cm^2^ s^–1^ (at room temperature) with electrochemical techniques and Li isotope tracing.^792−794^ Krauskopf et al. calculated a critical anodic current density of 10–100 μA cm^–2^ that is suitable for sustaining stable Li contact at the LLZO interface based on this value.^303^ This estimate shows the Li self-diffusion cannot be solely relied on to mitigate void formation, since current densities of >1 mA cm^–2^ are needed in practical batteries. Lu et al. used cryogenic FIB-SEM cross-sectional imaging and EIS to visualize void formation and growth.^795^ Contact loss was found to progress from 0D void nucleation to 3D extended voids and through stages of stability, transition, and failure. The observed void size was inversely proportional to current density, and the total contact loss rate was proportional to the square of the current density. Recent three-electrode electrochemical tests combined with EIS analysis have revealed that voltage polarization during Li stripping primarily arises at the interface where Li dissolves.^169,301,796^*Operando* X-ray tomography analysis established a direct link between polarization behavior and contact loss at the SSE interface (Figure 41b,c).^133^

#### 5.3.2 Effects of Stack Pressure and Current Density

During Li stripping, the growth of voids at the SSE interface causes contact loss and removes pathways for Li to transit to/from the interface. This increases interfacial resistance, causing electrochemical polarization and promoting filamentary growth during subsequent plating due to the constricted deposition current. Applying uniaxial stack pressure can deform Li metal to promote contact at the SSE interface via Li creep and plastic deformation.^797,798^ However, the local deformation of Li is complex, partially due to the strong size dependence of the Li yield strength. Kasemchainan et al. demonstrated with three-electrode electrochemical testing that the allowable current density for discharging a Li metal anode is proportional to the stack pressure, with 1 mA cm^–2^ of dissolution current requiring 7 MPa of stack pressure to retain contact at the interface.^169^ This is significantly higher than the yield strength of bulk Li metal (<1 MPa). Similarly, simulations have suggested that preventing void formation requires stack pressures over 12 MPa under practical current loads.^797,798^ This issue arises from the combined effects of current density, stack pressure, and microscale morphology evolution at the Li–SSE interface.^795,797,798^

In practice, the Li–SSE interface can exhibit heterogeneities, including roughness and nano-to-microscale contact variation resulting from insufficient contact establishment during fabrication, which affect cycling behavior under different stack pressures.^44,798^ Nanoindentation experiments have shown that the average yield strength of Li at an indentation depth of 40 nm ranges from ∼23 to 175 MPa in a strain rate range of 0.195–1.364 s^–1^, decreasing to ∼12 MPa at 1 μm indentation depth regardless of the strain rate.^166^ This is consistent with microcompression experiments performed by Xu et al.^729^ This indicates that Li deformation at nanoscale heterogeneities at the Li–SSE interface may require very high stresses, and size-dependent deformation mechanisms are likely to influence contact evolution at small-scale heterogeneities.^799^ Additionally, the Li mechanics at the SSE interface is relevant to other interfacial characteristics, including temperature effects and the chemo-mechanics of interphase formation.^56,729,791^

### 5.4 Li Growth and Filament Formation during Plating

Ideally, Li metal should be deposited with uniform thickness under cathodic loads across the electrode between the parent metal layer/current collector and the SSE. As previously shown in Figure 4a, the molar volume of Li metal is 12.97 cm^3^ mol^–1^, meaning that a thickness of 4.85 μm of dense Li corresponds to 1 mAh cm^–2^ areal capacity. Li metal exhibits a lower elastic modulus, lower yield strength, and greater ductility compared to typical SSEs.^729,800^ This simplistically suggests that the deposited Li could mechanically deform during deposition to retain a uniform thickness and morphology. However, in many cases, the deposition of Li metal at the SSE interface is accompanied by morphological instability, such as the growth of Li filaments within the SSE. This generally causes transmission of stress to the adjacent SSE material, which can cause SSE fracture and further filamentary penetration of Li to eventually short circuit the battery.

#### 5.4.1 Effect of Interface Morphology and SSE Properties

**Figure 42:**
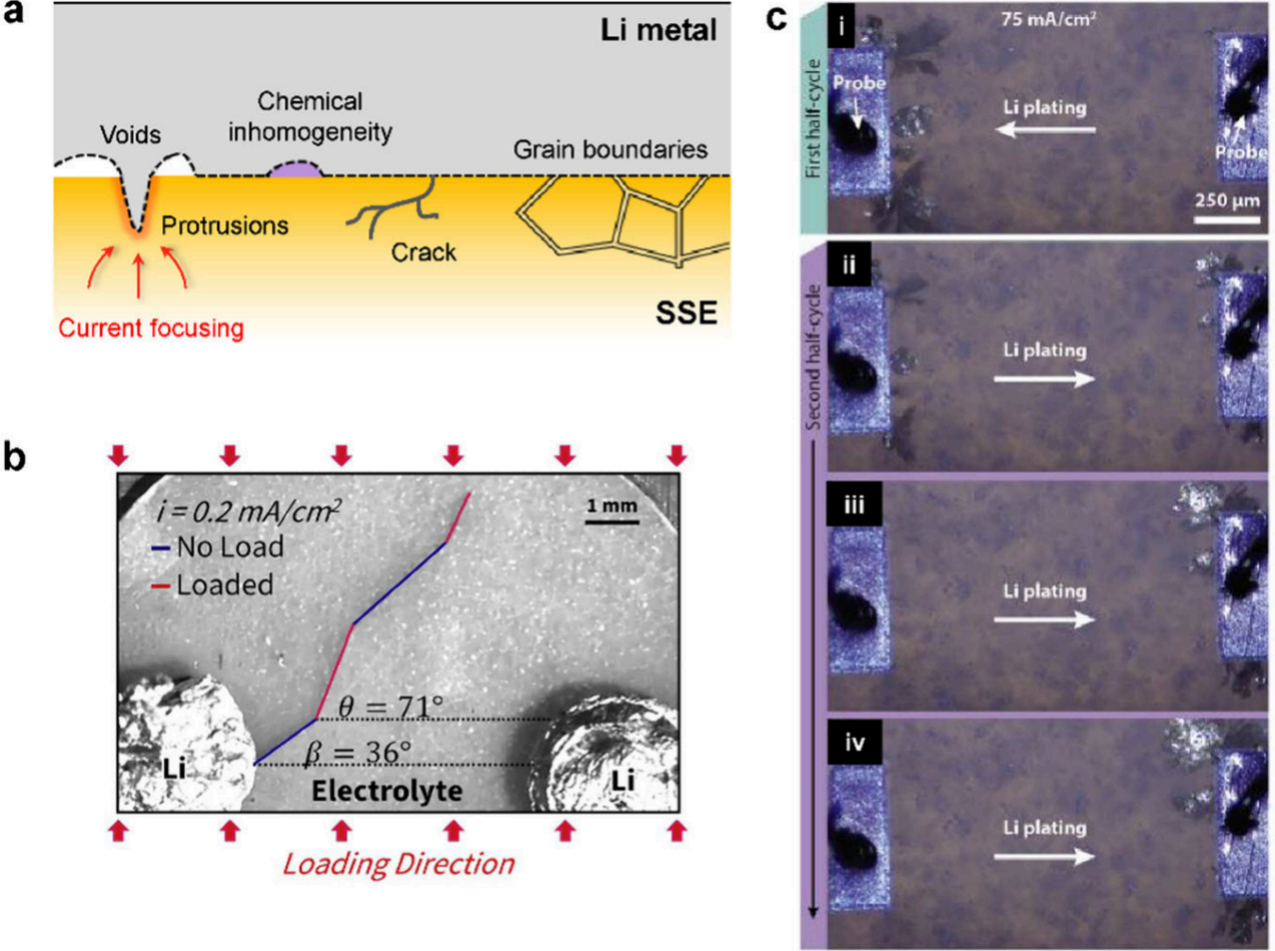
(a) Different phenomena causing inhomogeneous Li current distribution at the SSE interface, which can lead to SSE fracture and Li filament propagation through the SSE. (b) *Operando* optical microscopy imaging of propagation of a Li-filled crack through an oxide SSE and its deflections in response to the stress applied transverse to the Li transport direction. By applying 70 MPa of compressive stress, the direction of crack propagation was deflected toward the axis where the pressure was applied, demonstrating the governing influence of solid mechanics on Li filament growth. Reproduced with permission from ref (175). Copyright 2022 Elsevier. (c) *Operando* optical microscopy of SSE failure mechanisms using a Li–LLZO symmetric cell. The images show Li growth within the SSE during the (i) first and (ii–iv) second cycles. Adapted with permission from ref (363). Copyright 2020 Elsevier.

Imperfect interfacial contact or other interfacial heterogeneities are thought to cause cathodic current to be concentrated at certain points or regions of the Li–SSE interface. Li growth at these localized regions of high current density is more likely to cause fracture or spallation of the SSE, which can then become initiation points for Li filament propagation and further fracture of the SSE. Such current constriction effects during the Li plating process may originate from various sources, including the following (Figure 42a): (1) insufficient interfacial contact resulting from imperfect cell fabrication, (2) interfacial contact loss during preceding Li stripping steps, (3) chemical inhomogeneities at the interface (such as contaminants), (4) Li transport along SSE grain boundaries, and (5) pre-existing flaws at the SSE surface (e.g., defects, protrusions, or cracks) (refs (27, 44, 358, 798, 801−804)).

Physically irregular contacts between Li metal and the SSE can cause current localization, which may entail cases (1), (2), and (5) above. Singh et al. demonstrated the influence of SSE roughness on initial contact formation during cell fabrication using EIS, cross-section imaging with cryo-FIB-SEM, and finite element analysis. Interfacial impedance was compared at various contact formation pressures using an argyrodite sulfide SSE, revealing that stack pressures of >100 MPa were required to create conformal contact.^44^ Finite element analysis of digitalized FIB-SEM images demonstrated that microscale heterogeneities, such as SSE protrusions and Li–SSE disconnections, can significantly alter current pathways, leading to nonuniform Li deposition.^44,801^ A similar effect was also observed near interfacial voids. Other work used simulations to verify substantial distortions of current densities at the edges of microscale voids, with a nominal current density of 0.5 mA cm^–2^ found to be concentrated at the void edges and amplified up to ∼1000 mA cm^–2^.^802^

Surface defects at SSE interfaces include cracks, chemical inhomogeneities, and grain boundaries. These features can alter Li growth rates, causing Li penetration into SSEs.^358,805^ Porz et al. visualized Li metal penetration in an SSE using *in situ* and *ex situ* optical microscopy and proposed an electro-chemo-mechanical model of Li filament growth in the SSE.^358^ The results showed that localized Li growth at pre-existing defects can open cracks in the SSE, with Li filling the cracks. Once the interfacial flaws are filled by electrodeposition, the Li filaments build up mechanical stress during further deposition and cause the cracks to propagate.^170,511,728,729^ Kazyak et al. monitored Li filament propagation at the LLZO interface using *operando* optical microscopy and found that different types of morphological features were observed during filament growth.^363^ For a LLZO SSE, even well-polished surfaces can have defects such as grain boundaries and surface ledges that preferentially attract Li deposition.^805^ This indicates that filament propagation can be influenced by multiple factors, which may require a consolidated mechanism reconciling different filamentary growth modes. The SSE grain boundaries neighboring the electrode inherently have different Li transport rates and mechanical properties compared to the bulk SSE material. Simulations by Vishnugopi et al. demonstrated that variations of transport rates at grain boundaries can cause nonuniform Li deposition across an electrode area.^803^ Since the elastic modulus near grain boundaries can be different than the bulk material, this can also influence the mechanical aspects of filament propagation.^44,806^ This suggests that grain boundary engineering in the SSE may be beneficial to suppress morphological instabilities during Li plating.

Another scenario for Li growth within the SSE involves isolated deposition of Li within the SSE due to electronic conduction or related effects. This subsurface degradation has been occasionally observed in various SSEs.^358,363,385,636,807^ Han et al. compared isolated Li deposition in three representative SSEs (LiPON, LLZO, and amorphous Li_3_PS_4_) using *operando* neutron depth profiling and suggested that the relatively high electronic conductivities of LLZO and Li_3_PS_4_ (LLZO: 10^–5^–10^–3^ mS cm^–1^, Li_2_S-P_2_S_5_: 10^–6^–10^–5^ mS cm^–1^, and LiPON: 10^–12^–10^–9^ mS cm^–1^) are responsible for subsurface Li growth.^636^ Kazyak et al. demonstrated through *operando* optical microscopy that Li filaments can initiate from internally grown Li islands and propagate along grain boundaries.^363^ This suggests that grain boundaries may act as electron-conducting pathways to aid filament propagation, though further understanding is needed to fully clarify this mechanism. Electronic conductivity of SSEs is important to minimize to prevent shunt currents and to avoid the possibility of internal Li deposition,^808,809^ but in general, it is likely that Li filament growth governed by SSE fracture and chemo-mechanics is responsible for most observations of degradation under realistic, low-to-moderate overpotential conditions.

#### 5.4.2 Effects of Stack Pressure and Current Density

*Stack pressure*. As discussed earlier, localized Li growth due to current constriction can spall and crack inorganic SSE materials, with current focusing at a filament tip accelerating crack propagation and filament growth. Additionally, stack pressure can promote filament propagation. While moderate stack pressure is often needed to maintain intimate interfacial contact during Li cycling, once cracks form at the SSE interface, the applied stack pressure can drive localized Li flow into these cracks via creep, further propagating the cracks. Doux et al. demonstrated that increasing the stack pressure during Li metal cycling using LPSCl SSEs increased short-circuiting behavior.^311^ Fincher et al. also investigated the influence of stack pressure on Li filament propagation using *operando* optical microscopy (Figure 42b).^175^ The trajectory of the propagating Li filament was deflected in the direction of the compressive load applied to the cell, indicating that stack pressure directly promotes filament propagation. Similarly, a study by Ning et al. identified that higher stack pressure promotes crack propagation due to filament growth.^167^ Li plated under 0.1 MPa stack pressure exhibited longer cyclability without short-circuiting compared to plating under higher stack pressure (7 MPa), even at the relatively high current density of 4 mA cm^–2^. This suggests that while pre-existing cracks near the SSE interface can initiate filament growth, filament propagation and penetration through the SSE are influenced by the magnitude of stack pressure applied.^175^ Thus, although stack pressure helps suppress void formation, stack pressure can promote Li filament and propagation and SSE fracture.

“Critical current density” (CCD) measurements have been widely used within the SSB research community.^27,810^ This measurement involves subjecting a Li metal symmetric cell to sequentially higher current densities until short circuiting is observed. However, this approach can only provide partial information under specific experimental conditions, as cell failure can be influenced by various external factors, such as stack pressure, the amount of exchanged capacity, the counter electrode, and even the experimental setup.^178,316,811−814^ To address this, a number of studies have focused on correlating electrochemical data with other characterization methods to better understand cell failure modes. This strategy has been carried out using *operando* optical microscopy and X-ray CT imaging, as detailed in Section 4.3.^167,175,358,815,816^ For example, Kazyak et al. reported how electrochemical voltage signatures correlate with Li penetration behavior using *operando* optical microscopy analysis (Figure 42c).^363^ Although these techniques can provide interconnected results, the cells used for implementing these combined techniques often differ in physical scale or structure from actual laboratory or commercial cells, potentially leading to different behavior. Therefore, cell designs that closely resemble actual cells may be required to ensure comparability between realistic cell behavior and the characterization results.

### 5.5 Interphase Formation

**Figure 43:**
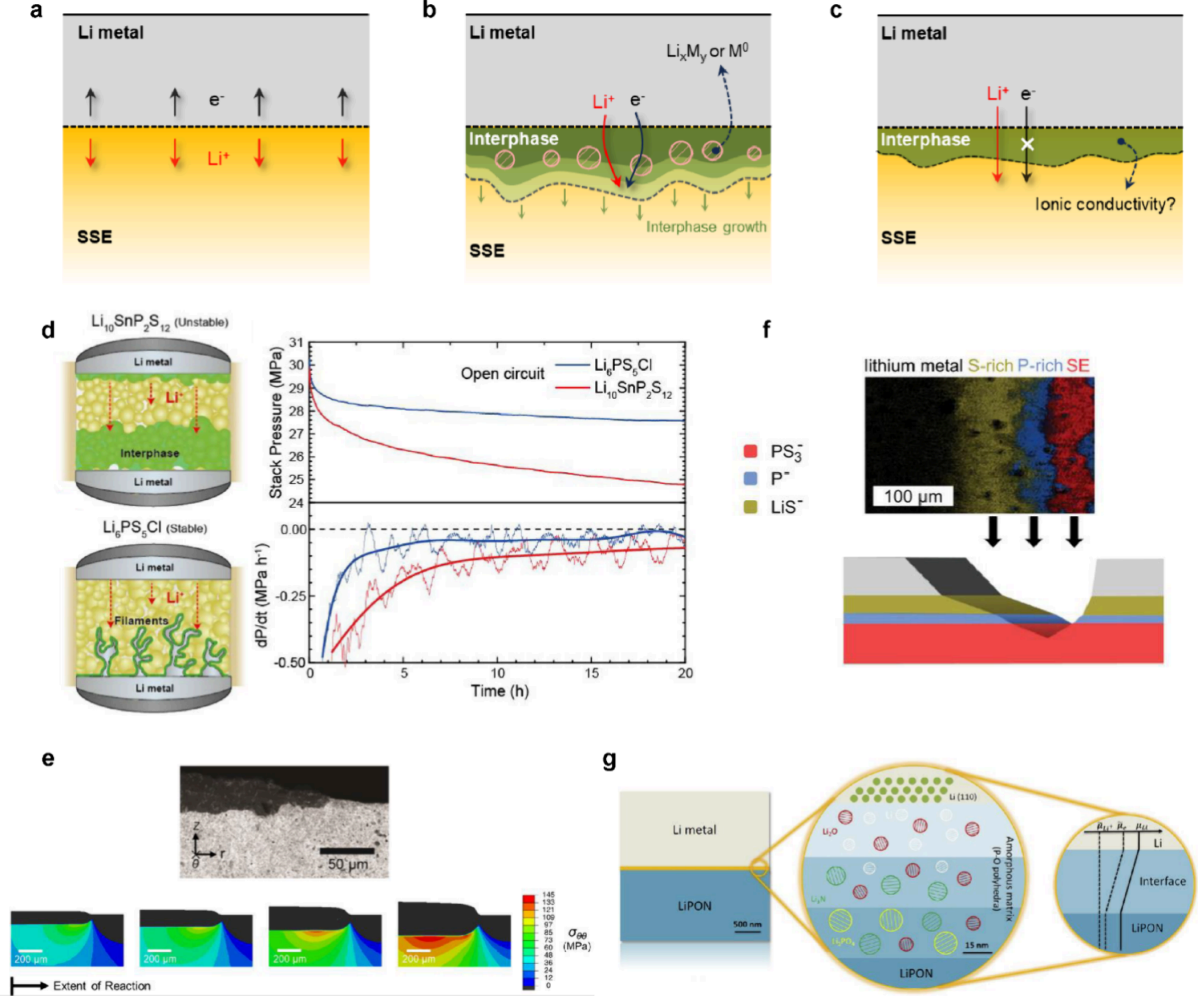
(a–c) Schematic images of three different types of Li metal–SSE interfaces. (a) Thermodynamically stable interface without an interphase layer. (b) Reactive interface with continued interphase growth, which occurs when the interphase is conductive to both Li ions and electrons. (c) Reactive interface with kinetically limited interphase growth. The interface schematics are depicted based on refs (125 and 561). (d) Schematics of different degradation mechanisms of Li_10_SnP_2_S_12_ and LPSCl in contact with Li metal electrodes (left) and stack pressure changes of Li_10_SnP_2_S_12_ and LPSCl symmetric cells under open circuit (right-top panel), along with the time derivatives of the stack pressure (right-bottom panel). Adapted with permission from ref (130). Copyright 2021 American Chemical Society. (e) Finite element analysis of stress evolution within a growing SSE interphase. In the cross-sectional SEM image of LAGP, the darker region is the interphase. The simulated stress evolution at different extent of reaction is shown in the image below. Adapted with permission from ref (293). Copyright 2019 American Chemical Society. (f) ToF-SIMS characterization of a bilayer Li–LPSCl interphase. Reproduced with permission from ref (445). Copyright 2022 Wiley-VCH GmbH. (g) Schematic image of a multilayered Li–LiPON interface, as characterized by cryogenic electron microscopy and XPS. Reproduced with permission from ref (131). Copyright 2020 Elsevier.

At an ideal Li–SSE electrode interface, SSEs would be thermodynamically stable in contact with Li metal, and interphase formation would not occur. Such a Li metal–SSE interface could have low interfacial resistance without sluggish Li transport through an interphase layer (Figure 43a).^125,132^ However, most SSEs are not stable in contact with Li and experience interphase formation through reduction and lithiation reactions by Li metal. Figure 2a (presented previously) shows examples of possible equilibrium phases that can form from typical SSEs. Critically, the reactions to form the interphase can either be self-terminating or continuous, depending on the interphase compounds generated at the interface, as shown in Figure 43b,c.^561^ Typical interphase compounds, such as Li_2_S, Li_2_O, Li_3_P, and LiCl, are generally observed in sulfide- and oxide-type electrolytes and are thermodynamically stable against Li metal (as previously shown in Figure 2a). Their electronic conductivities are extremely low (e.g., <10^–11^ mS cm^–1^ for Li_2_O and Li_2_S), which can block electronic charge transport across the Li–SSE interface.^115,118^ If the produced layer sustains electron transport, however, interphase growth can continue, proceeding into the bulk of the SSE (Figure 43b). SSEs containing metallic cations in their frameworks, such as LGPS, LAGP, LATP, LIC, and LYC, can form electronically conducting reaction products like reduced metal phases or Li alloys, leading to continued growth of the interphase and substantial SSE degradation (Figure 43b).^129,130,561^

If the interphase layer is ionically conductive and electronically insulating, its growth will be slowed after a certain thickness due to kinetic limitation of the interfacial reaction (Figure 43c). SSEs within this class include LLZO, argyrodite-type LPSCl, and LiPON, which can form Li_2_O, Li_2_S, Li_3_P, and Li_3_N when in contact with Li. These interphase compounds suppress unlimited interphase growth because of their low electronic conductivities and thermodynamic stability against Li metal. Additionally, these SSEs do not form other electronically conducting interphase components (such as Li metal alloys), contributing to a kinetically stable interphase. Although a relatively stable interphase may form, the cycling performance may still be affected by the ionic conductivity of the interphase layer, as it influences ion transfer kinetics for reversible redox reactions.^804^ Commonly formed phases like Li_2_O and Li_2_S, which have ionic conductivity less than 10^–5^ mS cm^–1^ (Figure 2b), pose major transport barrier for Li exchange at the SSE interface.^128,817^Figure 2b shows that most interphase materials have been reported to have low ionic conductivities (<10^–4^ mS cm^–1^), while Li_3_N and Li_3_P have higher ionic conductivities than the rest (>0.1 mS cm^–1^). This suggests that these phases could enhance the ionic conductivity of the interphase layer, but little is known about their roles at the Li–SSE interface. Particularly, P is present in many SSEs, but Li_3_P is challenging to observe due to its instability even in glovebox or ultrahigh vacuum (UHV) atmospheres.^817^

These interphase formation behaviors can impact the chemo-mechanical behavior of SSBs in various ways. Lee et al. investigated the evolution of the stack pressure in cells with different electrolytes, Li_10_SnP_2_S_12_ and LPSCl (Figure 43d).^130^ This study demonstrated substantial pressure relaxation in Li_10_SnP_2_S_12_ due to continued interphase growth (Figure 43b), in contrast to the LPSCl case. This behavior was attributed to the significant net volume reduction in the cell caused by interphase formation, which affects morphological stability at the Li–SSE interface. In other work, X-ray CT imaging of the Li metal–LAGP interface revealed the expansion of the SSE itself during interphase formation (Figure 43e),^293^ which caused in-plane stress evolution and fracture of the SSE. These examples highlight the strong correlation between electrochemical/chemical stability of the interface and the evolution of internal pressure, which can critically impact SSB operation.

We next focus on the interphase characteristics of a few SSE materials that have received the most attention for use in batteries.

#### 5.5.1 Li_7_La_3_Zr_2_O_12_ (LLZO)

The interfacial stability of LLZO in contact with Li metal has been theoretically predicted using first-principles calculations.^109,125,818^ Since the stability window of LLZO ends 0.05 V above the Li/Li^+^ redox potential, the LLZO interface is expected to form possible reaction products including Li_2_O, Zr_3_O, and La_2_O_3_ (Figure 2a). However, experiments have revealed that reality is different than these thermodynamic predictions. The chemical structure of the interphase varies sensitively depending on experimental conditions, such as measurement procedures (*in situ* or *operando* measurements vs. air exposure),^56,57,132^ SSE dopants,^819^ and interface preparation methods.^335^ Ma et al. used *in situ* TEM to observe the presence of a tetragonal LLZO interphase layer under vacuum condition, measuring about 6 nm in thickness. This interphase was different than the interphase character after air exposure.^132^ Connell et al. reported that reactivity between LLZO and Li metal, as well as the interphase structure, depend on the energetics of the Li deposition method when depositing on the LLZO surface.^335^ Sharafi et al. demonstrated that the interfacial resistance was affected by surface contaminants (such as Li_2_CO_3_ and LiOH) on LLZO and could be efficiently reduced to 2 Ω cm^2^ by removing them.^56,57^

#### 5.5.2 Li_6_PS_5_X (LPSX)

Typical sulfide SSEs generally show very narrow electrochemical stability windows (Figure 2a), which could be a challenging issue in terms of compatibility with Li metal and with cathode materials. However, argyrodite LPSX (X = Cl and Br) has shown promise due to self-limiting (or at least very slow) growth of the interphase in contact with Li metal. This characteristic, revealed through investigations of the time-dependent evolution of interphase resistance, is critical for its potential as a practical SSE candidate.^820^ Depth-resolved XPS confirmed that the reaction products at the Li–LPSX interface are composed of Li_2_S, Li_3_P, and LiCl or LiBr, aligning with thermodynamic predictions. Otto et al. later found that this interphase forms a ∼250 nm thick layered structure with Li_2_S-rich and Li_3_P/LiCl-rich layers using ToF-SIMS and XPS (Figure 43f).^445^ However, these interphase characteristics sensitively depend on experimental conditions, such as temperature and Li plating current density.^567,821^ Luo et al. demonstrated that the structure of the Li_2_S interphase layer varies with temperature. An interphase composed of more highly crystalline Li_2_S at higher temperatures can increase interfacial side reactions, leading to an increased interfacial resistance.^821^ Narayanan et al. reported that the interphase formation at the Li–LPSCl interface is dependent on current density, as revealed by *in situ* XPS measurements.^567^ Higher current densities tend to involve more Li_3_P in the interphase, potentially reducing the interfacial resistance.

#### 5.5.3 LiPON

LiPON has a low ionic conductivity (∼10^–3^ mS cm^–1^, Figure 2a),^822^ but it features relatively good interfacial stability with Li metal. This stability is evidenced by its extremely stable electrochemical cycling performance, achieving over 10,000 cycles with 99.98% Coulombic efficiency in thin film batteries.^141^ In contact with Li metal, the predicted thermodynamic phases include Li_3_P, Li_3_N, and Li_2_O (Figure 2a). Schwöbel et al. experimentally proved with *in situ* XPS that the interphase is self-limiting and contains Li_3_P, Li_3_N, Li_2_O, and Li_3_PO_4_ species. These species facilitate Li^+^ ion diffusion while blocking electron conduction.^823^ Recently, Cheng et al. investigated the nanoscale structure of this interphase using a combination of cryo-FIB and electron microscopy, revealing layered and gradient distributions of Li_3_N and Li_3_PO_4_ species (Figure 43g).^131^ Despite these insights, the full understanding of the origins of the outstanding cycling stability of LiPON-based cells and its correlation with interphase products remains unclear. It has been suggested that the extremely low electronic conductivity (10^–12^–10^–11^ mS cm^–1^) of LiPON as compared to other electrolytes might be one of the reasons for its exceptional cycling stability.^636^

Thermodynamic analysis of expected interphase products based on DFT calculations has provided valuable insights into interphase formation and characteristics at the anodes in SSBs. However, these examples demonstrate that the actual nature and structure of the interphase often diverge from thermodynamic predictions under practical conditions. Consequently, linking the properties of the interphase to the overall performance of the cells remains challenging. More comprehensive investigations are needed to understand interphase chemistry and structure in the context of SSB cell performance. The buried nature of the Li–SSE interface complicates this task, necessitating the use of innovative characterization techniques.

### 5.6 Interlayers

As discussed, interfacial instabilities and morphology evolution between Li metal and the SSE can result in deleterious effects on battery performance. To address this, engineering strategies involving interfacial modification are being actively pursued. These modifications typically involve adding interlayers at the Li–SSE interface made from various materials, such as metal alloys, Li compounds, or carbon materials.^27,824^ This approach differs from Li composite or alloy foil electrodes, since these interlayers are usually quite thin (micron- or submicron-scale) and are meant to control the growth of Li metal. In contrast, Li alloy electrodes are thicker and act as hosts for Li atoms. Interlayers can stabilize the interface by mediating and promoting contact between Li metal and SSE, leading to homogenized interfacial current densities and reducing interfacial resistance.^26,825^ These approaches can help avoid adverse cell failure arising from direct Li–SSE contact by physically separating the interfaces or by regulating interfacial transport effects.^824^ The following sections highlight a variety of interlayers that have been investigated, along with methods that have been used to characterize their behavior during cycling.

#### 5.6.1 Alloy Interlayers

**Figure 44:**
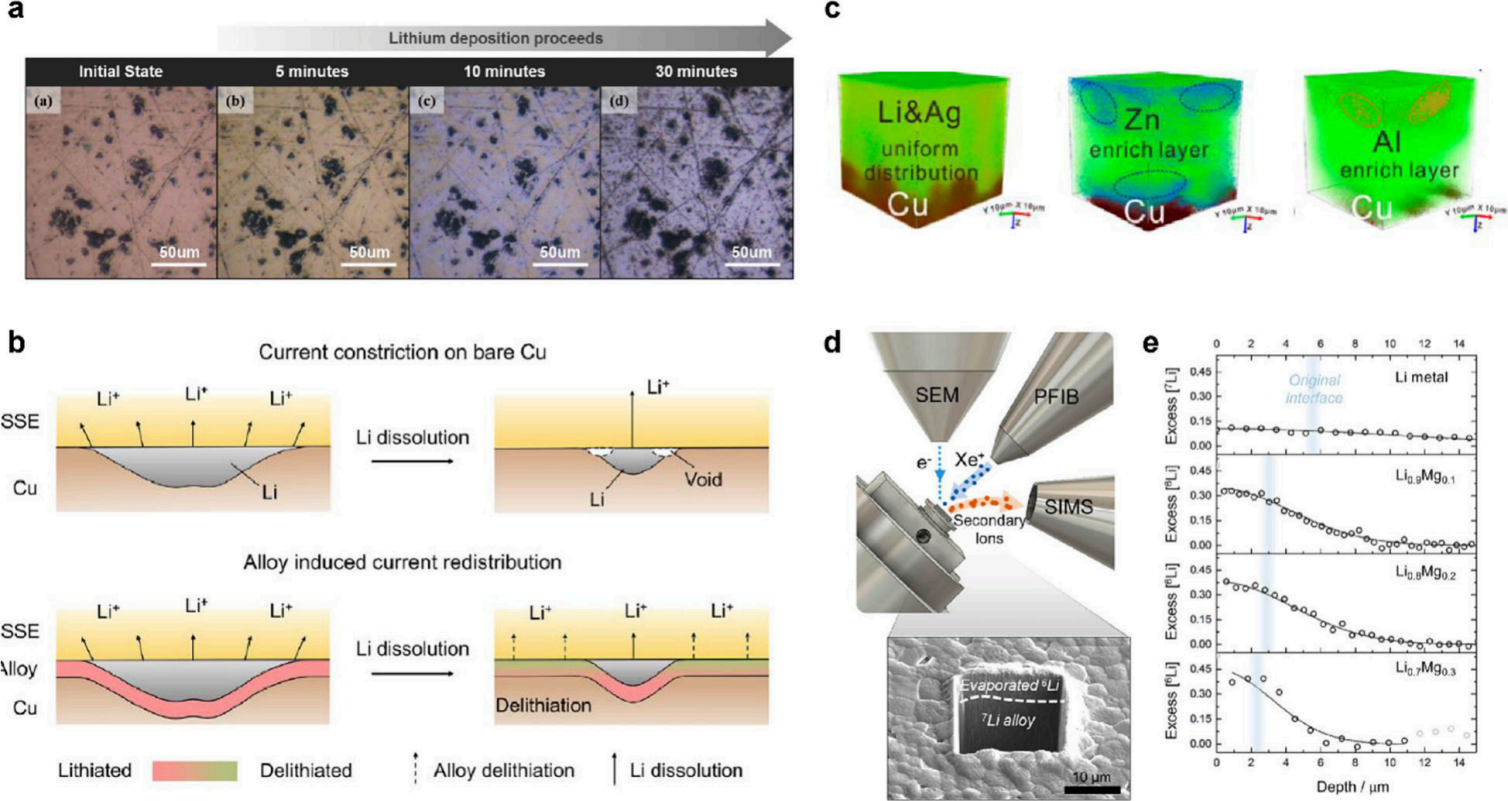
(a) *Operando* optical microcopy images of Li deposition on a 30 nm-thick Au layer sputtered on LLZO. Reproduced with permission from ref (457). Copyright 2020 WILEY-VCH Verlag GmbH & Co. KGaA. (b) Mechanism of Li stripping current homogenization by Li alloy interlayer. The top panel shows stripping from an isolated Li island surrounded by a Cu current collector; current concentration as the Li shrinks leads to void growth. The bottom panel displays a schematic image of stripping from an isolated Li island surrounded by a Li alloy layer. Li is dealloyed from the alloy interlayer as current is concentrated and the local potential increases, possibly mitigating void growth. Reproduced with permission from ref (315). Copyright 2023 Elsevier. (c) Reconstructed 3D subvolumes showing spatial distribution of Li–Ag, Li–Zn, and Li–Al alloys (from left to right) obtained with TOF-SIMS analysis. Adapted with permission from ref (834). Copyright 2023 American Chemical Society. (d, e) Li diffusivity measurement in a Li–Mg alloy by Li isotope tracer methods and using plasma-FIB-SEM and SIMS. (d) Experimental setup and ^6^Li–^7^Li cross-sectional FIB image as an example. (e) Tracer isotope distributions in different Li–Mg alloying conditions monitored by SIMS. (d, e) Reproduced with permission from ref (833). Copyright 2022 American Chemical Society.

Various metallic interlayers have been employed to control nucleation and growth of Li and enhance interfacial stability during Li cycling. A significant challenge arises at the Li metal–LLZO interface due to the lithiophobic nature of the LLZO surface. Interfacial modification with lithiophilic (semi)metallic interlayers has been investigated to alter the nature of the interface.^826−829^ Li alloy interlayers are capable of exchanging Li and electrons simultaneously, thereby lowering interfacial resistance by enhancing contact to Li metal. Krauskopf et al. conducted Li deposition on different metal substrates (Cu and Au) evaporated on LLZO, and analyzed their electrochemical and morphological behaviors using *operando* EIS and *in situ* SEM imaging.^805^ The results revealed that the Au interlayer primarily functions as a buffer layer, reducing the nucleation barrier during the early stages of electrochemical alloying and initial Li deposition. However, dendritic Li growth was observed after the formation of the alloy phase, leading to a loss of morphology control upon Li deposition. Kim et al. reported similar alloying behavior of nanoscale layers of Au, Ag, and Si on LLZO using *operando* optical microscopy (Figure 44a).^457^ As discussed earlier, SSE surface flaws are essentially unavoidable and can cause heterogeneous interfacial Li flux. The optical observations in this study showed that these metallic interlayers can redistribute Li flux, implying that Li diffusion rate within the alloy layer can affect its performance as a buffer layer. Sandoval et al. used FIB and X-ray CT to show that Ag and Au interlayers show the optimal Li nucleation behavior to create uniform Li thickness during growth, and that they improve uniformity during the stripping process as well.^315^*In situ* EIS analysis in this study verified that stripping from the alloy interlayers provided for retention of interfacial contact in contrast to a bare current collector, which exhibited contact loss. The alloy interlayer may reduce current concentrations by selective dealloying in regions where Li is exhausted, thereby mitigating void formation (Figure 44b).^315,830^ Overall, these studies demonstrate the potential benefits of alloy interlayers compared to bare Li metal.

Li alloy materials that form solid solution phases, such as Ag and Mg, may be advantageous as interlayers due to the wide range of Li solubility without significant structural changes.^313,457,830−833^ The different Li alloying behavior comparing solid solution and intermetallic compounds was compared using TOF-SIMS analysis with Ag, Zn, and Al (Figure 44c).^834^ The 3D reconstructed depth profile images demonstrated that the Li–Ag alloy displayed a homogeneous alloy phase, in contrast to the nonuniform alloy phase distribution observed in Zn and Al due to the two-phase nature of these reactions. Jin et al. confirmed the Li dissolution capability of Ag using *in situ* XRD analysis and GITT.^835^ Their study demonstrated fast Li exchange at the alloy-liquid electrolyte interface and high Li diffusivity within the Li–Ag alloy (10^–8^ cm^2^ s^–1^). For Li–Mg alloys, higher Li stripping capacities have been observed compared to bare Li at low stack pressure.^830,833^ This study estimated the effective Li diffusivity in Mg alloys as 1.4 and 2.3 × 10^–11^ cm^2^ s^–1^ for 5 and 10 at. % Mg, respectively, using Sand’s equation. This Li diffusivity was later confirmed through Li isotope distribution analysis using plasma FIB-SEM and SIMS (Figure 44d,e), which found values of 2.4, 2.6, and 1.4 × 10^–11^ cm^2^ s^–1^ for 10, 20, and 30 at. % Mg alloys, respectively.^833,836^ These analyses demonstrated that the Li diffusivity in Mg alloy is similar or lower than Li self-diffusivity (∼10^–10^ cm^2^ s^–1^),^792−794^ and it is also lower than in the Li–Ag alloy. This kinetics difference was linked to Li precipitation after alloying for Ag and Mg alloys, but further research is needed to fully understand this mechanism.^832,837^

There are several unresolved scientific questions and technical challenges related to the use of interlayers featuring metal alloys. One major challenge is the repeated volume changes and corresponding chemo-mechanical fatigue of the interlayer during long-term cycling.^824^ Typical Li alloying materials can experience significant volume changes (100–300%),^5^ leading to chemo-mechanical issues such as pulverization or delamination from the SSE after cycling.^138,332,824^ This can cause complex evolution of layer morphology and integration of the layer into the growing Li during Li deposition.^289,315^ Such behavior has frequently been observed in complex interlayer structures and is typically investigated using cross-sectional imaging techniques, such as cryogenic or plasma FIB-SEM combined with EDS or SIMS.^313,315,831^ However, these are generally destructive and only applicable *ex situ*, and they provide limited chemical information. Therefore, advanced techniques that monitor the spatiotemporal changes of nanoscale interlayers *operando* are urgently required to fully understand their dynamic evolution and influence on electrochemical behavior.

#### 5.6.2 Other Interlayer Materials

Interfacial engineering using Li compounds, such as Li_2_O, LiF, Li_3_N, Li_3_P, and Li_2_Te, has been found to be effective for promoting reversible electrochemical cycling. Unlike alloy interlayers which react with Li and transport both electrons and Li atoms, most Li compounds are electronically insulating and thermodynamically stable in contact with Li metal (potential windows are shown in Figure 2b).^113,117,838^ These features enable them to suppress SSE degradation by potentially preventing interphase reactions.^839−842^ For instance, the electronic conductivities of LiF-coated SSEs at various potentials and temperatures were evaluated using the electrochemical DC polarization method.^841^ The electronic leakage current of the LiF-coated interface was 1 order of magnitude lower than the pristine SSE interface. Li wettability on the SSE surface can also be increased via these interlayers.^840,843−846^ DFT has shown that interfacial Li wettability depends on the Li compound species present at the interface as follows: Li_2_O > LiPON > Li_2_CO_3_ > LiF.^847^ The study suggested that controlling the chemistry of the interface may minimize morphological irregularities, which could increase the current density attained for stable stripping.

Nitrogen-based compounds have also been investigated as interlayers. The effects of a LiPON interlayer coated on LPSCl were investigated by Su et al.^845^ Enhanced Li wettability of the LiPON interlayer was verified with FIB-SEM imaging, which was correlated with the improved Li cyclability in symmetric cells. A recent report by Cheng et al. characterized the Li–LiPON interface using cryogenic TEM, which was revealed as Li_2_O/Li_3_N/Li_3_PO_4_ multilayered mosaic structure (Figure 43g).^131^ Considering the Li wettability order,^847^ the Li_2_O phases distributed in the outermost layer (contacting Li metal) also might help to improve the morphological stability during cycling. The study also suggested that the superior ionic conductivity of Li_3_N may contribute to the outstanding stability of the Li–LiPON interface.^847,848^ Li_3_N and Li_3_P exhibit exceptionally high ionic conductivity compared to other phases (>0.1 mS cm^–1^, Figure 2b). Interfacial modification using these materials has been reported as a promising approach for stable Li cycling by drastically reducing the interfacial resistance.^849−851^

As an alternative to using Li compounds, interlayers made from other metal compounds have also been studied.^466,843,844,852−855^ These metal compounds, generally having the chemical structure MX (where M and X are non-Li metal cation and counteranion, respectively) can be converted into the reduced metal (M^0^) and the Li compounds (LiX) during an initial conversion reaction.^852^ Lee et al. demonstrated the conversion process of an AgF interlayer after Li plating using XPS and XRD, finding that AgF was reduced to form LiF and the Li_9_Ag_4_ phase. This Li–Ag phase served as a buffer layer for Li cycling.^852^ These findings highlight the potentially synergistic effects of conversion materials that form more complex mixtures of phases when reacted with Li.

### 5.7 Li Hosts and Composite Structures

3D structured electrodes containing Li metal that can conduct Li and electrons through their frameworks have been highlighted as a possible strategy to avoid morphological instabilities at the Li–SSE interface. This approach has been heavily investigated in liquid electrolyte systems,^856,857^ where the expanded surface area and infiltration of the liquid electrolyte into the framework promote reaction reversibility. However, this approach poses different challenges when applied to SSE systems. Unlike the liquid electrolyte, the SSE cannot spontaneously infiltrate into these complex structures. Therefore, the 3D framework must be conductive to both Li^+^ ions (or Li atoms) and electrons to facilitate the Li redox reaction across its surface area. If the conductivity or transport pathways are insufficient to deliver Li into the deeper areas of the 3D structure, most of the deposited Li may accumulate near the SSE interface, resulting in the same morphological instabilities observed when using Li alone. However, if Li can be reversibly hosted within the hollow spaces of the 3D framework and the framework remains in contact with the SSE, this can suppress interfacial instabilities. This section introduces and discusses the characterization and understanding of Li composite structures and their operational mechanisms.

#### 5.7.1 Diffusion Pathways

As mentioned in Section 5.3, Li self-diffusivity (∼10^–10^ cm^2^ s^–1^)^792−794^ is not high enough to prevent voiding in pure Li electrodes under practical battery cycling rates. However, Li can exhibit enhanced diffusion rates at heterogeneous interfaces, such as graphitic surfaces or within Li alloys. The Li diffusivity on graphitic surfaces is reported to be significantly higher than in bulk Li, approximately ∼10^–7^–10^–6^ cm^2^ s^–1^ and 5 × 10^–6^ cm^2^ s^–1^ for Li ions and Li atoms, respectively.^858,859^ Similarly, Li diffusivities in alloy materials, such as Li_*x*_Ag or LiIn, can also surpass Li self-diffusivity.^5,835^ For instance, Li diffusivity in LiIn is as high as 10^–6^ cm^2^ s^–1^.^860^ Thus, shifting Li transport pathways away from bulk self-diffusion to diffusion along heterogeneous surfaces or within alloy materials may enhance Li kinetics at the SSE interface. As an example, carbon materials such as CNTs, graphene oxide, graphite, and carbon black have been utilized as interfacial Li diffusion mediators or Li host structures to achieve stable electrochemical cycling.^288,313,824,861,862^

**Figure 45:**
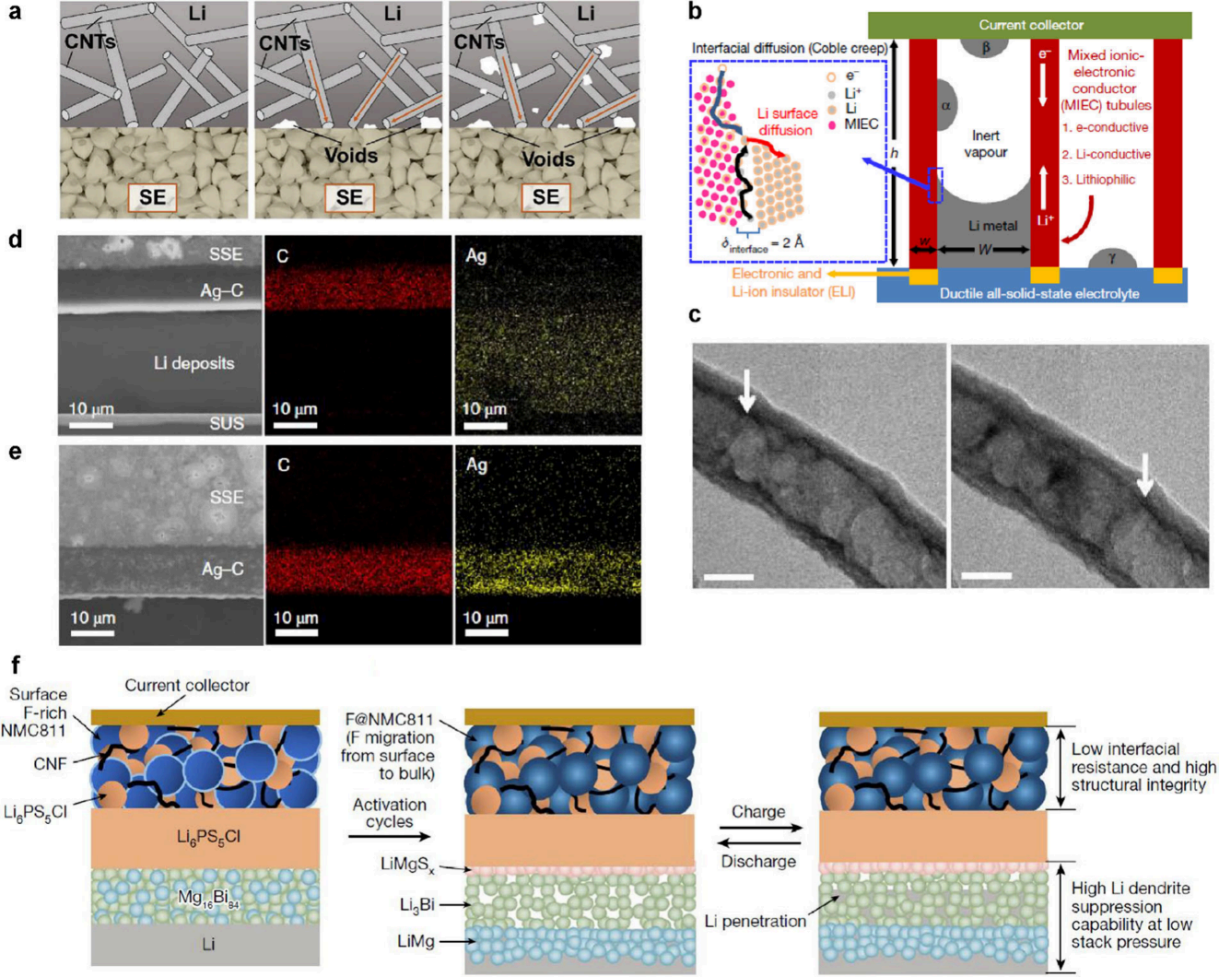
(a) Schematic images of Li stripping from a Li–CNT composite electrode, during which the nanotubes retain interfacial contact and deliver Li to the interface. Reproduced with permission from ref (861). Copyright 2022 Wiley-VCH-GmbH. (b, c) Hosting of Li in carbon nanotubes. (b) Schematic depiction of diffusional creep-driven Li plating and stripping in nanotubes. (c) *In situ* TEM images of Li growth inside a nanotube. Li filling fronts in the carbon tubule are marked with white arrows. The scale bar is 100 nm. (b, c) Adapted with permission from ref (288). Copyright 2020 Springer Nature. (d, e) SEM cross-sectional images of reversible Li (d) charging and (e) discharging at a Ag–C nanocomposite 3D interlayer and corresponding Ag and C elemental mapping images from EDS. (d, e) Reproduced with permission from ref (313). Copyright 2020 Springer Nature. (f) Schematic operating mechanism of the Mg_16_Bi_84_ interlayer at the Li–LPSCl interface, which transforms into LPSCl/LiMgS_*x*_/Li_3_Bi/LiMg by spontaneous lithiation and interfacial chemical reaction with LPSCl. The LiMg alloy formed *in situ* acts as a lithiophilic agent guiding the Li deposition away from LPSCl, while the LiMgS_*x*_ layer acts as an interlayer–LPSCl interphase preventing LPSCl reduction and Li dendrite growth. Reproduced with permission from ref (831). Copyright 2023 Springer Nature.

Fuchs et al. demonstrated that a Li–CNT composite enhances Li dissolution kinetics due to the structural effect of embedded carbon nanotubes (Figure 45a).^861^ During Li stripping under negligible stack pressure, the composite interface transforms from a 2D plane to a 3D scaffold, with nanotubes remaining and contact with the SSE and supporting Li delivery to the interface. Similarly, Li dissolution kinetics were enhanced in a Li-reduced graphene oxide composite.^862^ By using *in situ* EIS and *ex situ* FIB-SEM imaging, this work verified that the accumulation of carbon at the SSE interface kept interfacial impedance lower than that of a pure Li electrode during stripping. This suggests that the carbon scaffold accumulated at the interface can mediate Li delivery even when there is a disconnection between the Li reservoir and the SSE, suppressing interfacial void growth. Additionally, the accumulation of carbon was advantageous for homogenizing the current at the interface during deposition, enabling reversible Li cycling at low stack pressures.^862^ However, it was also revealed that carbon materials exhibit structure-dependent Li kinetics, indicating a need for more thorough investigations to understand this transport mechanism.^824^ A similar study was conducted with a Li–Mg alloy electrode, where diffusion kinetics was found to be governed by Li chemical diffusion in the Mg alloy. Interestingly, this alloy system exhibited lower Li stripping capacities than the CNT scaffold.^830^ Although a rigorous comparison is needed, this result suggests that carbon materials have superior capabilities as interfacial transport mediators.

#### 5.7.2 Host Structures

Li metal deposition into a 3D host structure is considered to be a promising strategy to buffer cell-level volume changes and internal stress evolution, perhaps reducing the risk of morphological instabilities.^863^ As mentioned earlier, the efficacy of hosting deposited Li depends on the transport properties of the scaffold (or interlayer), and various approaches have been taken.

Chen et al. demonstrated Li metal hosting inside carbon tubular structures, which was investigated with *in situ* TEM, as shown in Figure 45b,c.^288^ Since the carbon tubules are in thermodynamic equilibrium with the deposited Li and function as a mixed ionic-electronic conductor (MIEC), they can guide the deposited Li to enable plastic flow within the confined space through a diffusional creep mechanism (Coble creep, Figure 45b). Depositing Li into the prearranged pore space can minimize mechanical stress applied on the SSE. The study also predicted that Li alloy materials, such as Li_22_Si_5_ and Li_9_Al_4_, can serve as MIEC scaffolds enabling Coble creep due to their higher Li diffusivity at the Li interface than in their bulk.^288,864^

A similar strategy was demonstrated using a Ag–C nanocomposite 3D interlayer in an anode-free architecture.^313^ This study showed that incorporating Ag nanoparticles lowered the Li nucleation barrier within the porous carbon black scaffold, leading to preferential Li deposition at the current collector-carbon scaffold interface rather than at the carbon scaffold–SSE interface. Cross-sectional SEM and EDS imaging during Li deposition and dissolution (Figure 45d,e) revealed complex movement of the Ag seeds within the deposited Li and carbon scaffold, indicating that the Ag seeds facilitate preferential deposition beyond the carbon scaffold. This feature helps prevent mechanical damage to the SSE by spatially separating it from the deposited Li layer. However, the study left several questions unanswered about governing mechanisms, particularly the synergistic effect of the carbon scaffold and Ag nanoparticles. Kim et al. discussed the thermodynamic contributions of Ag nanoparticles to Li deposition behavior and the potential of Ag to enable long-range transport.^865^ The role of the adhesion energy across the current collector–C scaffold–SSE interface was also emphasized.^866,867^ This study revealed that higher adhesion energy at the C scaffold–SSE interface compared to the current collector-carbon scaffold interface favored Li deposition over the C scaffold, as shown by *ex situ* SEM characterization. As a proof-of-concept, a Ag interlayer was introduced at the carbon scaffold–SSE interface to enhance interfacial adhesion, resulting in excellent Li cyclability (∼150 cycles) in a 2 × 2 cm^2^ pouch cell without external pressure.^867^

Li hosting approaches have also been implemented using a garnet-type 3D porous SSE.^855,868,869^ A bilayer structure was constructed, comprising 3D porous and planar dense SSEs, which served as the Li hosting structure and separator, respectively. The porous SSE accommodated deposited Li in its confined space, reducing the risk of interfacial instabilities by expanding the SSE surface area and reducing local current densities. However, additional surface modifications are seemingly required, including zinc oxide, CNT, or amorphous carbon coatings, to reduce the intrinsic lithiophobicity and enhance the electronic conductivity of the garnet SSE as a MIEC.^855,868^ Recently, Alexander et al. devised a cation doping method for the porous garnet SSE to increase its electronic conductivity (10^–2^ mS cm^–1^ in Cr-doped SSE), thereby improving electron support for Li deposition within the porous SSE.^869^ This study demonstrated outstanding Li cyclabilities, achieving current densities up to 60 mA cm^–2^ with 30 mAh cm^–2^ capacity.

Another study proposed guidelines for designing Li host structures with anode-free architectures as follows:^870^ (1) the Li host structure should have moderate electronic conductivity and high ionic conductivity to facilitate Li deposition within the hosting framework. (2) The Li host structure should be lithiophobic to enable facile and reversible Li deposition and extraction, but (3) the region near the current collector needs to be lithophilic to guide uniform Li deposition near the current collector, effectively separating the deposited Li from the SSE. Based on these guidelines, a Li hosting framework made of a Li_3_N and Li–Bi alloy decorated LiF–carbon scaffold was implemented.^870^ The LiF–C scaffold served as a lithiophobic framework having moderate ionic and electronic conductivities (10^–2^ and 3.4 × 10^–4^ mS cm^–2^, respectively). ToF-SIM analysis verified the spatial distribution of Li_3_N and Li–Bi alloy particles within the C scaffold.^831,870^

Recently, an interlayer consisting of Li–Bi and Li–Mg alloys was implemented, demonstrating an *in situ* evolved LiMgS_*x*_-Li_3_Bi-LiMg structure capable of hosting Li successfully (Figure 45f).^831^ The Li–Mg alloy served as a lithiophilic agent to facilitate Li deposition within the Li_3_Bi alloy layer, which functioned as a Li hosting structure. While the relatively high electronic conductivity of the Li_3_Bi alloy interlayer may degrade the SSE (in this case, LPSCl was used as SSE), the *in situ* formed LiMgS_*x*_ interphase, positioned between the Li–Bi alloy and LPSCl, was found to minimize reduction of LPSCl while suppressing Li dendrite growth.^831,871^ Spatial evolution of the Mg alloy phases (LiMgS_*x*_ and LiMg) was analyzed using ToF-SIMS and SEM-EDS cross-sectional imaging. The LiMgS_*x*_ interphase in this study aligns with the requirement of an additional passivating layer to protect SSE ionically and electronically from MIEC, as suggested by Wang et al.^863^

The Li host structures introduced in these studies all involve efforts to chemically or mechanically guide Li deposition within confined spaces, which often entails the use of 3D ion/electron conducting networks or, in some cases, complex structural changes during initial lithiation. However, the mechanisms governing Li growth and dissolution within these structures are still debated, particularly regarding the transport kinetics of involved species such as atomic Li, Li^+^ ions, and electrons. The corresponding details of deposition and dissolution modes, as well as the structural and interfacial properties of the host layers, are not well understood.^27,288,865,866,872^ Understanding the hosting mechanisms during charge and discharge likely necessitates multifaceted characterization strategies, including assessments of the ionic/electronic transport capabilities of the interlayer components^831,870^ and examinations of structural and interfacial properties (e.g., porosity and lithiophilicity/phobicity),^866,867^ as well as spatiotemporal imaging of Li deposition/dissolution behaviors. Thus, more work remains to be done in characterizing the evolution of interlayers and host structures for Li metal electrodes in SSBs.

## 6 Alloy Anodes

This section discusses the characterization and mechanistic understanding of alloy anode materials as candidates for SSBs with high energy density. Section 6.1 focuses on the variety of alloy anode materials available and their fundamental properties. Section 6.2 discusses our emerging understanding of the reasons why SSB environments can offer improved performance of alloy materials compared to liquid electrolytes. The remainder of the section (Sections 6.3–6.5) focuses on our current understanding of alloy anode behavior that has been enabled by various characterization methodologies.

### 6.1 Overview of Alloy Anode Materials

Materials that electrochemically alloy with Li to form Li-rich compounds can exhibit high specific and volumetric capacities (up to 10 times higher than graphite).^873^ Alloy anodes, such as Si and Al, were investigated early in Li-ion battery research and development (R&D), but they exhibited poor safety and short cycle life in liquid electrolytes. Research on these materials has continued in the intervening decades to attempt to improve their electrochemical performance in Li-ion batteries, and they are one of the prime candidates to enhance energy density and specific energy beyond conventional limits. Alloy anodes, and especially Si, are currently being commercialized for Li-ion batteries, and they are under intense R&D focus for SSB technologies.

Alloys feature several advantages compared to graphite anodes and other candidate anode materials. They have the potential to easily be integrated into existing battery manufacturing processes, such as slurry casting. Some alloys can be rolled into metal foils and may be able to be used directly as foil anodes, increasing energy density and potentially removing the need for metal current collectors. Unlike Li metal batteries, manufacturing can occur with the cell in a discharged state, minimizing safety concerns.

**Figure 46:**
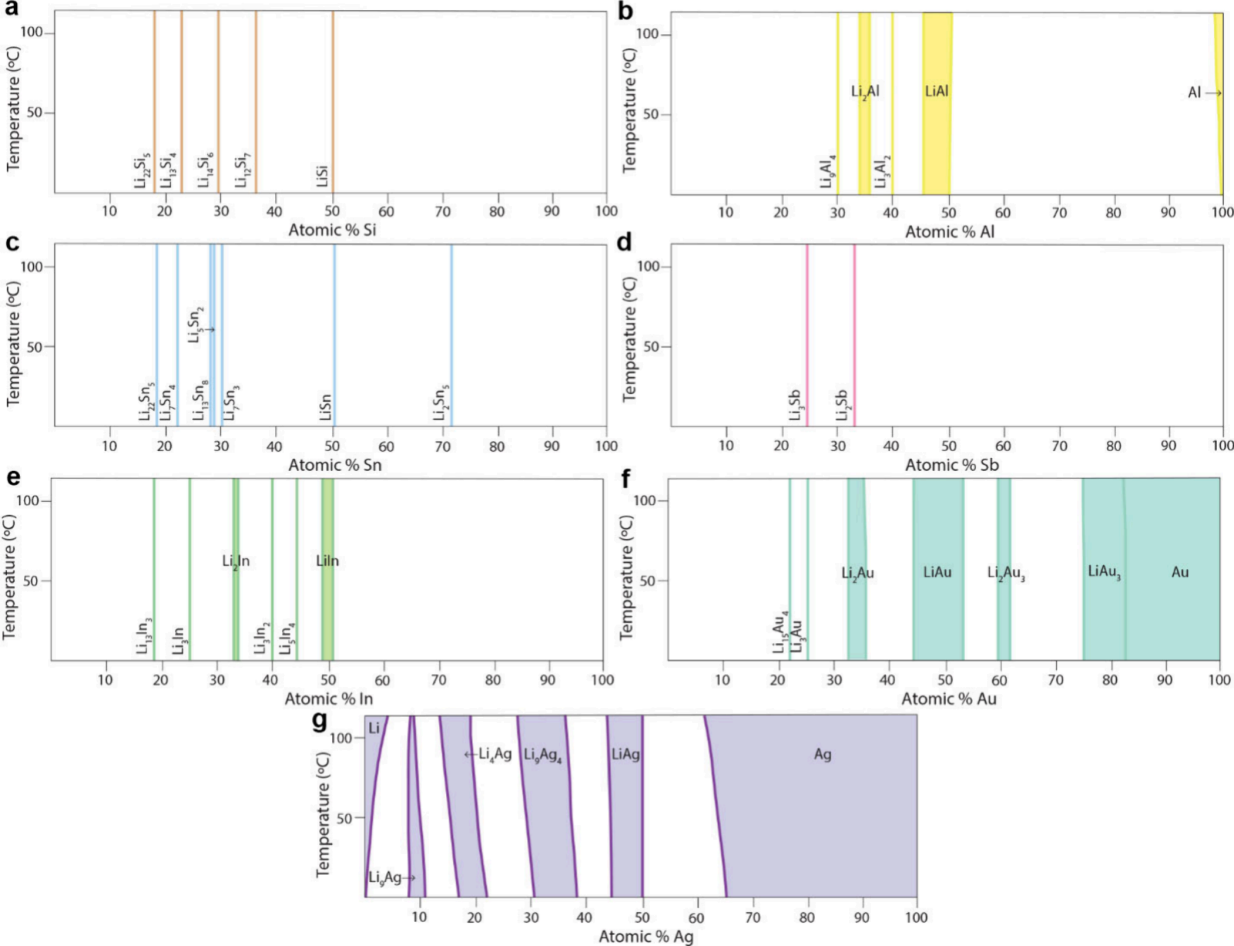
Phase diagrams across a limited temperature range for alloy anode materials of interest. Phase diagrams for (a) Li–Si (modified from^950^), (b) Li–Al (modified from^951^), (c) Li–Sn (modified from^952^), (d) Li–Sb (modified from^953^), (e) Li–In (modified from^954^), (f) Li–Au (modified from^955^), and (g) Li–Ag (modified from^955^).

Electrochemical alloying reactions generally fall into two categories: solid solution reactions and two-phase reactions. While both reactions generally follow the form *xM* + *yLi*^+^*ye*^–^ → *M*_*x*_*Li*_*y*_, the phase transformation mechanism differs based on the solubility of Li within the host material.^874^ When Li is miscible with the host phase, the reaction occurs via a solid-solution mechanism. When there is no miscibility between the host phase and Li, new intermetallic phases nucleate and grow.^2,873^Figure 46 is a compilation of the relevant portions of phase diagrams for various alloy materials and Li, showing that some form compounds and some form solid solutions.

Alloying reactions generally allow for higher Li contents to be accommodated within a host material compared to intercalation materials. For example, based on the intermetallic phases in Figure 46a, Si can accommodate 4.4 Li ions per Si atom in its fully lithiated form, while graphite can only accommodate one Li atom per six C atoms. This property allows for high specific and gravimetric capacities of alloys.

While alloying reactions allow for high Li storage capacity, these high capacities also cause extreme volume change. Si expands over 300% at full lithiation, whereas graphite only expands about 10%.^873^ Repetitive volume changes during long-term cycling can result in capacity loss through mechanical pulverization, loss of active Li to side reactions (such as SEI formation), Li trapping, and aggregation of the active material. Pulverized particles can lose contact with the electrode matrix or the SSE, becoming electrochemically inactive.^875,876^ Volume change can also cause fracture of the SEI layer in liquid electrolytes. This causes new active material to be exposed to the liquid electrolyte, and new SEI forms continuously and further consumes the electrolyte.^260,877^ Li trapping, another degradation mechanism, occurs when the Li alloy phase is surrounded by a different phase (such as the pure delithiated phase) with poor Li diffusivity,^878,879^ which reduces reversible capacity and CE. Aggregation of active particles can also occur throughout cycling, increasing diffusion lengths and hindering complete lithiation and delithiation.^877,880^ While these phenomena can occur in both liquid-electrolyte batteries and SSBs, there are different mechanisms involved in the two systems, as described subsequently in Section 6.2. Furthermore, different alloy materials exhibit different properties and degradation behavior. To set the stage for further discussion for characterization of alloy evolution mechanisms, an overview of the general reaction and phase behavior of various alloy materials is provided below.

#### 6.1.1 Silicon

Silicon has received the most research attention of all alloys due to its achievable specific capacity of 3579 mAh g^–1^ based on the fully lithiated Li_15_Si_4_ phase, as well as its abundance, lack of health and environmental concerns, and low potential (∼0.4 V vs Li/Li^+^).^2,873,881^ Si is currently included at low fractions in commercial graphite electrode composites for Li-ion batteries, with significant commercialization effort dedicated to increasing Si content (and thus battery energy density).^882−884^

The first lithiation of crystalline Si occurs via a two-phase reaction to form an amorphous Li-rich phase,^515,590^ bypassing the crystalline phases shown on the phase diagram due to kinetic limitations at room temperature (Figure 46a), as has been shown by *in situ* TEM, NMR, and XRD. At 50 mV vs Li/Li^+^, the Si–Li alloy crystallizes into the cubic intermetallic Li_15_Si_4_ phase,^885,886^ which is metastable but is the most highly lithiated phase in practical use.^887^ The 300% volume expansion during lithiation can cause fracture of particles, observed both with microscopy and through techniques such as acoustic emission.^888^ The delithiation process results in the formation of an amorphous Si phase, generally with low initial CE, with some crystalline Li_15_Li_4_ remaining. The following lithiation cycles follow a solid-solution reaction mechanism, with a sloping potential profile compared to the flat plateau typically seen in the first cycle.^889^

Because of the large volume changes and mechanical fracture which exposes new surface area, Si anodes often exhibit low CE in liquid cells due to continuous SEI formation. Significant research efforts have been dedicated to understanding and controlling the processes that result in the poor performance of Si in liquid-electrolyte batteries. Nanoscale particles of Si have been shown to reduce pulverization of Si and even entirely prevent fracture on the first cycle; however, these nanoscale materials have high surface area and exacerbate SEI formation.^890−892^ Other methods aim to accommodate volume changes though the integration of porosity and void spaces within the electrode architecture, which can increase cycle life of Si-based batteries but lead to reduction of volumetric energy density.^893,894^ Coatings and protective layers have also been used to control the growth and degradation of the SEI.^895−897^

#### 6.1.2 Aluminum

Aluminum alloy anodes were among the first to be considered as a replacement for Li metal early in Li-ion battery development.^898,899^ Although this material showed poor cycle life at that time, research interest has been renewed in recent years because of the high capacity of Al (993 mAh g^–1^), its low potential, and its highest earth abundance of any metal.^900,901^ Additionally, Al mining/refining, processing, and manufacturing are well established in industry, potentially simplifying widespread integration of Al alloy anodes.

*In situ* neutron depth profiling and XRD have shown that lithiation of Al occurs via a two-phase reaction mechanism in which the β-LiAl phase nucleates and grows to consume the α-Al phase, as shown by the plateau at ∼0.3 V vs Li/Li^+^.^899,902^ Initial lithiation also results in the lithiation of the aluminum oxide surface layer, sometimes leading to large transient overpotentials.^903^ During delithiation, the LiAl phase contracts as Li is removed to reform the pure Al phase, shown by *operando* optical microscopy, and this contraction can cause cracks within the LiAl domains.^904^ Despite the existence of thermodynamically favorable intermetallic phases with higher Li content than LiAl (Figure 46b), these phases tend not to form electrochemically at room temperature under practical currents.^905,906^

Unlike other alloys, Al anodes are commonly used as metal foils instead of particulate composites, which increases electrode-level volumetric capacity.^907^ However, without the electrode matrix to accommodate volume changes, degradation can be rapid.^908,909^ Alloying Al with other metals has been shown to increase the lifetime of Al foil anodes by altering the distribution of stress within the foil.^900^ Al anodes have also been shown to be particularly susceptible to Li trapping due to differences of the Li diffusivity between the reacted and unreacted phases.^878,910^ Creating multiphase foils with Al and other metals can enhance Li transport and reduce trapping effects.^908^

#### 6.1.3 Tin

Tin anodes exhibit a high theoretical specific capacity of 993 mAh g^–1^ and a volumetric capacity of 2020 mAh cm^–3^ at full lithiation to the Li_22_Sn_5_ phase.^136^ Sn-based materials have been integrated with some success into commercial batteries produced by Sony.^911^ Sn has a higher reaction potential (multiple plateaus between 0.8 and 0.2 V vs Li/Li^+^) compared to graphite and Si, which reduces battery voltage.^912^ Sn-based electrodes can be manufactured either as slurry cast electrodes^913^ or as foil electrodes.^2^

Lithiation of Sn occurs through a multistep reaction in which multiple intermetallic Sn–Li phases are formed. The terminal crystalline phase is generally reported to be the monoclinic Li_7_Sn_3_ phase, with intermediate phases of tetragonal Li_2_Sn_5_ and monoclinic LiSn forming, following the Sn–Li phase diagram (Figure 46c).^912,914^ Formation of the thermodynamically predicted Li_22_Sn_5_ phase is usually not experimentally observed with XRD in micron-scale particles at room temperature, which has been suggested to be due to large crystal structure differences inhibiting the transformation.^912^ Instead, further lithiation past the Li_7_Sn_3_ phase may result in an amorphous Li-rich phase.^912,915^ Reaction pathways have been shown to be dependent on currents and particle sizes. At relatively high current, the multistep phase transformations are not present and only one reaction occurs, transforming tetragonal Sn into the monoclinic Li_7_Sn_3_ phase.^914^ At lower currents, the multistep phase transformation is present. Micron-scale Sn particles undergo lithiation-induced fracture, followed by pulverization upon further cycling. Nanoscale Sn has been observed to undergo a two-step transformation to an amorphous phase before forming the Li_22_Sn_5_ phase with *in situ* TEM.^916,917^ The reverse pathway is usually followed on delithiation.^916^ Morphological changes of the Sn anode are also dependent on particle size, with large particles forming porous structures after delithiation, while nanoscale particles remain dense, leading to improved cycling stability of smaller particles.^918^

Micron-scale Sn particles integrated into traditional slurry cast electrodes tend to exhibit poor cycle life and fail within 50 cycles in liquid-electrolyte batteries, likely due to the large volume change on lithiation and delithiation.^919,920^ The use of Sn intermetallic alloys and carbon-based microstructures has been investigated for improving performance. Sn can be initially alloyed with other metals that are electrochemically inactive, like Ni or Co, and these nonreactive components can help accommodate the volume changes and associated stresses.^921,922^ Integrating Sn within carbon structures also results in better particle stability and longer cycle life.

#### 6.1.4 Antimony

Antimony has received less attention as a Li alloy anode, partially due to its lower specific capacity (660 mAh g^–1^) and higher reaction potential (0.9 V vs Li/Li^+^) compared to other alloy materials, resulting in reduced specific energy.^873^ It is also toxic and presents an environmental challenge. Despite these negative features, Sb is of interest because it shows remarkably stable cycling at room temperature in liquid-electrolyte batteries,^920,923^ which may partly be due to its lower volume expansion (135%).^924^ Sb is brittle and is usually studied as a particulate composite electrode.

Investigations of the lithiation pathway of Sb through NMR and XRD have shown that it largely follows the intermetallic Sb–Li phases on the phase diagram (Figure 46d), with some minor differences. The intermediate hexagonal Li_2_Sb phase is first formed, with further lithiation forming the cubic Li_3_Sb phase.^925,926^ This cubic Li_3_Sb phase is a high-temperature polymorph which typically forms above 650 °C, rather than the α-Li_3_Sb phase expected at room temperature.^924^ Diffusion of Li within the pure rhombohedral Sb phase occurs through Li hopping between interstitial sites, while the two intermediate compounds exhibit vacancy diffusion, and both can accommodate high Li vacancy concentrations.^927^ On delithiation, Li_3_Sb converts directly to pure Sb,^924,926,927^ skipping the intermediate phase seen during lithiation due to kinetic hindrance of Li_2_Sb nucleation.^927^

Despite its large volume change compared to graphite (135% vs 10%), Sb has been shown to exhibit good cycling stability compared to other alloys in liquid-electrolyte batteries. Sb nanoparticles have shown stability,^928^ and Boebinger et al. found that smaller particles were able to form internal voids on delithiation as the external oxide layer was able to withstand buckling, while the larger particles buckled on delithiation.^923^ Combining Sb with carbon materials can help accommodate volume changes and extend cycle life of Sb-based batteries.^929,930^

#### 6.1.5 Indium

Indium is generally not used as an alloy anode in liquid-electrolyte batteries due to electrolyte instabilities. It is very commonly used in SSB research as a counter electrode due to its high CE, high Li diffusivity, and good reversibility in conjunction with certain SSEs. In exhibits a high theoretical capacity of 1012 mAh g^–1^, but it is expensive and not as abundant as other alloy choices.^2^

Indium is lithiated via two-phase reactions with plateaus at 0.6 and 0.03 V vs Li/Li^+^,^931^ corresponding to the formation of crystalline LiIn and Li_13_In_3_ (Figure 46e). There is a sloping potential below 0.25 V vs Li/Li^+^, which arises from Li solubility in the LiIn phase.^931^ The two crystalline phases observed through XRD appear on the phase diagram, which also includes other phases which do not typically form electrochemically. The final plateau results in the simultaneous formation of Li_2_In and Li_13_In_3_,^931,932^ although by the end of the plateau only Li_13_In_3_ remains. There are three plateaus on delithiation at 0.09, 0.12, and 0.25 V vs Li/Li^+^. The first two plateaus are affiliated with the conversion of Li_13_In_3_ into Li_2_In, with the distinct plateaus possibly relating to Li removal from different crystallographic sites.^931^ The plateau at 0.25 V vs Li/Li^+^ occurs during the formation of Li_3_In_2_, with higher-potential plateaus then occurring to transform to LiIn and then to pure In.

Indium has been used within batteries in a variety of contexts. The In–Sb intermetallic phase has been investigated as an anode for Li-ion batteries. A few studies have found that this intermetallic phase can exhibit high specific capacity and relatively reversible cycling,^933,934^ while others have questioned its stability.^935^ Covalent In compounds can also be formed with elements like O,^936^ and P,^937^ demonstrating high capacity, low overpotential, and improved cycling stability. In is also frequently used as an interlayer to control the deposition of Li in both liquid^938,939^ and solid-state^940,941^ batteries, which can improve the uniformity of the Li metal during growth.

#### 6.1.6 Gold

Although Au alloys with Li, it is not typically used as an alloy anode because of its high cost.^942^ Au exhibits a moderate theoretical specific capacity of 408 mAh g^–1^. The lithiation of Au begins with a plateau around 0.2 V vs Li/Li^+^ to form a crystalline Li–Au phase (suggested to be Li_5_Au_3_)^943^ not predicted on the phase diagram (Figure 46f), as based on synchrotron XRD results.^942,944^ A second transition then occurs at 0.1 V vs Li/Li^+^ to form the cubic Li_3_Au phase, which is on the phase diagram.^943,944^ On delithiation, the intermediate Li_5_Au_3_ phase forms before undergoing three more two-phase transformations to other metastable phases and then to delithiated Au.^943,944^

Rather than as a bulk electrode material, Au has mostly been explored as an interlayer to control the deposition of Li metal in cells with liquid or solid-state electrolytes. Yan et al. found that in Li-ion batteries with liquid electrolytes the use of a lithiated Au interlayer completely removed the nucleation overpotential seen during Li deposition on Cu foil.^483^ Due to this nucleation behavior, Au interlayers have been shown to enable the electrochemical growth of highly uniform Li films, preventing void formation at solid–solid interfaces.^315^

#### 6.1.7 Silver

Silver is also infrequently used directly as an anode material in Li-ion batteries due to its high cost. Instead, it has found beneficial use as an interlayer to improve Li deposition or to alter the kinetic and transport properties of electrodes.^315^ While it has a low specific capacity of 250 mAh g^–1^ for the LiAg phase, it exhibits a high volumetric capacity of 2200 mAh cm^–3^.^873^

Lithiation of Ag at practical currents occurs below 0.1 V vs Li/Li^+^, with the first transformation forming two unknown Li–Ag phases detected by XRD, one of which appears to be an irreversible transformation while the other is reversible.^945^ A relatively flat plateau appears around 0.04 V vs Li/Li^+^ corresponding to the formation of the β-LiAg phase (Figure 46g).^946^ Interestingly, the phase transformations in Ag have been shown to be strongly current dependent, indicating that nucleation of certain Li–Ag phases are kinetically hindered.^945,946^ In more recent work, the γ-Li_4_Ag phase has also been detected as the terminal phase, with a reconstitution reaction occurring around 0.05 V vs Li/Li^+^.^946^ Upon delithiation, an initial plateau occurs around 0.1 V vs Li/Li^+^ and has been shown through XRD to involve the transformation of β-LiAg into pure Ag. A second plateau is observed at 0.3 V vs Li/Li^+^ and is thought to be the transformation of the reversible unknown Li–Ag phase back into Ag.^945^

Silver electrodes generally exhibit limited capacity and rapid capacity degradation in Li-ion batteries. Corsi et al. found that bulk Ag films require very low currents for lithiation to occur,^946^ while nanoscale Ag particles incorporated into slurry-cast electrodes can be cycled at significantly higher currents with improvements in cycling stability.^947^ 3D electrode architectures involving Ag nanorods attached to graphene sheets have been shown to effectively increase capacity and improve rate performance.^948,949^ Ag particles have been found to be useful for guiding the growth of Li in composite C/Ag anodes for Li metal SSBs.^313^

### 6.2 Mechanistic Advantages of Solid-State Environments for Alloy Materials

#### 6.2.1 Challenges of Using Alloys with Liquid Electrolytes

**Figure 47:**
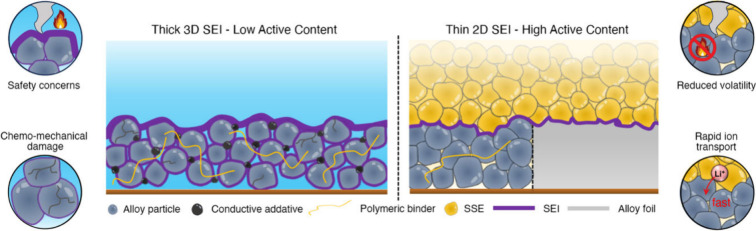
Schematic highlighting challenges of alloy anodes in liquid electrolytes (left) and the benefits of alloy anodes in batteries with SSEs (right). A significant benefit is the potential for reduced SEI formation in SSBs, as denoted by the thin SEI only at the alloy/SSE interface in the right schematic.

Alloy anodes tend to exhibit rapid capacity degradation when used in liquid electrolytes. Like graphite, many alloy anodes operate outside of the electrochemical stability window of liquid electrolytes, causing degradation of the solvents and salts to form an SEI (Figure 47). In contrast to graphite, the extreme volume change of alloy anodes can cause the alloy material and the SEI to undergo mechanical degradation/fracture.^5,956,957^ This process allows the liquid electrolyte to flow to contact new alloy surface area, which results in new SEI formation and continuous consumption of the electrolyte. Continued cycling exacerbates the issue with additional SEI formation occurring with every cycle. Eventually, this process can entirely consume the electrolyte in lean electrolyte conditions or result in electrochemically isolated particles due to the thickness of the SEI, both of which cause cell degradation.^956^

While all alloy anodes experience material volume changes, the details of morphology and stress evolution are different for different materials.^739,958−960^ Stress evolution within the active material depends on factors including material composition, phase transformations, mechanical properties, and crystallographic orientation.^515,739,900,958−960^ Multiple studies have shown that reducing particle size to the nanometer range can allow for accommodation of reaction-induced stress without fracture,^891,923^ with the downside being that small particles have greater surface area for SEI formation.^913,961^ Other studies have developed 3D architectures that can prevent fracture and reduce SEI growth.^893,962^ Despite progress, electro-chemo-mechanical degradation of alloy anodes in liquid electrolytes continues to hinder commercial application of alloy anodes.

#### 6.2.2 Advantages of Using Alloys with SSEs: Interfaces

**Figure 48:**
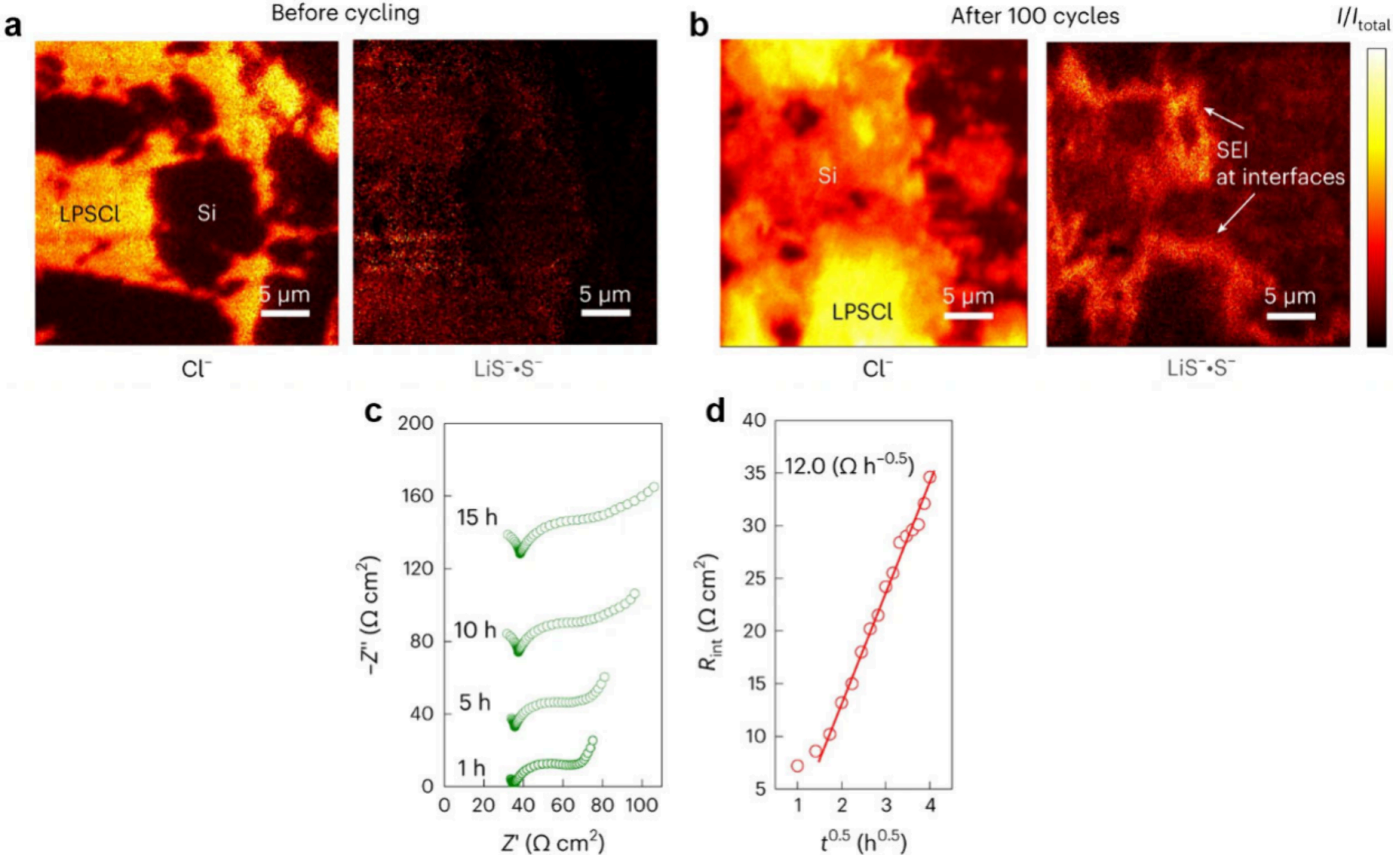
Characterization the interphase in SSBs. ToF-SIMS images of a Si-LPSCl interface (a) before and (b) after cycling. (c) Nyquist plot demonstrating an increase an impedance at the Si-LPSCl interface during rest after maximum lithiation. (d) Interface resistance from panel c as a function of t^0.5^, revealing growth rate of the interphase. (a–d) Reproduced with permission from ref (500). Copyright 2024 Springer Nature.

Unlike liquid electrolytes, which can wet the entire internal surface of porous electrodes,^963^ SSEs contact surfaces but cannot flow to wet new surfaces created due to fracture of electrode materials. This results in interphase formation occurring only at the initial points of contact between an SSE and electrode (Figure 47).^424^ For alloy anodes which may experience significant morphology changes during cycling, this effect substantially reduces the interphase that grows compared to liquid electrolytes, resulting in higher initial CEs in SSBs.^964,965^ Huo et al. used ToF-SIMS to show that interphase only formed at contacts between Si and LPSC electrolyte, and not within the volume of the Si electrode (Figure 48a,b), and EIS can be used to track the interphase growth rate (Figure 48c,d).^500^ Titration gas chromatography has shown that the amount of Li trapped in the SEI using liquid electrolytes increases significantly with cycling, accounting for the majority of capacity loss for Si anodes.^135,963,966^ Similar work on Si in SSBs has shown little change in the amount of Li trapped within the interphase with cycling along with stable interphase resistance, indicating that a passivating interphase layer can be formed using certain SSEs.^424^ The stability of the interphase on alloy anodes can also be inferred through the long-term cycle life demonstrated for various alloys compared to their performance with liquid electrolytes.^296,965,967^

The nature of the interphase when comparing SSEs and liquid electrolytes is also quite different. For instance, the LPSCl SSE is reduced by Li to form Li_2_S, Li_3_P, and LiCl, while interphases formed from typical liquid electrolytes contain inorganic and organic constituents.^424,968^ While there is clear evidence that there can be less electrolyte consumption when alloy anodes are used with SSEs compared to liquids, there is still much work to do in understanding the structure and properties of the interphases that form at various SSE/anode interfaces.

#### 6.2.3 Safety Benefits of Alloy Anodes in SSBs

SSBs are generally considered to be safer than traditional Li-ion batteries because of the reduced flammability and lower volatility of SSEs,^25,969^ although this has recently been questioned.^168,970^ Recent work has shown that thermal runaway can occur at greater rates in a SSB with a Li anode and oxide SSE than in Li-ion batteries.^970^ Furthermore, when Li metal anodes are used, the growth of Li dendrites through the SSE readily causes short circuiting, which can be a safety risk. The growth of dendrites has been characterized using X-ray computed tomography,^168^ FIB-SEM,^315^ and optical microscopy,^175^ as described in the previous section. The formation of voids at the Li–SSE interface exacerbates nonuniform growth of Li, increasing current concentration and promoting dendritic growth.^133,315,971^ Thus, the use of Li metal anodes still poses challenges even in SSB architectures.

Alloy anodes may offer safety benefits compared to Li metal, with the most obvious being that Li metal plating, and thus dendrite growth, is entirely avoided. Although Li metal can grow if alloys are overlithiated, some alloys can support high-rate alloying processes without the formation of Li dendrites.^967^ Lithiated alloys may also be more chemically stable upon environmental exposure than Li metal.^972^ Finally, contact loss is likely different at alloy/SSE interfaces compared to voiding at Li–SSE interfaces, which may help to reduce current constriction and its detrimental consequences.^133,315,964,973^

#### 6.2.4 Chemo-Mechanics of Alloys in SSBs

**Figure 49:**
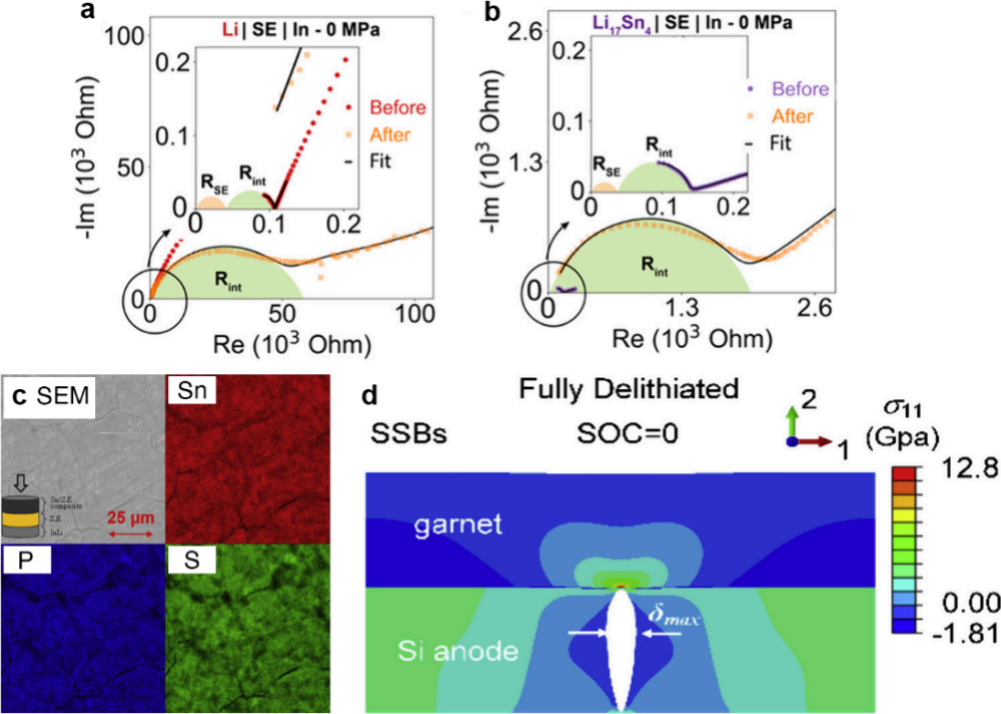
Chemo-mechanical characterization of alloys in SSBs. Nyquist plots of (a) a Li|LPSC|In cell and (b) a Li_17_Sn_4_|LPSC|In cell before and after stripping at 0 MPa stack pressure. (a, b) Reproduced with permission from ref (967). Copyright 2021 American Chemical Society. (c) SEM image and associated EDS maps of a tin composite anode after delithiation. Adapted with permission from ref (974). Copyright 2019 Elsevier. (d) Finite element modeling of normal in-plane stress distribution at a crack in a Si electrode in contact with a SSE. Reproduced with permission from ref (964). Copyright 2019 Elsevier.

The nature of the evolution of structure and morphology of alloy anodes in all solid-state environments is different than in liquid electrolytes, and these differences may also contribute to improved performance. For instance, Hänsel et al. used EIS to show that a tin electrode exhibited much more stable interfacial impedance in SSBs compared to Li metal, which tends to form voids during stripping (Figure 49a,b).^967^ Imaging and EDS, shown in Figure 49c, has shown that charge/discharge cycling does not significantly disrupt contact between alloy particles and SSEs in composite anodes, although this may not be true at low stack pressures.^974^ Contact loss at SSE interfaces still can occur when using alloy anodes,^500,973^ and improved understanding of contact loss dynamics of alloy anodes in SSBs is needed.

The chemo-mechanical environment of SSBs also impacts the fracture behavior of alloy anodes and may constrain volume change and/or mitigate mechanical degradation. Fracture of active alloy particles is common in liquid batteries due to the large volume changes that occur with lithiation and delithiation, and mechanical degradation can lead to short cell lifetimes. Relatively high stack pressures can cause merging of particles of Li_*x*_Si during lithiation,^424^ and fracture is less apparent.^964,975^ Modeling, such as that in Figure 49d, has revealed that the SSE can both influence volume change of alloy anodes and reduce the extent of chemo-mechanical damage to the electrode.^964^ However, the relationship between chemo-mechanical fracture and stack pressure is still unclear for alloy anodes. For instance, Han et al. found that various alloys exhibited unique stress responses in SSBs, emphasizing the need to better understand chemo-mechanics in these battery chemistries.^296^

#### 6.2.5 Diffusion Coefficients

**Figure 50:**
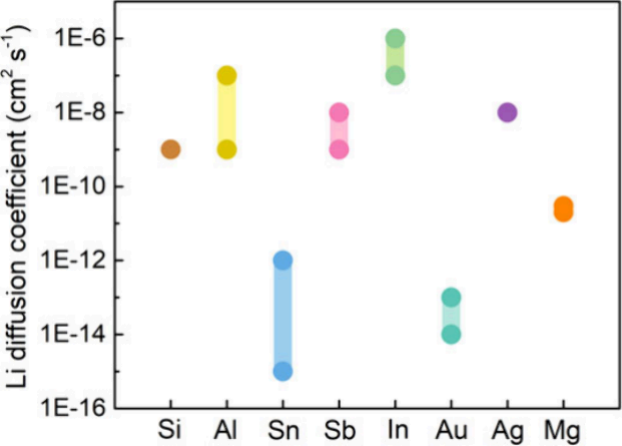
Measured Li diffusion coefficients of various Li alloy materials. Data is compiled from refs (424, 830, 833, 835, 860, 983−987).

For intercalation and alloy materials, solid-state diffusion through the active material must be sufficiently fast to transport Li across micron-scale distances to support the required charge/discharge rates. In liquid-electrolyte batteries with alloy anodes, solid-state diffusion limitations are less common since the liquid can infiltrate pores and cracks to deliver ions to the material. SSBs tend to feature composite electrodes (i.e., mixtures of the active material and SSE) to reduce diffusion lengths for intercalation materials such as NMC.^134,976^ Although the composite electrodes facilitate intercalation of the active material, the active material density in such structures is reduced.^5^ Because many alloy anodes have higher solid-state diffusivities (shown in Figure 50) than intercalation materials or even pure Li metal (∼10^–10^ cm^2^ s^–1^),^792−794,977−981^ they can potentially be used in SSBs either directly as foils or with lower fractions of SSEs in composites. Alloy foil anodes, which have high volumetric capacity since they have no inactive components, have shown much better performance in SSBs than in Li-ion batteries.^982^ In other cases, alloy particles can be slurry-cast with no conductive additive and with very little binder content. For example, Tan et al. showed that 99.9 wt % particulate Si anodes exhibit good capacity retention over 500 cycles with a sulfide SSE at 5 mA cm^–2^.^424^

#### 6.2.6 Manufacturing Advantages of SSBs with Alloy Anodes

Li metal is sensitive to both air and moisture, requiring atmospheric control solutions such as dry rooms for handling. Exposure of Li to air creates thick oxide and carbonate layers,^988,989^ which negatively impacts electrochemical behavior. Exposure to water produces gaseous hydrogen,^990^ which is a safety concern. While coatings can improve the stability of Li metal,^988,990^ incorporating Li metal into battery manufacturing processes will likely increase cost and complexity.

Many alloy anodes can be more easily integrated into roll-to-roll battery manufacturing while still enabling high capacity. Alloy anode materials are chemically stable in air and are mostly environmentally benign (like Si, Al, and Sn). Many alloy materials can be manufactured as micro- and nanoscale particles, which can be mixed into slurries, making use of current Li-ion battery electrode fabrication facilities.^991^ Additionally, alloy slurries are compatible with water instead of n-methyl-2-pyrrolidone,^923^ reducing processing cost and preventing the need for solvent capture systems.^992^ Foil-based alloy anodes could be directly used in liquid-electrolyte batteries or SSBs, potentially doubling as the current collector to reduce weight.^2,908^

### 6.3 Mechanistic Challenges of Solid-State Environments for Alloy Materials

#### 6.3.1 Solid-State Transport

Stable cycling of alloy anodes in SSBs relies on solid-state Li (de)alloying reactions. In contrast to liquid electrolytes that fully wet porous electrodes, ion transport usually occurs over longer distances in alloy electrodes within SSBs.^5^ Additionally, when compared to Li metal in SSBs, alloys must support diffusion within the host structure, while Li metal only requires electrodeposition at an interface. The rate-determining step for an alloying or dealloying reaction is often either bond breaking at the two-phase reaction front, or Li diffusion within the alloy. Therefore, low energy barriers at reaction fronts and rapid Li diffusion (generally, Li diffusivity >10^–11^ cm^2^ s^–1^) in Li alloys are essential factors for enabling reactions at rates that are appropriate to support reasonable charge/discharge behavior.^993^

Alloy phases exhibit a wide range of Li diffusion coefficients, as previously shown in Figure 50. Alloy phases with relatively high Li diffusivity, such as LiIn, Li_*x*_Si, and LiAl, have been observed to support stable cycling in SSBs.^332,424,494^ Other alloy metals, such as Ag and Mg, also show high Li diffusivity and have been demonstrated to be useful as interfacial layers to prevent void formation,^315,830^ although such effects are not solely due to diffusivity. While the high diffusivity of Li in many alloys is an advantage, diffusive transport through thick alloy materials within electrode architectures can be a challenge.

We consider the Li–In alloy system as an example. As discussed in Section 6.1, Li and In form three primary alloy phases, including LiIn and Li_13_In_3_. Electrochemical alloying of In with Li results in three plateaus, with potentials of 0.62, 0.33, and 0.12 V vs Li/Li^+^.^994^ These reactions involve the movement of reaction fronts with end-member phases of each plateau on either side of the reaction front, with Li diffusion to the front required to support the reaction. The reaction of In to form LiIn at 0.62 V vs Li/Li^+^ features low overpotentials, which is partially due to the very high Li diffusivity of Li in the LiIn phase (10^–6^ to 10^–7^ cm^2^ s^–1^).^332,985^ This is one of the reasons that Li–In alloys are widely used as counter electrodes in SSBs. Li–In phases with higher Li contents show decreased Li diffusion coefficient (10^–12^ to 10^–13^ cm^2^ s^–1^), which leads to higher overpotentials and reduced cycling stability.^993^ Thus, certain alloys with intrinsically lower Li diffusivities, such as Li–Sn alloys, may present challenges for incorporation into SSBs.

**Figure 51:**
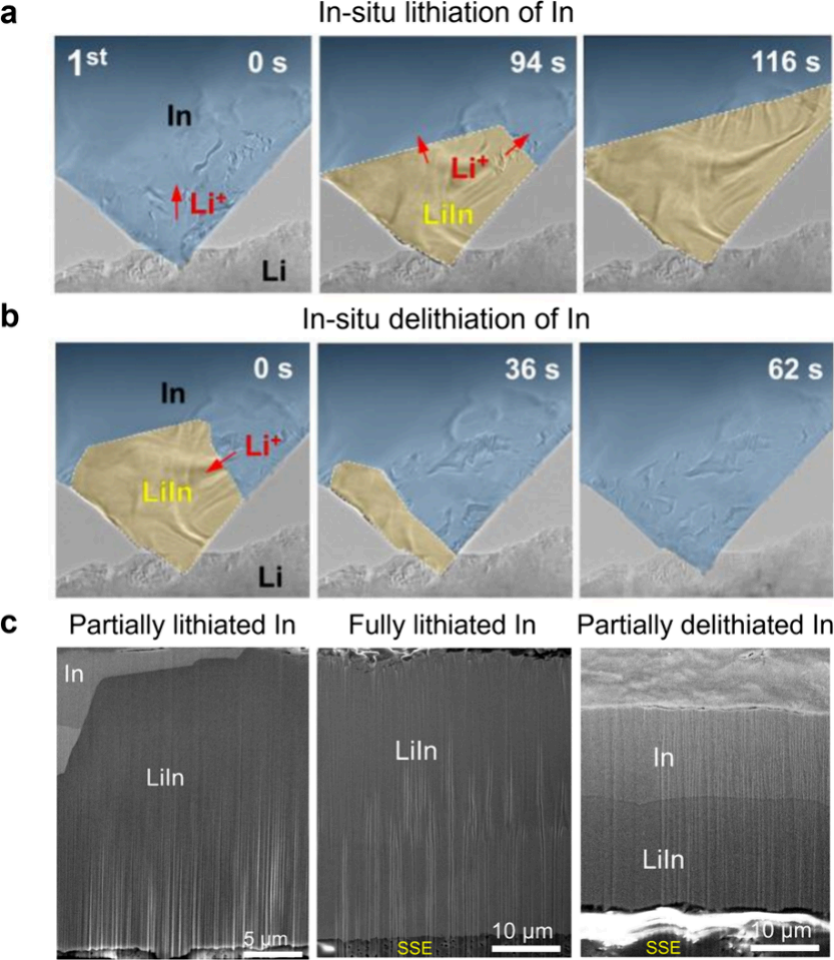
Characterization of the lithiation and delithiation behavior of Li–In alloys. (a) *In situ* TEM images showing the rapid lithiation of In to form LiIn. (b) *In situ* TEM images showing the rapid delithiation of LiIn to In. The yellow shaded areas represent the lithiated In. (a, b) Reproduced with permission from ref (995). Copyright 2023 Elsevier. (c) Cross-sectional cryo-FIB-SEM images of dense In foil anodes after partial lithiation, full lithiation, and partial delithiation. Adapted with permission from ref (973). Copyright 2024 American Chemical Society.

Beyond diffusion coefficients, the microstructural and phase evolution of alloy materials during charge/discharge are important for ensuring good electrochemical reversibility in SSBs. *In situ* TEM observations by Wang et al. have shown that rapid alloying/dealloying reactions are supported by fast Li transport in a material, which is critical for achieving stable cycling and high charge/discharge rates.^995^ When a negative current was applied to an In electrode in the TEM, the lithiation front moved rapidly through the In, as shown in Figure 51a. Upon reversing the current, delithiation was observed to initiate immediately, as shown in Figure 51b. Jeong et al. used cryo-FIB to investigate the structural evolution and electrochemical behavior of In foil in SSBs (Figure 51c).^973^ The results showed a gradual receding of the reaction front toward the SSE interface during delithiation, confirming (along with the TEM experiments in Figure 51b) that the LiIn phase remained in contact with the SSE during delithiation. This is likely due to the fast transport of Li in the LiIn phase. This continued contact of the lithiated phase with the SSE facilitates Li transport to the SSE, which is highly advantageous for full capacity utilization. Alloy materials with lower Li diffusivity could exhibit greater concentration gradients which can cause nucleation of delithiated phases near the SSE interface, which can lead to diffusional Li trapping (as described in the next section).

#### 6.3.2 Li Trapping

**Figure 52:**
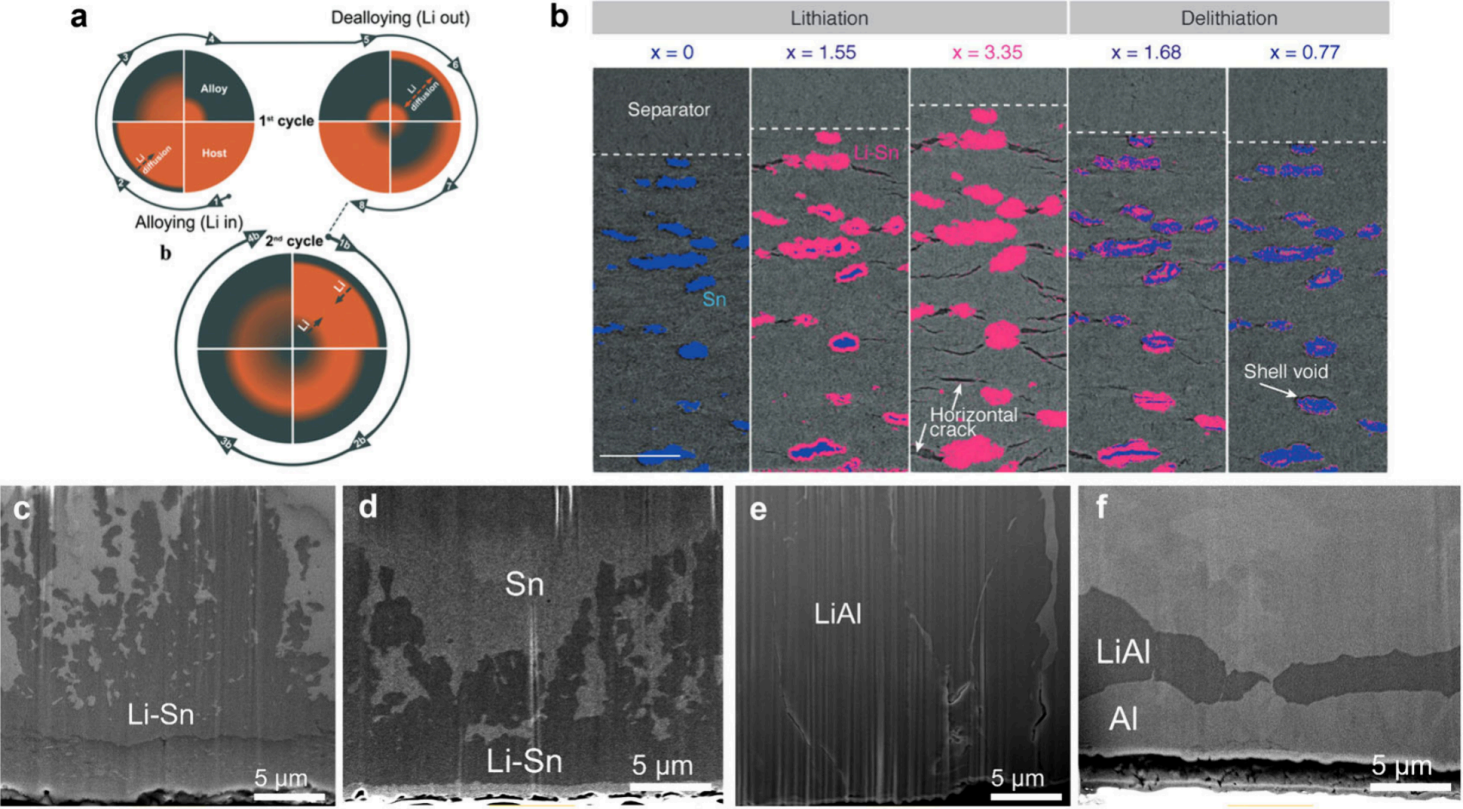
Diffusional Li trapping. (a) The diffusion trapping model. Li diffuses into the electrode during the lithiation step and the Li concentration near the electrode surface decreases during the subsequent delithiation step. Some Li remains in the particle after delithiation due to diffusional trapping. Reproduced with permission from ref (996). Copyright 2017 Royal Society of Chemistry. (b) Cross-sectional X-ray computed tomography images of a Sn composite electrode at various (de)lithiation states. The Sn and Li–Sn phases are shown in blue and pink, respectively. The dashed line represents the border between the electrodes and separator. Reproduced with permission from ref (382). Copyright 2019 WILEY-VCH Verlag GmbH & Co. KGaA. (c–f) Cross-sectional cryo-FIB-SEM images of dense alloy anodes after lithiation to 1.5 mAh cm^–2^ and after delithiation. (c) Sn after lithiation, (d) Sn after delithiation, (e) Al after lithiation, (f) Al after delithiation. Reproduced with permission from ref (973). Copyright 2024 American Chemical Society.

Diffusional Li trapping is a key challenge for the use of alloy anodes in SSBs, especially for materials that undergo two-phase reactions.^973^ Typical schematics of Li concentration profiles in two-phase alloy materials are shown in Figure 52a.^996^ During the first lithiation, Li diffuses into the material behind the reaction front to form the Li alloy phase. Upon dealloying, Li can be removed from near the surface of particles, and if the newly delithiated phase exhibits low Li diffusivity, it can act to kinetically trap Li in the lithiated phase(s) behind it.^996^ Indeed, many metals that form alloys have low Li diffusivity in their pure phases, which makes them susceptible to Li trapping. The extent of Li trapping should depend on the time scale of the experiments and the thickness of material through which diffusion must occur. This model could explain why improved cycling performance generally is seen for electrodes with thin-film and nanoparticle anodes.^296^

*Operando* X-ray computed tomography has been used to observe Li trapping in alloys for SSBs. Wu et al. studied the lithiation/delithiation dynamics of tin particle composites with the LPS SSE, observing interfacial crack formation and Li trapping.^382^ The difference of X-ray attenuation coefficient enables identification of pure Sn vs alloy phases. Figure 52b shows a cross-sectional XCT image of the Sn anode during lithiation and delithiation, with pink-highlighted Li–Sn phases observable in the interior of particles after delithiation due to trapping. Microstructural evolution and Li trapping have also been observed with cryo-FIB-SEM. Figure 52c shows a Sn foil electrode after partial lithiation, detailing the morphological distribution of the Li–Sn phase (dark gray) and Sn (bright gray). After delithiation, a tin layer with lighter contrast is visible at the interface with the SSE at the bottom of the image (Figure 52d). This is a direct observation of diffusional Li trapping, as it appears that the Li cannot be effectively transported through the delithiated tin to reach the SSE interface.^973^ Similarly, an Al foil electrode after lithiation (Figure 52e) and delithiation (Figure 52f) shows evidence of Li trapping in the LiAl phase, with a brighter delithiated Al region adjacent to the SSE interface.^973^

#### 6.3.3 Contact Loss at Interfaces

**Figure 53:**
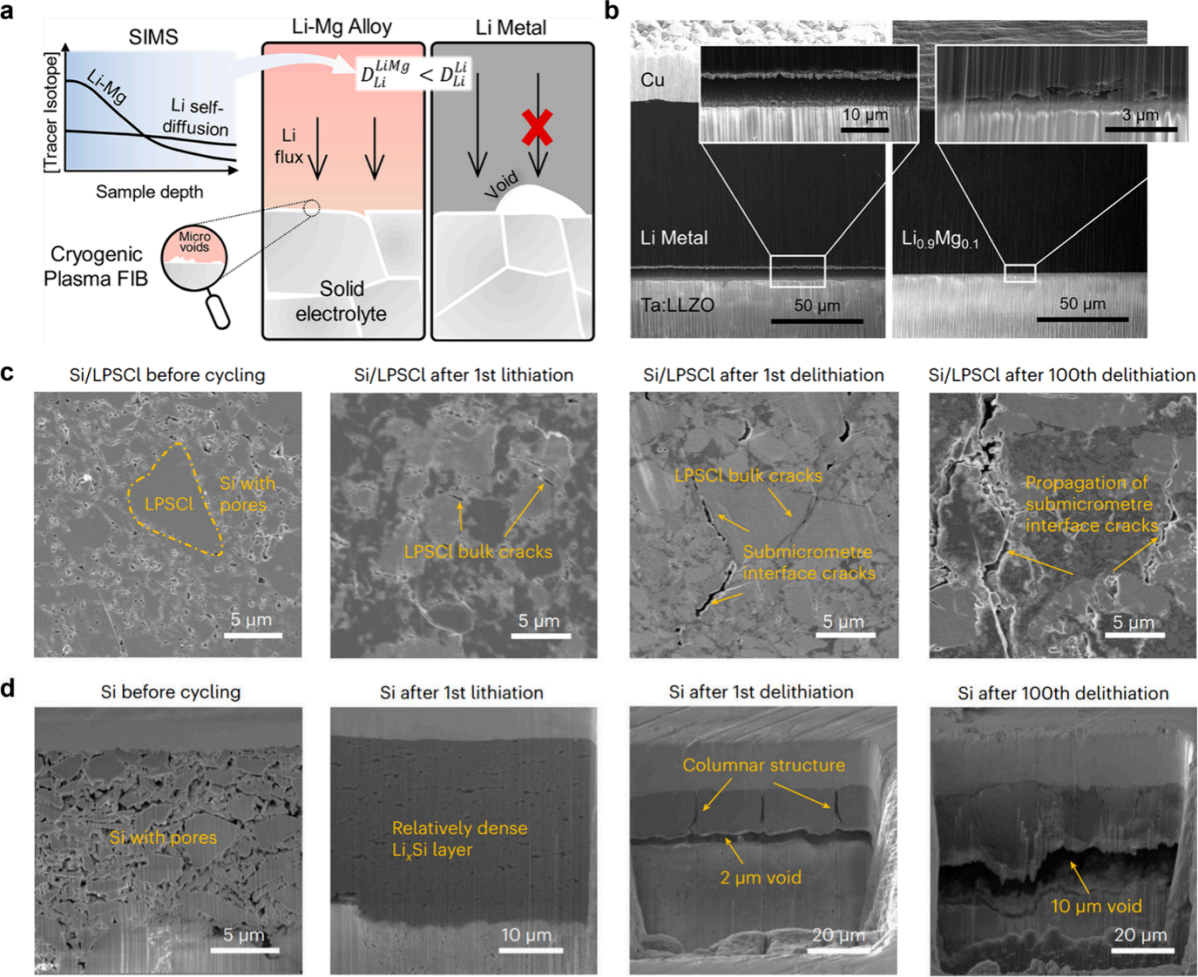
Void formation and contact loss during cycling in SSBs. (a) Schematic of experiments to measure diffusivity in Li and a Li–Mg alloy with SIMS. (b) Cryogenic FIB-SEM characterization of the cross-sections of Li metal and Li_0.9_Mg_0.1_ after stripping in an SSB. (a, b) Reproduced with permission from ref (833). Copyright 2022 American Chemical Society. (c) Cross-sectional SEM images of a Si/LPSCl composite anode before cycling, after the first lithiation, after the first delithiation, and after the 100th delithiation. (d) Cross-sectional SEM images of an SSE-free Si anode before cycling, after the first lithiation, after the first delithiation, and after the 100th delithiation. (c, d) Reproduced with permission from ref (500). Copyright 2024. Nature Springer.

Contact loss at alloy anode/SSE interfaces is a challenge that manifests in SSBs but not in conventional Li-ion batteries.^500^ Contact loss occurs because of morphological changes or mechanical damage at the interface, and loss of contact leads to higher impedance and cell degradation. FIB-SEM characterization has been used to observe this phenomenon.^833^ For example, the evolution of contact between a Li–Mg anode and the SSE has been investigated (Figure 53a).^833^ This study showed that a Li_0.9_Mg_0.1_ anode retained relatively uniform contact with the SSE after delithiation to a capacity of 1 mAh cm^–2^, with a few voids forming at the interface (Figure 53b). In contrast, a Li metal electrode exhibited complete loss of contact at the SSE interface after stripping only 0.2 mAh cm^–2^. This result suggests that while alloys exhibit different contact loss mechanisms than Li metal electrodes, in some cases contact retention may be improved for alloys.

Other work has used cryogenic FIB-SEM to characterize Si anodes and their contact evolution in SSBs.^424^ C-free micro-Si particulate electrodes were used with a porosity of 40% after calendering. After lithiation/delithiation, the Si electrode did not revert to its original microparticle structure but instead evolved to form larger particles with cracks between them; this particle merging is likely affected by applied stack pressure. Different from In and Al, Li incorporation into Si decreases the hardness.^997^ The mechanical damage and interfacial fracture/contact loss may thus be affected by the mechanical properties of the specific alloy composition being investigated.

Other work by Huo et al. studied the chemo-mechanical failure mechanisms of both composite and pure Si anodes in SSBs.^500^ Si/LPSC composite anodes (Figure 53c) developed submicron cracks at interfaces after the first delithiation due to the shrinkage of Si. These cracks propagated after the 10th and 100th delithiation, causing electrode degradation. For the SSE-free Si electrode in (Figure 53d), a dense and interconnected Li–Si microstructure with minimal porosity was observed after the first lithiation. The expansion of Si enables good interfacial contact and densifies the electrode during lithiation (this densification process is likely affected by stack pressure). However, in the following delithiation step, the Si layer shows a columnar microstructure and a ∼ 2 μm void gap between the Si layer and the SSE. An increased extent of voiding was observed at the interface after the 100th delithiation. While this *ex situ* characterization could unintentionally induce damage to the electrode structures from the disassembly of the SSB cells, these results indicate that composite and pure Si electrode structures undergo different contact loss and damage evolution. *Operando* or *in situ* characterization of Si and other alloy anode materials is needed for improved understanding of damage evolution that is unique to SSB environments.

### 6.4 Novel Electrode Structures Enabled by Solid-State Architectures

#### 6.4.1 Micron-Scale Active Material Particles

As previously discussed, particulate alloy anodes in liquid-electrolyte Li-ion batteries tend to continuously form SEI at their surfaces during cycling due to mechanical fracture of the existing SEI and rewetting of the electrolyte. SSBs offer the possibility of mitigating this issue since SSEs do not flow to wet internal surfaces. As a result, SSBs may enable the direct use of micron-scale alloy particles, which tend to exhibit very poor performance in Li-ion batteries. Indeed, SSBs with micron-scale Si^424^ and other alloy materials^296^ have shown much improved cycling stability compared to Li-ion batteries.

**Figure 54:**
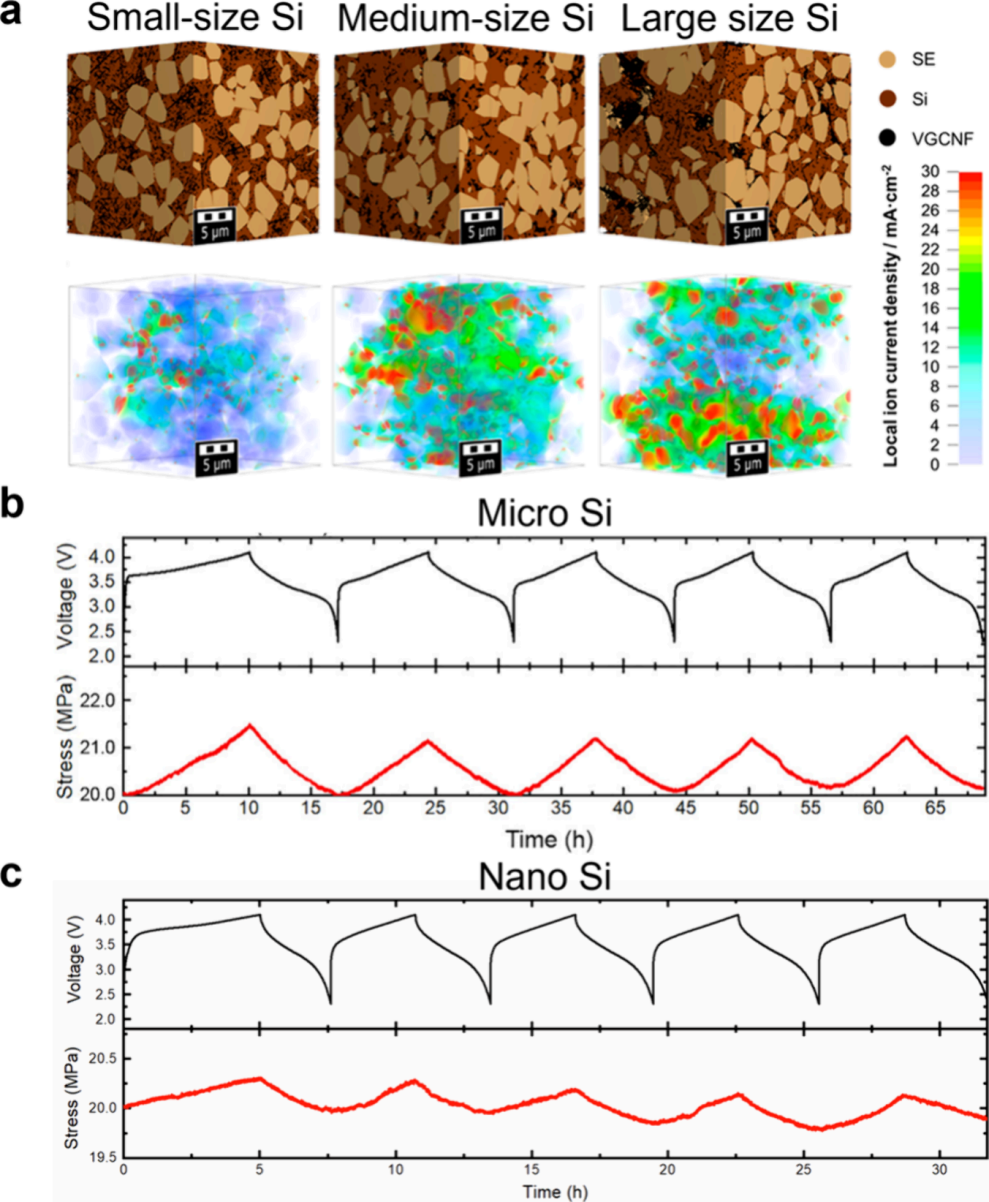
Effects of particle size on electrochemical performance and electro-chemo-mechanical behavior of alloy anodes. (a) Microstructure models and corresponding current distribution through composite Si electrodes with varying Si particle sizes. Adapted with permission from ref (998). Copyright 2023 American Chemical Society. (b, c) Galvanostatic cycling of composite electrodes with (b) micro- and (c) nanosized Si particles in SSBs with LPSC solid electrolyte and NMC-111 cathodes, along with the measured stack pressure changes in each cell. (b, c) Reproduced with permission from ref (296). Copyright 2021 Elsevier.

Rana et al. investigated the effects of particle size within composite alloy anodes in SSBs.^998^ As shown in Figure 54a, Si composite anodes with different particle sizes were tested, and their effective ionic conductivity was simulated based on electrochemical data. Composites with nano- vs micro-sized Si were compared, and the larger size mismatch between the SSE and active material with larger particle sizes led to more detrimental pores and nonuniform ionic current distribution. Thus, matching length scales between active particles and SSE in composites is important.

Han et al. measured the stack pressure evolution of full cells with composite anodes featuring Si particles with different sizes (microscale and nanoscale, Figure 54b,c).^296^ An initial stack pressure of 20 MPa was applied, and the measured stack pressure repeatedly increased and decreased during charge/discharge. Even when normalized to areal capacity, the cell with the micro-Si anode showed larger stress changes every cycle, which indicates that particle size of alloy anode composites affects stress evolution. Smaller particles can expand into available porosity within the composite electrode more easily, resulting in active material volume changes with minimal stress increase, while larger particles will force the entire electrode structure to deform. Thus, although electrodes comprising micron-scale particles can be stable in SSBs, the chemo-mechanical environments of different particle sizes must be carefully considered.

#### 6.4.2 Alloy Foils

**Figure 55:**
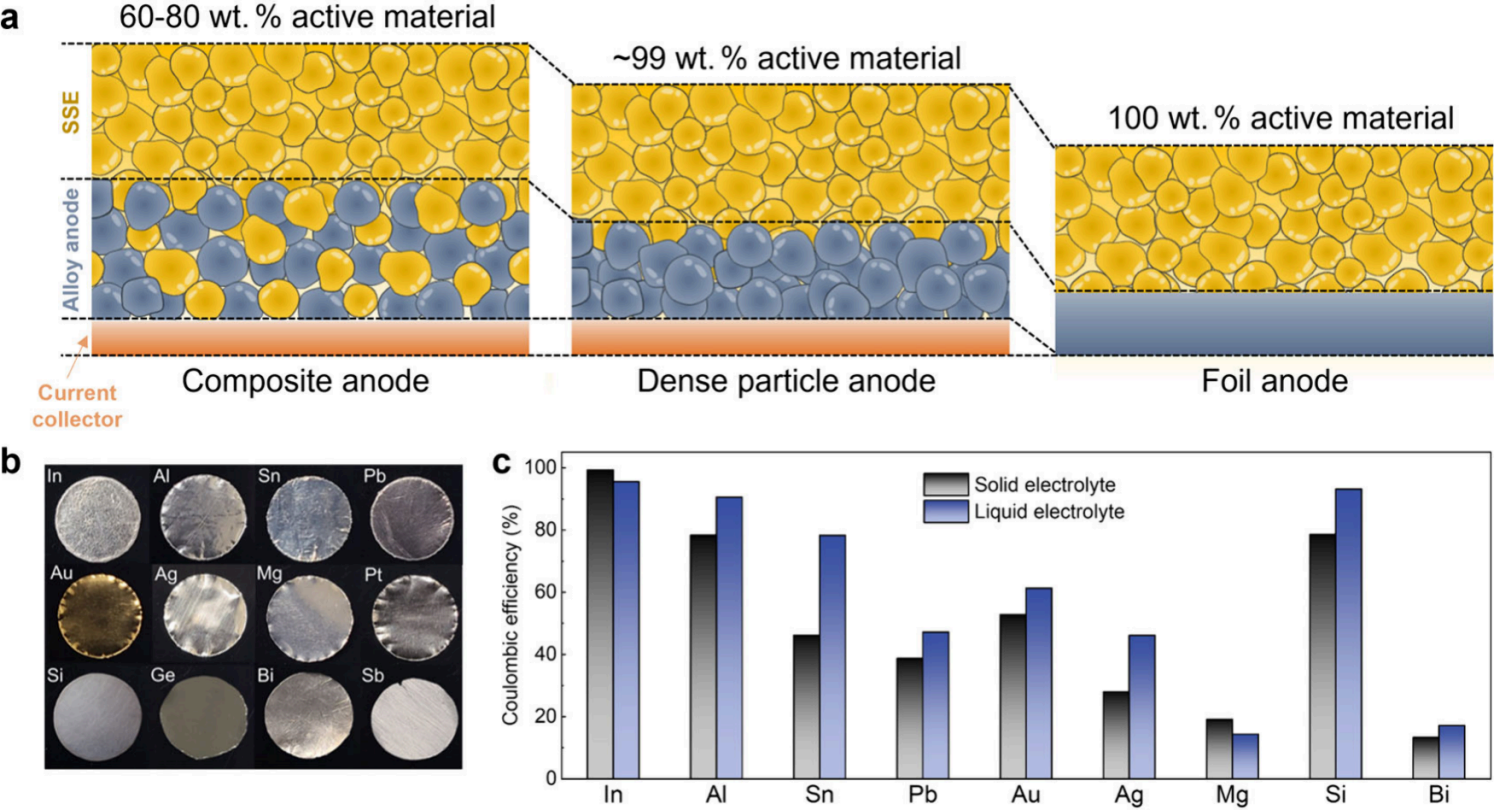
Electrochemical behavior and (de)lithiation mechanisms of foil anodes. (a) Schematics showing reduced electrode thickness and therefore increased energy density when moving from composite anodes to dense foil-based anodes. (b) Photographs of 12 elemental alloy anodes. (c) First-cycle CE comparison of alloy anodes in liquid-electrolyte and SSE cells at 25 °C with stack pressure of 8 MPa. (b, c) Reproduced with permission from ref (973). Copyright 2024 American Chemical Society.

Most research on alloy anodes for SSBs has focused on the development of particulate-based slurry-cast electrodes, in line with conventional Li-ion battery electrode processing. Due to the dimensional stability of SSEs, however, dense metal foil alloy anodes can be used directly without the extensive SEI growth that plagues these materials in Li-ion batteries.^908^ Metallic alloy foils represent a broad yet underexplored class of anode materials for SSBs, and they are particularly attractive because of their dense material packing and thus high volumetric capacity, as shown in Figure 55a. Furthermore, they may remove the need for a Cu current collector, which further enhances specific energy (Figure 55a). Of particular interest are systems based on Al and In foils, as these materials provide high capacities and can be engineered for stable cycling.^332^

Jeong et al. investigated the fundamental electrochemical behavior of a series of elemental foil electrodes in conjunction with argyrodite-based SSE (Figure 55b).^973^ The materials showed highly divergent first-cycle CE, ranging from above 99% for In to ∼20% for Sb (Figure 55c). In is unique since its initial CE approaches 100%, which is enabled both by the high diffusivity of Li in the LiIn phase (Section 6.3.1) and the microstructural evolution that avoids Li trapping (Section 6.3.2). Among the 12 different elemental electrodes investigated, Al, In, and Si were highlighted as promising candidates for direct use as dense active materials, with each offering high CEs and the potential for improved energy density over graphite-based systems.^973^

#### 6.4.3 Engineered Multiphase Foils for Improved Performance

**Figure 56:**
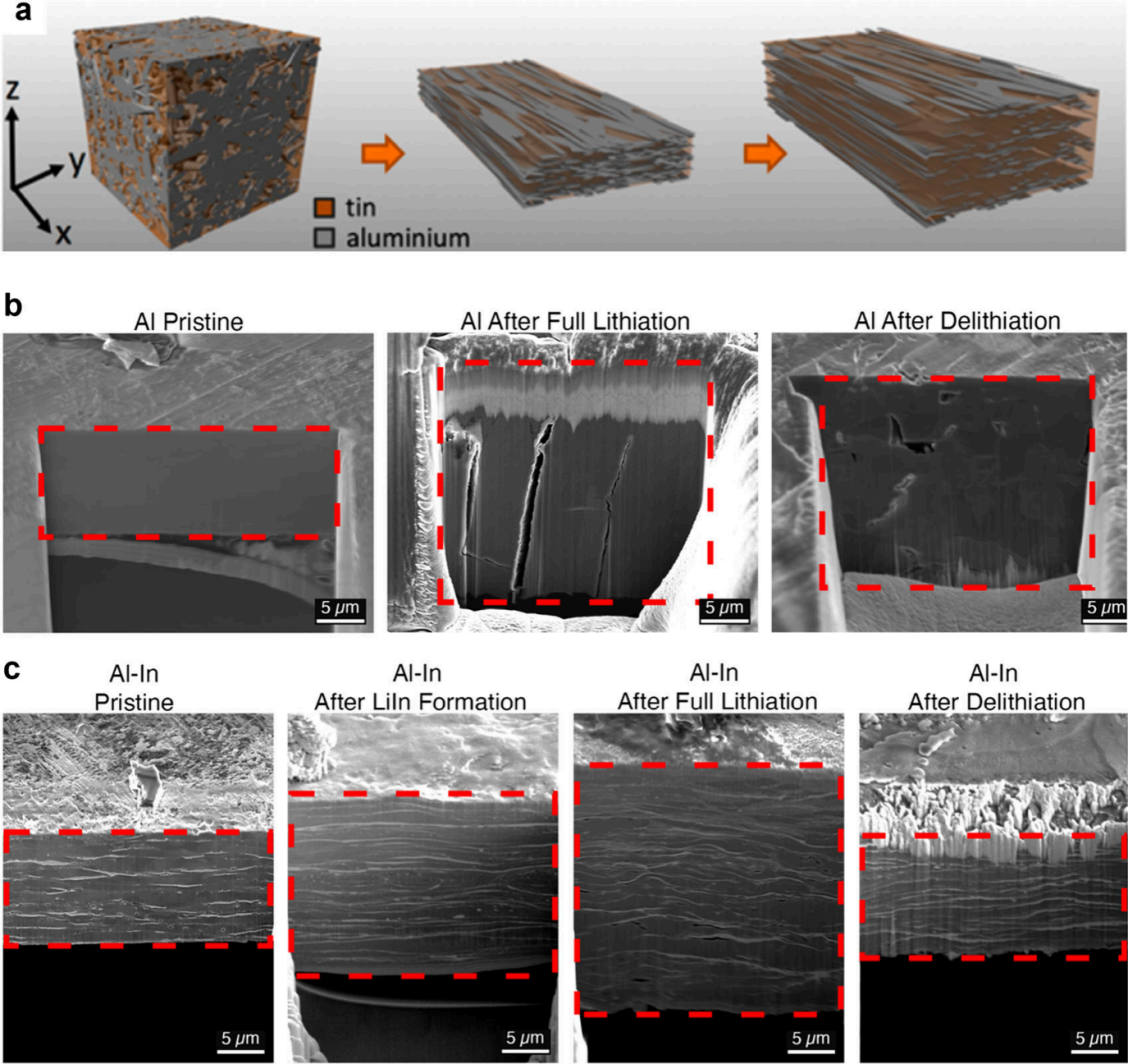
Characterization of multiphase foil electrodes for batteries. (a) Illustration of the design and fabrication of microstructured multiphase foils via rolling. Reproduced with permission from ref (1000). Copyright 2022 American Chemical Society. (b) Cross-sectional cryo-FIB-SEM images of a pristine Al foil, Al foil after full lithiation, and Al foil after delithiation, showing the presence of cracks and damage. (c) Cross-sectional cryo-FIB-SEM images of pristine Al–In foil, Al–In foil after LiIn formation, Al–In foil after full lithiation, and Al–In foil after delithiation. The lighter In regions remain intact, largely preventing mechanical degradation and ensuring fast Li transport through the foil. (b, c) Reproduced with permission from ref (332). Copyright 2023 Nature Springer.

Microstructure engineering of multiphase foil materials has been shown to be beneficial for electrochemical cycling stability compared to pure metal foils with a single phase.^999^ “Interdigitated eutectic” Al–Sn alloy foils have been fabricated via cold rolling and tested in liquid-electrolyte batteries (Figure 56a).^999,1000^ The microstructured anode framework and the interfaces between the two phases enhanced Li ion transport, leading to high specific volumetric capacity and good cyclability.^999^

For SSBs, such microstructured multiphase designs can mitigate diffusional Li trapping and chemo-mechanical degradation of foil anodes.^332^ Liu et al. designed multiphase Al–In foils with 95 wt % Al and 5 wt % In and investigated the microstructural evolution of multiphase anodes as shown in Figure 56b.^332^

Figure 56b shows cryo-FIB-SEM imaging results of a pure Al electrode at different stages of cycling.^332^ After lithiation, the foil anode has expanded and shows distinct cracks and voids. For the multiphase Al–In foil anode (Figure 56c), the material expands and contracts during lithiation/delithiation with the LiIn layers remaining intact throughout the entire process, which is beneficial for enhancing the reversibility, rate behavior, and performance. The LiIn phase can support relatively fast Li diffusion, enabling transport of Li from the LiIn network to react with Al to form LiAl, which plays an important role in minimizing Li trapping.^332^ This multiphase design does not exclusively apply to Al foil-based anodes, as the design concept of an interspersed mixed-ion-electron-conducting phase within a dense foil can also be effective for improved performance in other alloy material systems as SSB anodes.^332^

### 6.5 Effects of Stack Pressure and Current Density on Morphology Evolution

**Figure 57:**
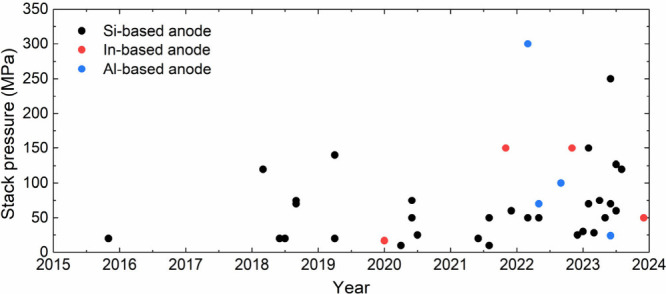
Applied stack pressure used for alloy anode testing in SSBs reported in literature in recent years (refs (296, 328, 424, 491, 547, 755, 956, 975, 991, 998, 1001−1023)).

Extremely high stack pressures are widely used in literature for stable cycling of alloy anodes, with stack pressures on the order of 50 MPa commonly used for testing Si anodes in SSBs.^424^ As previously noted, much lower stack pressures (<1 MPa) are required for commercial applications.^790^Figure 57 compiles and compares the electrochemical performance of various alloy anodes and the stack pressures used for cycling of the SSB cells as published in recent literature. Most works use high stack pressures ranging from 20 to 100 MPa for cycling of alloy anodes in SSBs, and there is no consistent trend of stack pressure with time of publication. This points to a critical gap in our understanding: the influence of stack pressure on structural evolution and fundamental alloying/dealloying processes within alloy anodes is not well understood. Characterization of the electro-chemo-mechanical evolution of alloy anodes and their interfaces is thus important for enabling advances to reduce stack pressure.

Prior work has compared stress–strain curves of various Li alloys.^1001^ The high compressive strength and slightly higher Young’s modulus of Li–Al and Li–Si can affect contact evolution with the SSE during long-term cycling, leading to cracking and contact loss during repeated mechanical loading and unloading.

Several studies have shown a pressure dependence of the electrochemical performance of Si anodes in SSBs.^975,1008^ For example, under stack pressures of 140 and 20 MPa, half cells with Si electrodes have been observed to exhibit similar rate performance, achieving 2000 mAh g^–1^ specific capacity of Si at C/5. Further lowering the external stack pressure to 5 MPa results in significantly lowered capacity and CE (CE is ∼80% under 140 and 20 MPa vs 64% under 5 MPa).^1008^ Lower stack pressures (<20 MPa) also tend to result in reduced cycling stability. For instance, a cell at 3 MPa exhibited a low initial CE of 55% and only survived eight cycles of operation before failure despite a slow rate of C/20 being used.^975^

Optimizing the processing of SSEs and the use of different pressurizing media can help improve electrochemical performance of SSBs at low stack pressures. Li et al. analyzed fabrication methods and corresponding properties of sulfide-based composite SSE films.^1024^ Chen et al. applied isostatic pouch cell holders utilizing air as a pressurizing media to achieve relatively low cycling pressure for Si-anode SSBs.^1025^ Compared to traditional uniaxial cell holders, the isostatic press chamber applied accurately regulated and homogeneously distributed pressure. Pouch cells were fabricated and tested under 1 to 5 MPa, revealing improved electrochemical performance with higher cycling pressures. A bilayer pouch cell cycled under the isostatic pressure showed stable cycling for 100 cycles at C/10 under 5 MPa.

## 7 Composite Cathodes for SSBs

### 7.1 Challenges of Composite Cathodes for SSBs

**Figure 58:**
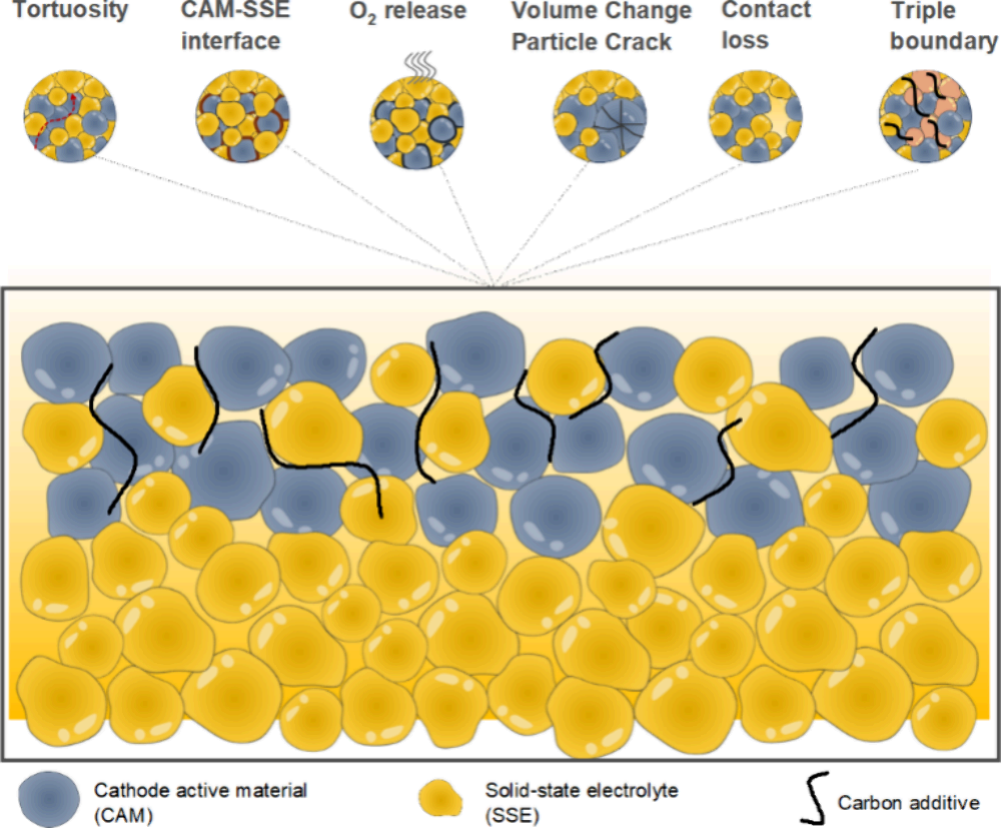
Challenges associated with composite cathodes in SSBs.

Cathode active materials (including intercalation, insertion, and conversion-type materials) are generally semiconductors or insulators. They are therefore often mixed with SSEs, C additives, and binder to form composite electrodes to achieve the needed ionic and electronic conductivity for use in SSBs.^1026−1029^ The inclusion of these multiple phases results in various interfaces that influence the electrochemical properties of the electrodes. Cathode composites for SSBs feature distinct chemo-mechanical challenges compared to anodes that need to be characterized and overcome to improve performance. The main challenges associated with cathode composites (Figure 58) include the electrochemical degradation of SSEs at high voltages, fracture of particles, delamination/contact loss at interfaces, and transport limitations. In this section, we discuss the challenges facing cathode composites for SSBs and how the mechanisms associated with these challenges have been characterized.

#### 7.1.1 Transport Limitations and Microstructure Control

The ionic and electronic conductivity of composite cathodes are critical for enabling sufficient transport of ions and electrons for electrochemical reactions. Since cathode composites consist of multiple solid phases (active material, SSE, C, and binder), particular loadings of various components are needed to optimize transport pathways.^77^ Thus, understanding and controlling the complex microstructure of cathode composites is important for maximizing performance.

**Figure 59:**
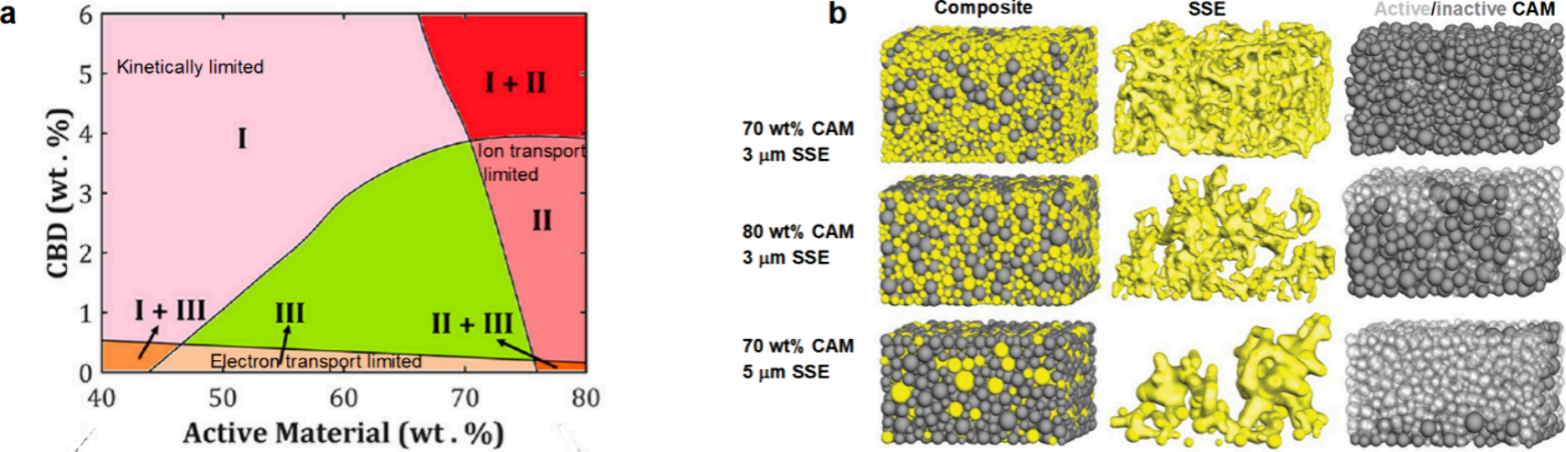
(a) Summary of cathode microstructure-dependent kinetic, ionic, and electronic transport limitations. Adapted with permission from ref (1030). Copyright 2022 American Chemical Society. (b) Visualization of ionic percolation networks of model composites (left), isolated SSE networks (middle), and cathode active materials (right) with varying particle size and active material content. Yellow represents SSE, while light and dark gray represent active materials that are electrochemically active or inactive, respectively. Adapted with permission from ref (1032). Copyright 2019 WILEY-VCH Verlag GmbH & Co. KGaA.

In general, high active material loading is needed to enable SSBs with high energy density. However, active material loading is usually increased at the expense of the SSE material, which tends to detrimentally affect the ion transport characteristics of the composite (Figure 59a). Therefore, control of the microstructure of cathode composites to optimize ion transport is useful to diminish tortuosity effects and enable higher active material loading.^1030^ For instance, there have been research efforts to increase interfacial area, homogenize the distribution of various phases, and decrease voids in the microstructure.^1031,1032^

Composite cathodes with intercalation materials and sulfide SSEs typically comprise between ∼30 and ∼50 wt % of SSE.^77,1033,1034^ Reducing the SSE content improves volumetric capacity at the expense of ionic conductivity.^1033^ Intercalation cathode composites usually include 2–3 wt % C additives for electronic conduction enhancement, whereas S-based cathodes typically include ∼20 wt % C content due to the highly insulating nature of S.^498,1035^ Dewald et al. investigated the optimum ratio between S and C in SSB electrode composites by analyzing electronic and ionic tortuosity factors using DC polarization experiments. The results showed that the ionic conductivity value of the SSE is critical for reducing the amount of SSE in the composite. Furthermore, the authors reported a decrease of the ionic conductivity of thiophosphate SSEs upon processing with the cathode.^1035^ Ohno et al. investigated charge transport properties of sulfur composite electrodes using EIS and also reported decreased ionic conductivity with decreased SSE fraction.^1034^ Similar challenges exist for sulfur-based composite film pouch cells. Readers are referred to the review by Ye et al., which provides valuable insights into how active material loading and microstructural adjustments impact the processing of sulfur cathode films for pouch cell SSBs.^1036^

The tortuosity of composite SSB electrodes is generally higher than in liquid electrolyte batteries since liquids can wet all void areas. Tortuosity limitations can be minimized by using SSEs and active materials with smaller particle sizes, by using SSEs with high ionic conductivity, and by using high-energy ball-milling during composite preparation.^1037,1038^ The particle size of the various constituents is another parameter that can be controlled to enhance ionic percolation pathways in composite cathodes (Figure 59b).^1032,1038^ NMC622 particles less than 10 μm in size have been shown to achieve theoretical capacity in the initial cycle when used with LPS SSE, and XRD revealed inactive NMC material with higher particle sizes.^1039^ Work by Schlautmann et al. combined XRD, PDF, and microstructural modeling to show that SSEs with smaller particle size in NMC composite cathodes result in favorable charge transport and increased interfacial area between the active material and SSE.^1040^ The morphology of the SSE is another factor that affects the transport properties and electrode behavior. Improved ionic percolation pathway have been demonstrated through the development of vertically aligned Li_0.35_La_0.35_TiO_3_ (LLTO) nanowires within composites, showing enhancements of ionic conductivity by a factor of 2.^1041^

Carbon additives improve the electronic conductivity of cathode composites and may help to mitigate delamination effects.^134^ The type, particle size, and structure of the C additive affect electrochemical behavior.^1042^ Carbon black, single-walled carbon nanotubes (CNTs), and hybrid additives have been investigated and compared, with carbon black showing good contact but lower electronic conductivity than CNTs.^1043−1045^

Minimizing SSE content can improve the energy density of cells but must be balanced with the possibility of ion transport limitations within the electrode composite.^1046,1047^ Zahiri et al. demonstrated a dense LiCoO_2_ (LCO) cathode formed via electrodeposition onto a current collector with controlled crystallographic orientation at the SSE interface. The LCO layer featured the (003) basal plane oriented perpendicular to the substrate, and the electrodes were characterized with FIB-SEM techniques. The materials exhibited excellent Li-ion accessibility and interfacial kinetics, and the highly dense material could provide for high energy density.^9^

Conversion-type materials such as S and Li_2_S are electronic insulators, necessitating copious use of electronic additives.^1048−1052^ The addition of carbon additives creates a triple-phase boundary that detrimentally affects ionic transportation pathways and increases overpotential.^172,1053^ Bradbury et al. investigated ion transport within S composite cathodes with *operando* neutron imaging and reported residual Li close to the current collector and the formation of an inhomogeneous interface.^412^ Instead of using S, using metal sulfide active materials that form metal discharge products may enhance electronic conductivity and reduce the need for C additives.^1054^ Yu et al. investigated CuS:LPSC composite electrodes, using *ex situ* XRD and XPS to confirm the conversion reaction of CuS CAM.^1055^ In general, the microstructural evolution of conversion cathodes in SSBs differs from that of intercalation materials.^1056−1059^ More work is needed to understand microstructural evolution of conversion materials and how microstructural degradation influences transport and electrochemical reversibility.

#### 7.1.2 Stack Pressure Effects

As previously discussed, external stack pressure is often used in SSB investigations to improve contact between composite components and to preserve ionic conduction pathways. The stack pressures used in academic investigations vary widely from sub-MPa to several hundreds of MPa.^160,1060^

**Figure 60:**
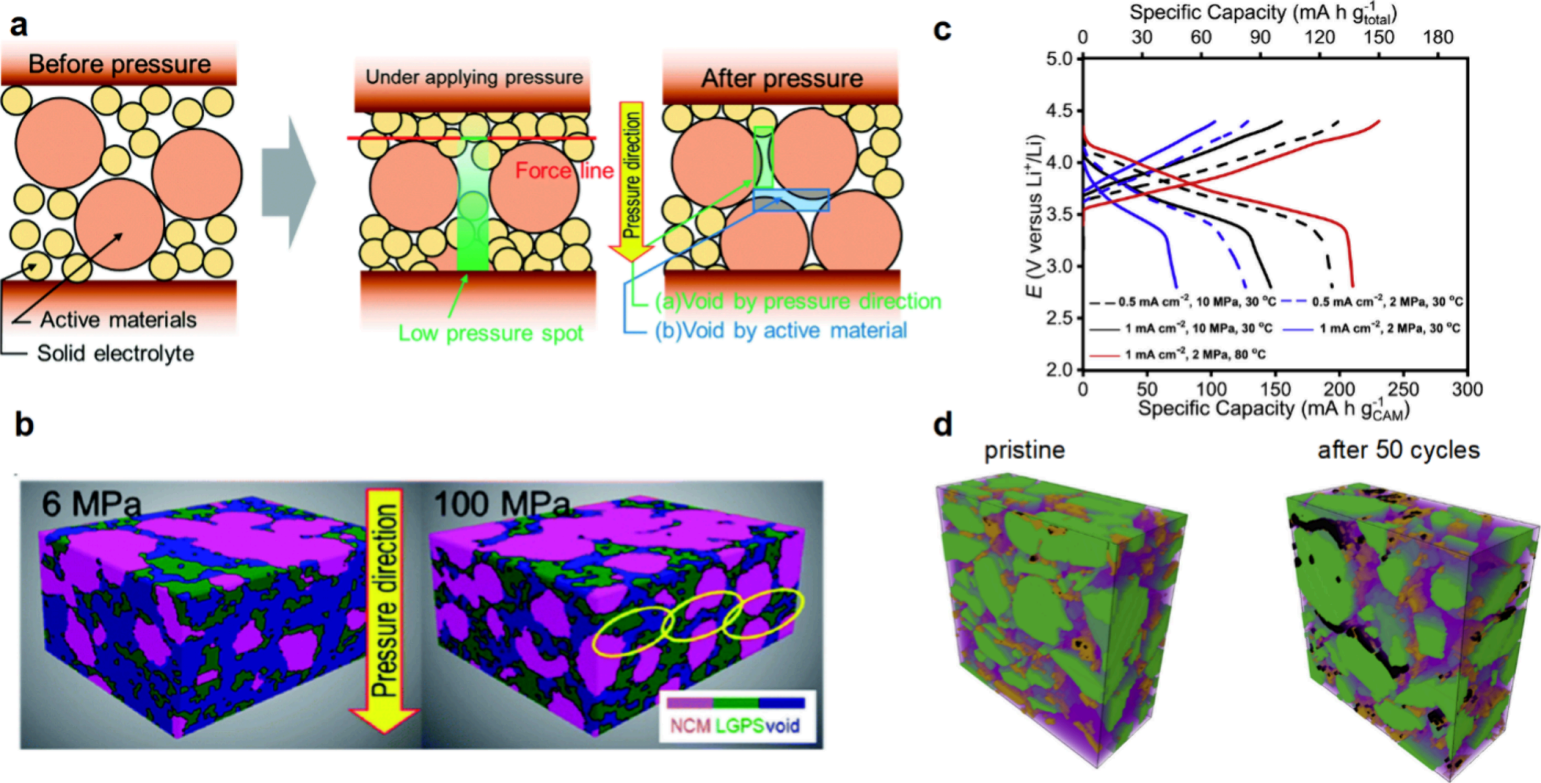
Stack pressure effects on composite cathodes. (a) Schematics that highlight microstructural changes of composite electrodes under uniaxial pressure. (b) Reconstructed 3D XCT images of NMC cathode composites that highlight the effect of different stack pressures on the contact area between constituents. (a, b) Reproduced with permission from ref (390). Copyright 2022 Royal Society of Chemistry. (c) Stack pressure and temperature dependent voltage-capacity curve of LiNi_0.83_Mn_0.06_Co_0.11_O_2_, Li_3_InCl_6_, and carbon nanofiber composite cathode. (d) Reconstructed plasma-FIB tomographic images of cathode composite before and after 50 cycles (green: NMC, purple: SSE, orange: CNF, and black: void). The reconstructed 3D structure volume is 5.8 μm × 5.8 μm × 2.5 μm. (c, d) Adapted with permission from ref (73). Copyright 2022 Elsevier.

Although increased stack pressure often has a favorable effect on cell performance,^1010^ the application of uniaxial stress during fabrication and under cell operation can cause irregular strains and particle agglomeration.^790^Figure 60a schematically shows how stack pressure can affect the microstructure of cathode composites. Voids and particle size discrepancies can lead to low-pressure regions of the electrode, detrimentally affecting contact at active material interfaces.^390^ Sakka et al. monitored the effect of stack pressure on the performance of composite cathodes using XCT. Cathode composites that include NMC and LGPS exhibited stack pressure-dependent microstructure evolution, with void formation reduced by a factor of 2 when the stack pressure increased from 6 to 100 MPa (Figure 60b).^390^ Other work has found that the low-volume-change LTO electrode material can be stably cycled at stack pressures as low as 0.1 MPa, but that voids formed in the counter electrode can cause degradation of the cell.^1061^ Extensive work by Gao et al. demonstrated the optimization of NMC-based cathode composites for use at 2 MPa stack pressure (Figure 60c).^73^ Plasma-FIB SEM investigations showed retained contact between constituents in the composite after 50 cycles (Figure 60d).

#### 7.1.3 Effect of SSE Properties

The ionic conductivity, mechanical properties, electrochemical stability, and thermal characteristics of the SSE within an electrode composite significantly influence the cycling stability, energy density, and the rate capability of the cell. The electrochemical compatibility of the SSE with the active material influences the interfacial chemical and structural stability, and thus electrochemical performance. High-voltage cathode materials such as NMC may require SSEs with wide stability windows and intrinsic resistance to oxidation, or interfacial coatings. Here, the electrochemical compatibility of sulfide, oxide, and halide-based SSEs with cathode active materials is discussed. The details of electrochemical stability windows of SSEs were previously discussed in Sections 3 and 5.

##### 7.1.3.1 Sulfides

Sulfide SSEs have been heavily investigated due to their high ionic conductivity at room temperature. However, many sulfides feature narrow electrochemical stability windows, with oxide cathode materials falling outside the windows. This can result in the oxidation of sulfide SSEs in contact with NMC and other active materials, causing side reactions and forming interphases.^442,1062−1064^ Using XPS, Jung et al. reported the formation of byproducts after contact between NMC and LPSC.^1064^ Oxygen release from active materials at high potentials can also cause a detrimental interfacial layer with high resistance. Walther et al. investigated the degradation of the NMC622 cathode material in contact with LPSC SSE using ToF-SIMS and XPS. XPS detected the formation of polysulfides and showed that the interphase layer was less than 10 nm thick.^442^ Banerjee et al. reported the oxidation of LPSC and the formation of NiS_2_ or Ni_3_S_4_ species as decomposition products resulting from SSE–NCA cathode interactions.^1065^

Sulfide SSEs have been shown to exhibit reversible redox activity when C is blended in the composite. Zhu et al. investigated redox reactions of the Li_7_P_3_S_11_ SSE, revealing the formation of Li_2_S and Li_3_P during discharge and S and P_2_S_5_ species during charging.^1066^ Formation of these species can add “extra” capacity to measurements of Li–S SSBs, but they cause faster SSE degradation.^1051^ Similar redox reactions have been characterized for other sulfide electrolytes including LGPS and LPSC.^1053,1067,1068^

**Figure 61:**
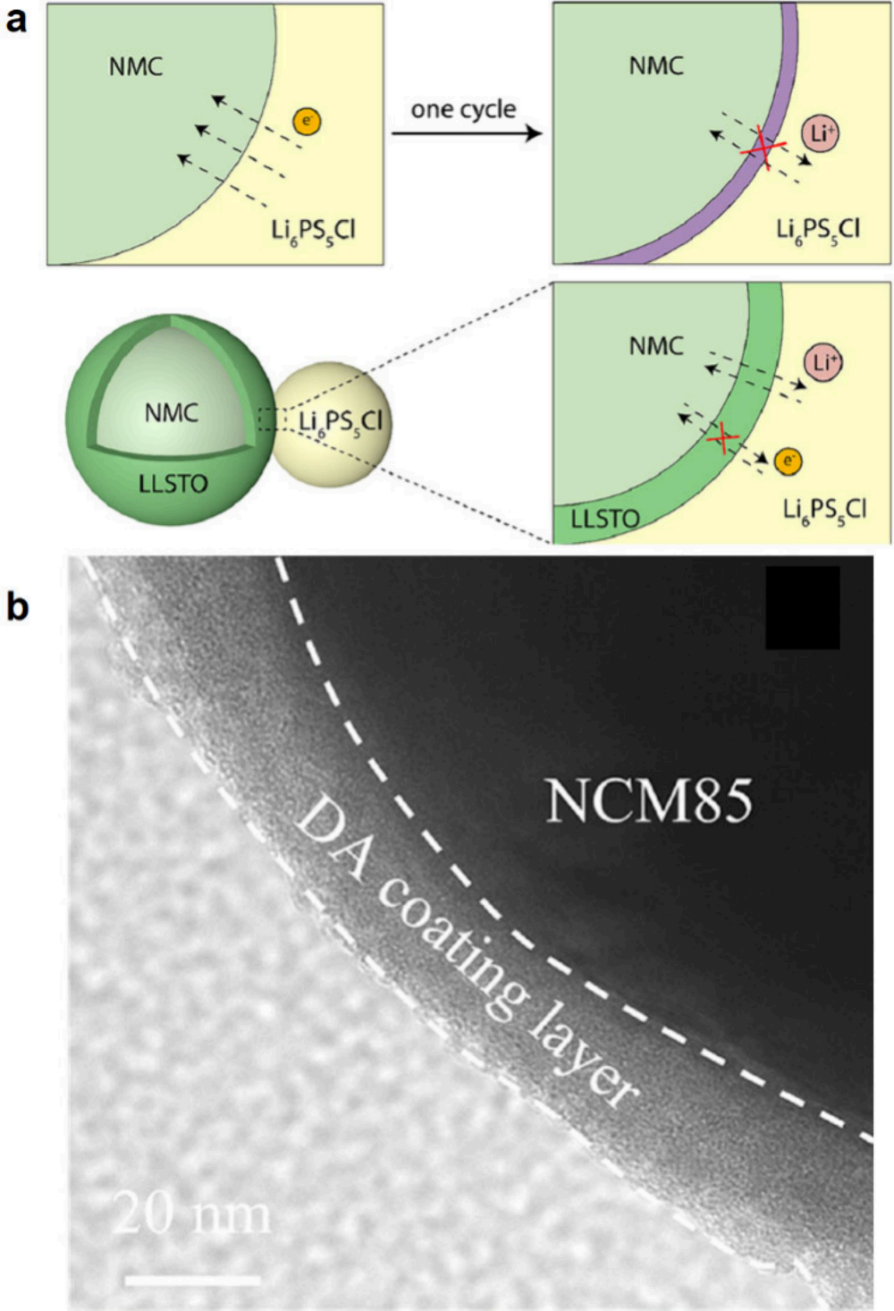
(a) Schematic of interphase engineering through surface coating of NMC cathode. Reproduced with permission from ref (490). Copyright 2020 American Chemical Society. (b) TEM image of decanoic acid-coated LiNi_0.85_Co_0.1_Mn_0.05_O_2_. Reproduced with permission from ref (663). Copyright 2023 American Chemical Society.

Several strategies have been investigated to promote interfacial stability between sulfide SSEs and cathode active materials. Coatings of oxide active materials with electronically insulating and ionically conducting interlayer materials (e.g., LiNbO_3_, Li_2_ZrO_3_, Li_1+*x*_Al_*x*_Ti_2–*x*_(PO_4_)_3_ (LATP), and Li_4_Ti_5_O_12_) have been widely investigated and shown to improve stability (Figure 61a).^490,550,1069−1076^ Coating NCA with Li_3_InCl_6_ using a mechanofusion technique was shown to suppress side reactions between LPSC and NCA at higher voltages, mitigate delamination and particle cracking, and enhance specific capacity by 80 mAh g^–1^.^1077^ Instead of coating with interlayers, Zuo et al. mixed active materials with SSE and annealed the composite to form Li_2_SO_4_ and Li_3_PO_4_ at the interface, as shown with XPS.^1078^ Carboxyl groups in organic acids have been reported to stabilize the cathode interface in Li-ion batteries by reacting with Li in NMC and forming an organo-lithium layer,^1079^ and a similar strategy was recently applied to SSB cathode composites. The treatment of NMC with decanoic acid stabilized the cathode–electrolyte interface up to potentials of 4.3 V vs Li/Li^+^ (Figure 61b).^663^ Although coatings can improve stability, they can also interdiffuse and become chemo-mechanically damaged.^550^ More investigation is needed into the structural and chemical evolution of coating layers for composite cathodes.

##### 7.1.3.2 Oxides

Garnet-type Li_7_La_3_Zr_2_O_12_ (LLZO) and its various doped derivatives have attracted attention because of their air processability, high ionic conductivity up to 2 mS cm^–1^, and their compatibility with Li metal anodes.^1080^ However, integration of LLZO within composite cathodes poses several challenges. The main difficulty is that sintering of LLZO and the cathode active material is needed to ensure contact between materials, but this sintering can cause chemical reactions and interfacial instabilities.^1081^ Understanding the evolution of such composite microstructures during processing and sintering is needed. XRD, electron microscopy, EELS, EDS, and EBSD have been used to investigate the structure and morphology of multiphase LLZO–SSE composites.^1080,1082^

Various parameters, including the nature of dopants, processing conditions, and pressure can affect microstructure formation of LLZO composite electrodes.^512,1083−1086^ The sintering temperature can control the nature of interfacial product phases. XRD and Raman techniques have been used to show that Ta-doped LLZO is chemically compatible with various cathode materials.^1087^ The authors reported reactions of LFP and LMO with the LLZO at 500 °C, while NMC and LCO were found to react at 700 °C. LaCo_1–*x*_Mn_*x*_O_3_ and LaCoO_3_ were found as reaction products. Kim et al. reported a ∼50 nm thick reaction layer comprising La_2_CoO_4_ at the LCO–LLZO interface using TEM and nanobeam electron diffraction (NBD).^1088^ Using synchrotron XRD and XAS, Vardar et al. reported that the interface between LCO and Al-doped LLZO evolves to form La_2_Zr_2_O_7_ and LaCoO_3_ at 300 °C and Li_2_CO_3_ at 500 °C.^1089^ TOF-SIMS has been used to identify the tetragonal LLZO phase at the LCO–LLZO interface that reduces electrochemical reversibility.^492^ The interface between various NMC materials and LLZO has also been investigated. The reaction products of LLZO and NMC are LaNiO_3_, LaCo_1–*x*_Mn_*x*_O_3_, LaCo_1–*x*_Ni_*x*_O_3_, and others.^1087^ Kim et al. investigated various heating environments and concluded that the SSE and NMC622 sintered in O_2_ environment has better chemical stability and lower interfacial resistance.^1090^

Stress and strain evolution due to thermal expansion during sintering is another factor that poses a challenge to the development of oxide SSE electrode composites. The mismatch of the thermal expansion coefficients (CTEs) for NMC and LLZO results in stress and strain during heating,^1091^ which can lead to cracks and increased interfacial resistance. Tsai et al. reported an absence of secondary phases when the active material and SSE have comparable CTE values, as confirmed by micro-Raman and EDS analysis. This is because elemental interdiffusion and secondary phase growth is prevented with faster sintering at higher temperatures, which is enabled when the SSE and active material have similar CTE values.^1092^ In general, understanding thermal stresses in composite cathodes requires accurate estimates of CTE values. DFT has been used to calculate such values,^1093,1094^ and temperature-dependent XRD and dilatometry can also be used to measure CTE values.^1095−1097^

The charge transfer resistance of the LCO–LLZO interface has been reported to be between 1 and 5 kΩ cm^2^ and is highly dependent on processing and the nature of the interphase.^1080^ Reducing this interfacial resistance is a key goal of composite development. Additive materials, such as Li_3_PO_4_^468^ and Li_3_BO_3_/Li_3_BO_2_,^1098−1100^ could inhibit interdiffusion and enhance contact between the active material and LLZO. Advanced sintering techniques have also been proposed, such as high-pressure field assisted sintering^1101,1102^ and fast sintering.^1103^ The influence of various sintering strategies on microstructural evolution and the formation of grain boundaries requires more investigation.

##### 7.1.3.3 Halides

**Figure 62:**
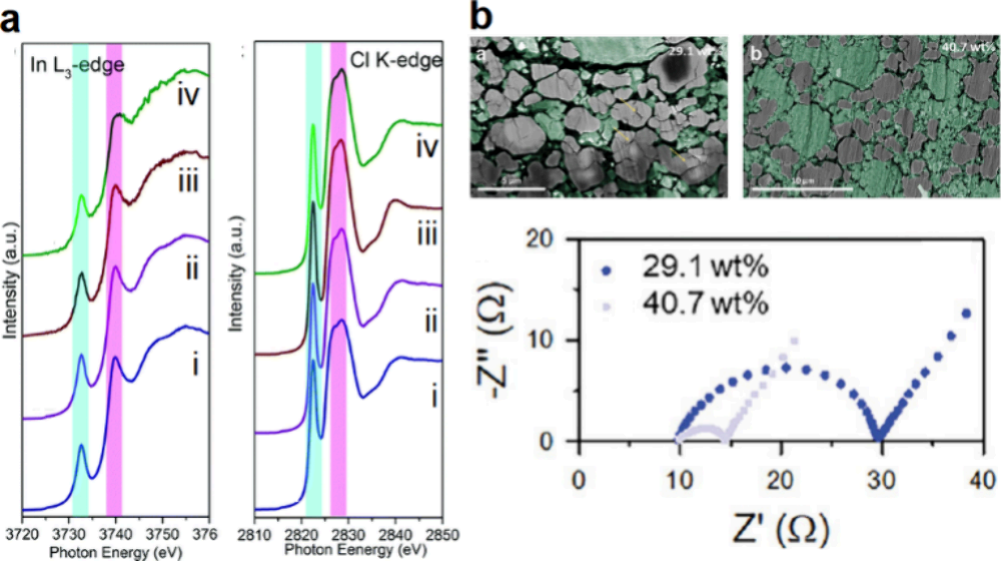
Stability and compatibility of halide SSEs with cathode active materials. (a) In L-edge and Cl K-edge XANES spectra of (i) a pristine Li_3_InCl_6_ SSE, as well as a LiCoO_2_–Li_3_InCl_6_ composite cathode (ii) in the pristine state, (iii) after the first charge, and (iv) after the first discharge. Reproduced with permission from ref (67). Copyright 2019 Royal Society of Chemistry. (b) SEM images of 29.1 and 40.7 wt % LYC–NCA composites and Nyquist plots of two composites. Adapted with permission from ref (1104). Copyright 2021 Wiley-VCH GmbH.

Halide SSEs generally feature better stability at high potentials compared to sulfides and thus do not require the use of coating layers when used with oxide cathode active materials.^65,72^ Asano et al. investigated the use of Li_3_YCl_6_ and Li_3_YBr_6_ SSEs in conjunction with LCO and reported 94% initial CE, suggesting good interfacial stability.^66^ Li et al. used XANES to probe composites of Li_3_InCl_6_ SSE and LCO, and the In L-edge and Cl K-edge from the SSE preserved its features after charge and discharge (Figure 62a).^67^ Han et al. compared NCA compatibility with sulfide and halide electrolytes, finding that the halides exhibited improved interfacial compatibility and that the capacity retention with cycling of the NCA composite was dependent on Li_3_YCl_6_ content (Figure 62b).^1104^ A downside of halides is that their ionic conductivity is typically lower than sulfides.^42,71,72^

##### 7.1.3.4 Charge Effects

**Figure 63:**
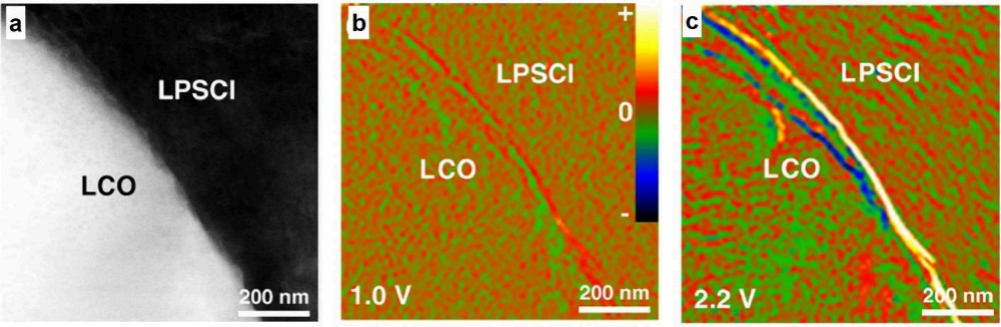
Effect of space charge layer on the SSE-active material interface. (a) HAADF-STEM image of LCO–LPSC interface. (b, c) *In situ* differential phase contrast STEM observation of net-charge-density accumulation at LCO–LPSC interface at applied voltages of (b) 1.0 V and (c) 2.2 V. The color bar represents positive (yellow) and negative (black) charge density. Adapted with permission from ref (1114). Copyright 2020 Nature Springer.

The space charge layer (SCL) is a region of nonuniform charge distribution that is formed when two materials with different chemical potentials of a species are contacted. The chemical potential of Li in sulfide SSEs is substantially higher than cathode materials such as Ni-rich NMC. Although the presence of space charge layers in Li-based SSBs is somewhat controversial,^472,1105−1109^ an electric field exists at such interfaces which can influence ion transfer and impedance.^1029,1105,1110,1111^ Direct observation of SCLs in SSBs has been limited, but several experimental works have investigated the influence of gradients at interfaces via indirect characterization using techniques including XPS,^1112^ ss-NMR,^1108^ Kelvin probe force microscopy,^472^*in situ* electron holography,^1113^ STEM,^1114^ and *operando* Raman spectroscopy.^1115^ Wang et al. investigated the charge density across an LCO–LPSC interface with *in situ* differential phase contrast STEM and observed Li-poor and Li-rich regions in the vicinity of the SSE and active material, respectively (Figure 63).^1114^ Overall, investigating how ions and electric fields interact at various SSB interfaces can enhance our understanding of kinetics and ion transport pathways.

### 7.2 Chemo-Mechanics of Composite Cathodes for SSBs

#### 7.2.1 Volume Change

Most cathode active materials undergo volumetric changes during ion insertion and extraction, ranging from −5–9% for intercalation materials to ∼100% for conversion materials.^171,173,582,1116−1118^ Volume change within a composite can lead to contact loss between the active material and SSE and correspondingly higher interfacial resistance.^171,1119,1120^ Understanding of and control over composite electrode evolution during active material volume change are thus key areas of focus in SSB characterization and development.

**Figure 64:**
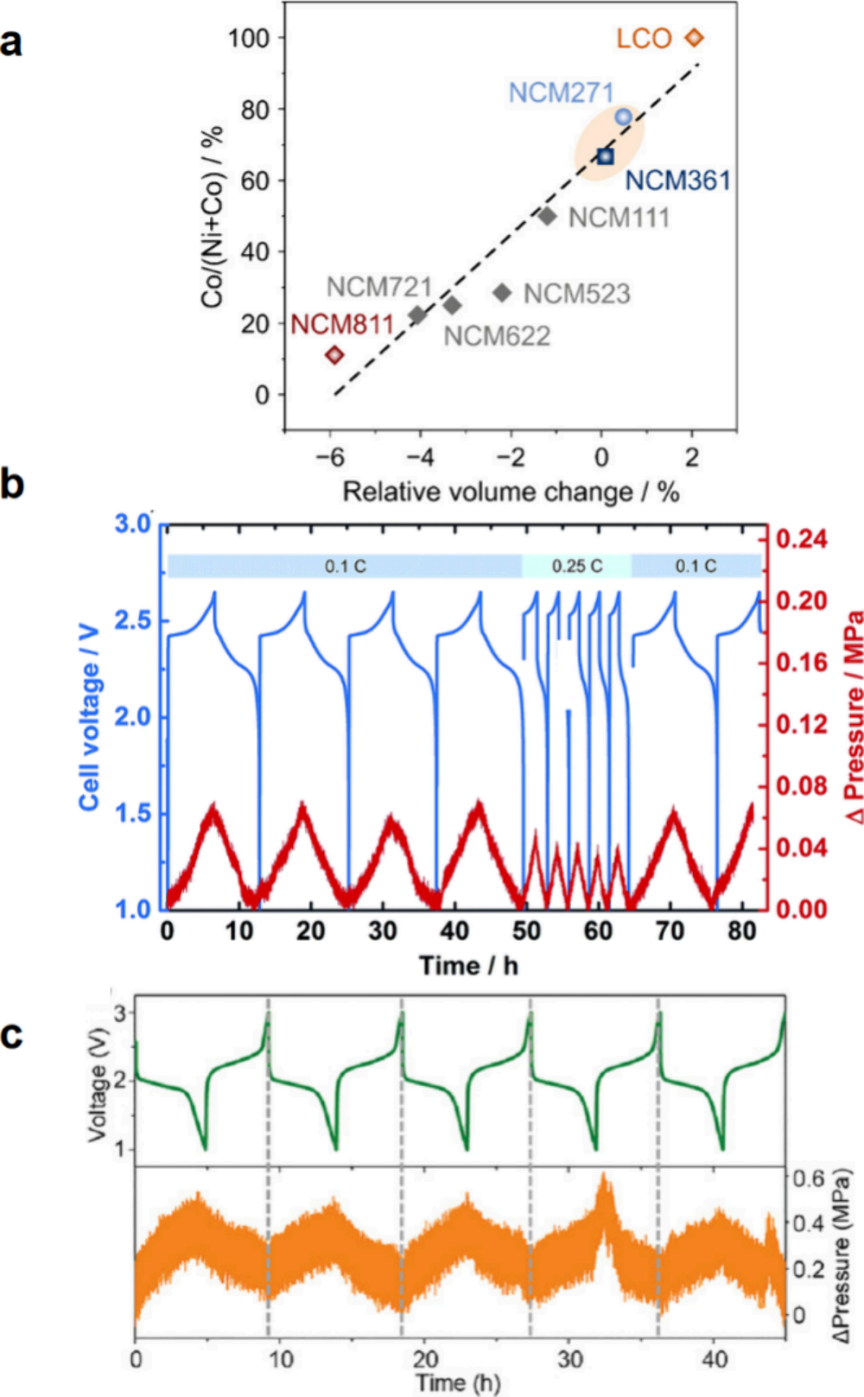
Volume change in cathode active materials. (a) Relative volume change of NMC cathodes during charging with respect to Co and Ni composition. Data obtained from XRD measurements. Reproduced with permission from ref (173). Copyright 2020 American Chemical Society. (b) *In situ* pressure monitoring of a Li_4_Ti_5_O_12_/Li_10_GeP_2_S_12_/LiCoO_2_ cell. Reproduced with permission from ref (300). Copyright 2017 Royal Society of Chemistry. (c) *In situ* pressure monitoring of a Se-Li_3_HoCl_6_-C/Li_3_HoCl_6_/Li SSB. Reproduced with permission from ref (1124). Copyright 2022 Wiley-VCH GmbH.

The lattice expansion and contraction in NMC is directly proportional to Ni content, and Ryu et al. previously demonstrated the effect of volume change on capacity retention in NMC in Li-ion batteries.^1121^ In general, materials with negligible volume expansion or contraction during cell cycling show the potential for improved electro-chemo-mechanical stability over time.^173,1122,1123^ Design of zero-strain cathode active materials has been performed by composition screening and evaluation of lattice parameters. Strauss et al. screened various NMC materials that undergo volume changes from −7% to +2% (Figure 64a).^173^ Based on the *a* and *c* lattice parameters and stress measurements, Co-rich (70% Co) NMCs were found to exhibit minor volume changes during battery operation.

The effects of cathode composite volume change on stress evolution can be monitored with a force sensor integrated into the battery cell, as well as with other methods. Zhang et al. investigated volume expansion and contraction properties of In|Li_10_GeP_2_S_12_|LiCoO_2_ cells using a dilatometer and *in situ* pressure sensor, observing continuous increments and decrements in height during charging and discharging that indicate electrode expansion and contraction (Figure 64b).^300^ Koerver et al. comprehensively investigated the chemo-mechanical behavior of a wide range of cathode materials for SSBs and suggested tailoring mechanical properties by blending cathode composites that possess opposite volume change during cycling. Pressure evolution is minimized by blending LCO and NMC cathode materials.^171^

The volume change of conversion cathode materials is greater than intercalation-type cathode materials. For instance, the conversion reaction of S to form Li_2_S results in 80% volume expansion,^1125,1126^ and the volume expansion of Se to form Li_2_Se is 98%.^1118^ Li et al. investigated the pressure evolution of a SSB cell with a Se-based cathode and observed approximately 0.5 MPa pressure change during cell cycling (Figure 64c).^1124^ Similar work has been performed on CuS cathode materials.^1056^

#### 7.2.2 Particle Fracture and Void Formation

The presence of voids and cracks in SSB cathode composites is common even when applying very high formation pressures (hundreds of MPa) during cell assembly. The presence of cracks and voids within the composite affects ion transport and can increase impedance. As a result of the relatively large volume changes of conversion-type and Ni-rich intercalation-type cathodes, cracks within active particles themselves have been observed.^174,653,1127,1128^ Fracture of active materials can also occur due to application of uniaxial pressures during cell fabrication.^1129^ In contrast to batteries with liquid electrolytes, cracked particles in SSBs inhibit transport since the SSE does not flow to wet internal surfaces like liquid electrolytes.^1130−1133^

**Figure 65:**
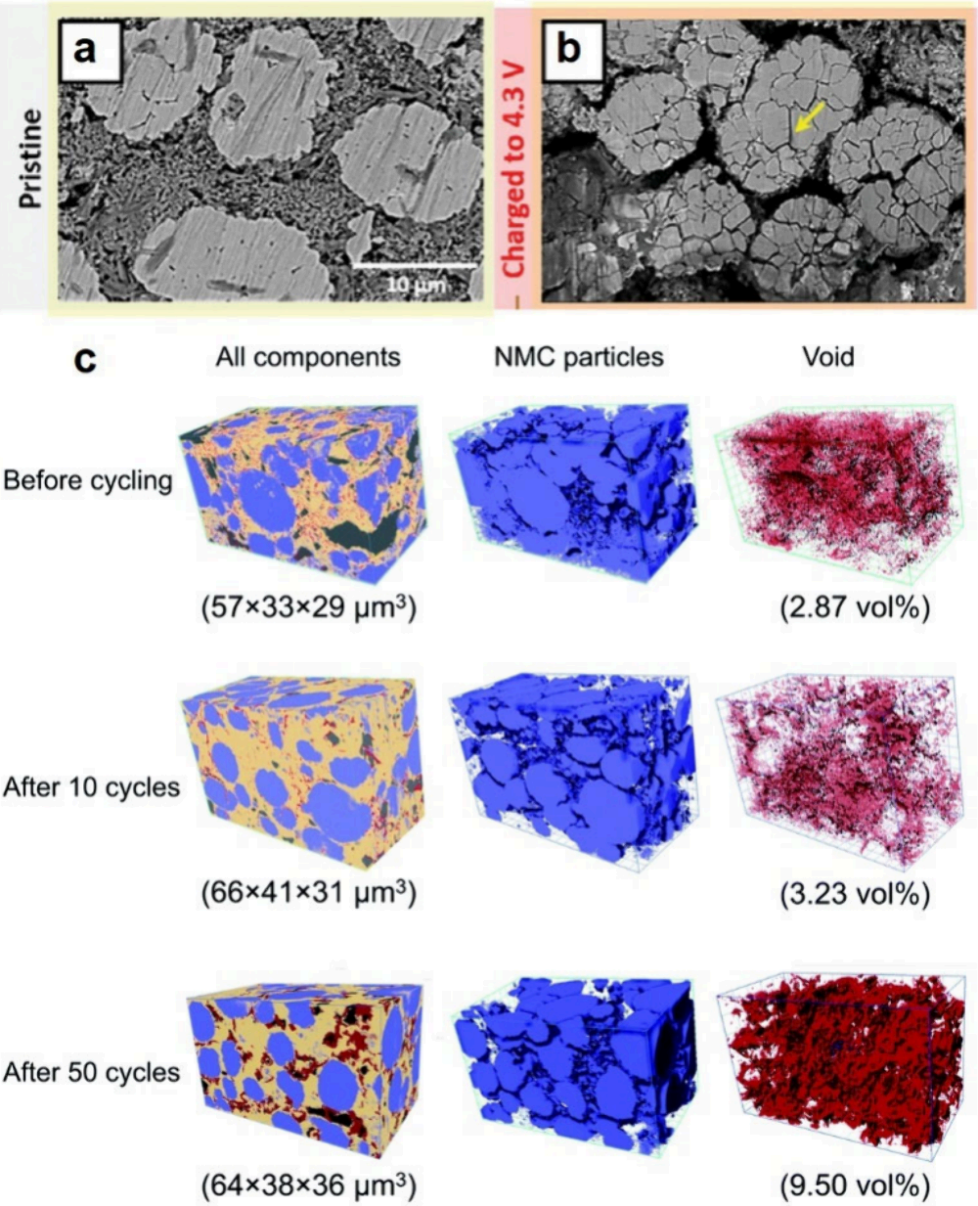
Formation of cracks and voids in cathode composites for SSBs. Cross-sectional SEM images of (a) pristine Li[Ni_0.8_Co_0.16_Al_0.04_]O_2_ and (b) an electrode after charge to 4.3 V vs Li/Li^+^, where yellow arrows indicate formation of intergranular cracks. (a, b) Reproduced with permission from ref (1133). Copyright 2019 WILEY-VCH Verlag GmbH & Co. KGaA. (c) FIB-SEM tomography of NMC composites in the pristine state (upper panel), after 10 cycles (middle panel), and after 50 cycles (bottom panel). Reproduced with permission from ref (294). Copyright 2020 Royal Society of Chemistry.

The formation of microcracks or voids can be characterized with various imaging and spectroscopic techniques, and their effects are often correlated to electrochemical behavior by evaluating capacity retention, ionic conductivity, and charge transfer resistance. Crystallographically controlled active materials could potentially mitigate the formation of cracks since ion transport can be influenced by grain boundaries and surfaces.^1134^ Single-crystalline cathode active materials have been widely shown to be useful for improving electrochemical and chemo-mechanical stability in SSBs.^1135−1138^ Jung et al. investigated the chemo-mechanical properties of randomly oriented grains of Li[Ni_0.80_Co_0.16_Al_0.04_]O_2_ and radially oriented grains of Li[Ni_0.75_Co_0.10_Mn_0.15_]O_2_ active material. Cross-sectional SEM imaging revealed the formation of intergranular cracks and voids after the first charge (Figure 65a,b).^1133^ FIB-SEM tomography is a useful technique to evaluate void and crack formation within composite cathodes.^294,1139^ Shi et al. investigated the chemo-mechanical degradation process of cathode composites that consisted of NMC532, LPS SSE, and carbon nanofiber. The authors reported cracks and voids near cathode particles after 50 cycles (Figure 65c).^294^

## 8 Conclusion

This paper has presented an overview of characterization methods used to investigate materials and interfaces in SSBs, and we have discussed the current state of knowledge of the mechanisms that govern the behavior of various electrode materials and their interfaces. Key differences of the material and interface evolution between SSBs and liquid-electrolyte batteries were discussed. This set the stage for a comprehensive overview of characterization techniques that have been applied to SSB systems, including imaging, scattering, spectroscopy, and other techniques. Differences among *ex situ*, *in situ*, and *operando* techniques were presented, along with the unique aspects of SSBs that determine the characterization strategy needed to answer a given scientific question. Our current understanding of lithium metal anodes in SSBs was presented in detail, which has primarily been acquired from electrochemical, mechanical, and imaging techniques. The operational mechanisms and degradation modes of alloy anodes and composite cathodes were also discussed.

Driven by a fundamental understanding of the evolution of materials and interfaces, SSB research has progressed significantly over the past decade. However, there are critical questions and characterization needs that have yet to be addressed. These future directions have been highlighted throughout this paper, and some of these next frontiers for SSB characterization are discussed here.

An area of need is the systematic characterization of interphase growth in SSB systems. The properties of the SEI in liquid-electrolyte batteries have attracted enormous attention,^135−138,231^ but much less is known about interphase characteristics in SSBs. Important prior work detailed in Sections 3 and 5 has provided a framework for understanding the thermodynamics of interphases at SSE-lithium interfaces, but kinetics-driven factors require more detailed investigation. Characterization of the distribution of phases and their transport properties within interphase layers of various SSEs in contact with lithium is needed. Investigating interphase growth over long times in SSEs that are nominally “kinetically stable” in contact with lithium is also necessary to understand the ultimate stability of SSBs. Understanding interphase growth within SSEs in contact with other negative electrodes beyond lithium is of interest, as little is known about the chemistry, structure, and properties of interphases that form at alloy anodes, for instance. Furthermore, characterization methods to systematically investigate the effects of stack pressure and temperature on interphase growth are needed. Void formation and contact loss at the alloy anode and SSE interfaces, typically characterized using *ex situ* methods, requires *operando* or *in situ* techniques for better tracking of dynamic evolution.

Considering image-based characterization of SSBs, there have been numerous important *in situ* and *operando* studies that have revealed electrode and interface behavior at the microscale, including X-ray imaging and optical microscopy.^133,315,355,375^ There have also been a handful of nanoscale TEM studies that have investigated interphase growth between lithium and various SSEs.^127,132,512,1140^ Overall, however, efforts to uncover nanoscale dynamics *in situ* at SSB interfaces have lagged behind investigations at greater length scales. This may be at least partially due to the challenges of using TEM and related methods to investigate SSEs, which tend to be beam-sensitive due to the high mobility of Li^+^ in the materials. Regardless, we lack understanding of nanoscale phenomena such as the early dynamics of void formation and filament initiation for lithium metal anodes. Creative approaches to uncovering nanoscale interfacial dynamics in SSBs should be a goal of the field.

Future research should focus on understanding elemental and morphological changes at electrode–electrolyte interfaces and how these changes contribute to cell failure mechanisms. However, the scarcity of access to synchrotron facilities for advanced techniques such as XCT and XAS limits progress. To mitigate this, developing capabilities using lab-scale XAS and XCT instruments would allow for more rapid and potentially more practical analysis. Neutron-based techniques such as NDP may also offer opportunities for overcoming the limitations of X-ray-based methods, potentially offering deeper insights into lithium stripping/deposition and other phenomena.

It would be useful if the design of *operando* and *in situ* SSB cells received more systematic attention in the literature, as current studies often omit essential details, precluding reproducibility. Furthermore, the electro-chemo-mechanical behavior of SSBs is highly sensitive to specific experimental conditions such as cell housing design, applied pressure, and atmospheric conditions during assembly. For comparability among studies, it is essential that these details are consistently reported, which will help standardize SSB technologies and guide future advancements.

Further progress is needed toward characterizing the evolution of materials and interfaces in SSBs under practically relevant testing conditions, and especially under low stack pressures (<1 MPa). This is a challenge since most literature testing of various SSBs takes place at higher stack pressures because of the performance benefits of these conditions. However, divergent material mechanisms may control behavior at lower stack pressures, and such low-pressure regimes are critical to understand for translation of SSBs to applications. Such efforts will require careful control and design of cells for characterization, as well as increased cooperation among groups (both in academic research and industry) to share best practices for material processing and investigation. In addition, investigation of practical cell formats such as pouch cell architectures with thin SSEs at relevant current densities is important to ensure the practical relevance of new findings.

Overall, the characterization of electrode materials and their interfaces has been a critical aspect of SSB development over the past decade. Many characterization techniques that were originally developed or used for Li-ion batteries have been modified to uncover the behavior of SSB systems, and SSB development has been accelerated because of our knowledge of Li-ion batteries. The move from solid–liquid to solid–solid interfaces is a paradigm shift, however, and continued fundamental investigations tied to engineering development are needed for rapid advancements. With a dedicated focus on these aspects, we look forward to a bright future for SSBs.

## List of Abbreviations

2D EXSY: Two-dimensional exchange spectroscopy
ABF: Annular bright field
AC: Alternating current
ADF: Annular dark field
AFM: Atomic force microscopy
ALD: Atomic layer deposition
ARC: Accelerated rate calorimetry
BMS: Battery management system
CAM: Cathode active material
CBED: Convergent beam electron diffraction
CE: Coulombic efficiency
CEI: Cathode electrolyte interface
CNF: Carbon nanofiber
CPD: Contact potential difference
Cryo-FIB: Cryogenic focused ion beam
CT: Computed tomography
CTE: Coefficient of thermal expansion
CV: Cyclic voltammogram
DC: Direct current
DEMS: Differential electrochemical mass spectrometry
DF: Dark field
DFT: Density functional theory
DIC: Digital image correlation
DSC: Differential scanning calorimetry
EBSD: Electron backscatter diffraction
EDS or EDX: Energy dispersive X-ray spectroscopy
ED-XRD: Energy dispersive X-ray diffraction
EELS: Electron energy loss spectroscopy
EH: Electron holography
EIS: Electrochemical impedance spectroscopy
EPR: Electron paramagnetic resonance
EXAFS: Extended X-ray absorption fine structure
FBG: Fiber Bragg grating
FIB: Focused ion beam
FT-IR: Fourier transform infrared spectroscopy
GITT: Galvanostatic intermittent titration technique
HAADF: High-angle annular dark field
HAXPES: Hard X-ray photoelectron spectroscopy
HR-TEM: High-resolution transmission electron microscopy
ic-ac-SECM: Intermittent contact alternating current scanning electrochemical microscopy
KPFM: Kelvin probe force microscopy
LAGP: Lithium aluminum germanium phosphate (Li_1+*x*_Al_*x*_Ge_2–*x*_(PO_4_)_3_)
LATP: Lithium aluminum titanium phosphate (Li_1+*x*_Al_*x*_Ti_2–*x*_(PO_4_)_3_)
LCO: Lithium cobalt oxide (LiCoO_2_)
LCP: Lithium cobalt phosphate (LiCoPO_4_)
LFP: Lithium iron phosphate (LiFePO_4_)
LGPS: Lithium germanium phosphorus sulfide (Li_10_GeP_2_S_12_)
LIB: Lithium-ion battery
LiBOB: Lithium bis(oxalate) borate
LIC: Lithium indium chloride (Li_3_InCl_6_)
LiPON: lithium phosphorus oxynitride
LISICON: Lithium superionic conductor
LiTFSI: Lithium bis(trifluoromethanesulfonyl)imide
LLTO: Lithium lanthanum titanate (LiLaTiO_3_)
LLZO: Lithium lanthanum zirconate (Li_7_La_3_Zr_2_O_12_)
LLZTO: Lithium lanthanum tantalum zirconate (Li_6.4_La_3_Zr_1.4_Ta_0.6_O_12_)
LNMO: Lithium nickel manganese oxide (LiNi_*x*_Mn_*y*_O_4_)
LNO: Lithium nickel oxide (Li_1–*x*_Ni_1+*x*_O_2_)
LPS: Lithium phosphorus sulfide (Li_7_P_3_S_11_)
LPSX: Argyrodite-type solid ionic conductor (Li_6_PS_5_X X = Cl, Br, I)
LSPS: Lithium tin phosphorus sulfide (Li_10_SnP_2_S_12_)
LTO: Lithium titanate (Li_4_Ti_5_O_12_)
LYC: Lithium yttrium chloride (Li_3_YCl_6_)
MAS: Magic angle spinning
MEMS: Micro-electro-mechanical system
MIEC: Mixed ionic-electronic conductor
MOSS: multibeam optical stress sensor
MRI: Magnetic resonance imaging
MS: Mass spectrometry
MWCNT: Multiwalled carbon nanotube
NASICON: Na superionic conductor
NCA: Lithium nickel cobalt aluminum oxide (LiNi_0.8_Co_0.15_Al_0.05_O_2_)
ND: Neutron diffraction
NDP: Neutron depth profiling
NMC: Lithium nickel manganese cobalt oxide (LiNi_*x*_Co_*y*_Mn_*z*_O_2_)
NMC-111: Lithium nickel manganese cobalt oxide (LiNi_0.33_Co_0.33_Mn_0.33_O_2_)
NMC-532: Lithium nickel manganese cobalt oxide (LiNi_0.5_Co_0.2_Mn_0.3_O_2_)
NMC-622: Lithium nickel manganese cobalt oxide (LiNi_0.6_Co_0.2_Mn_0.2_O_2_)
NMC-811: Lithium nickel manganese cobalt oxide (LiNi_0.8_Co_0.1_Mn_0.1_O_2_)
NMR: Nuclear magnetic resonance
N-PDF: Neutron pair distribution function
OIPC: Organic ionic plastic crystal
PDF: Pair distribution function
PEEK: Polyether ether ketone
PEO: Poly(ethylene oxide)
PFG: Pulsed field gradient
PFIB: Plasma focused ion beam
PTFE: polytetrafluoroethylene
PVDF: Poly(vinylidene fluoride)
QENS: Quasi-elastic neutron scattering
R&D: Research and development
RS: Raman spectroscopy
SAED: Selected-area electron diffraction
SANS: Small angle neutron scattering
SCL: Space charge layer
SEI: Solid-electrolyte interface
SEM: Scanning electron microscopy
SPM: Scanning probe microscopy
SSB: Solid-state battery
SSE: Solid-state electrolyte
ss-NMR: Solid-state NMR
SSPE: Solid-state polymer electrolyte
STEM: Scanning transmission electron microscopy
SWCNT: Single-walled carbon nanotube
TEM: Transmission electron microscopy
TGA: Thermogravimetric analysis
ToF-SIMS: Time-of-flight secondary ion mass spectrometry
trEFM: Time-resolved electrostatic force microscopy
TXM: Transmission X-ray microscopy
UPS: Ultraviolet photoelectron spectroscopy
WT: Wavelet transform
XANES: X-ray absorption near-edge structure
XAS: X-ray absorption spectroscopy
XCT: X-ray computed tomography
XPS: X-ray photoelectron spectroscopy
XRD: X-ray diffraction
